# Transcriptome and gene expression analysis of three developmental stages of the coffee berry borer, *Hypothenemus hampei*

**DOI:** 10.1038/s41598-019-49178-x

**Published:** 2019-09-05

**Authors:** Daniel D. Noriega, Paula L. Arias, Helena R. Barbosa, Fabricio B. M. Arraes, Gustavo A. Ossa, Bernardo Villegas, Roberta R. Coelho, Erika V. S. Albuquerque, Roberto C. Togawa, Priscila Grynberg, Haichuan Wang, Ana M. Vélez, Jorge W. Arboleda, Maria F. Grossi-de-Sa, Maria C. M. Silva, Arnubio Valencia-Jiménez

**Affiliations:** 1 Department of Cellular Biology, University of Brasília, Brasília-DF, Brazil; 2 Embrapa Genetic Resources and Biotechnology, Brasília-DF, Brazil; 3 Departamento de Ciencias Biológicas, Universidad de Caldas, Manizales, Colombia; 4 Biotechnology Center, UFRGS, Porto Alegre-RS, Brazil; 5 Departamento de Producción Agropecuaria, Universidad de Caldas, Manizales, Colombia; 6 University of Nebraska-Lincoln, Nebraska, United States of America; 7 Centro de Investigaciones en Medio Ambiente y Desarrollo – CIMAD, Universidad de Manizales, Manizales, Caldas, Colombia; 8 Catholic University of Brasília - Postgraduate Program in Genomic Sciences and Biotechnology, Brasília-DF, Brazil

**Keywords:** RNA sequencing, RNA sequencing, Transcriptomics, Transcriptomics, Entomology

## Abstract

Coffee production is a global industry valued at approximately 173 billion US dollars. One of the main challenges facing coffee production is the management of the coffee berry borer (CBB), *Hypothenemus hampei*, which is considered the primary arthropod pest of coffee worldwide. Current control strategies are inefficient for CBB management. Although biotechnological alternatives, including RNA interference (RNAi), have been proposed in recent years to control insect pests, characterizing the genetics of the target pest is essential for the successful application of these emerging technologies. In this study, we employed RNA-seq to obtain the transcriptome of three developmental stages of the CBB (larva, female and male) to increase our understanding of the CBB life cycle in relation to molecular features. The CBB transcriptome was sequenced using Illumina Hiseq and assembled *de novo*. Differential gene expression analysis was performed across the developmental stages. The final assembly produced 29,434 unigenes, of which 4,664 transcripts were differentially expressed. Genes linked to crucial physiological functions, such as digestion and detoxification, were determined to be tightly regulated between the reproductive and nonreproductive stages of CBB. The data obtained in this study help to elucidate the critical roles that several genes play as regulatory elements in CBB development.

## Introduction

Coffee (*Coffea* spp.) is one of the most traded commodities in the world and is the second-most consumed beverage, with more than 9 million tons being consumed annually^1^. Currently cultivated in over 70 countries, the coffee industry is valued at approximately 173 billion US dollars, representing an important source of employment around the world^2^. Crop yield is reduced by more than 30 species of insect pests^3^. Among these pests, the coffee berry borer (CBB), *Hypothenemus hampei* (Ferrari, 1867) (Coleoptera: Curculionidae), is considered the most damaging pest for the coffee industry, causing annual global losses in excess of 500 million US dollars^4^.

The CBB life cycle occurs within the coffee seed, with the adult female laying eggs in galleries formed throughout the endosperm. After hatching, the larvae feed on the coffee seed, reducing grain quality and increasing seed susceptibility to pathogen attack^5^. The endophytic behavior of CBB limits the use of traditional management methods due to poor cost-effectiveness, biosafety concerns and the development of insect resistance^4^. Biological control and volatile repellents have shown moderate efficiency for CBB management under specific field conditions. However, the success of these strategies remains limited when applied under a wide range of environmental conditions^6,7^. A number of alternatives have been proposed for CBB control based on biotechnological approaches. Molecules with known entomotoxic activity, such as the *Bacillus thuringiensis* (Bt) proteins Cry1Ba and Cry3Aa, have been successfully tested against *H*. *hampei* first instar larvae^8^. Furthermore, molecules with inhibitory effects on digestive enzymes have been shown to generate toxic effects in CBB by reducing the enzymatic activity of endogenous alpha-amylases^9,10^ and inhibiting endopeptidases^11^. More recent technologies for crop protection, such as the highly specific RNA interference (RNAi) mechanism, have shown promising results for the management of coleopteran pests^12^.

In addition to the application of RNAi in pest control, the discovery of RNAi has also enabled the identification of gene function with high accuracy^13^. Crop protection using this technology has been well-studied through the administration of double-stranded RNA (dsRNA) molecules, which trigger specific degradation of homologous mRNA in the target insect. Silencing genes with essential functions has been suggested to be a useful approach to ensure insect mortality, thereby increasing host plant resistance to such pests^14–16^. For more detailed information about RNAi mechanisms and their application in pest management, we recommend a recent review^17^.

‘Omic’ technologies are important tools not only for molecular, genetic and cellular research on insects but also for identifying and selecting target genes for RNAi^12,18^. The availability of sequence databases is very useful for a better understanding of host-pathogen interactions^19^, feeding mechanisms^20^, development^21^, and defense pathways in phytophagous insects^22^. Additionally, a significant number of studies using transcriptomic-based approaches have focused on identifying genes involved in developmental processes, with expression profiles among different life stages reported in several insect species^21,23,24^. Recent studies have reported such gene expression analyses across different developmental stages in lepidopteran pests of global economic relevance^24–26^. Similarly, a number of such studies have been performed on coleopteran pests^21,27,28^. Advances have also been described with regard to the expression patterns of chemosensory-related proteins during nonreproductive and reproductive phases and the differences associated with sex differentiation^26,28^. Despite such recent investigations, a gene expression analysis across different life cycle stages has not been performed to date in *H*. *hampei*.

The female genome sequence database is a valuable resource for CBB with a genome size of 163 Mb containing more than 19,000 predicted protein-coding genes^29^. RNA-seq data and genome sequencing of CBB have enabled the identification of important features, including horizontal gene transfer events, a significant repertoire of antimicrobial peptides, noticeable expression of genes encoding glycosidases and characterization of different detoxification pathways, including caffeine detoxification enzymes^29^. Nevertheless, no studies to date have assessed how the expression of these genes varies across the CBB developmental stages. In this work, we present the sequenced transcriptome of three different developmental stages of *H*. *hampei*. Using a *de novo* assembly method, we obtained a set of 53,978 transcripts clustered in 29,434 unigenes. Ultimately, 4,664 of these transcripts were found to be differentially expressed, with genes with known essential functions for insect fitness being highlighted as potential RNAi targets.

## Results

### Sequencing and *De Novo* transcriptome assembly

Illumina sequencing of nine *H*. *hampei* libraries (female, male and L2 larva; three replicates per stage) yielded a total of 265.3 million paired-end (PE) reads, totaling ~66.8 Gbp. The library size ranged from 16.3 to 49.2 million PE reads. Reads below a quality score of 28 (~24%) were removed, with a total of 201.8 high-quality PE reads being submitted to digital expression analysis. Additionally, only 95.1 million high-quality PE reads (~47%) were used for transcriptome assembly after digital normalization. A *de novo* approach was employed for transcriptome assembly, as the percentage of accurately mapped reads on the available reference genome^29^ was too small (<40%) to enable robust expression analysis. Clean reads were assembled into 116,587 contigs. A total of 56,738 (48.7%) contigs were identified as putatively homologous based on BLAST alignment against the NR database. After filtering noncoding sequences and 2,732 (2.4%) contaminant sequences (Supplementary File S1), a final assembly of ~106.6 Mbp was obtained, containing 53,978 contigs and 29,434 unigenes (longest isoform). The assembly quality showed 98.9% completeness. A more detailed list with the assembly statistics is given in Table 1. This transcriptome assembly project has been deposited at GenBank (NCBI database) under the submission number SUB4491034. In addition to transcriptome data, the genome size of CBB females was estimated by flow cytometry at 2n = 400 Mbp (Supplementary Fig. S1).

**Table 1:** Transcriptome assembly summary statistics.

Assembly Statistic	Prefiltered assembly*		Postfiltered assembly	
Number of Unigenes	80,046		29,434	
Number of Contigs	116,587		53,978	
GC %	34.99		35.77	
Completed BUSCOs			1,054 (98.9%)	
Single-copy BUSCOs			536 (50.3%)	
Duplicated BUSCOs			518 (48.6%)	
**Contigs**	**Contigs**	**Unigenes**	**Contigs**	**Unigenes**
Median (bp)	705	573	1,549	1,114
Mean (bp)	1,258.25	970.67	1,975.49	1,609.92
Total assembled bases:	146,695,858	77,697,918	106,632,933	47,386,471
Contig N10 (bp)	5,626	4,739	6250	5735
Contig N30 (bp)	3,288	2,546	3948	3551
Contig N50 (bp)	2,079	1,388	2795	2427

*Prefiltered assembly includes noncoding and contaminant sequences.

### Functional annotation

**Figure 1:**
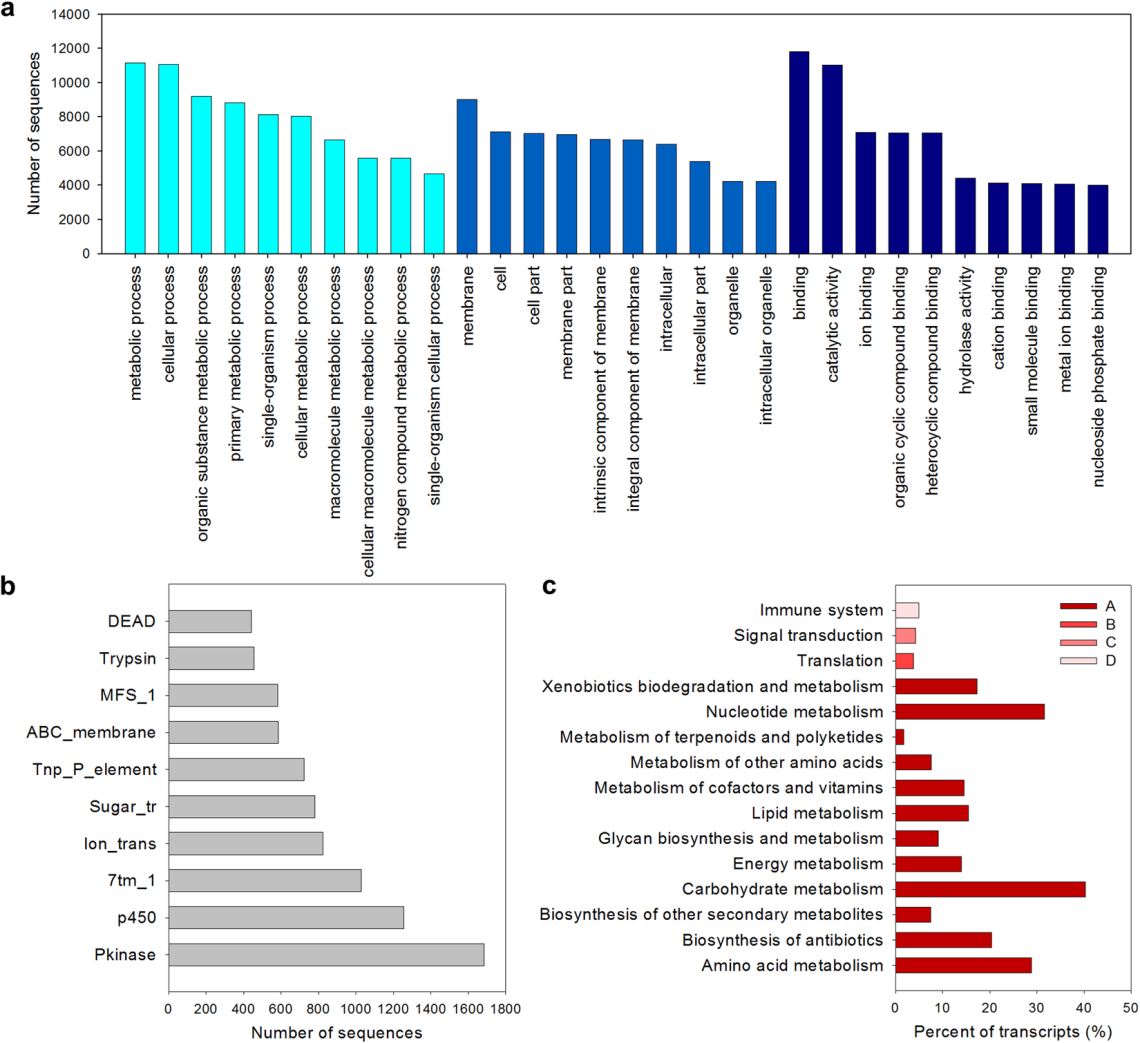
Summary of *H*. *hampei* transcript classification according to the Gene Ontology (GO), protein families (Pfam) and Kyoto Encyclopedia of Genes and Genomes (KEGG) databases. (**a**) The GO classification summarizes a multilevel distribution of the top ten annotated terms for each of three main GO categories (cellular component, molecular function, and biological process). (**b**) Top ten most abundant domains from the Pfam classification. (**c**) Enzyme classification showing the main pathways (y-axis) from four functional categories (metabolism, genetic information processing, environmental information processing and organismal systems) and the percent of transcripts in reference to the total number transcripts annotated using the KEGG database (x-axis). The numbers to the right of the bars represent the total number of transcripts annotated for each pathway. (A) Metabolism, (B) genetic information processes, (C) environmental information processes, and (D) organismal systems.

Functional annotation was performed to provide an overview of protein-coding sequences among the assembled CBB contigs and to predict gene functions associated with each contig. All transcripts from the final assembly were annotated against the NR, InterPro, GO, Pfam and KEGG databases. For the 53,978 contigs annotated in the NR database, more than 75% of the hits for top-hit species were against coleopteran species. Furthermore, when comparing our transcripts with the data available in the CBB genome database, 51,048 (94.6%) transcripts had at least one hit with the protein sequences predicted from the genome, and 16,252 (84.5%) of the 19,222 predicted proteins were aligned to at least one of our transcripts. As a result, *H*. *hampei* was the organism with most number of hits in blast analysis, *Dendroctonus ponderosae* was the 2^nd^ top-hit species with 29,502 (54.4%) annotated contigs and *Tribolium castaneum* was the 3^rd^ top-hit species with 11,245 (20.7%) annotated contigs (Supplementary Fig. S2). For annotation, a total 45,759 GO terms (1,176 single GOs and 1,888 single GOs) were distributed into 98 functional categories according to GO classification, all belonging to the three main GO categories: biological process (50 subcategories), molecular function (21 subcategories), and cellular component (27 subcategories), which comprised 17,036, 20,101 and 14,739 contigs, respectively (Fig. 1a). Additionally, a total of 19,027 (35.2%) contigs within 8,611 (29.3%) unigenes were annotated with at least one Pfam code. The three protein family domains with most associated sequences were protein kinase (PF00069), with 1,683 contigs (8.8%), cytochrome p450 (PF00067), with 1,256 contigs (6.6%) and 7-transmembrane receptors type 1 (PF00001), with 1,029 contigs (5.4%) (Fig. 1b). Finally, 2,685 contigs (1,499 unigenes) were associated with at least one enzyme code following KEGG database annotation. Overall, 344 enzymes were identified and classified into 121 metabolic pathways (Fig. 1c).

### Digital gene expression profiling

**Figure 2:**
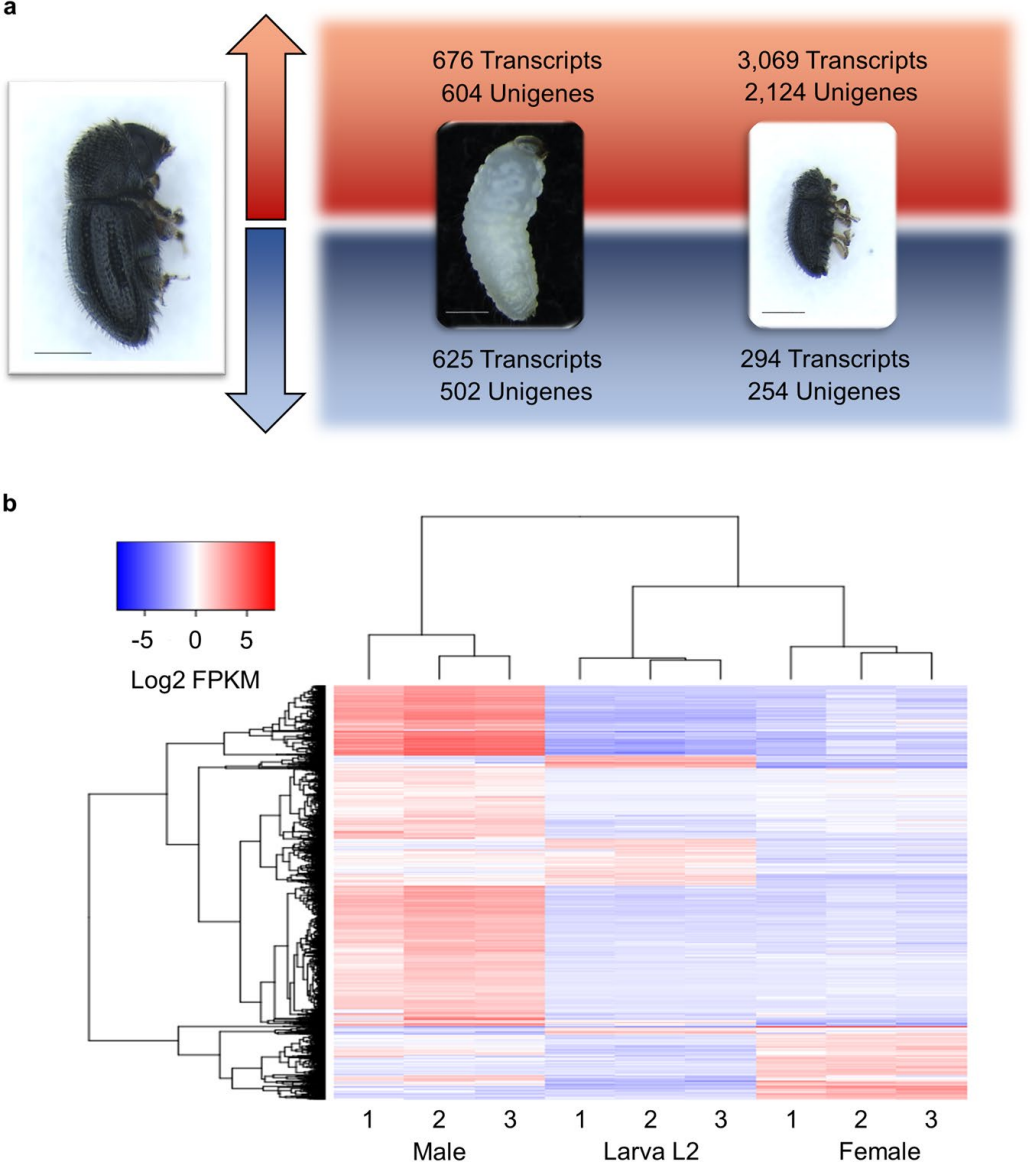
Pairwise comparisons of differentially expressed genes between the different developmental stages of *H*. *hampei*. (**a**) Number of transcripts and unigenes differentially expressed in L2 larvae and males in reference to females. Red and blue arrows represent upregulated and downregulated genes, respectively. The scale bar shown in insect pictures corresponds to 500 µm. (**b**) Heatmap and clustering of all DEGs. Rows represent single transcripts, and columns represent the replicates for each developmental stage. Genes were clustered based on expression similarity (Euclidean distance). Blue and red colors in the heatmap correspond to low and high relative gene expression, respectively.

An average of 73.2% of high-quality filtered reads (ranging from 68.9% to 75.3% across libraries) were successfully mapped against our final assembly to enable FPKM-based analysis of expression values for transcripts for each developmental stage. All three replicate libraries for each developmental stage showed a high correlation (Pearson r = 0.86–0.99) and were used for expression analysis. A total of 4,664 transcripts (3,484 unigenes) were differentially expressed across the three evaluated developmental stages of *H*. *hampei* (Fig. 2a). Using the female life stage as a reference, 676 and 625 transcripts were upregulated and downregulated, respectively, in L2 larva. In contrast, 3,069 and 294 transcripts were upregulated and downregulated, respectively, in males. Furthermore, among all the DEGs, a total of 316 transcripts comprising 162 upregulated and 154 downregulated transcripts were differentially expressed in both male and L2 larva compared to expression in the female. Four main sets of DEGs (L2 larvae upregulated, L2 larvae downregulated, male upregulated and male downregulated) were generated (Fig. 2b). The majority of the DEGs were expressed with fold change values >2 and ≤5.5 (36.6). In contrast, DEGs showing values >12.5 and ≤15.6 were less abundant (1.9%). A plot showing the distribution of fold change values is presented in Supplementary Fig. S3. Surprisingly, 803 DEGs (17.2%) were “incompletely” annotated, matching only genes associated with unknown functions, hypothetical proteins and uncharacterized proteins, thereby indicating the need for improved annotation and genetic resources for the CBB.

### Gene ontology enrichment analysis

**Figure 3:**
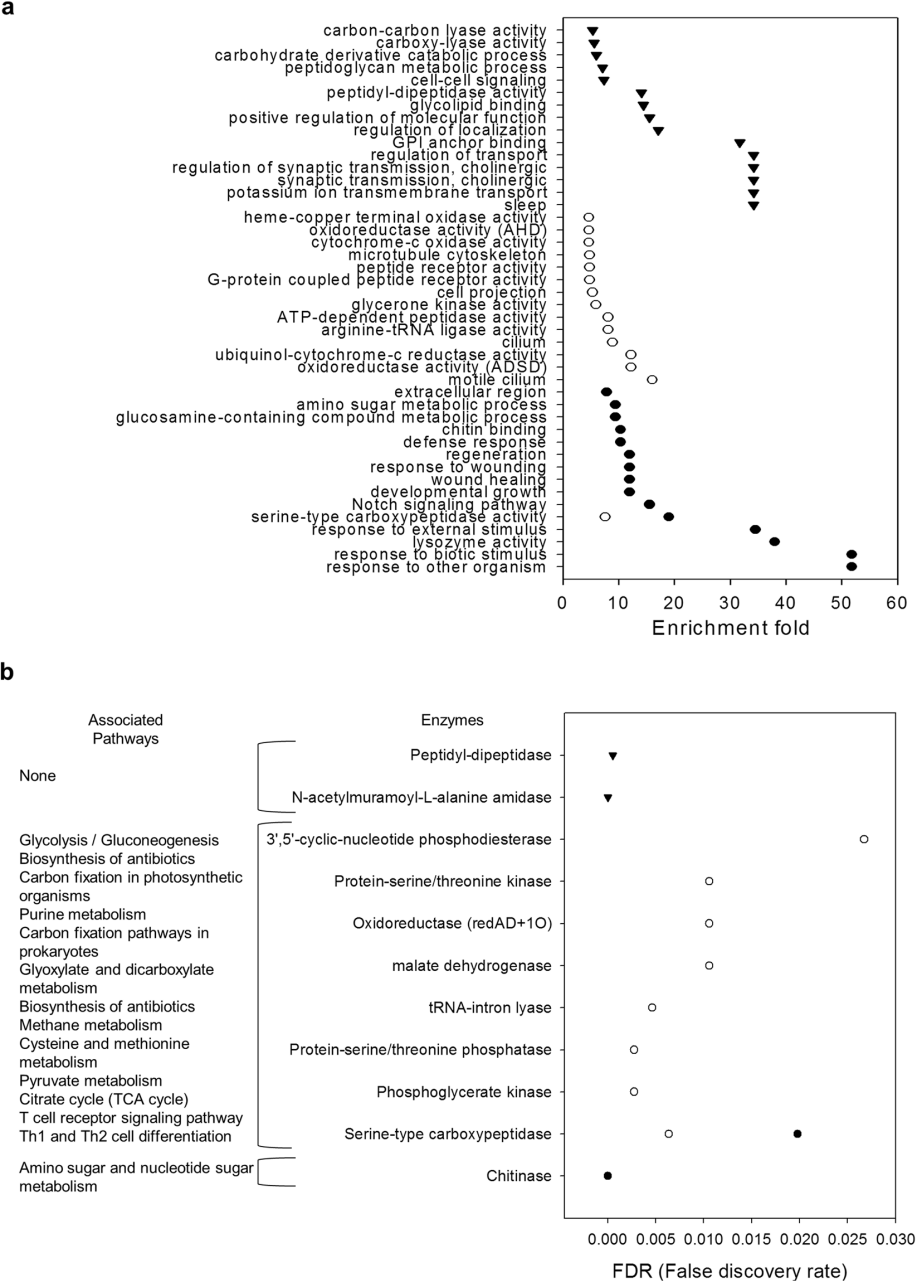
Gene ontology and enzyme enrichment analysis for each *H*. *hampei* set of DEGs. (**a**) Top 15 GO terms with the highest enrichment values for each pairwise comparison. (**b**) False discovery rates for each enzyme and the associated pathways for each pairwise comparison in the enrichment analysis. Black circles indicate transcripts upregulated in larvae. White circles indicate transcripts upregulated in males. Black triangles indicate transcripts downregulated in larvae.

GO enrichment analysis was performed separately for all four sets of DEGs. After filtering redundant GO terms, a total of 117 GO terms were associated with all the DEG sets, of which 32, 24, 61 and 0 were enriched in the L2 larva upregulated, L2 larva downregulated, male upregulated and male downregulated sets, respectively. Additionally, 8 GO terms were identified to be shared between the L2 larva upregulated and male upregulated sets. The most significant GO terms for each set of DEGs across the different developmental stages are shown in Fig. 3a (full list provided in Supplementary File S2). Interestingly, genes related to drug metabolism, chitin metabolism, proteolysis, and cuticle regeneration were upregulated in both L2 larvae and males. Genes related to developmental processes, carbohydrate metabolism, defense responses and cell signaling pathways were upregulated only in L2 larva. Additionally, genes related to transport processes, neuronal activity, sleep behavior and vitamin binding were downregulated only in L2 larva.

### Enzyme enrichment

Enzyme enrichment was performed to identify highly specific metabolic pathways associated with each developmental stage. Overall, 15 pathways were associated with 12 enzymes that showed significant enrichment (FDR > 0.05) in our DEG sets. Interestingly, most of the pathways (14) were associated with the male upregulated set, while the remaining pathway was associated with the L2 larva upregulated set (Fig. 3b). On the other hand, no enzyme enrichment was observed for the male downregulated set.

### DEGs between males and females

**Figure 4:**
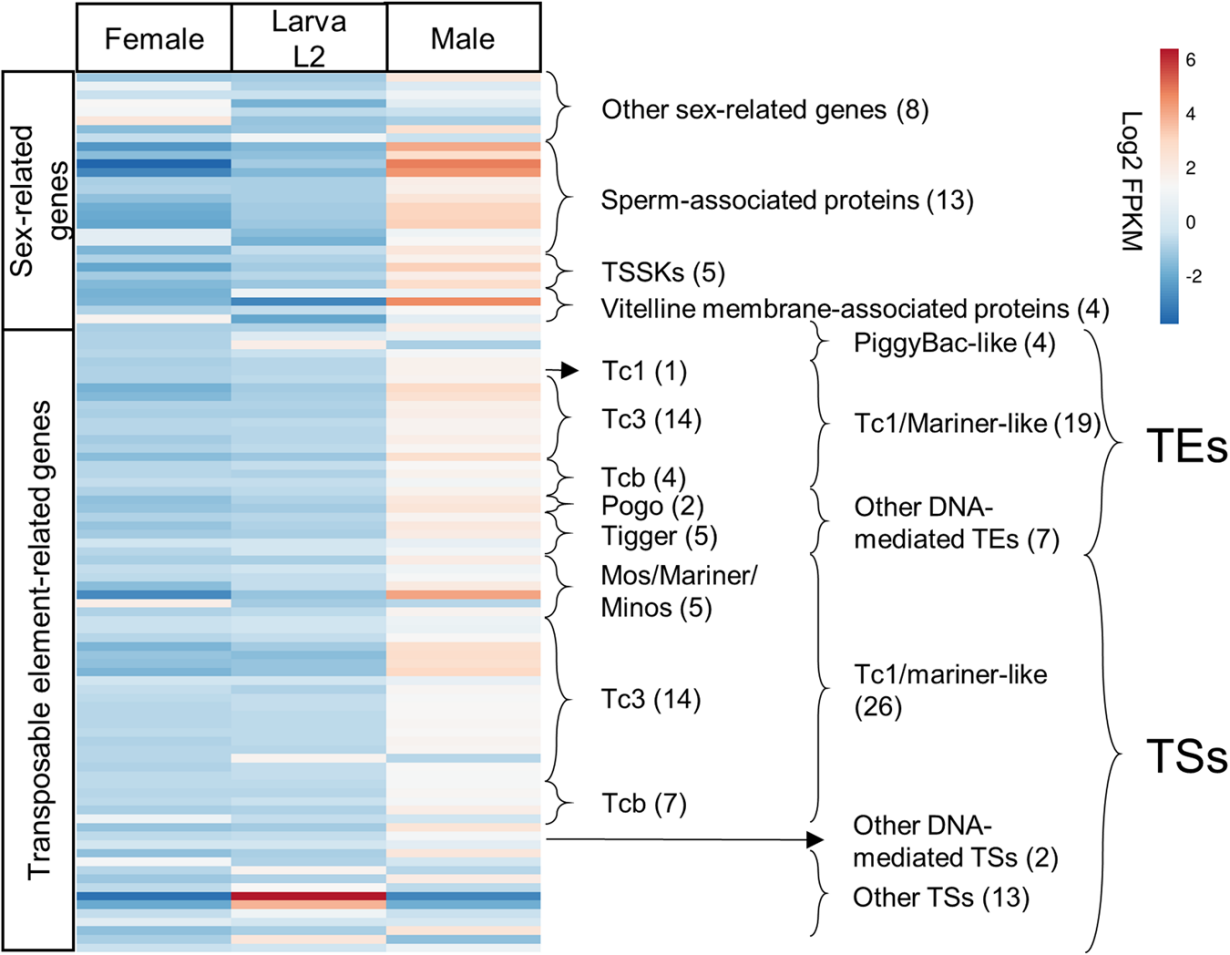
Heatmap showing expression differences between *H*. *hampei* libraries for female CBBs, CBB L2 larva and male CBBs for genes encoding TE-related proteins and sex-related proteins. TEs: transposable element-like proteins, TSs: transposase-like proteins.

From the total number of unigenes that were differentially expressed, 2,124 unigenes (68.3% of total DEGs) were upregulated in males, of which 1,979 (56.8% of total DEGs) were exclusively upregulated in males and 145 were upregulated in L2 larvae. For a more detailed analysis of this predominant group of DEGs, unigenes associated with enriched GO terms/pathways were grouped by gene function (Fig. 4). Notably, transposable element-related proteins (TEs) and reproductive organ-related proteins were mainly upregulated in males. Among the TE-related proteins, genes encoding 41 transposases (TSs) and 20 TE-derived proteins were identified as DEGs. Among the TEs and TSs, those belonging to the Tc1/mariner superfamily were the most abundant, with 19 and 26 DEGs, respectively, being identified.

### DEGs between larva and adult stages

**Figure 5:**
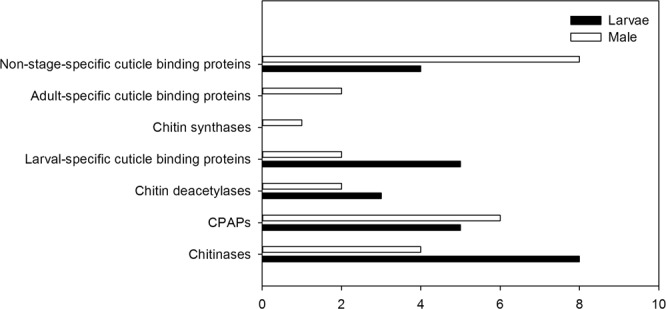
Number of unigenes (x-axis) upregulated in *H*. *hampei* L2 larva and male in reference to female for each group of genes related to cuticle and chitin metabolism (y-axis). CPAPs: cuticular proteins analogous to peritrophins.

**Figure 6:**
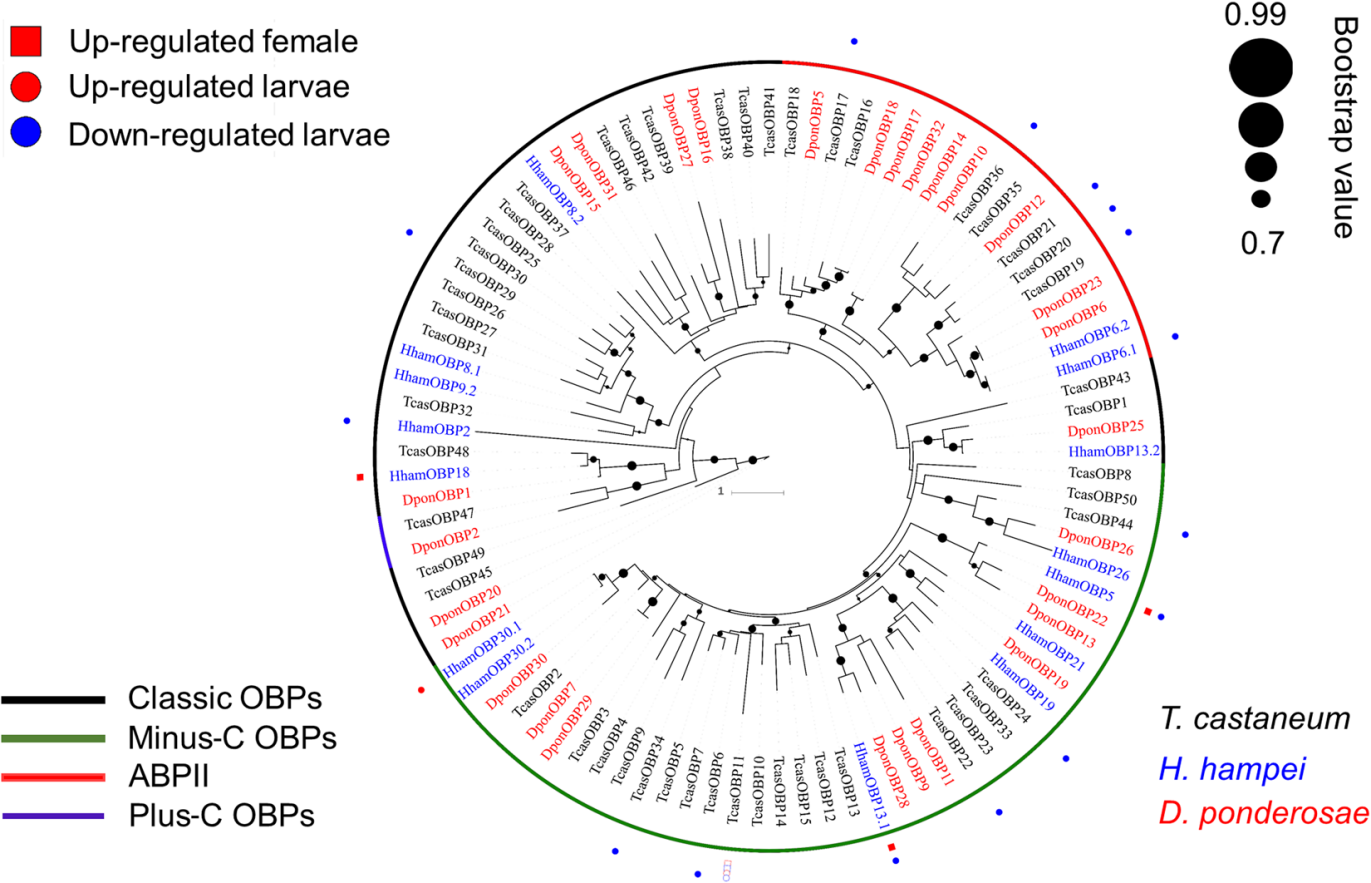
Phylogenetic relationship between *H*. *hampei* OBP candidate genes and other coleopteran OBPs and DEGs observed in *H*. *hampei* and *T*. *castaneum*.

We identified several differentially expressed genes that could be key elements for understanding the physiological and metabolic differences between reproductive and nonreproductive stages of the CBB by comparing L2 larva and female libraries (Supplementary Table S1). Genes annotated with cytochrome p450 (CYP) detoxification family domains were both upregulated (5 unigenes) and downregulated (7 unigenes) in the larva. Interestingly, for cuticle metabolism-related genes, all DEGs were upregulated in larvae and males (25 unigenes each). The DEGs that were annotated with functions related to cuticle and chitin metabolism were grouped according to their predicted proteins and are shown in Fig. 5. Finally, chemosensory candidate genes belonging to the Odorant Binding Protein (OBP) family (Fig. 6) were clustered in different classes using phylogenetic analysis. Most of the OBP genes were upregulated in adults (4), with two of them being exclusively expressed in females and belonging to the minus-C OBP class. Additionally, the only OBP upregulated in larvae was grouped within this class. No OBPs were upregulated in males compared with females.

### RNA-seq data validation by real-time quantitative reverse transcription PCR (RT-qPCR)

**Figure 7:**
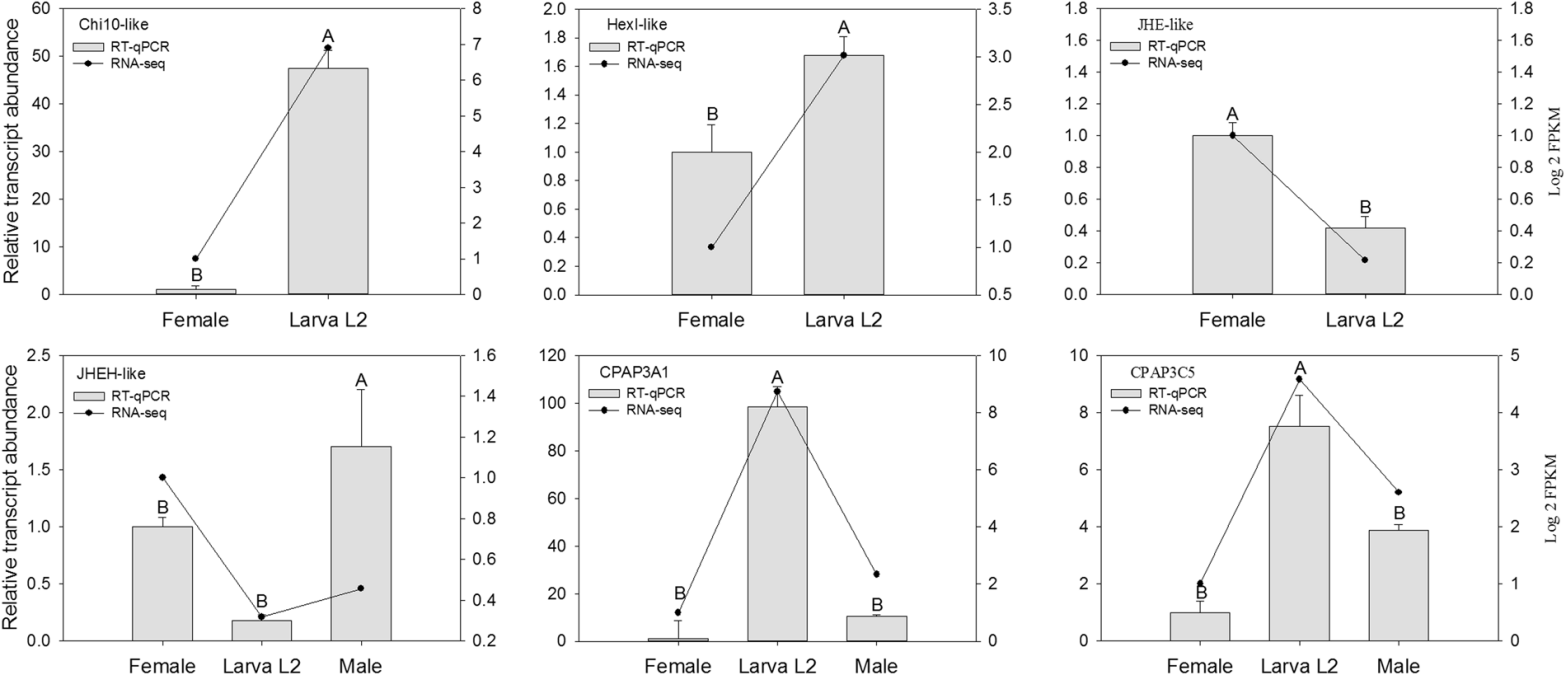
RT-qPCR results for *H*. *hampei* differentially expressed genes across different developmental stages of the CBB. Different capital letters denote a significant difference between stages (P < 0.05). JHE: juvenile hormone esterase, JHEH: juvenile hormone epoxide hydrolase, CPAP: cuticular protein analogous to peritrophins, HexI: hexosaminidase I, Chi10: chitinase 10.

RT-qPCR was conducted to validate the differentially expressed transcripts identified by DEG analyses of RNA-seq data. Six genes related to developmental processes were validated: juvenile hormone (JH)-related enzymes (JH esterase and JH epoxide hydrolase), cuticular proteins analogous to peritrophins (CPAPs) (CPAP3A1 and CPAP3C5) and proteins involved in chitin metabolism (hexosaminidase I and chitinase 10). Overall, the RT-qPCR results were consistent with the RNA-seq data with a correlation value of 0.79 for the entire set of genes that were validated (Fig. 7).

## Discussion

Multiple studies have sought to identify alternative methods for CBB management based on the physiological, ecological and behavioral characteristics of this damaging pest^30–32^. However, few studies have described the molecular features of CBB (*i*.*e*., its genome structure, transcription profiles or proteome)^29,33^. Transcriptomics is a useful approach for providing complementary data for available genomes, as well as gene expression data, for an organism under a specific condition or developmental stage. Considering that sequencing insect transcriptomes can enable the complete identification of novel genes, with functional characterization of large datasets of genes and transcript abundance, this approach can provide valuable information for insect pest control strategies. In addition to the transcriptomes of well-characterized model organisms, such as *T*. *castaneum*^34^ and *D*. *ponderosae*^35^, the transcriptomes of different developmental stages of other coleopteran insects, including *Cylas formicarius*^21^, *Batocera horsfieldi*^36^ and *Cyrtotrachelus buqueti*^27^, have been recently sequenced, increasing sequencing data that are relevant for research into many other insect targets.

In this study, we used high-throughput sequencing to generate a 47.4-Mbp assembly of 29,434 unigenes, which is considerably larger than the number predicted in the draft genome of the CBB (~28.4 Mbp) with a total of 19,222 predicted genes, of which only 15,546 were expressed in females^29^. Additionally, the predicted CBB genome size determined by flow cytometry (~200 Mbp) was slightly higher than that described for the CBB draft genome (~163 Mbp)^29^. Although the number of sequences in our transcriptome is higher, approximately 15% of the CBB genome predicted proteins were not aligned to any of our transcripts. Those genes could be only expressed in phases, such as pupa and eggs, that were not sequenced in this work or even expressed in specific physiological conditions. Otherwise, we found that 2,930 transcripts from our assembly did not have a single hit with the predicted proteins of the CBB genome. It is important to note these transcripts as possible new genes that were not identified in the currently available genome. Another fact supporting the need to have sequenced the presented transcriptome is that the N50 of our assembly (2,427) is almost 1.5 times higher than the current transcriptome (1,638), also published by Vega *et al*.^29^. Thus, we provide a transcriptome with larger fragments than currently published^29^. It is important to highlight that the current versions of both the genome and transcriptome of the CBB were obtained by sequencing RNA from individuals with more than 10 years of meridic diet in the laboratory. It is expected that the variability observed in such a population should be different from the variability observed in the population used in the present work, which was obtained from insects raised in parchment coffee for only one generation. Therefore, when comparing transcriptomes, our data set is a more realistic and comprehensive representation of the gene set and transcript expression patterns in the CBB, since the population used in this work was maintained in conditions closer to the natural conditions of field populations of the CBB.

In this report, we present additional information complementary to the draft genome reported by Vega *et al*.^29^, including not only sequences obtained from female but also from male and larval phases. In our dataset, 3.7% and 8.1% of genes were differentially expressed between L2 larvae and females and between females and males, respectively. Previous reports have shown that the percentage of DEGs between nonreproductive and reproductive stages (1–5.5%) is slightly lower than the percentage of DEGs between sexes in the adult stage (2.7–8.8%) in other coleopteran species^21,27,36^. An important factor to be considered in future studies is that CBB males only exhibit one instar in the larval stage, while females have two instars. We observed greater differences in gene expression when comparing males and females or males and L1 larvae than when comparing males and L2 larvae, since the last larval stage is a female-specific stage^37^. In this work, we focus not only on identifying target genes specific for larval instars but also for females. Early stages are usually the most appropriate targets for the management of insect pests. However, due to the endophytic behavior of the CBB’s early stages, this approach severely limits the method used for control. In the case of larvae, transgenic plants should be more efficient. Otherwise, nontransformative methods could suit better when applied to females, which is the only stage of the CBB that leaves the endosperm. Hence, only females are exposed to control methods that are not able to permeate the endosperm of the coffee bean. Thus, understanding the expression profile and RNAi target identification in larval and adult stages of the CBB is equally important.

From a functional perspective, we observed that for both the GO and the pathway enrichment analysis, the upregulated genes in males were enriched with more functional categories (80 GO terms and 14 KEGG pathways) than any other DEG set. This finding is critical to improve understanding of sex determination in the CBB, in which the sex ratio is distorted towards females with a 10:1 ratio^5^. The concept of male haploid (arrhenotoky) has been used to explain sex determination in the CBB, and linkage between haplodiploidy and insecticide resistance has been proposed^38^. However, the genetics associated with the life history of CBB are poorly understood, and the specific genes or metabolic pathways involved in sex determination remain unknown. In this work, we showed that over 68% of the DEGs were upregulated in males, with DEGs associated with TEs and sex determination highlighted. Hernandez-Hernandez *et al*.^39^ characterized the TEs present in the CBB genome, revealing that 11.51% of the TEs belong to the Tc1/mariner superfamily. Furthermore, the authors confirmed that at least 8.2% of the CBB genome is composed of TEs. In this study, we identified 724 transcripts annotated with the TE Pfam family (Fig. 1b) and 1,367 unigenes with at least one match to TE-related proteins in BLAST analysis. Thus, at a minimum, 2.5% of the TE-related proteins are expressed in the CBB transcriptome, of which 4.7% are differentially expressed genes. As discussed by Hernandez-Hernandez *et al*.^39^, insect reproductive characteristics may be related to low TE content. Haplodiploidy, in which a chromosome set from males is not transmitted to the next generation, prevents the spread of certain TE genes. This fact may explain the higher abundance of genes encoding TE-related proteins in the male mRNA libraries reported in this work.

In addition to the comparison of expression between males and females, comparing the reproductive and nonreproductive stages also revealed interesting gene expression variation across the CBB life cycle. The larva-to-adult transition and developmental regulation in holometabolous insects are critical, not only from an evolutionary and ecological perspective but also from the perspective of developing sustainable methods for insect pest management^40^. This study describes expression differences between the adult and immature stages of CBB and identified genes that were previously detected in the genome of this insect that could be linked to insect development^29^. As shown in both GO and pathway enrichment analyses, several genes related to carbohydrate and protein metabolism were differentially expressed in larvae compared to their expression in females. Overall, most of the predicted proteins involved in cellulose and hemicellulose metabolism, such as endoglucanases, cellulases (glycoside hydrolase family 48) and xylanases, were upregulated in larva. Interestingly, these enzymes have been reported to digest plant cell wall components in many coleopteran species, specifically cellulose, hemicellulose and lignocellulose, as their main carbohydrate sources^41^.

Moreover, genes encoding enzymes with protease activity were upregulated in larvae (serine proteases and thiol proteases) and females (peptidyl-dipeptidases and metalloproteases). Several studies have shown higher expression of digestive enzyme-coding genes in larval stages, probably because these nonreproductive stages, often called the feeding stages, involve a more active feeding behavior than observed in adults^21,25^. Digestive enzymes are considered potential targets for insect pest management, as their function in insect nutrition is essential, and their inhibition can cause lethal or deleterious effects in the larval stages^42^. However, it has been reported that insect genes rapidly evolve in response to inhibitors targeting digestive enzymes^43^, meaning that the identification of novel variants of these genes could be crucial in designing more precise and efficient strategies for pest control^44,45^.

Other genes that can be considered potential targets for RNAi and insecticide development are those encoding cuticular proteins (CPs) and enzymes involved in chitin metabolism. In insects, the structure of the cuticle is determined by cuticular proteins, which play roles in many physiological processes, such as protection against dehydration, insecticide penetration, pathogens and physical injury^46^. Therefore, CPs could be used as potential targets for the management of insect pests^47^. Some genes encoding cuticular binding proteins and cuticular proteins analogous to peritrophins (CPAPs) were differentially expressed in our data set, with higher expression being observed in larvae and males than in females (Fig. 5). Similarly, certain chitin synthases, chitinases and chitin deacetylases showed higher expression in larvae and males than in females. Interestingly, genes involved in chitin degradation appeared upregulated to a greater extent in males compared to larvae or females. Precise control of chitin synthesis and degradation rates is essential for insect homeostasis. Determining the expression profiles of genes that regulate these rates could be useful in the design of pest control strategies^48^.

Finally, we highlighted a group of genes encoding proteins with chemosensory functions. The multigenic family of OBPs is thought to be involved in chemical recognition, which regulates pivotal behaviors, including host choice, copulation, and reproduction^49^. In coleopteran adults, olfaction plays a major role in finding mates, locating oviposition sites, foraging, aggregating and avoiding predators^50^. Despite the importance of the olfaction mechanism, the molecular basis of chemoreception in coleopterans is still poorly understood compared to lepidopterans and dipterans^51,52^. The OBP family can be separated into four different phylogenetic groups: the classic OBPs, which are characterized by six cysteine residues at conserved positions; the minus-C class, which lost two of the conserved cysteine residues; the plus-C OBPs, which contain four to six additional cysteines and one characteristic proline; and the highly specific antennal binding proteins (ABPII)^53^. A few studies conducted in coleopteran pests have evaluated the tissue-specific expression profiles of most of the OBPs described in insects, in some cases showing few differences between male and female tissues. However, little is known about the expression profiles of OBP genes across different developmental stages^28,54^. In this study, we identified 15 full-length candidate genes encoding OBPs, of which six were differentially expressed during different stages. Most of the candidate OBP genes showed the highest expression in adults, mainly in females, and only one of these genes was upregulated in larvae.

Moreover, when we compared the *H*. *hampei* and *T*. *castaneum* OBP profiles^54^ in our phylogenetic analysis (Fig. 6), we observed that no OBP genes were upregulated in *T*. *castaneum* larvae. Additionally, of a total of 16 OBPs that were differentially expressed in *T*. *castaneum* and *H*. *hampei*, eight belonged to the minus-C class. These findings suggest that differences in the expression of OBP genes in Coleoptera species may be determined by the transition to a reproductive stage. Since the CBB males never leave the coffee seeds, it is possible that OBPs with higher expression in females are related to the females’ colonizing role, which involves detecting new and suitable coffee seeds. The role of minus-C OBPs in this process has not been determined to date, but its potential as a target for pest management is of great interest. As proposed by Zhou *et al*. in 2010, interruption of signal perception by blocking OBPs could prevent mating events or affect normal host selection^55^. Indeed, knockdown of OBP genes in recently published work has shown lethal effects in different insect pests^56,57^. In the CBB, OBP knockdown may also show lethal effects because of the CBB’s endophytic lifestyle, in which odor-based communication is considered essential to insect reproduction and survival.

## Methods

### Insects

Second instar larvae and adult females and males of the CBB were collected in 2015 from established laboratory colonies located at the Universidad de Caldas in Manizales, Caldas, Colombia. Insects were obtained from infested coffee plants in the field and then raised in parchment coffee bean. Only insects with one generation raised in the laboratory were collected in 1.5 ml RNase-free tubes with 1 ml of RNAlater stabilization solution (Qiagen, Germany) and then stored at −80 °C until further processing. Samples of 20 mg from the whole insect body were prepared from each developmental stage and used for RNA extraction.

### RNA isolation and Illumina sequencing

Total RNA was isolated from each developmental stage of *H*. *hampei* using the RNeasy Mini Kit (Qiagen, Valencia, CA, USA) according to the manufacturer’s instructions and stored at −80 °C until use. RNA extraction was performed on three biological replicates. RNA integrity was assessed using a 2100 Bioanalyzer system (Agilent Technologies, Santa Clara, CA, USA) and through 1% denaturing agarose gel electrophoresis. RNA concentration was measured using a NanoDrop 1000 (Thermo-Fisher Scientific, Wilmington, DE, USA). Stage-specific samples from RNA extractions were used for cDNA library construction with a TruSeq RNA Library Prep Kit v2 (Illumina, San Diego, CA, USA). Finally, cDNA libraries were purified using a Ribo-Zero rRNA Removal Kit and sequenced in a 125 bp PE run on an Illumina HiSeq^TM^ 2500 platform at the University of Minnesota Genomics Center (MN, USA).

### *De Novo* transcriptome assembly and functional annotation

The raw reads were cleaned using Trimmomatic v0.33 software^58^ for removal of adaptors, sequences containing unknown nucleotides (N over 5%) and low-quality sequences (Phred < 28). Initially, clean reads were mapped against the current CBB genome sequences (https://genome.med.nyu.edu/coffee-beetle/cbb.html). TopHat software was used to map our reads against the genome scaffolds^59^. For the assembly, clean reads were digitally normalized using KHMER v2.0 software^60^ with a maximum coverage of 50 bp for each read. Normalized reads were assembled into longer fragments (contigs) using Trinity v2.0.6 software^61^, which generated a set of components (unigenes) containing a variable number of transcripts (isoforms). Transcripts were BLASTed against the National Center for Biotechnology Information (NCBI) nonredundant protein (NR) database using the blastx algorithm with an e-value cutoff < 1E-5. Additionally, since no RefSeq from the current CBB genome is available in the NCBI database, a BLASTx was performed by aligning our transcripts to the predicted protein sequences in the CBB genome project database (https://genome.med.nyu.edu/coffee-beetle/cbb.html). To remove potential microbial sequences and other contaminants from the assembly, *in silico* curation was performed by manual removal of transcripts that only matched sequences from plants and/or microorganisms in BLAST analysis. The remaining transcripts were annotated using Blast2GO Basic v4.1^62^ by identifying protein domains from the InterPro database^63^, protein families from the Pfam database^64^, GO terms (GOs) from the GO database^65^ and metabolic pathways from the KEGG (Kyoto Encyclopedia of Genes and Genomes) database^66^. Finally, to estimate the genome size of female CBB, flow cytometry was performed following the protocol of Gregory *et al*.^67^.

### Analysis of differentially expressed genes

To compare gene expression profiles across three developmental stages (L2 larva, adult male, and adult female) of *H*. *hampei*, clean reads of each library were mapped to our assembly using RSEM software^68^, and abundance estimation was performed by using the script *align_and_estimate_abundance*.*pl* in the Trinity package. Expression levels of the transcripts were calculated based on the FPKM normalization method using the edgeR package^69^. Comparisons of differentially expressed genes (DEGs) were performed using female libraries as a reference. Female libraries were used as reference with the objective of properly showing the two main comparisons in this work, between sexes (female and male) and between reproductive and nonreproductive phases (female and L2 larva), considering that L2 instar is specific to females in the CBB. The criteria for statistical significance of DEGs were a false discovery rate (FDR) less than 0.001 and a log_2_ (fold change) >2. Finally, GO enrichment analysis was performed for DEG sets using FUNC software^70^. Removal of redundant GO terms was performed in REVIGO software with an allowed similarity value of 0.5^71^. Visualization of the expression profiles was performed using ClustVis software^72^.

### Phylogenetic analysis

Multiple alignments were performed with the full-length amino acid sequences of chemosensory candidate genes belonging to the odorant binding protein (OBP) family. To this end, sequences from two well-characterized genomes of coleopteran pests (*T*. *castaneum* and *D*. *ponderosae*) and those identified in our assembly were used. After the removal of a signal peptide in the N-terminal region (SignalP4.1)^73^, sequences were aligned with MAFFT v7 software^74^ and further edited with trimAL software^75^. Phylogenetic trees were constructed using RAxML v8 software^76^, using the maximum likelihood method with the LG model and 1000 bootstrap replicates. The accession numbers of the OBP sequences from *D*. *ponderosae* and *T*. *castaneum* are listed in Supplementary File S3. The candidate OBP-coding genes from the *H*. *hampei* assembly, along with their BLAST results and FPKM values, are also listed (Supplementary File S4).

### Quantitative real-time PCR (RT-qPCR) validation

To validate data obtained from RNA-seq, total RNA was extracted from different samples of those used in the sequencing but treated under the same experimental conditions. This approach was used to improve the reliability of the expression patterns by considering technical and biological variation. Insect cDNA was synthesized from 1000 ng of RNA using a QuantiTect Reverse Transcription Kit (Qiagen, Valencia, CA, USA) following the manufacturer’s instructions. The RT-qPCR reactions included 2 μl of diluted cDNA (1:50), 5 μl of Fast SYBR^®^ Green Master Mix (Applied Biosystems, Foster City, CA, USA), 0.2 μl of forward and reverse primers at 10 μM, and 2.6 μl of nuclease-free water, for a total volume of 10 μl. Primers were designed using Primer3^77^ and validated by analysis of their PCR amplification efficiencies (E) and correlation coefficients (R2) (Supplementary Table S2). Both primer efficiency tests and RT-qPCR were performed on a 7500 Fast RT-PCR System (Applied Biosystems, Foster City, CA, USA). The RT-qPCR program included a holding stage of 95 °C for 20 s followed by 40 cycles of denaturation at 95 °C for 3 s and annealing/extension at 60 °C for 30 s (cycling stage). A melting curve was generated to confirm the production of a single peak and to rule out the possibility of primer dimers and nonspecific product formation. The expression of the genes was calculated using the comparative 2^−ΔΔCT^ method^78^. Relative gene expression analysis was performed using β-actin as a normalizer.

## Supplementary information

Transcriptome and gene expression analysis of three developmental stages of the coffee berry borer, Hypothenemus hampei

Supplementary Dataset 1

Supplementary Dataset 4

Supplementary Dataset 3

Supplementary Dataset 2

## Data Availability

The datasets generated during the current study are available in supplementary information. Raw sequencing and transcriptome assembly data were deposited in NCBI databases, but restrictions apply to the availability of these data. Data will be released for public access once this paper is published.
