# Supplementary material for: Transcriptome and gene expression analysis of three developmental stages of the coffee berry borer, Hypothenemus hampei
Source: Sci Rep. 2019 Sep 5;9:12804. doi: 10.1038/s41598-019-49178-x (PMC6728347; doi:10.1038/s41598-019-49178-x)
Supplement: Supplementary file 1 — Transcriptome and gene expression analysis of three developmental stages of the coffee berry borer, Hypothenemus hampei [file 41598_2019_49178_MOESM1_ESM.docx]

**Transcriptome and gene expression analysis of three developmental stages of the coffee berry borer, *Hypothenemus hampei***

Daniel D. Noriega^1, 2, §,^ **^*^**, Paula L. Arias^3, §^, Helena R. Barbosa^2, 4, §^, Fabricio B.M. Arraes^2, 4^, Gustavo A. Ossa^3^, , Bernardo Villegas^5^, Roberta R. Coelho^2^, Erika S. Albuquerque^2^, Roberto C. Togawa^2^, Priscila Grynberg^2^, Haichuan Wang^6^, Ana M. Vélez^6^, Jorge W. Arboleda^7^, Maria F. Grossi-de-Sa^2, 8,^ **^*^**, Maria C.M Silva^2, δ^, Arnubio Valencia-Jiménez^5, δ,^ **^*^**

^1^Department of Cellular Biology, University of Brasília, Brasília-DF, Brazil.

^2^Embrapa Genetic Resources and Biotechnology, Brasília-DF, Brazil.

^3^Departamento de Ciencias Biológicas, Universidad de Caldas, Manizales, Colombia.

^4^Biotechnology Center, UFRGS, Porto Alegre-RS, Brazil.

^5^Departamento de Producción Agropecuaria, Universidad de Caldas, Manizales, Colombia.

^6^University of Nebraska-Lincoln, Nebraska, United States of America.

^7^Centro de Investigaciones en Medio Ambiente y Desarrollo – CIMAD, Universidad de Manizales, Manizales, Caldas, Colombia.

^8^Catholic University of Brasília - Postgraduate Program in Genomic Sciences and Biotechnology, Brasília-DF, Brazil

^§^Authors contributed equally to the work.

^δ^ Co-last authors

**
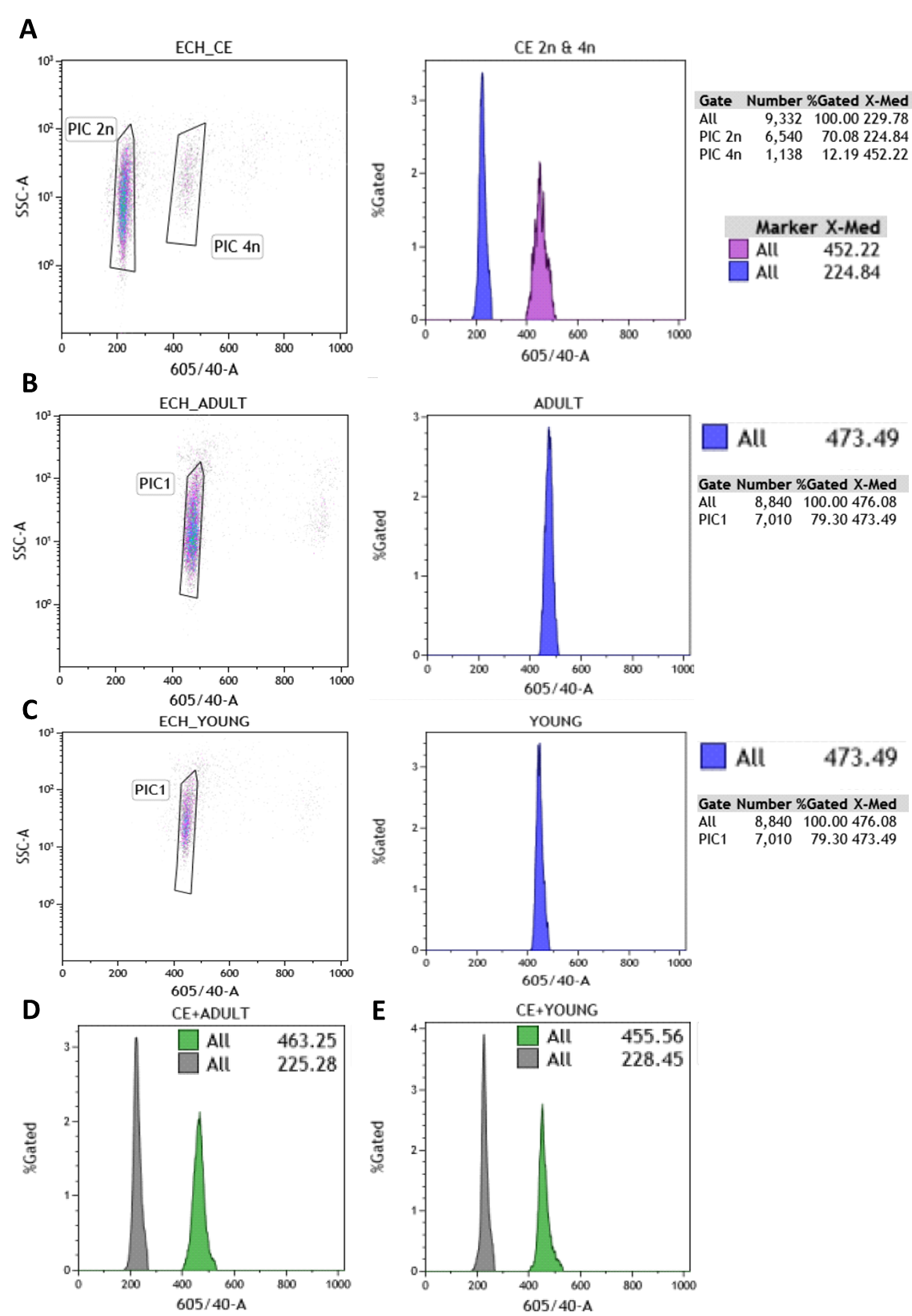
**

**Supplementary Figure S1.** **Flow cytometry estimation of the genome size for *Hypothenemus hampei***. *Caenorhabditis elegans* was used as a reference standard (known genome size 1C=100 Mb). The X-axis represents the relative fluorescence intensity of nuclei stained with propidium iodide in a nuclear suspension from an adult insect. The Y-axis represents the number of nuclei. (**a**) *C. elegans* 2C and 4C. (**b**) *H. hampei* adult female. (**c**) *H. hampei* young female *L. oryzophilus* females. (**d**) and (**e**) are mixed samples of *C. elegans* and *H. hampei* adult or young females, respectively. **Green square**: (CE 4n + CBB 2n) 605\ 40-A. **Grey square**: (CE 2n) 605\ 40-A. **Violet** **square**: (PIC 4n) 605\ 40-A. **Purple square**: (PIC 2n) 605\ 40-A.


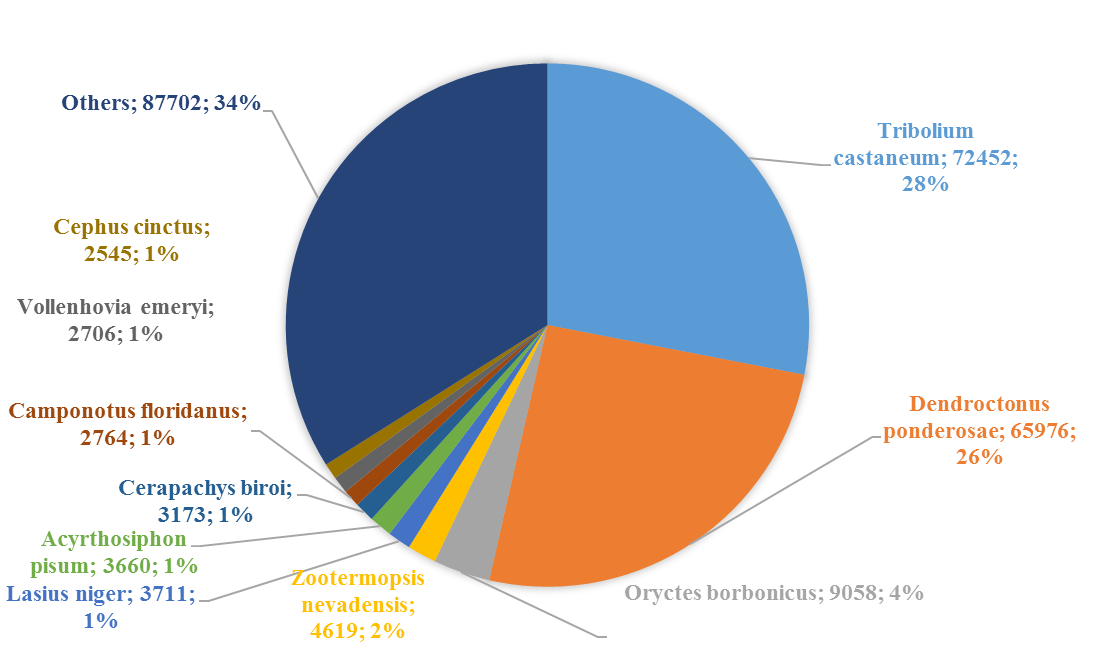


**Supplementary Figure S2.** **Top-hit species distribution for BLAST analysis to the NR database from NCBI.** Right to species name is showed the number of contigs with at least one match and the respective percent.


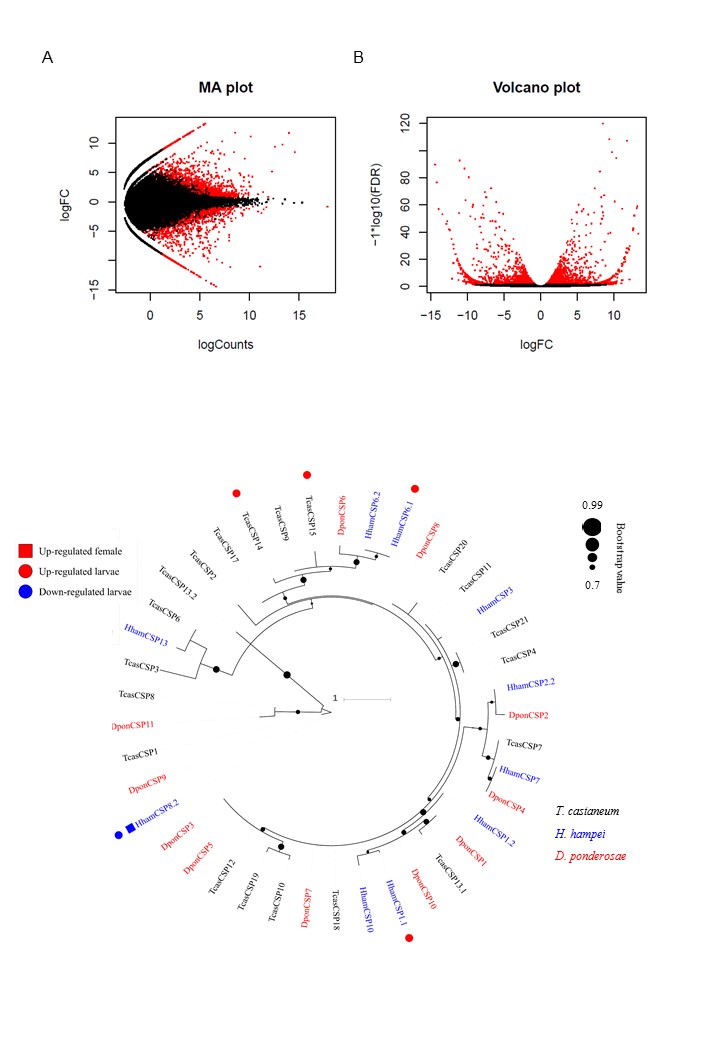

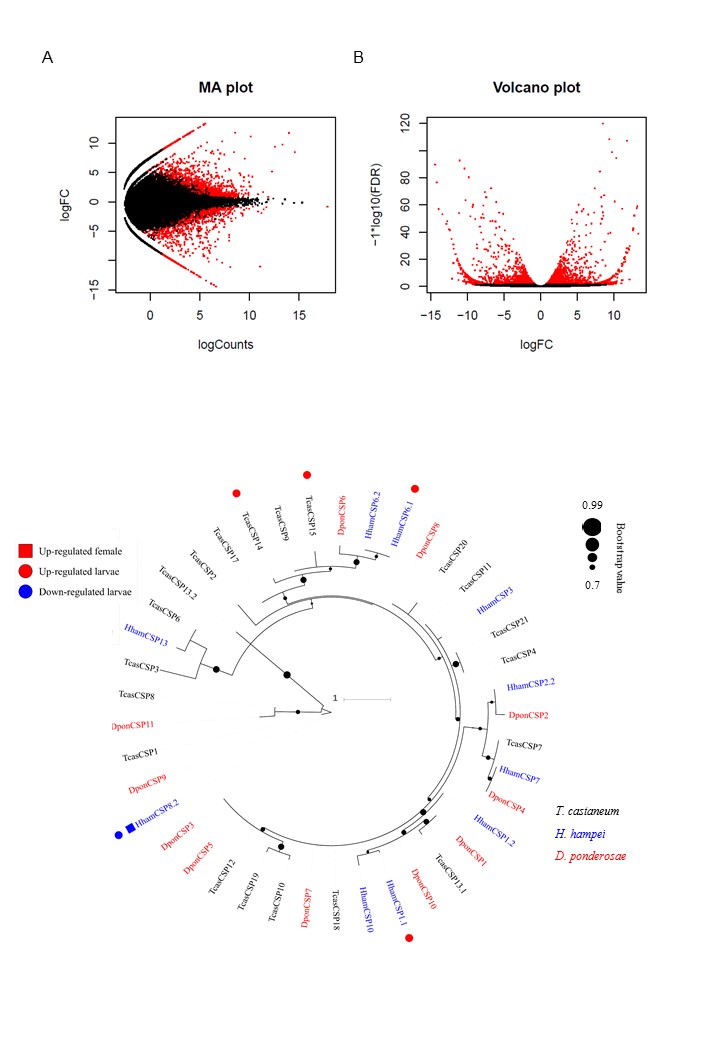


**Supplementary Figure S3.** **Foldchange (FC) values distribution of differential expression analysis.** (**a**) MA plot showing counts distribution among FC value. (**b**) Volcano plot showing statiscal significance (FDR) across FC range in the analysis.

| Functional group | DEG set | Contig | Log2FC | Predicted protein annotation |
| --- | --- | --- | --- | --- |
| Chemosensory receptors | L2 Larvae Down | Hypothenemus_hampei_contig_12430\|c2_g1_i2 | -7.364387221 | chemosensory 8 |
|  |  | Hypothenemus_hampei_contig_154\|c0_g2_i1 | -8.498910824 | odorant binding 1 |
|  |  | Hypothenemus_hampei_contig_18674\|c0_g1_i1 | -7.259677487 | odorant binding 5 |
|  |  | Hypothenemus_hampei_contig_24447\|c0_g2_i1 | -7.584157057 | odorant-binding 6 |
|  |  | Hypothenemus_hampei_contig_28774\|c0_g1_i1 | -8.870528234 | chemosensory 10 |
|  |  | Hypothenemus_hampei_contig_41080\|c0_g1_i1 | -2.081118277 | odorant binding 2 |
|  | L2 Larvae Up | Hypothenemus_hampei_contig_14038\|c0_g3_i1 | 2.475518468 | odorant binding 13 |
|  |  | Hypothenemus_hampei_contig_1665\|c0_g3_i1 | 8.325106899 | odorant binding partial |
|  |  | Hypothenemus_hampei_contig_23152\|c0_g11_i1 | 8.699211567 | chemosensory 2 |
|  |  | Hypothenemus_hampei_contig_24369\|c0_g1_i5 | 3.098464896 | odorant-binding 30 |
|  |  | Hypothenemus_hampei_contig_36517\|c2_g1_i2 | 2.479809422 | chemosensory 1 |
|  |  | Hypothenemus_hampei_contig_41326\|c0_g1_i4 | 2.648608777 | odorant binding |
|  |  | Hypothenemus_hampei_contig_43372\|c0_g1_i1 | 5.230483388 | odorant-binding partial |
|  |  | Hypothenemus_hampei_contig_46690\|c4_g83_i2 | 3.577373325 | chemosensory 6 |
|  |  | Hypothenemus_hampei_contig_5388\|c0_g2_i1 | 9.046621442 | odorant receptor 15 |
|  | Male Down | Hypothenemus_hampei_contig_18674\|c0_g1_i1 | -6.007212075 | odorant binding 5 |
|  |  | Hypothenemus_hampei_contig_24447\|c0_g1_i1 | -5.142375943 | odorant-binding 6 |
|  | Male Up | Hypothenemus_hampei_contig_11391\|c1_g1_i1 | 10.65449291 | odorant receptor 297 |
|  |  | Hypothenemus_hampei_contig_13390\|c0_g1_i1 | 3.689765561 | odorant binding 18 |
|  |  | Hypothenemus_hampei_contig_14038\|c0_g3_i1 | 2.31085715 | odorant binding 13 |
|  |  | Hypothenemus_hampei_contig_15489\|c2_g3_i1 | 5.93059934 | chemosensory 8 |
|  |  | Hypothenemus_hampei_contig_15508\|c0_g1_i15 | 8.751246469 | odorant receptor 5 |
|  |  | Hypothenemus_hampei_contig_1665\|c0_g3_i1 | 5.314689903 | odorant binding partial |
|  |  | Hypothenemus_hampei_contig_1701\|c0_g1_i1 | 6.738745942 | odorant receptor 8 |
|  |  | Hypothenemus_hampei_contig_21708\|c0_g1_i1 | 4.633997268 | odorant binding 13 |
|  |  | Hypothenemus_hampei_contig_41326\|c0_g1_i2 | 2.151226339 | odorant-binding 11 |
|  |  | Hypothenemus_hampei_contig_43372\|c0_g1_i1 | 5.172777189 | odorant-binding partial |
|  |  | Hypothenemus_hampei_contig_44237\|c0_g2_i1 | 8.474440924 | odorant receptor 23 |
|  |  | Hypothenemus_hampei_contig_7961\|c0_g1_i1 | 9.355341059 | chemosensory ionotropic receptor partial |
| Juvenile Hormone-related proteins | L2 Larvae Down | Hypothenemus_hampei_contig_16847\|c0_g1_i1 | -6.858838883 | juvenile hormone esterase |
|  |  | Hypothenemus_hampei_contig_22441\|c1_g2_i1 | -8.836359879 | juvenile hormone esterase |
|  |  | Hypothenemus_hampei_contig_32744\|c0_g1_i1 | -4.330971965 | juvenile hormone inducible partial |
|  |  | Hypothenemus_hampei_contig_33576\|c0_g1_i5 | -6.921776554 | hemolymph juvenile hormone-binding partial |
|  |  | Hypothenemus_hampei_contig_36519\|c1_g2_i1 | -3.156955487 | juvenile hormone epoxide hydrolase 2 |
|  | L2 Larvae Up | Hypothenemus_hampei_contig_28980\|c0_g2_i1 | 3.281054999 | Juvenile hormone epoxide hydrolase 1 |
|  |  | Hypothenemus_hampei_contig_43090\|c0_g1_i1 | 2.605937818 | juvenile hormone binding partial |
|  | Male Down | Hypothenemus_hampei_contig_32744\|c0_g1_i1 | -3.385304319 | juvenile hormone inducible partial |
|  |  | Hypothenemus_hampei_contig_36519\|c1_g2_i7 | -5.190221679 | Juvenile hormone epoxide hydrolase 1 |
|  |  | Hypothenemus_hampei_contig_46456\|c0_g1_i1 | -2.349915233 | juvenile hormone inducible partial |
|  | Male Up | Hypothenemus_hampei_contig_12157\|c2_g2_i1 | 7.881819692 | juvenile hormone binding partial |
|  |  | Hypothenemus_hampei_contig_36519\|c1_g2_i6 | 4.906379336 | Juvenile hormone epoxide hydrolase 1 |
|  |  | Hypothenemus_hampei_contig_43090\|c0_g1_i1 | 3.517623035 | juvenile hormone binding partial |
| Cytochrome P450 | L2 Larvae Down | Hypothenemus_hampei_contig_19523\|c0_g1_i12 | -3.607762784 | cytochrome P450 CYP9Z18 |
|  |  | Hypothenemus_hampei_contig_20776\|c0_g4_i1 | -12.39663459 | cytochrome P450 4C1-like isoform X1 |
|  |  | Hypothenemus_hampei_contig_29164\|c0_g2_i1 | -3.738540115 | cytochrome P450 CYP6DJ1 |
|  |  | Hypothenemus_hampei_contig_41707\|c0_g2_i1 | -9.958282254 | cytochrome P450 CYP6DK1 |
|  |  | Hypothenemus_hampei_contig_42922\|c0_g1_i3 | -2.666546622 | cytochrome P450 |
|  |  | Hypothenemus_hampei_contig_47722\|c0_g2_i1 | -4.281093434 | cytochrome P450 4d2 |
|  |  | Hypothenemus_hampei_contig_9539\|c0_g1_i12 | -11.08721952 | cytochrome P450 |
|  | L2 Larvae Up | Hypothenemus_hampei_contig_16892\|c0_g3_i3 | 3.949868276 | cytochrome P450 CYP410a1 |
|  |  | Hypothenemus_hampei_contig_26843\|c0_g1_i2 | 4.456558812 | cytochrome P450 CYP6DG1v3 |
|  |  | Hypothenemus_hampei_contig_2800\|c0_g1_i2 | 2.129248554 | cytochrome P450 |
|  |  | Hypothenemus_hampei_contig_30541\|c0_g1_i1 | 3.990036997 | cytochrome P450 |
|  |  | Hypothenemus_hampei_contig_46655\|c3_g3_i4 | 9.038733964 | cytochrome P450 CYP9Z18 |
|  | Male Down | Hypothenemus_hampei_contig_20776\|c0_g4_i1 | -4.994447758 | cytochrome P450 4C1-like isoform X1 |
|  |  | Hypothenemus_hampei_contig_9539\|c0_g1_i29 | -11.49454208 | cytochrome P450 |
|  | Male Up | Hypothenemus_hampei_contig_15417\|c0_g1_i6 | 11.84472649 | cytochrome P450 |
|  |  | Hypothenemus_hampei_contig_15515\|c3_g3_i1 | 10.08424781 | cytochrome P450 301B1 |
|  |  | Hypothenemus_hampei_contig_27911\|c0_g2_i1 | 8.254635333 | cytochrome P450 4- partial |
|  |  | Hypothenemus_hampei_contig_38896\|c0_g1_i1 | 8.317881171 | cytochrome P450 |
|  |  | Hypothenemus_hampei_contig_41710\|c0_g1_i2 | 8.883828658 | cytochrome P450 CYP334e1 |
|  |  | Hypothenemus_hampei_contig_46708\|c10_g15_i1 | 2.707082132 | cytochrome P450 CYP4g56 |
| Chitin and cuticle metabolism | L2 Larvae Up | Hypothenemus_hampei_contig_22262\|c1_g3_i1 | 3.705852995 | chitin deacetylase 2 isoform A precursor |
|  |  | Hypothenemus_hampei_contig_25945\|c0_g1_i4 | 9.03947933 | chitinase 10 isoform X1 |
|  |  | Hypothenemus_hampei_contig_2772\|c0_g1_i18 | 11.82909541 | probable chitinase 3 isoform X2 |
|  |  | Hypothenemus_hampei_contig_34530\|c0_g1_i2 | 3.320319165 | acidic mammalian chitinase-like |
|  |  | Hypothenemus_hampei_contig_34894\|c0_g1_i1 | 2.613834113 | chitinase 3 |
|  |  | Hypothenemus_hampei_contig_38387\|c0_g2_i1 | 2.302089878 | chitin deacetylase 1 precursor |
|  |  | Hypothenemus_hampei_contig_38648\|c0_g1_i1 | 9.226841757 | chitinase 3 |
|  |  | Hypothenemus_hampei_contig_41684\|c0_g1_i1 | 6.898576189 | chitinase 10 precursor |
|  |  | Hypothenemus_hampei_contig_41905\|c0_g2_i1 | 3.883482818 | chitin deacetylase 4 precursor |
|  |  | Hypothenemus_hampei_contig_42180\|c8_g21_i9 | 3.647977925 | probable chitinase 2 |
|  |  | Hypothenemus_hampei_contig_42493\|c2_g2_i1 | 2.629436698 | chitinase 2 |
|  |  | Hypothenemus_hampei_contig_10377\|c0_g1_i1 | 8.732506739 | cuticular analogous to peritrophins 3-A1 precursor |
|  |  | Hypothenemus_hampei_contig_10456\|c0_g2_i1 | 3.845468686 | Larval cuticle 5 |
|  |  | Hypothenemus_hampei_contig_1161\|c0_g1_i1 | 6.633876703 | Endocuticle structural glyco bd-1 |
|  |  | Hypothenemus_hampei_contig_17490\|c0_g1_i1 | 5.167043527 | flexible cuticle 12-like |
|  |  | Hypothenemus_hampei_contig_24570\|c0_g1_i1 | 6.862918603 | larval pupal cuticle H1C |
|  |  | Hypothenemus_hampei_contig_25260\|c0_g1_i1 | 4.317273345 | cuticular analogous to peritrophins 1-I precursor |
|  |  | Hypothenemus_hampei_contig_28252\|c0_g1_i1 | 6.36297928 | larval pupal cuticle H1C |
|  |  | Hypothenemus_hampei_contig_28334\|c0_g1_i1 | 4.654366648 | cuticular analogous to peritrophins 3-B precursor |
|  |  | Hypothenemus_hampei_contig_32180\|c0_g1_i1 | 6.623967078 | larvae cuticle |
|  |  | Hypothenemus_hampei_contig_36516\|c0_g3_i1 | 9.703418726 | endocuticle structural glyco bd-3-like |
|  |  | Hypothenemus_hampei_contig_41008\|c0_g4_i1 | 4.914158627 | larval cuticle A2B |
|  |  | Hypothenemus_hampei_contig_43159\|c0_g1_i1 | 2.558831583 | cuticular analogous to peritrophins 1-H isoform X1 |
|  |  | Hypothenemus_hampei_contig_47696\|c0_g2_i1 | 7.159814466 | flexible cuticle 12-like |
|  |  | Hypothenemus_hampei_contig_6260\|c0_g2_i2 | 4.575007002 | cuticular analogous to peritrophins 3-C5 isoform 1 precursor |
|  | Male Up | Hypothenemus_hampei_contig_22262\|c1_g2_i2 | 5.147090168 | chitin deacetylase 2 isoform B precursor |
|  |  | Hypothenemus_hampei_contig_22482\|c0_g1_i2 | 3.716844783 | chitin synthase I |
|  |  | Hypothenemus_hampei_contig_2959\|c0_g1_i1 | 5.637187262 | Chitinase Idgf4 |
|  |  | Hypothenemus_hampei_contig_34894\|c0_g1_i1 | 3.295033366 | chitinase 3 |
|  |  | Hypothenemus_hampei_contig_37458\|c0_g1_i2 | 5.400556347 | chitin deacetylase 5 isoform X5 |
|  |  | Hypothenemus_hampei_contig_39314\|c0_g1_i2 | 2.899599712 | chitinase 3 |
|  |  | Hypothenemus_hampei_contig_42493\|c2_g2_i1 | 2.569405783 | chitinase 2 |
|  |  | Hypothenemus_hampei_contig_10377\|c0_g2_i1 | 2.331728354 | cuticular analogous to peritrophins 3-A1 precursor |
|  |  | Hypothenemus_hampei_contig_10456\|c0_g2_i1 | 2.586900208 | Larval cuticle 5 |
|  |  | Hypothenemus_hampei_contig_10475\|c0_g3_i1 | 8.156682199 | cuticle 7 |
|  |  | Hypothenemus_hampei_contig_12353\|c0_g1_i1 | 8.141146694 | adult-specific cuticular ACP-20 |
|  |  | Hypothenemus_hampei_contig_1308\|c1_g2_i1 | 11.82367254 | Adult-specific rigid cuticular |
|  |  | Hypothenemus_hampei_contig_21349\|c0_g1_i1 | 9.961775767 | cuticle 19 |
|  |  | Hypothenemus_hampei_contig_23394\|c0_g1_i2 | 6.2511725 | Insect cuticle partial |
|  |  | Hypothenemus_hampei_contig_25099\|c0_g1_i1 | 5.684376167 | cuticle precursor |
|  |  | Hypothenemus_hampei_contig_25260\|c0_g1_i1 | 3.488849275 | cuticular analogous to peritrophins 1-I precursor |
|  |  | Hypothenemus_hampei_contig_28334\|c0_g1_i1 | 3.23955422 | cuticular analogous to peritrophins 3-B precursor |
|  |  | Hypothenemus_hampei_contig_28839\|c0_g5_i1 | 8.99950278 | Insect cuticle partial |
|  |  | Hypothenemus_hampei_contig_40497\|c2_g2_i1 | 2.748881597 | cuticular analogous to peritrophins 1-J precursor |
|  |  | Hypothenemus_hampei_contig_41008\|c0_g2_i1 | 9.002801673 | larval cuticle A2B |
|  |  | Hypothenemus_hampei_contig_41551\|c0_g3_i1 | 3.673346651 | cuticular precursor |
|  |  | Hypothenemus_hampei_contig_43159\|c0_g1_i1 | 2.474745336 | cuticular analogous to peritrophins 1-H isoform X1 |
|  |  | Hypothenemus_hampei_contig_46076\|c0_g1_i1 | 4.062737975 | Insect cuticle partial |
|  |  | Hypothenemus_hampei_contig_5351\|c0_g1_i4 | 9.128104103 | cuticle CP7 |
|  |  | Hypothenemus_hampei_contig_6260\|c0_g2_i2 | 2.602113098 | cuticular analogous to peritrophins 3-C5 isoform 1 precursor |

**Supplementary Table S1**. Physiological and metabolic processes enriched in differential expression analysis. Contigs with their respective foldchanges are given for each differentially expressed genes set.

| Gene | Primer Sequence Forward (5'-3') | Primer Sequence Reverse (5'-3') | Amplicon (bp) | Efficiency (%) | Correlation efficiency |
| --- | --- | --- | --- | --- | --- |
| Act2 | F: GGGAAATTGTTCGTGACATCA | R: GGGCAACGGAATCTTTCG | 157 | 90.79 | 0.9964 |
| ChiX | F: TTGGATAGAACAAGGAGCTGAC | R: CTCTTGCACGGGTATCTTCTC | 149 | 105.98 | 0.9907 |
| CPAP3A1 | F: AATATGCCCATCCCACAGAC | R: GTCTTCACATCCAGCAACATTC | 146 | 107.52 | 0.9949 |
| CPAP3C5 | F: GGAGGTCAGTAACACAGGTTC | R: ACCAGTACCATCTGCATCAC | 153 | 101.15 | 0.9713 |
| HexI | F: AAGTCAAGCGGTGGAGAAAG | R: TGTTCATCCTGGCCTGTTG | 145 | 109.53 | 0.9988 |
| JHEHII | F: AAATTCCTGCGACCGTACC | R: AGATCAGGTACTTCCAAAGCAG | 154 | 105.64 | 0.9856 |
| JHE | F: ACCCGAGGAAACTAGAAAGC | R: GGTCCTCCAGGTTAATTTGTTC | 158 | 99.27 | 0.9881 |

**Supplementary Table S2**. Primers used for RT-qPCR validation with their respective PCR amplification efficiencies (E) and correlation coefficients (R2).
