# Supplementary material for: Transcriptome and gene expression analysis of three developmental stages of the coffee berry borer, Hypothenemus hampei
Source: Sci Rep. 2019 Sep 5;9:12804. doi: 10.1038/s41598-019-49178-x (PMC6728347; doi:10.1038/s41598-019-49178-x)
Supplement: Supplementary file 2 — Supplementary Dataset 1 [file 41598_2019_49178_MOESM2_ESM.docx]

>Hypothenemus_hampei_contig_10064|c0_g1_i1 hypothetical protein Eint_061570

gtaaatatttgtcgggtgttgtttgtttcgagtaaaaatggagcggcccccacccctggaagtgaagggagagatggaca

ctggggacggagcggaggactctgacggccaccggctgtgcgaggctgcagatgcactgggcagggttgtggtggcaacc

gggccgatgagcaagagagatgcgagctgtgtgctgtgcgacggcgatgcaagtgtgtcgacggtcgacgacgttgcgcg

cgtgtactcgtgcctccttgcatgtgtgtctgagcatgtgtctgtggtttctgccgtgagcttcagggacctgtttattg

tcgagagttttctgcttgaccaggtgtgcgaggagaagacgcttgactttgacaggattttcgagaggaaggggggctct

ggagagcgacgcgagagggtgctgagggcggtgagggagtggtttccaaacaactgcataaacatagactgcgagcagga

gcccgagtcgctgatgtacaaggtgtacgagcgaagcgatgcagcggcaaatgcagagccgcgggacgtggagcttctac

ttgtgattctggcacgcaggtgcgagtcgtcgaggttcttcaaggtgcttggcaggcacaagaggaccaaggggatgttc

cggttggggcttctgatgcgcctccgggacggggacgatgcgggcgtcgagaggatgctgcggagctgcgacgagggcag

tctttgtgccgagagcagctttgtggatggtgaggaggccgagcggagtgctgcggcc

>Hypothenemus_hampei_contig_10065|c0_g1_i1 SUBTILISIN-LIKE SERINE PROTEASE PRECURSOR

tggacaggacctccatgagggcattatacagcatgaatgtacttgaggcgcagaacatcctgcagcctgacgagagcatt

ggggcaaagatggtcaatgggtttgtggcacgtatgagcgactccacggccgagaagatgagggaacatccaaacgtgaa

gatggtggtcaaagacagccctgtgagcatcagcaggttcaaggctctcgacattcccaaggacgattcaggagacacga

ttataatccaaaggaacgccccctggggactagctcgtgtgggggggagtgcgtcgctggtacatgggaagtactactat

cctgtgaactctgggaagggtgtcaacgtctatgtgttggacacaggcgtggaagtgacccatccggagtttgagggaag

ggctaggtggggagccaactttgtctcgaaaagccccgatagggacgagcatggacatggaacgcattgtgcaggaatca

ttggggggaagaacttcgggataaccaagaagtccaatatcattgctgtcaaggttcttgacaagtacggatctgggatg

atctcccggcttctccaaggagtggactttgtcatccgagagcacgagaggaggaaggacgagttgtacagcgcagcagc

agacaagtaccttagcatggagaggtcctctagctcgattgaaatagacgtgagcgactcagagagcttttcattcctta

gaagtgagggcaaagaggaggctttgtcgatgcagcgcctggcagatgtgatttccagtaaggccctgcagccaaagacc

gttgtgaatctcagcgtcggaggattcaggaacgcagccttgaactttgcaatcgaatatgcatcgaagctaggaatcca

tttctcgacagctgcaggcaacgagcatgaaaacgcatgtgacttttcgcccggctcatcccgtgcaagcataacaacgg

gagcctcgacatatcgggacacagttgcgttcttcagcaactttggaaggtgcgtcaacatatttgccccgggagtggac

atactcagctcgtggatagggggggcacaaaagattgcatccggaacgtccatggctgctccacataccactggagtaat

ggctgcataccttacatactacgactacgacccccatatcctcaagagccacatcatagatgatgcccataatgtggtag

aggaggatacgtccgatggccacgggaagttggcctctccatggcccttgccgttgttcagcagcagcagaaagaggctt

ccgatgctatctatggagagccttctgaagagcctgcagaataaatcgcggtgaaatgctccagatcgg

>Hypothenemus_hampei_contig_10067|c0_g1_i1 Hsp90 ATPase activator

gatcaggcaggcgcttgagtcccgaggatatacagtgcatgagatggatgtgatgacgaaggtgtgccaaaggatgaata

cgttggggcttgtgtacatgataagctttgagtgtgtcaaggacgggaagcactgcggggtaaggggcttctactctgtg

tctgagaaggccaacggaatcgaggacttcgagtggtttccagggttcttcaaggagatggaggccgaggcagtgctcaa

gttcggagacagggttctcgacgtggacaggcccgttgagaggaagccttgtgtaaagaacatagccgctgaggatgcta

aggtcgtggacgtcacctacagggcaagcatcaactgcggtgtggatgagctcaagagctttattctgtctcccgagttt

atttccatgtggagcgggcacagggcgttgttcgagagtggtagagtcctgatcgacggggttgaaatgcatgatctgag

ggaggattctgggtgcatcaagatgggatggaagcgtgcggagtgggccggggtgtcggacgtcaggatggagcttgaga

gctttctaagcagcacaaaggtaaaggtggtccagaagggtgtcccgatcaaggaggcgggaagtgtccgggcgtggtgg

catgagagggtatttgctccgatttccggatcctttgggtttatgctgaagcccggggagtaggcatgtgggcatggaga

gatc

>Hypothenemus_hampei_contig_10089|c0_g1_i1 ubiquitin L40 ribosomal fusion

gatctgtctgaggtttggcggaaagacttcatttgtccaggtcgatcctctggagtcgattctgtccgtaaggagtcgtt

ccagcggctcttgtggtgcaagcgcctctgcaattgcgcttctgcacaactccaggatgctggaggactcaatgacagtg

gctggttgcggcataagaagcctcgacacaatcgcggtgtgtccccggcttcttgggggaggtggaaacatgtccgaaaa

cgacagggccatggccatgaaggagaagaacgactgcctgatctgccgcagatgctatgcaaggtccggcaaggctgcgc

agaagtgcaggaagtgctgcagcacagaccttaggccgaagaagcccctcaaggcaatgaagaagaaataagaagtttaa

atattaaacatttactt

>Hypothenemus_hampei_contig_10096|c0_g1_i1 TRANSCRIPTIONAL REGULATOR-LIKE PROTEIN

gagcgggataaggctttgcgtggatgatttggaggtgatgggggacgtaatgagtctttgcgaggtctatgtggagtgtg

ggcatggagagagcaggagaggcgcagtgcctggggcttggcctgttgtgaagtctgtttttgagaagcttgggatggac

gagtttgtgatggtgtccgactttcctttgctctgcatcttcaggttcattgccttggtgtatgagaggctagtggaggt

caagaagatgtaccccgtggagtcttctgcagacagcccgtctgagagcaggccgaagtacatcgaggtgagggacacgt

gccttgagttcatgaggaagaatatcgacggatacacctttgaggatagaatcagagagctgaccgagtgcagggggttc

aagctctacaacatgaagaagatggtgtcgaagatagagaagcaggccgtgtctctgatggagagccctctgtcgcttga

gtctctccgcgggacgtgtgagtttccggccggcgaggatctgtattgcttccgaaagacaggggccgtcattgaggtta

aggcagttgggcccaggaaggacgaggagtgggatgagtatgtggagaagtttgagtcgttatctccttgtgaggaggtg

ggggctgtggcaccgtttctcccacgaacctgcaggaagcctccgataacggggagcctgaggcaggtcctgaaggttca

gctgagccctgcaacatacaagctcaggtatgtgactgggtctgagttcctttacatgaatctgtggtcttgcccgaaga

gaagatagccttgtggcaagga

>Hypothenemus_hampei_contig_10117|c0_g1_i1 muskelin N-terminus domain-containing

aagtcgaggacggaggccggcgtggacatcatgcctgcgccgcggtctgcgcaccagtttgtcgagatgaacgggtggtt

ttatctgttcggaggcaacgtttctgagaggatggagcgacgtgtgaacgacatgtgggtgttcaagctcgcaaagaaga

caaagcaggagatatgccacctgtcgaagttcctggtcagaaagcacaagtacctccaccttcttggggctgacgaggag

agggcaattgattatctgcggacttctgtgctaagcatggttgtcgacgacgacacaagagagctcttcgaagagctgtg

tcacgaggtgtatagaaggaggatctttgtcgatcctgtcgaggacatttgtagattattggtcac

>Hypothenemus_hampei_contig_10119|c0_g1_i1 ATP ADP translocase

cgggcctattgttgccattgtctcgctagttctgttctttccaatggtgtttctcaacaacatcgctgagggagacctga

ttgctccctcgccggggagcattggaaggtttgttcttgaaaactacacgggaatgttcatgacgtctatcatcaggata

tcgaagtactgcttcttcgacattgcaaaggaggccgcctgcataagaataagccctgtccacagacaggttttcagagg

aatacatgacgggctgggaataaacatcggaaagacaatagggtccgtgtattgcacggccgtgacggttctgtttgacg

tcagggatgtccggaacaccgtcacgatatcgactgtgttcgtggggatgttctgcatcgcatggattaagtggatcttt

tatataaataagaagtacaaggcggcgatcgagagaaacggcttcatcgacgtagagatggctgaggaagg

>Hypothenemus_hampei_contig_10122|c0_g1_i1 Alpha-L-rhamnosidase N-terminal domain [ [

tggcagtgccttactccggtgaaccgctggatacagatactacctatttctggcgggtgaaaagcttggtgtccggcaat

gaatccagctggtcacaaccacaaaaatttattaccgcagcttccgatgccgattggaaagccaaagcaatctgggtaga

gggttctaccggcaactccgcggccgtagatccttataattggatgctgacccgaaaagggttttcagctagcaccagct

tacttgttagcgcctggattcgcattactgcacagtctccggagactgcgcgtcagtacgtttacaagctatggttaaat

aacaatctcattggactgggaccagtgcgtgcccagaataccaacaatgaggaagctcgttatcatatttatgatatcag

cgaccagttgcaggaaggggacaatgccgtcgcagccctggcttatgctaataacaaccatgctttcatgctggatttgg

ttgttcagaatactgacggcacaaaaagcatcttgtgcagcagcggagccaactggaaggcgcgttccgctggagcatgg

ttgccagagatc

>Hypothenemus_hampei_contig_1023|c0_g1_i1 hypothetical protein

tggttgtctacacaagcagcgacgactttgagtcgtccaggcttccctgtgccggattgctcgacctttcgagcaggacg

cttcgggtgaagggagataacgatgcagaatatatgtggggggaggcagatgggaatggggacactaggcacggtgctgc

agaggctgtgggggaggcagccaggaatagaagagcagccattgtgtcgcaaagcactcctcgagagctgccgcatgtgt

ttgccaggtacaggatcaagctccgggaagttgccgacgagtgctgtggctctgcaattgttgtgtttggtggaaggctt

tccaagtgggacgcagcaagcatagcgcggaaagccggcgttgcaaaggggattatggggtacttgtccagatgcctgtc

gtacatgggggcaggcgagaagccttgggcagcagagggccttggaaacagccttaggaaggaggaggcaacgagggttc

ttgagaagacccttgacgcaggagaggtagatgatcgtgttctctcaaggtatggtgtcaaggacattctctggctactg

acgcatctcgtatgcagcgacatatacttgttcttcgagatcaaggacatagagatgctccatagggcattggctgtgga

caagacatacttcctgggggcatctgccccaactgcgagcaattcgtcgcttgccttgtctctcttcgaggtcatgctta

ccgcaagagacaaggtctcgccggcgccgatggtggagatgctcttgggcatgcttgcagcatttgacgtcggcagggct

gaggtggcagaggccgtggtcaggatccattggtga

>Hypothenemus_hampei_contig_10233|c0_g1_i1 60S ribosomal L10

tgccatcaccacatttccagcactcaaaatggcccgccgtcccgcacgttgctaccgctactgcaagaacaagccctacc

ctaagtcccggttcaaccgtggtgttcccgaccctaagattcgcatcttcgatcttggtcgcaagaaggcttccgtcgat

gacttccccacctgcgttcacctcgtctccaacgagtacgagcagctgtcctccgaggctcttgaggccgcccgtatctg

tgccaacaagtacctcgtgaagatcgccggtaaggaaggtttccacctgcgtgtccgtgtccaccccttccacgtcgtcc

gtatcaacaagatgttgtcgtgcgccggtgccgatcgtctccagaccggtatgcgtggtgccttcggtaagcccaacggt

ctcgttgcccgtgtgaacatcggccagatcatcctgtccgtccgcacccgtgactcaaaccgtgccgccgccatcgaggc

tctccgccgctcgatgtacaagttccccggtcgccaaaagatcgtcgtctccaagaactggggtttcactcccgtccgcc

gcgaggag

>Hypothenemus_hampei_contig_10233|c0_g2_i1 60S ribosomal L10-A

ctcgaggccgctcgtatctgcgccaacaagtacctcgtcaagcacagtggcaaggagggtttccacctgcgcgtccgcgc

ccaccccttccacgtcgtccgtatcaacaagatgttgtcgtgcgccggtgccgatcgtctccagaccggtatgcgtggtg

ccttcggtaagcccaacggtctcgttgcccgtgtgaacatcggccagatcatcctgtccgtccgcacccgtgactcaaac

cgtgccgccgccatcgaggctctccgccgctcgatgtacaagttccccggtcgccaaaagatcgtcgtctccaagaactg

gggtttcactcccgtccgccgcgaggag

>Hypothenemus_hampei_contig_10233|c0_g3_i1 60S ribosomal L10

ccagttcgcaggaagtgcagccaggtcatttccagcagtcaagatggcccgtcgtcccgcgagatgttaccgctactgca

agaacaagccttaccctaagtcccggttcaaccgtggtgttcccgaccccaagatccgtatcttcgatctgggacgtaag

aaggccaacgtcgatgacttccctctgtgtgtgcacttggtctccaacgagtacgagcagctgtcctccgaggctctcga

agccgcccgtatctgtgccaacaagtacctcgtgaagatcaccggtaaggaaggtttccacctccgtgtccgtgtgcacc

ccttccacgtcgtccgtatcaacaagatgttgtcgtgcgccggtgccgatcgtctccagaccggtatgcgtggtgccttc

ggtaagcccaacggtctcgttgcccgtgtgaacatcggccagatcatcctgtccgtccgcacccgtgactcaaaccgtgc

cgccgccatcgaggctctccgccgctcgatgtacaagttccccggtcgccaaaagatcgtcgtctccaagaactggggtt

tcactcccgtccgccgcgaggag

>Hypothenemus_hampei_contig_1025|c0_g1_i1 DNA mismatch repair

ggccaggaaaaaggttgagatgctaaatgatctctacaaggccaacgggaagcttgtctcttcgtcggatgcaggttatt

ttgcagtgttttcgactgcacaattcagcctaagaaagggagtgttcgtgttgtttgtaaatggaagactggttacaagc

caggagatgaaggagggcctgttcaaggtatataaagatgtgcttcctgcgcagaggtaccctctcatatatatagagct

caacttagagaagagtatggtcgacgtcaatgttcatccgagcaaaagggagtttctttttgctgaagaagaactgatga

caagaaggttgtc

>Hypothenemus_hampei_contig_10251|c0_g1_i1 tetratricopeptide repeat family

ggcaagtatcgtgaagctcacagtaaatatcatgaagcatatgataaatctaatgttagtagacagcatcataaatatag

cactaacagagacaaagccaaagttgagctggatgcattaaatgcgtatcaacttggcgagatattgtttaataaaagaa

gatacaatgatgctttaaaggaatatcaggctgcccatagtagttctcaggtgataaatgcaaaatcaatttataatagt

ggaatatctaaagtacaagtagaaataattgctgagcagttatatgaacaaggtgaggtgttatattgtaccggaaagta

tgtggaagctaagacaaaatataatgaagcagtacatacatctaaagttcacaaggattactatagtagtttaggtgttg

taaaagctcaaacagagttagatgcaattgcattgaatgatcgtgctgacgttctcttaagtcagggtaagtttgctgag

gctataaaaaagtatcaagcggcatatgataaaacacaagtatttaatcaacgtgaacaatataaaaataatagagattc

aatacaggcaaaagtatataatcaagagggttataaattattaagtgaagctacagctgaacgagactttgctcttaaaa

ttctcaaatataaaaatgcacaagaaaaatttgaaaaggctataaataaacgtcctagtagtcatgtgtttagaaatagt

ctgactaaaactttagcagctatgaaggtgattgagacatataattctgtattcaaggactttagccttatgttaacttc

agatatcacggatagtgagttagaagaactaggccatagattagatagtttacttgatatagcggagtatggaaatgact

ctactattgtaaatagatttcatatagtatccttaagaatatccaggaggaaactgaatagtcaattagaatcaattaat

cgaaaagatataaacgacctcatagaggcaattatgaacctaatagaaaccgaaattgaggtattaaagcagctaaatat

tattgaccataagttggaacaagaattaaaatcattagaaggacaattaaatgctctagatctaaataatgaatgtatgc

aaagcaataatgacaacatattagtaaatataacatcaattgcatctcgtgttgaggaatactatgaagaagtagagaac

agggcattggagagattagcacaaaatgcacagcaagagcattgtggaaatactctataactacaagacccattaagtgc

ttctatagttgaaacatacataatggatggaatgaatatgattttacaattaagaattgtatttagagaagcaggaaata

caaaacaaatcacataattatcttttaatatatgcctatatcgaaattaacaatatgaacttacatgatctgttaaaccg

tctggcacaagaaaccataaagaaaaccctaattccatttcttgttacagagaaacgccaatataggaggcaacgacagt

cattaagtatatctaattgatgaacgtagcttggatggaattgtagtaagttatttaaatttagaaaacctaagtgtgga

tggattagaaattccagtagaacagaaactgtatgattgttgcggctctgaactaaatgaaaatatatacaacctacata

taacaggatctaaggaatctactatttaccttcattcctcattacaagagcagagttgaatattaactgatattgagatc

catcaaaagaagaatacagattttgcttatggtatagaatggaacacagactatgaaaatgtagttacctcaacttgtga

atgtacaaaactcagtagttttattaaatacgatatggagaactgataacatactaaacagtaaatcaataatttattga

aaaataaagtattatggccaaataaaaatcagaacaaggaactcaaattaatcaagtaatgatggctgatgataaaattc

aattgtcaaatgaacttaattctttactcgaagctttggtaaattacaaatttatttttgatgctagagtatccgataag

attcctagctctttaccaagaactattactgtaaaactaaataaagcgagaacattattatgtttagactataataataa

tgaatttgaaattgagaataatgacaaaataataaaaaagacagactatgataacgttagaaacaccctgaaagataaaa

gaactcttattcacgaaaataaatggaatgaatcttttaagctggttaagcaacaaattttgtcttatttaattaaacaa

aatgaattagaggaccgagagaattttttaaaagaatttcaacaatttatcgataaattaccggaagtaccgcatgaatt

tattgattgtactcgtaaacttaaggaaattaattttaatgattgggatattgaggaaagtgaagcaaatgatccacgcc

tgcaaactttagaagaaatacataaaaaaatgttagaatctttcgacttatataaggctataggcgaaaactatattaaa

ctaagaaatcaagccgtaattgatccaaacgtaaatactccaaaagaggagaaaaatcctgaagctgctcctgtacagaa

cacagatatcaatatgttcaatgtggtaaaaatccctagagatagtaattgtttttatactgctgttgctgatcagctta

gaagacttaaaataaaaaacaaaaaccctactaatttactagacacttactatacctatgaagaccttagagcattagct

atagattatataaagaaaaatagaaaacaagagtttggtaaagatattgaagaaagattgcaaactcaaaatacagaacc

attacatggtgatttaccatcaaatatacataaaattgatcaatacatcgagcttcacagtcaagaaggagtatgggctg

attccggaatgattggggcattatcatatgcattagatattaccataaa

>Hypothenemus_hampei_contig_10251|c0_g1_i10 tetratricopeptide repeat family

ggcaagtatcgtgaagctcacagtaaatatcatgaagcatatgataaatctaatgttagtagacagcatcataaatatag

cactaacagagacaaagccaaagttgagctggatgcattaaatgcgtatcaacttggcgagatattgtttaataaaagaa

gatacaatgatgctttaaaggaatatcaggctgcccatagtagttctcaggtgataaatgcaaaatcaatttataatagt

ggaatatctaaagtacaagtagaaataattgctgagcagttatatgaacaaggtgaggtgttatattgtaccggaaagta

tgtggaagctaagacaaaatataatgaagcagtacatacatctaaagttcacaaggattactatagtagtttaggtgttg

taaaagctcaaacagagttagatgctgtagagttaaacgagcaagggaatatgctatttgtgagtggtaattttagtgaa

gcaagagacaaataccaacaggcttatgacatatctgaagttgctaaaaaaagtgatggttttttcaggaaattattata

tagtccccatgaatacaaaagtaatatcgataaagctcaaacagagttaaatgcaattgcattgaatgatcgtgctgacg

ttctcttaagtcagggtaagtttgctgaggctataaaaaagtatcaagcggcatatgataaaacacaagtatttaatcaa

cgtgaacaatataaaaataatagagattcaatacaggcaaaagtatataatcaagagggttataaattattaagtgaagc

tacagctgaacgagactttgctcttaaaattctcaaatataaaaatgcacaagaaaaatttgaaaaggctataaataaac

gtcctagtagtcatgtgtttagaaatagtctgactaaaactttagcagctatgaaggtgattgagacatataattctgta

ttcaaggactttagccttatgttaacttcagatatcacggatagtgagttagaagaactaggccatagattagatagttt

acttgatatagcggagtatggaaatgactctactattgtaaatagatttcatatagtatccttaagaatatccaggagga

aactgaatagtcaattagaatcaattaatcgaaaagatataaacgacctcatagaggcaattatgaacctaatagaaacc

gaaattgaggtattaaagcagctaaatattattgaccataagttggaacaagaattaaaatcattagaaggacaattaaa

tgctctagatctaaataatgaatgtatgcaaagcaataatgacaacatattagtaaatataacatcaattgcatctcgtg

ttgaggaatactatgaagaagtagagaacagggcattggagagattagcacaaaatgcacagcaagagcattgtggaaat

actctataactacaagacccattaagtgcttctatagttgaaacatacataatggatggaatgaatatgattttacaatt

aagaattgtatttagagaagcaggaaatacaaaacaaatcacataattatcttttaatatatgcctatatcgaaattaac

aatatgaacttacatgatctgttaaaccgtctggcacaagaaaccataaagaaaaccctaattccatttcttgttacaga

gaaacgccaatataggaggcaacgacagtcattaagtatatctaattgatgaacgtagcttggatggaattgtagtaagt

tatttaaatttagaaaacctaagtgtggatggattaggtgaatatattttatatgttttcaaagttacgtatcccaaata

taaattctcttttaaagaaattccagtagaacagaaactgtatgattgttgcggctctgaactaaatgaaaatatataca

acctacatataacaggatctaaggaatctactatttaccttcattcctcattacaagagcagagttgaatattaactgat

attgagatccatcaaaagaagaatacagattttgcttatggtatagaatggaacacagactatgaaaatgtagttacctc

aacttgtgaatgtacaaaactcagtagttttattaaatacgatatggagaactgataacatactaaacaagtatatttta

ggtaaatcaataatttattgaaaaataaagtattatggccaaataaaaatcagaacaaggaactcaaattaatcaagtaa

tgatggctgatgataaaattcaattgtcaaatgaacttaattctttactcgaagctttggtaaattacaaatttattttt

gatgctagagtatccgataagattcctagctctttaccaagaactattactgtaaaactaaataaagcgagaacattatt

atgtttagactataataataatgaatttgaaattgagaataatgacaaaataataaaaaagacagactatgataacgtta

gaaacaccctgaaagataaaagaactcttattcacgaaaata

>Hypothenemus_hampei_contig_10251|c0_g1_i2 tetratricopeptide repeat family

ggcaagtatcgtgaagctcacagtaaatatcatgaagcatatgataaatctaatgttagtagacagcatcataaatatag

cactaacagagacaaagccaaagttgagctggatgcattaaatgcgtatcaacttggcgagatattgtttaataaaagaa

gatacaatgatgctttaaaggaatatcaggctgcccatagtagttctcaggtgataaatgcaaaatcaatttataatagt

ggaatatctaaagtacaagtagaaataattgctgagcagttatatgaacaaggtgaggtgttatattgtaccggaaagta

tgtggaagctaagacaaaatataatgaagcagtacatacatctaaagttcacaaggattactatagtagtttaggtgttg

taaaagctcaaacagagttagatgcaattgcattgaatgatcgtgctgacgttctcttaagtcagggtaagtttgctgag

gctataaaaaagtatcaagcggcatatgataaaacacaagtatttaatcaacgtgaacaatataaaaataatagagattc

aatacaggcaaaagtatataatcaagagggttataaattattaagtgaagctacagctgaacgagactttgctcttaaaa

ttctcaaatataaaaatgcacaagaaaaatttgaaaaggctataaataaacgtcctagtagtcatgtgtttagaaatagt

ctgactaaaactttagcagctatgaaggtgattgagacatataattctgtattcaaggactttagccttatgttaacttc

agatatcacggatagtgagttagaagaactaggccatagattagatagtttacttgatatagcggagtatggaaatgact

ctactattgtaaatagatttcatatagtatccttaagaatatccaggaggaaactgaatagtcaattagaatcaattaat

cgaaaagatataaacgacctcatagaggcaattatgaacctaatagaaaccgaaattgaggtattaaagcagctaaatat

tattgaccataagttggaacaagaattaaaatcattagaaggacaattaaatgctctagatctaaataatgaatgtatgc

aaagcaataatgacaacatattagtaaatataacatcaattgcatctcgtgttgaggaatactatgaagaagtagagaac

agggcattggagagattagcacaaaatgcacagcaagagcattgtggaaatactctataactacaagacccattaagtgc

ttctatagttgaaacatacataatggatggaatgaatatgattttacaattaagaattgtatttagagaagcaggaaata

caaaacaaatcacataattatcttttaatatatgcctatatcgaaattaacaatatgaacttacatgatctgttaaaccg

tctggcacaagaaaccataaagaaaaccctaattccatttcttgttacagagaaacgccaatataggaggcaacgacagt

cattaagtatatctaattgatgaacgtagcttggatggaattgtagtaagttatttaaatttagaaaacctaagtgtgga

tggattaggtgaatatattttatatgttttcaaagttacgtatcccaaatataaattctcttttaaagaaattccagtag

aacagaaactgtatgattgttgcggctctgaactaaatgaaaatatatacaacctacatataacaggatctaaggaatct

actatttaccttcattcctcattacaagagcagagttgaatattaactgatattgagatccatcaaaagaagaatacaga

ttttgcttatggtatagaatggaacacagactatgaaaatgtagttacctcaacttgtgaatgtacaaaactcagtagtt

ttattaaatacgatatggagaactgataacatactaaacagtaaatcaataatttattgaaaaataaagtattatggcca

aataaaaatcagaacaaggaactcaaattaatcaagtaatgatggctgatgataaaattcaattgtcaaatgaacttaat

tctttactcgaagctttggtaaattacaaatttatttttgatgctagagtatccgataagattcctagctctttaccaag

aactattactgtaaaactaaataaagcgagaacattattatgtttagactataataataatgaatttgaaattgagaata

atgacaaaataataaaaaagacagactatgataacgttagaaacaccctgaaagataaaagaactcttattcacgaaaat

aaatggaatgaatcttttaagctggttaagcaacaaattttgtcttatttaattaaacaaaatgaattagaggaccgaga

gaattttttaaaagaatttcaacaatttatcgataaattaccggaagtaccgcatgaatttattgattgtactcgtaaac

ttaaggaaattaattttaatgattgggatattgaggaaagtgaagcaaatgatccacgcctgcaaactttagaagaaata

cataaaaaaatgttagaatctttcgacttatataaggctataggcgaaaactatattaaactaagaaatcaagccgtaat

tgatccaaatgtaaatactccagaagaggagaaaaatcctgaagctgctcctgtacagaacacagatatcaatatgttca

atgtggtaaaaatccctagagatagtaattgtttttatactgctgttgctgatcagcttagaagacttaaaataaaaaac

aaaaaccctactaatttactagacacttactatacctatgaagaccttagagcattagctatagattatataaagaaaaa

tagaaaacaagagtttggtaaagatattgaagaaagattgcaaactcaaaatacagaaccattacatggtgatttaccat

caaatatacataaaattgatcaatacatcgagcttcacagtcaagaaggagtatgggctgattccggaatgattggggca

ttatcatatgcattagatattaccataaa

>Hypothenemus_hampei_contig_10251|c0_g1_i3 tetratricopeptide repeat family

ggcaagtatcgtgaagctcacagtaaatatcatgaagcatatgataaatctaatgttagtagacagcatcataaatatag

cactaacagagacaaagccaaagttgagctggatgcattaaatgcgtatcaacttggcgagatattgtttaataaaagaa

gatacaatgatgctttaaaggaatatcaggctgcccatagtagttctcaggtgataaatgcaaaatcaatttataatagt

ggaatatctaaagtacaagtagaaataattgctgagcagttatatgaacaaggtgaggtgttatattgtaccggaaagta

tgtggaagctaagacaaaatataatgaagcagtacatacatctaaagttcacaaggattactatagtagtttaggtgttg

taaaagctcaaacagagttagatgcaattgcattgaatgatcgtgctgacgttctcttaagtcagggtaagtttgctgag

gctataaaaaagtatcaagcggcatatgataaaacacaagtatttaatcaacgtgaacaatataaaaataatagagattc

aatacaggcaaaagtatataatcaagagggttataaattattaagtgaagctacagctgaacgagactttgctcttaaaa

ttctcagatataaaaacgcgcaagaaaaatttgaaaaggctataaataaacgtcctagttatcatgtgtatagaaatagt

ctgactaaagctttagcggctataaagatgattgagatatataattctgcattcaaggactttagccttatgttaacttc

agatatcacggatagtgagttagaagaactaggccatagattagatagtttacttgatatagcggagtatggaaatgact

ctactattgtaaatagatttcatatagtatccttaagaatatccaggaggaaactgaatagtcaattagaatcaattaat

cgaaaagatataaacgacctcatagaggcaattatgaacctaatagaaaccgaaattgaggtattaaagcagctaaatat

tattgaccataagttggaacaagaattaaaatcattagaaggacaattaaatgctctagatctaaataatgaatgtatgc

aaagcaataatgacaacatattagtaaatataacatcaattgcatctcgtgttgaggaatactatgaagaagtagagaac

agggcattggagagattagcacaaaatgcacagcaagagcattgtggaaatactctataactacaagacccattaagtgc

ttctatagttgaaacatacataatggatggaatgaatatgattttacaattaagaattgtatttagagaagcaggaaata

caaaacaaatcacataattatcttttaatatatgcctatatcgaaattaacaatatgaacttacatgatctgttaaaccg

tctggcacaagaaaccataaagaaaaccctaattccatttcttgttacagagaaacgccaatataggaggcaacgacagt

cattaagtatatctaattgatgaacgtagcttggatggaattgtagtaagttatttaaatttagaaaacctaagtgtgga

tggattagaaattccagtagaacagaaactgtatgattgttgcggctctgaactaaatgaaaatatatacaacctacata

taacaggatctaaggaatctactatttaccttcattcctcattacaagagcagagttgaatattaactgatattgagatc

catcaaaagaagaatacagattttgcttatggtatagaatggaacacagactatgaaaatgtagttacctcaacttgtga

atgtacaaaactcagtagttttattaaatacgatatggagaactgataacatactaaacaagtatattttaggtaaatca

ataatttattgaaaaataaagtattatggccaaataaaaatcagaacaaggaactcaaattaatcaagtaatgatggctg

atgataaaattcaattgtcaaatgaacttaattctttactcgaagctttggtaaattacaaatttatttttgatgctaga

gtatccgataagattcctagctctttaccaagaactattactgtaaaactaaataaagcgagaacattattatgtttaga

ctataataataatgaatttgaaattgagaataatgacaaaataataaaaaagacagactatgataacgttagaaacaccc

tgaaagataaaagaactcttattcacgaaaata

>Hypothenemus_hampei_contig_10251|c0_g1_i4 tetratricopeptide repeat family

ggcaagtatcgtgaagctcacagtaaatatcatgaagcatatgataaatctaatgttagtagacagcatcataaatatag

cactaacagagacaaagccaaagttgagctggatgcattaaatgcgtatcaacttggcgagatattgtttaataaaagaa

gatacaatgatgctttaaaggaatatcaggctgcccatagtagttctcaggtgataaatgcaaaatcaatttataatagt

ggaatatctaaagtacaagtagaaataattgctgagcagttatatgaacaaggtgaggtgttatattgtaccggaaagta

tgtggaagctaagacaaaatataatgaagcagtacatacatctaaagttcacaaggattactatagtagtttaggtgttg

taaaagctcaaacagagttagatgctgtagagttaaacgagcaagggaatatgctatttgtgagtggtaattttagtgaa

gcaagagacaaataccaacaggcttatgacatatctgaagttgctaaaaaaagtgatggttttttcaggaaattattata

tagtccccatgaatacaaaagtaatatcgataaagctcaaacagagttaaatgcaattgcattgaatgatcgtgctgacg

ttctcttaagtcagggtaagtttgctgaggctataaaaaagtatcaagcggcatatgataaaacacaagtatttaatcaa

cgtgaacaatataaaaataatagagattcaatacaggcaaaagtatataatcaagagggttataaattattaagtgaagc

tacagctgaacgagactttgctcttaaaattctcaaatataaaaatgcacaagaaaaatttgaaaaggctataaataaac

gtcctagtagtcatgtgtttagaaatagtctgactaaaactttagcagctatgaaggtgattgagacatataattctgta

ttcaaggactttagccttatgttaacttcagatatcacggatagtgagttagaagaactaggccatagattagatagttt

acttgatatagcggagtatggaaatgactctactattgtaaatagatttcatatagtatccttaagaatatccaggagga

aactgaatagtcaattagaatcaattaatcgaaaagatataaacgacctcatagaggcaattatgaacctaatagaaacc

gaaattgaggtattaaagcagctaaatattattgaccataagttggaacaagaattaaaatcattagaaggacaattaaa

tgctctagatctaaataatgaatgtatgcaaagcaataatgacaacatattagtaaatataacatcaattgcatctcgtg

ttgaggaatactatgaagaagtagagaacagggcattggagagattagcacaaaatgcacagcaagagcattgtggaaat

actctataactacaagacccattaagtgcttctatagttgaaacatacataatggatggaatgaatatgattttacaatt

aagaattgtatttagagaagcaggaaatacaaaacaaatcacataattatcttttaatatatgcctatatcgaaattaac

aatatgaacttacatgatctgttaaaccgtctggcacaagaaaccataaagaaaaccctaattccatttcttgttacaga

gaaacgccaatataggaggcaacgacagtcattaagtatatctaattgatgaacgtagcttggatggaattgtagtaagt

tatttaaatttagaaaacctaagtgtggatggattagaaattccagtagaacagaaactgtatgattgttgcggctctga

actaaatgaaaatatatacaacctacatataacaggatctaaggaatctactatttaccttcattcctcattacaagagc

agagttgaatattaactgatattgagatccatcaaaagaagaatacagattttgcttatggtatagaatggaacacagac

tatgaaaatgtagttacctcaacttgtgaatgtacaaaactcagtagttttattaaatacgatatggagaactgataaca

tactaaacagtaaatcaataatttattgaaaaataaagtattatggccaaataaaaatcagaacaaggaactcaaattaa

tcaagtaatgatggctgatgataaaattcaattgtcaaatgaacttaattctttactcgaagctttggtaaattacaaat

ttatttttgatgctagagtatccgataagattcctagctctttaccaagaactattactgtaaaactaaataaagcgaga

acattattatgtttagactataataataatgaatttgaaattgagaataatgacaaaataataaaaaagacagactatga

taacgttagaaacaccctgaaagataaaagaactcttattcacgaaaataaatggaatgaatcttttaagctggttaagc

aacaaattttgtcttatttaattaaacaaaatgaattagaggaccgagagaattttttaaaagaatttcaacaatttatc

gataaattaccggaagtaccgcatgaatttattgattgtactcgtaaacttaaggaaattaattttaatgattgggatat

tgaggaaagtgaagcaaatgatccacgcctgcaaactttagaagaaatacataaaaaaatgttagaatctttcgacttat

ataaggctataggcgaaaactatattaaactaagaaatcaagccgtaattgatccaaacgtaaatactccaaaagaggag

aaaaatcctgaagctgctcctgtacagaacacagatatcaatatgttcaatgtggtaaaaatccctagagatagtaattg

tttttatactgctgttgctgatcagcttagaagacttaaaataaaaaacaaaaaccctactaatttactagacacttact

atacctatgaagaccttagagcattagctatagattatataaagaaaaatagaaaacaagagtttggtaaagatattgaa

gaaagattgcaaactcaaaatacagaaccattacatggtgatttaccatcaaatatacataaaattgatcaatacatcga

gcttcacagtcaagaaggagtatgggctgattccggaatgattggggcattatcatatgcattagatattaccataaa

>Hypothenemus_hampei_contig_10251|c0_g1_i5 tetratricopeptide repeat family

ggcaagtatcgtgaagctcacagtaaatatcatgaagcatatgataaatctaatgttagtagacagcatcataaatatag

cactaacagagacaaagccaaagttgagctggatgcattaaatgcgtatcaacttggcgagatattgtttaataaaagaa

gatacaatgatgctttaaaggaatatcaggctgcccatagtagttctcaggtgataaatgcaaaatcaatttataatagt

ggaatatctaaagtacaagtagaaataattgctgagcagttatatgaacaaggtgaggtgttatattgtaccggaaagta

tgtggaagctaagacaaaatataatgaagcagtacatacatctaaagttcacaaggattactatagtagtttaggtgttg

taaaagctcaaacagagttagatgctgtagagttaaacgagcaagggaatatgctatttgtgagtggtaattttagtgaa

gcaagagacaaataccaacaggcttatgacatatctgaagttgctaaaaaaagtgatggttttttcaggaaattattata

tagtccccatgaatacaaaagtaatatcgataaagctcaaacagagttaaatgcaattgcattgaatgatcgtgctgacg

ttctcttaagtcagggtaagtttgctgaggctataaaaaagtatcaagcggcatatgataaaacacaagtatttaatcaa

cgtgaacaatataaaaataatagagattcaatacaggcaaaagtatataatcaagagggttataaattattaagtgaagc

tacagctgaacgagactttgctcttaaaattctcaaatataaaaatgcacaagaaaaatttgaaaaggctataaataaac

gtcctagtagtcatgtgtttagaaatagtctgactaaaactttagcagctatgaaggtgattgagacatataattctgta

ttcaaggactttagccttatgttaacttcagatatcacggatagtgagttagaagaactaggccatagattagatagttt

acttgatatagcggagtatggaaatgactctactattgtaaatagatttcatatagtatccttaagaatatccaggagga

aactgaatagtcaattagaatcaattaatcgaaaagatataaacgacctcatagaggcaattatgaacctaatagaaacc

gaaattgaggtattaaagcagctaaatattattgaccataagttggaacaagaattaaaatcattagaaggacaattaaa

tgctctagatctaaataatgaatgtatgcaaagcaataatgacaacatattagtaaatataacatcaattgcatctcgtg

ttgaggaatactatgaagaagtagagaacagggcattggagagattagcacaaaatgcacagcaagagcattgtggaaat

actctataactacaagacccattaagtgcttctatagttgaaacatacataatggatggaatgaatatgattttacaatt

aagaattgtatttagagaagcaggaaatacaaaacaaatcacataattatcttttaatatatgcctatatcgaaattaac

aatatgaacttacatgatctgttaaaccgtctggcacaagaaaccataaagaaaaccctaattccatttcttgttacaga

gaaacgccaatataggaggcaacgacagtcattaagtatatctaattgatgaacgtagcttggatggaattgtagtaagt

tatttaaatttagaaaacctaagtgtggatggattaggtgaatatattttatatgttttcaaagttacgtatcccaaata

taaattctcttttaaagaaattccagtagaacagaaactgtatgattgttgcggctctgaactaaatgaaaatatataca

acctacatataacaggatctaaggaatctactatttaccttcattcctcattacaagagcagagttgaatattaactgat

attgagatccatcaaaagaagaatacagattttgcttatggtatagaatggaacacagactatgaaaatgtagttacctc

aacttgtgaatgtacaaaactcagtagttttattaaatacgatatggagaactgataacatactaaacagtaagctgagc

agatagataagttataaactataaattatagagtatattttaggtaaatcaataatttattgaaaaataaagtattatgg

ccaaataaaaatcagaacaaggaactcaaattaatcaagtaatgatggctgatgataaaattcaattgtcaaatgaactt

aattctttactcgaagctttggtaaattacaaatttatttttgatgctagagtatccgataagattcctagctctttacc

aagaactattactgtaaaactaaataaagcgagaacattattatgtttagactataataataatgaatttgaaattgaga

ataatgacaaaataataaaaaagacagactatgataacgttagaaacaccctgaaagataaaagaactcttattcacgaa

aata

>Hypothenemus_hampei_contig_10251|c0_g1_i6 tetratricopeptide repeat family

ggcaagtatcgtgaagctcacagtaaatatcatgaagcatatgataaatctaatgttagtagacagcatcataaatatag

cactaacagagacaaagccaaagttgagctggatgcattaaatgcgtatcaacttggcgagatattgtttaataaaagaa

gatacaatgatgctttaaaggaatatcaggctgcccatagtagttctcaggtgataaatgcaaaatcaatttataatagt

ggaatatctaaagtacaagtagaaataattgctgagcagttatatgaacaaggtgaggtgttatattgtaccggaaagta

tgtggaagctaagacaaaatataatgaagcagtacatacatctaaagttcacaaggattactatagtagtttaggtgttg

taaaagctcaaacagagttagatgcaattgcattgaatgatcgtgctgacgttctcttaagtcagggtaagtttgctgag

gctataaaaaagtatcaagcggcatatgataaaacacaagtatttaatcaacgtgaacaatataaaaataatagagattc

aatacaggcaaaagtatataatcaagagggttataaattattaagtgaagctacagctgaacgagactttgctcttaaaa

ttctcagatataaaaacgcgcaagaaaaatttgaaaaggctataaataaacgtcctagttatcatgtgtatagaaatagt

ctgactaaagctttagcggctataaagatgattgagatatataattctgcattcaaggactttagccttatgttaacttc

agatatcacggatagtgagttagaagaactaggccatagattagatagtttacttgatatagcggagtatggaaatgact

ctactattgtaaatagatttcatatagtatccttaagaatatccaggaggaaactgaatagtcaattagaatcaattaat

cgaaaagatataaacgacctcatagaggcaattatgaacctaatagaaaccgaaattgaggtattaaagcagctaaatat

tattgaccataagttggaacaagaattaaaatcattagaaggacaattaaatgctctagatctaaataatgaatgtatgc

aaagcaataatgacaacatattagtaaatataacatcaattgcatctcgtgttgaggaatactatgaagaagtagagaac

agggcattggagagattagcacaaaatgcacagcaagagcattgtggaaatactctataactacaagacccattaagtgc

ttctatagttgaaacatacataatggatggaatgaatatgattttacaattaagaattgtatttagagaagcaggaaata

caaaacaaatcacataattatcttttaatatatgcctatatcgaaattaacaatatgaacttacatgatctgttaaaccg

tctggcacaagaaaccataaagaaaaccctaattccatttcttgttacagagaaacgccaatataggaggcaacgacagt

cattaagtatatctaattgatgaacgtagcttggatggaattgtagtaagttatttaaatttagaaaacctaagtgtgga

tggattagaaattccagtagaacagaaactgtatgattgttgcggctctgaactaaatgaaaatatatacaacctacata

taacaggatctaaggaatctactatttaccttcattcctcattacaagagcagagttgaatattaactgatattgagatc

catcaaaagaagaatacagattttgcttatggtatagaatggaacacagactatgaaaatgtagttacctcaacttgtga

atgtacaaaactcagtagttttattaaatacgatatggagaactgataacatactaaacagtaagctgagcagatagata

agttataaactataaattatagagtatattttaggtaaatcaataatttattgaaaaataaagtattatggccaaataaa

aatcagaacaaggaactcaaattaatcaagtaatgatggctgatgataaaattcaattgtcaaatgaacttaattcttta

ctcgaagctttggtaaattacaaatttatttttgatgctagagtatccgataagattcctagctctttaccaagaactat

tactgtaaaactaaataaagcgagaacattattatgtttagactataataataatgaatttgaaattgagaataatgaca

aaataataaaaaagacagactatgataacgttagaaacaccctgaaagataaaagaactcttattcacgaaaata

>Hypothenemus_hampei_contig_10251|c0_g1_i7 tetratricopeptide repeat family

ggcaagtatcgtgaagctcacagtaaatatcatgaagcatatgataaatctaatgttagtagacagcatcataaatatag

cactaacagagacaaagccaaagttgagctggatgcattaaatgcgtatcaacttggcgagatattgtttaataaaagaa

gatacaatgatgctttaaaggaatatcaggctgcccatagtagttctcaggtgataaatgcaaaatcaatttataatagt

ggaatatctaaagtacaagtagaaataattgctgagcagttatatgaacaaggtgaggtgttatattgtaccggaaagta

tgtggaagctaagacaaaatataatgaagcagtacatacatctaaagttcacaaggattactatagtagtttaggtgttg

taaaagctcaaacagagttagatgctgtagagttaaacgagcaagggaatatgctatttgtgagtggtaattttagtgaa

gcaagagacaaataccaacaggcttatgacatatctgaagttgctaaaaaaagtgatggttttttcaggaaattattata

tagtccccatgaatacaaaagtaatatcgataaagctcaaacagagttaaatgcaattgcattgaatgatcgtgctgacg

ttctcttaagtcagggtaagtttgctgaggctataaaaaagtatcaagcggcatatgataaaacacaagtatttaatcaa

cgtgaacaatataaaaataatagagattcaatacaggcaaaagtatataatcaagagggttataaattattaagtgaagc

tacagctgaacgagactttgctcttaaaattctcaaatataaaaatgcacaagaaaaatttgaaaaggctataaataaac

gtcctagtagtcatgtgtttagaaatagtctgactaaaactttagcagctatgaaggtgattgagacatataattctgta

ttcaaggactttagccttatgttaacttcagatatcacggatagtgagttagaagaactaggccatagattagatagttt

acttgatatagcggagtatggaaatgactctactattgtaaatagatttcatatagtatccttaagaatatccaggagga

aactgaatagtcaattagaatcaattaatcgaaaagatataaacgacctcatagaggcaattatgaacctaatagaaacc

gaaattgaggtattaaagcagctaaatattattgaccataagttggaacaagaattaaaatcattagaaggacaattaaa

tgctctagatctaaataatgaatgtatgcaaagcaataatgacaacatattagtaaatataacatcaattgcatctcgtg

ttgaggaatactatgaagaagtagagaacagggcattggagagattagcacaaaatgcacagcaagagcattgtggaaat

actctataactacaagacccattaagtgcttctatagttgaaacatacataatggatggaatgaatatgattttacaatt

aagaattgtatttagagaagcaggaaatacaaaacaaatcacataattatcttttaatatatgcctatatcgaaattaac

aatatgaacttacatgatctgttaaaccgtctggcacaagaaaccataaagaaaaccctaattccatttcttgttacaga

gaaacgccaatataggaggcaacgacagtcattaagtatatctaattgatgaacgtagcttggatggaattgtagtaagt

tatttaaatttagaaaacctaagtgtggatggattaggtgaatatattttatatgttttcaaagttacgtatcccaaata

taaattctcttttaaagaaattccagtagaacagaaactgtatgattgttgcggctctgaactaaatgaaaatatataca

acctacatataacaggatctaaggaatctactatttaccttcattcctcattacaagagcagagttgaatattaactgat

attgagatccatcaaaagaagaatacagattttgcttatggtatagaatggaacacagactatgaaaatgtagttacctc

aacttgtgaatgtacaaaactcagtagttttattaaatacgatatggagaactgataacatactaaacagtaaatcaata

atttattgaaaaataaagtattatggccaaataaaaatcagaacaaggaactcaaattaatcaagtaatgatggctgatg

ataaaattcaattgtcaaatgaacttaattctttactcgaagctttggtaaattacaaatttatttttgatgctagagta

tccgataagattcctagctctttaccaagaactattactgtaaaactaaataaagcgagaacattattatgtttagacta

taataataatgaatttgaaattgagaataatgacaaaataataaaaaagacagactatgataacgttagaaacaccctga

aagataaaagaactcttattcacgaaaataaatggaatgaatcttttaagctggttaagcaacaaattttgtcttattta

attaaacaaaatgaattagaggaccgagagaattttttaaaagaatttcaacaatttatcgataaattaccggaagtacc

gcatgaatttattgattgtactcgtaaacttaaggaaattaattttaatgattgggatattgaggaaagtgaagcaaatg

atccacgcctgcaaactttagaagaaatacataaaaaaatgttagaatctttcgacttatataaggctataggcgaaaac

tatattaaactaagaaatcaagccgtaattgatccaaatgtaaatactccagaagaggagaaaaatcctgaagctgctcc

tgtacagaacacagatatcaatatgttcaatgtggtaaaaatccctagagatagtaattgtttttatactgctgttgctg

atcagcttagaagacttaaaataaaaaacaaaaaccctactaatttactagacacttactatacctatgaagaccttaga

gcattagctatagattatataaagaaaaatagaaaacaagagtttggtaaagatattgaagaaagattgcaaactcaaaa

tacagaaccattacatggtgatttaccatcaaatatacataaaattgatcaatacatcgagcttcacagtcaagaaggag

tatgggctgattccggaatgattggggcattatcatatgcattagatattaccataaa

>Hypothenemus_hampei_contig_10251|c0_g1_i8 tetratricopeptide repeat family

ggcaagtatcgtgaagctcacagtaaatatcatgaagcatatgataaatctaatgttagtagacagcatcataaatatag

cactaacagagacaaagccaaagttgagctggatgcattaaatgcgtatcaacttggcgagatattgtttaataaaagaa

gatacaatgatgctttaaaggaatatcaggctgcccatagtagttctcaggtgataaatgcaaaatcaatttataatagt

ggaatatctaaagtacaagtagaaataattgctgagcagttatatgaacaaggtgaggtgttatattgtaccggaaagta

tgtggaagctaagacaaaatataatgaagcagtacatacatctaaagttcacaaggattactatagtagtttaggtgttg

taaaagctcaaacagagttagatgcaattgcattgaatgatcgtgctgacgttctcttaagtcagggtaagtttgctgag

gctataaaaaagtatcaagcggcatatgataaaacacaagtatttaatcaacgtgaacaatataaaaataatagagattc

aatacaggcaaaagtatataatcaagagggttataaattattaagtgaagctacagctgaacgagactttgctcttaaaa

ttctcaaatataaaaatgcacaagaaaaatttgaaaaggctataaataaacgtcctagtagtcatgtgtttagaaatagt

ctgactaaaactttagcagctatgaaggtgattgagacatataattctgtattcaaggactttagccttatgttaacttc

agatatcacggatagtgagttagaagaactaggccatagattagatagtttacttgatatagcggagtatggaaatgact

ctactattgtaaatagatttcatatagtatccttaagaatatccaggaggaaactgaatagtcaattagaatcaattaat

cgaaaagatataaacgacctcatagaggcaattatgaacctaatagaaaccgaaattgaggtattaaagcagctaaatat

tattgaccataagttggaacaagaattaaaatcattagaaggacaattaaatgctctagatctaaataatgaatgtatgc

aaagcaataatgacaacatattagtaaatataacatcaattgcatctcgtgttgaggaatactatgaagaagtagagaac

agggcattggagagattagcacaaaatgcacagcaagagcattgtggaaatactctataactacaagacccattaagtgc

ttctatagttgaaacatacataatggatggaatgaatatgattttacaattaagaattgtatttagagaagcaggaaata

caaaacaaatcacataattatcttttaatatatgcctatatcgaaattaacaatatgaacttacatgatctgttaaaccg

tctggcacaagaaaccataaagaaaaccctaattccatttcttgttacagagaaacgccaatataggaggcaacgacagt

cattaagtatatctaattgatgaacgtagcttggatggaattgtagtaagttatttaaatttagaaaacctaagtgtgga

tggattaggtgaatatattttatatgttttcaaagttacgtatcccaaatataaattctcttttaaagaaattccagtag

aacagaaactgtatgattgttgcggctctgaactaaatgaaaatatatacaacctacatataacaggatctaaggaatct

actatttaccttcattcctcattacaagagcagagttgaatattaactgatattgagatccatcaaaagaagaatacaga

ttttgcttatggtatagaatggaacacagactatgaaaatgtagttacctcaacttgtgaatgtacaaaactcagtagtt

ttattaaatacgatatggagaactgataacatactaaacagtaagctgagcagatagataagttataaactataaattat

agagtatattttaggtaaatcaataatttattgaaaaataaagtattatggccaaataaaaatcagaacaaggaactcaa

attaatcaagtaatgatggctgatgataaaattcaattgtcaaatgaacttaattctttactcgaagctttggtaaatta

caaatttatttttgatgctagagtatccgataagattcctagctctttaccaagaactattactgtaaaactaaataaag

cgagaacattattatgtttagactataataataatgaatttgaaattgagaataatgacaaaataataaaaaagacagac

tatgataacgttagaaacaccctgaaagataaaagaactcttattcacgaaaata

>Hypothenemus_hampei_contig_10251|c0_g1_i9 tetratricopeptide repeat family

ggcaagtatcgtgaagctcacagtaaatatcatgaagcatatgataaatctaatgttagtagacagcatcataaatatag

cactaacagagacaaagccaaagttgagctggatgcattaaatgcgtatcaacttggcgagatattgtttaataaaagaa

gatacaatgatgctttaaaggaatatcaggctgcccatagtagttctcaggtgataaatgcaaaatcaatttataatagt

ggaatatctaaagtacaagtagaaataattgctgagcagttatatgaacaaggtgaggtgttatattgtaccggaaagta

tgtggaagctaagacaaaatataatgaagcagtacatacatctaaagttcacaaggattactatagtagtttaggtgttg

taaaagctcaaacagagttagatgcaattgcattgaatgatcgtgctgacgttctcttaagtcagggtaagtttgctgag

gctataaaaaagtatcaagcggcatatgataaaacacaagtatttaatcaacgtgaacaatataaaaataatagagattc

aatacaggcaaaagtatataatcaagagggttataaattattaagtgaagctacagctgaacgagactttgctcttaaaa

ttctcaaatataaaaatgcacaagaaaaatttgaaaaggctataaataaacgtcctagtagtcatgtgtttagaaatagt

ctgactaaaactttagcagctatgaaggtgattgagacatataattctgtattcaaggactttagccttatgttaacttc

agatatcacggatagtgagttagaagaactaggccatagattagatagtttacttgatatagcggagtatggaaatgact

ctactattgtaaatagatttcatatagtatccttaagaatatccaggaggaaactgaatagtcaattagaatcaattaat

cgaaaagatataaacgacctcatagaggcaattatgaacctaatagaaaccgaaattgaggtattaaagcagctaaatat

tattgaccataagttggaacaagaattaaaatcattagaaggacaattaaatgctctagatctaaataatgaatgtatgc

aaagcaataatgacaacatattagtaaatataacatcaattgcatctcgtgttgaggaatactatgaagaagtagagaac

agggcattggagagattagcacaaaatgcacagcaagagcattgtggaaatactctataactacaagacccattaagtgc

ttctatagttgaaacatacataatggatggaatgaatatgattttacaattaagaattgtatttagagaagcaggaaata

caaaacaaatcacataattatcttttaatatatgcctatatcgaaattaacaatatgaacttacatgatctgttaaaccg

tctggcacaagaaaccataaagaaaaccctaattccatttcttgttacagagaaacgccaatataggaggcaacgacagt

cattaagtatatctaattgatgaacgtagcttggatggaattgtagtaagttatttaaatttagaaaacctaagtgtgga

tggattaggtgaatatattttatatgttttcaaagttacgtatcccaaatataaattctcttttaaagaaattccagtag

aacagaaactgtatgattgttgcggctctgaactaaatgaaaatatatacaacctacatataacaggatctaaggaatct

actatttaccttcattcctcattacaagagcagagttgaatattaactgatattgagatccatcaaaagaagaatacaga

ttttgcttatggtatagaatggaacacagactatgaaaatgtagttacctcaacttgtgaatgtacaaaactcagtagtt

ttattaaatacgatatggagaactgataacatactaaacaagtatattttaggtaaatcaataatttattgaaaaataaa

gtattatggccaaataaaaatcagaacaaggaactcaaattaatcaagtaatgatggctgatgataaaattcaattgtca

aatgaacttaattctttactcgaagctttggtaaattacaaatttatttttgatgctagagtatccgataagattcctag

ctctttaccaagaactattactgtaaaactaaataaagcgagaacattattatgtttagactataataataatgaatttg

aaattgagaataatgacaaaataataaaaaagacagactatgataacgttagaaacaccctgaaagataaaagaactctt

attcacgaaaata

>Hypothenemus_hampei_contig_10260|c0_g1_i1 Mitochondrial substrate carrier

tacgcatccaattccatcggaaatccatctatccatcaattgcagccactaactgattcatagtgggtggtgtttccgcc

gctgtctccaagaccgccgctgcccccattgagcgtatcaagctcctgatccagaaccaggatgagatgctcaaggccgg

tcgtctcgaccgcaagtacgccggtatcggtgactgcttcaagcgtaccgccgctcaggagggtgttgtctcgctgtggc

gtggtaacaccgccaacgtcatccgttacttccccacccaggctcttaacttcgctttccgtgacacctacaagtccatg

ttcgcttacaagaaggagcgtgatggatacggcaagtggatgatgggtaaccttgcctccggtggtgccgctggtgccac

ttccctcctgttcgtctactctctggactacgcccgtacccgtctggccaacgacgccaagtcctcaaagggctccggtg

agcgccagttcaacggtctggttgatgtctac

>Hypothenemus_hampei_contig_10260|c0_g2_i1 ADP,ATP carrier

ccccatctcctctcttttttttagttgtcgcatccatcttttgcacaacctttcacaatggctcaaggtgacgttaacca

gggcgagaagtccgtcttcggcatgcccggcttcgttgtcgacttcctgatgggtggtgtttccgctgctgtctccaaga

ctgctgccgcccccatcgagcgtatcaagctcctcatccagaaccaggatgagatgcttcgcgccggtcgtctcgaccgc

aagtacaacggtatcactgactgcttccgtcgtaccgccgcctccgagggtgtcgtctccctgtggagaggtaacactgc

caacgtcatccgttacttccctacccaggctctcaacttcgccttccgtgacacctacaagtccatgttctcctacaaga

aggatcgtgatggatatgccaagtggatgatgggtaaccttgcctccggtggtgctgctggtgccacttccctcctcttc

gtctactctctcgactacgcccgtacccgtcttgccaacgacgccaagtccgccaagggcggtggtgaccgtcagttcaa

cggtctcgttgacgtctacaagaagaccctcgcctccgacggtattgccggtctctaccgtggtttcggtccctccgttc

tcggaatcgttgtctaccgtggtctgtacttcggtatgtacgactctatcaagcccgttctcctggtcggtcctcttgag

ggctctttcctcgcttccttcctgctcggctggactgttaccactgctgctggtgttgcttcctaccctctggacactat

ccgtcgtcgtatgatgatgacctctggtgaggccgtcaagtactcctcctccatggatgctgcccgccagatcatcgcca

aggagggtaccaagtccctgttcaagggtgccggtgccaacatcctccgtggtgttgccggtgctggtgtcctgtccatc

tacgatcaggtccagctgatcctcttcggcaagaagttcaaatagatgactcgcccctagcgaattatcttaaaacacct

gtagctatactggggatggggatgaaaccctgctcggtgtaaccgggtgatggggaagcagaggatgtgggaactgcaaa

agatagatgcgtcaagaaatgggttcggggggatcttgaagcatagaatggtgtgattcttccctttttgtaccc

>Hypothenemus_hampei_contig_10260|c0_g2_i2 ADP,ATP carrier

ccccatctcctctcttttttttagttgtcgcatccatcttttgcacaacctttcacaatggctcaaggtgacgttaacca

gggcgagaagtccgtcttcggcatgcccggcttcgttgtcgacttcctgatgggtggtgtttccgccgctgtctccaaga

ccgccgctgcccccattgagcgtatcaagctcctgatccagaaccaggatgagatgctcaaggctggtcgtctttcccac

aagtacaacggtctcgccgactgcttcaagcgtactgccgctgatgagggtatcgtctctctgtggcgtggtaacaccgc

caacgtcatccgttacttccctacccaggccctgaacttcgctttccgtgacacctacaagtccatgttcgcctacaaga

aggaccgtgatggctacgccaagtggatgatgggtaacctggcctccggtggtgctgccggtgccacttccctgctgttc

gtctactccctggactacgcccgtacccgtctggccaacgacgctaagtccaccaagggtggcggtgaccgtcagttcaa

cggtct

>Hypothenemus_hampei_contig_10260|c0_g2_i3 ADP,ATP carrier

ccccatctcctctcttttttttagttgtcgcatccatcttttgcacaacctttcacaatggctcaaggtgacgttaacca

gggcgagaagtccgtcttcggcatgcccggcttcgttgtcgacttcctgatgggtggtgtttccgctgctgtctccaaga

ctgctgccgcccccatcgagcgtatcaagctcctcatccagaaccaggatgagatgcttcgcgccggtcgtctcgaccgc

aagtacaacggtatcactgactgcttccgtcgtaccgccgcctccgagggtgtcgtctccctgtggagaggtaacactgc

caacgtcatccgttacttccctacccaggctctcaacttcgccttccgtgacacctacaagtccatgttctcctacaaga

aggagcgtgatggatacgccaagtggatgatgggtaacctggcctccggtggtgctgccggtgccacttccctgctgttc

gtctactccctggactacgcccgtacccgtctggccaacgacgctaagtccaccaagggtggcggtgaccgtcagttcaa

cggtct

>Hypothenemus_hampei_contig_1028|c0_g1_i1 malate dehydrogenase

actactgttaaaaacccaactgccttcaggttcagaactctctctgtatgatatcgctccagtgactcccggtgtggctg

tcgatctgagccatatccctactgctgtgaaaatcaaaggtttttctggtgaagatgcgactccggcgctggaaggcgca

gatgtcgttcttatctctgcaggcgtagcgcgtaaaccgggtatggatcgttccgacctgtttaacgttaacgccggcat

cgtgaaaaacctggtacagcaagttgcgaaaacctgcccgaaagcgtgcattggtattatcactaacccggttaacacca

cagttgcaattgctgctgaagtgctgaaaaaagccggtgtttatgacaaaaacaaactgttcggcgttaccacgctggat

atcattcgttccaacacctttgttgcggaactgaaaggcaaacagccaggcgaagttgaagtgccggttattggcggtca

ctctggtgttaccattctgccgctgctgtcacaggttcctggcgttagttttaccgagcaggaagtggctgatctgacca

aacgcatccagaacgcgggtactgaagtggttgaagcgaaggccggtggcgggtctgcaaccctgtctatgggccaggca

gctgcacgttttggtctgtctctggttcgtgcactgcagggcgaacaaggcgttgtcgaatgtgcctacgttgaaggcga

cggtcagtacgcccgtttcttctctcaaccgctgctgctgggtaaaaacggcgtggaaga

>Hypothenemus_hampei_contig_10315|c0_g1_i1 hypothetical protein G7K_1412-t1

cttggagggcaagtctggtgccagcagccgcggtaattccagctccaatagcgtatattaaagttgttgcagttaaaaag

ctcgtagttgaaccttgggcccgtcctgccggtccgcctcaccgcgagtactggtccggatgggtctttctttctgggga

atcccatggccttcactggctgtggtggggaaccaggacttttactgtgaaaaaattagagtgttcaaagcaggcctttg

ctcggatacattagcatggaataataaaataggacgtgcggttctattttgttggtttctaggaccgccgtaatgattaa

tagggacagtcgggggcatcagtattcaattgtcagaggtgaaattcttggatttgctgaagactaactactgcgaaagc

attcgccaaggatgttttcattaatcaggaacgaaagttaggggatcgaagacgatcagataccgtcgtagtcttaacca

taaactatgc

>Hypothenemus_hampei_contig_10315|c0_g1_i2 hypothetical protein G7K_1412-t1

ggtccggccgggcctttccctctggggaaccgcatgcccttcactgggtgtgtcggggaaccaggacttttaccttgaaa

aaattagatcgcttaaagaaggcctatgctcgaatacattagcatggaataatagaataggacgtgcggttctattttgt

tggtttctaggaccgccgtaatgattaatagggacagtcgggggcatcagtattcaattgtcagaggtgaaattcttgga

tttgctgaagactaactactgcgaaagcattcgccaaggatgttttcattaatcaggaacgaaagttaggggatcgaaga

cgatcagataccgtcgtagtcttaaccataaactatgc

>Hypothenemus_hampei_contig_10315|c0_g1_i3 hypothetical protein ACN38_g13110

agccgcggtaattccagctccaatagcgtatattaaagttgttgcagttaaaaagctcgtagttgaactttgggcttggt

tggccggtccgcctttttggcgagtactggacccaaccgagcctttccttctggctaaccattcgcccttgtggtgtttg

gcgaaccaggacttttactttgaaaaaattagagtgttcaaagcaggcctttgctcgaatatattagcatggaataatag

aataggacgtgcggttctattttgttggtttctaggaccgccgtaatgattaatagggatagtcgggggcgtcagtattc

agctgtcagaggtgaaattcttggatttgctgaagactaactactgcgaaagcattcgccaaggatgttttcattaatca

gggaacgaaagttaggggatcgaagacgatcagataccgtcgtagtcttaaccataaactatgccgactagggatcggac

gggattctatgatgacccgttcggcaccttacgagaaatcaaagtttttgggttctggggggagtatggtcgcaaggctg

aaacttaaaggaattgacggaagggcaccaccaggagtggagcctgcggcttaatt

>Hypothenemus_hampei_contig_10315|c0_g1_i4 rRNA intron-encoded homing partial

ggtccggccgggcctttccctctggggaaccgcatgcccttcactgggtgtgtcggggaaccaggacttttaccttgaaa

aaattagatcgcttaaagaaggcctatgctcgaatacattagcatggaataatagaataggacgtgcggttctattttgt

tggtttctaggaccgccgtaatgattaatagggacagtcgggggcatcagtattcaattgtcagaggtgaaattcttgga

tttatgaaagacgaacaactgcgaaagcatttgccaaggatgttttcattaatcaagaacgaaagttgggggctcgaaga

cgatcagataccgtcctagtctcaaccataaacgatgccgaccagggatcggcggatgttactttaaggactccgccggc

accttatgagaaatcaaagtttttgggttccggggggagtatggtcgcaaggctgaaacttaaaggaattgacggaaggg

caccaccaggagtggagcctgcggcttaatt

>Hypothenemus_hampei_contig_10315|c0_g1_i5 hypothetical protein ACN38_g13110

cttggagggcaagtctggtgccagcagccgcggtaattccagctccaagagcgtatgtaaaagttgttgcagttaaaaag

ctcgtagttgaaccttgggattggttggccggtccgcttttttgcgagtactggattcaaccgatcctttccttctggct

aactagtcgttctgcggctagcgaaccaggacatttactttgagaaaattagagtgttcaaagcaggcattagcttgaat

atattagcatggaataatagaataggacgtgcggttctattttgttggtttctaggaccgccgtaatgattaatagggat

agtcgggggcgtcagtattcagctgtcagaggtgaaattcttggatttgctgaagactaactactgcgaaagcattcgcc

aaggatgttttcattaatcagggaacgaaagttaggggatcgaagacgatcagataccgtcgtagtcttaaccataaact

atgccgactagggatcggacgggattctatgatgacccgttcggcaccttacgagaaatcaaagtttttgggttctgggg

ggagtatggtcgcaaggctgaaacttaaaggaattgacggaagggcaccaccaggagtggagcctgcggcttaatt

>Hypothenemus_hampei_contig_10315|c0_g1_i6 hypothetical protein ACN38_g13110

cttggagggcaagtctggtgccagcagccgcggtaattccagctccaatagcgtatattaaagttgttgcagttaaaaag

ctcgtagttgaaccttgggtctggctgaccggtccgcctcaccgcgagtactggtccggctggacctttccttctgggga

accctatggccttcactggctgtagggggaaccaggacttttactgtgaaaaaattagagtgttcaaagcaggcctttgc

tcgaatacattagcatggaataatagaataggacgtgcggttctattttgttggtttctaggaccgccgtaatgattaat

agggatagtcgggggcgtcagtattcagctgtcagaggtgaaattcttggatttgctgaagactaactactgcgaaagca

ttcgccaaggatgttttcattaatcagggaacgaaagttaggggatcgaagacgatcagataccgtcgtagtcttaacca

taaactatgccgactagggatcggacgggattctatgatgacccgttcggcaccttacgagaaatcaaagtttttgggtt

ctggggggagtatggtcgcaaggctgaaacttaaaggaattgacggaagggcaccaccaggagtggagcctgcggcttaa

tt

>Hypothenemus_hampei_contig_10315|c0_g1_i7 hypothetical protein L484_002552

cttggagggcaagtctggtgccagcagccgcggtaattccagctccaatagcgtatattaaagttgttgcagttaaaaag

ctcgtagttggactttgggatgggccggccggtccgccgtacggtgtgcacctgtcgtctcgtcccttctgccggcgatg

cgctcctggccttaactggccgggtcgtgcctccggcgctgttactttgaagaaattagagtgttcaaagcaagcctacg

ctctgaatacattagcatgggataacattataggatttcggtcctattacgttggccttcgggatcggagtaatgattaa

cagggacagtcgggggcattcgtatttcatagtcagaggtgaaattcttggatttatgaaagacgaacaactgcgaaagc

atttgccaaggatgttttcattaatcaagaacgaaagttgggggctcgaagacgatcagataccgtcctagtctcaacca

taaacgatgccgaccagggatcggcggatgttactttaaggactccgccggcaccttatgagaaatcaaagtttttgggt

tccggggggagtatggtcgcaaggctgaaacttaaaggaattgacggaagggcaccaccaggagtggagcctgcggctta

att

>Hypothenemus_hampei_contig_10352|c0_g1_i1 transcription elongation factor S-II

atgcaaggacatccacccttctgcagcgctcgaggaggtggcaacgggagacgcgcagaaggataggtccattaaaatgt

ttttcgttgcttttaagacaaatatccccggctgtagcaacaggtctgccgcgcttcttgcagggcagataaccgccgag

atatttgcgggaagtccatcggagatagcaaggcttgtacggagcaagtgcctgaacttgaaggacaggaacaacccggc

gctgtgcaggcgcgtgtacggcggggagatctctccctcgcgatacgttgcaatgacgtccgaggagatgaagagcgagc

tgctgaggagttcggaggtcaagatgatcgaggacagcctgtacgagtgccagatcccgacgcagaaggccgagacagac

atgttcaagtgcagccgctgcggcgagaggaagtgctcgtacaggcagctgcagacacgctctggggacgagcccatgac

tacgtttgtgacgtgcgagtgcggaaacaagtggaggttctgttgataggcacatgaggccctgcagcccatg

>Hypothenemus_hampei_contig_10387|c0_g1_i1 hypothetical protein P875_00127701

gaggacccctgctgatagattcatcaagtatcatcaccacactacttgaccatcatcattttgctacaatgagtagtaac

aaccagcaacccgagtcaccccgccattggcatggtgatatcggcaatcgtcacgagcatcataatgtctgggtaagagg

cggtcctgcgcaggcctaccagggccggcgaccttctatgactgaagctgtcaatgccatggctgatagacgtgactcca

catcttcgtcgacttctgcgaataacaacaacagtcctcccactgctccggagagaagacgatctagcggccacggaccg

tcgactcttttcgagagtctgacgagccagaaacggaactcaaccgatgcagcccgtcgggcaagctaccatgaacagtc

tcagcagggcggctttttctcgaaatggtgggacggatatacgcgtggcaattaatttggctttgtagtgtgaagaaacg

tatccttataggaaagtaccagatctgttgggca

>Hypothenemus_hampei_contig_10387|c0_g2_i1 hypothetical protein P875_00127701

gaggacccctgctgatagattcatcaagtatcatcaccacactacttgaccatcatcattttgctacaatgagtagtaac

aaccagcaacccgagtcaccccgccattggcatggtgatatcggcaatcgtcacgagcatcataatgtctgggtaagagg

cggtcctgcgcaggcctaccagggccggcgaccttctatgactgaagctgtcaatgccatggctgatagacgtgactcca

catcttcgtcgacttctgcgaataacaacaacagtcctcccactgctccggagagaagacgatctagcggccacggaccg

tcgactcttttcgagagtctgacgagccagaaacggaactcaaccgatgcagcccgtcgggcaagctaccatgaacagtc

tcagcagggcggctttttctcgaaatggtgggacggatatacgcgtggcaattaaaccgtcttgtagttggacggtattg

ttctggggaaatgatttcgtcggtcgcatgtgataggacagtcattttgttccaagttgtttgatgcatttgccctgata

tgctcaagctgtgatgctgcgttgctttccttggtttccaagaggcactggatggaaagat

>Hypothenemus_hampei_contig_10396|c0_g1_i1 endoplasmic reticulum membrane-associated oxidoreductin

ctgggagatctcaagcaaggacagcctgctccccacgctcgtcagcgggctccagttctcgattctcacccacctctgct

cgttccacaggaggttcctcgggacatacctcccaaacccccttctcttcctcagaagattcagagacccccacaggctg

aacttccacctgacctacctgcttgtacgtagctgtgtcggcaccatggaggccgacccagacctacagggcatagctcg

cacaatagcggcacaggggctctggatccccggggacgtggatctgtcggaatccatcaggaggatcgacgagatggtcg

ggctcctgagacacgtggactgcgagaagtgccagctgtgggggaccatccagctcaaggggctgcgagcagcgctcaag

gcagcttctggcgcccccctcgacggcctcgagaggttcttcctcgtcaacctgttcatgaggctctctgtgtctgtcgg

ggaaagcatcaagctcaggaggttctgggccccccgccttgtcacggcagccctctactgggtggaggtgctgtcgcttg

ccgcatccgttgcagcaatcctcttggcccggggaatccgcagaaggctcaagagcaaggttgctctcaagagctgtggg

tgaccaattaaaccccttgtatccgacacaccggcagctgtcgttgccgatataaaccaggcgccaatagcccctatgaa

cagga

>Hypothenemus_hampei_contig_10399|c0_g1_i1 DNA replication licensing factor Mcm7

gaggttgacagaatcagcatccatgaggtgatggagcagcagagtgtgtctgtcagcaaggcgggcattaatacaaacct

gaatgcaaggtgttctgttcttggggcggcaaacccaataaagggccgatacgatgcgagatacagcatcgagcacaacg

tcgggcttccctgtgcgcttctttcccgattcgacgttcttgccattttaagagacgatccggacctagaaagggatgag

aggcttgccaaccacatcacctcgatccatctcgacgagaggcccgaatcgatcccatacgatgtaataagattggtgat

tgatgaagcaaagaagctggatcccattctcccctcccacctctccagcaagctgacggatgcatatgtaaaggccagga

aggaaaacccgtatgtaacgcccaggtaccttctatcgctcataaggctaagccttgcgcatgcccggctcaggctccgt

ggggaggttggggagggcgacgtcgacgaagccctgcggcttatggaagtgacaaaggtccctgtgacgaagaagaagaa

ggaggaggtctccagcaaaagagcaatatacaacctcattctctcacttgcagtggaggagggcagcaaaaggtgtgtca

agctggctcatctatgggatgccacaaagggcaagtacagcggaagcgaggtcgaggatgtcatttcggactttgcttca

tgtgggatctggattagaaacaacgaggaactggttatctttaattaaaggcattgttggagaatggcttttaatagaaa

aa

>Hypothenemus_hampei_contig_10399|c0_g2_i1 DNA replication licensing factor Mcm7

gcatatgtaaaggccaggaaggagaacccgtatgtaacgcccaggtaccttctatcgctcataaggctaagccttgcgca

tgcccggctcaggctccgtggggaggttggggagggcgacgtcgacgaagccctgcggcttatggaagtgacaaaggtcc

ctgtgacgaagaagaagaaggaggaggtctccagcaaaagagcaatatacaacctcattctctcacttgcagtggaggag

ggcagcaaaaggtgtgtcaagctggctcatctatgggatgccacaaagggcaagtacagcggaagcgaggtcgaggatgt

catttcggactttgcttcatgtgggatctggattagaaacaacgaggaactggttatctttaattaaaggcattgttgga

gaatggcttttaatagaaaaa

>Hypothenemus_hampei_contig_10402|c0_g1_i1 hypothetical protein M970_091730

caatgtggacggactcagtcttatgaagagggtggataagagggtgtcctcgctgctgtttgctggagacctgtatgtgg

tcactgagaagaagctgtatgggttccacgatgtgatcaattcgccagactcgctttccggcctgctagagccggagagg

aagaggaagaaggcaagcgggtctctactcagcgacgagtctcatgaggaatatacgtatagactctcgaggaaaggaca

aagaatcgtcttcaaaagggaggaggggaagtctgacatagcggacaactgggaggggttctttgtggcagcaggcctgg

tccacaaggtggtatgcgaaggcgagagctacggctttgtatataactccaggaagcattcctgtgacaaggaaatcaag

ggaatcgtgtacagcggcggcctgctggcttactaccagaagggaaggaactccgtgctgtgcctgcagggggagcaaga

gaagtcatatgccattccgaagatcacagcaatgtcgtgtgaaggccagtatacaaccgtcggaacgggagacgggtatg

tctatctcttcaacggccttgctctgagggggaggaagaaggtgtgtgacttcccaataacaggagttgggttcatggaa

gggcatgtttatttctcctctctggatggccttgtcggggccaaaaagatgcctggcgagtgtaagtccccctgcatcgc

aatgtctgtcatcatctttgtccttgcagtgctggttggcctgctcatcaaaagcggcaggattcccttcgtagccaaat

aaagcctttgcttgacacctgggggttcaggcccactggtcgaacaccagcctcttggtctctacaacagccgggagctc

cttcctgaacatctcaaatacctgtctttgcatgtccatccttccacacacataca

>Hypothenemus_hampei_contig_10457|c0_g1_i1 40S ribosomal S12

aagaatccggttctatctctattgaagatgctttaaaagttgtcttaagaacttctttagttcacgatggtttagctaga

ggtttaagagaagcttctaaagctttatctagaagagaagctcaattatgtgtcttatgtgactctgttactgaagaatc

tattattaaattggttgaagctttatgtaacgaaccagaagaaaaaatcccattgattaaagtttctgacgctaaattat

taggtgaatgggccggtctttgtcaattagacagagaaggtaacgccagaaaagttgttggtgcttcttgtgttgtcatc

aagaactggggtaccgatactgaagaaagaaacatcttattagaacacttctcccaacaataagcgagtcgtatctaagt

tgaacatgtttctctttaggcctttttaggcacttaccaaaaaaatatagt

>Hypothenemus_hampei_contig_10457|c0_g2_i1 40S ribosomal S12

aacttacttattactaaaactatttaaaatgtctgacattgaagaacaaccagttgtcgaagaagttgccgttgaacaaa

gcgaagaaatctccatcgaagatgccttaaaggttgtcttgagaacctctttggttcacgatggtttagctagaggttta

agagaagcctctaagaccttatctagaggtgaagcccaattatgtgtcttgtgtgaatccgtcactgaagactctattgt

taagttggtcgaagctttatgtaacgaaccagaacaaaagattccattaatcaaggtctctgacgctaagcaattaggtg

aatgggctggtctttgtcaattagacagagaaggtaacgccagaaaggtcgttggtgcttcttgtgtcgttgtcaagaac

tggggtgctgactctgaagaaagaaacatgttgttggaacactttgcccaac

>Hypothenemus_hampei_contig_10489|c0_g1_i1 40S ribosomal S1

acctttgtctgtcttcaactaactaacagatagacccgattcgcaatggctgttggcaagaacaagcgccttagcaaggg

taagaagggtatcaagaagcgtaccgtcgaccccttctcgcgcaaggacgagtactccgtgaaggccccctccaccttcc

agacccgcgatgtcggcaagaccctcgtcaaccgtaccagcggtctgaagaacgccaacgactctctcaagggccgtatc

ttcgaggtctccctcgctgacctgcagaacgatgaggaccactccttccgcaaggtcaagctccgcgtcgatgagatcca

gggcaagaactgcctgaccaacttccacggcctggacttcaccaccgacaagctgcgctccctcgtccgcaagtggcagt

ctctcattgaggccaacgtcaccgtgaagaccactgacgactacctcatccgtctgttcgccattgccttcaccaagcgt

cgccccaaccagattaagaagactacctatgctcgctcttcccaaatccgcgccatccgcaagaagat

>Hypothenemus_hampei_contig_10489|c0_g2_i1 40S ribosomal S1

ggtaagaagggtatcaagaagcgcactgttgaccccttctcccgcaaggatgagtactcagtgaaggctccctccacctt

ccagactcgcgatgtcggcaagacccttgtcaaccgcaccagcggtctcaagaacgccaacgactccctgaagggccgta

tcttcgaggtctccctcgccgatctgcagaacgatgaggaccactccttccgcaaggtcaagctccgcgtcgatgaggtt

cagggcaagaactgtctgaccaacttccacggtctggacttcaccaccgacaagctgcgctccctcgtccgcaagtggca

gtctctcattgaggccaacgtcaccgtgaagaccactgacgactacctcatccgtctgttcgccattgccttcaccaagc

gtcgccccaaccagattaagaagactacctatgctcgctcttcccaaatccgcgccatccgcaagaagat

>Hypothenemus_hampei_contig_10540|c0_g1_i1 ATP synthase subunit mitochondrial

tcgcagcgccgctctcaagatcgactgggccaaggtgtccacctccctcggcctccgcggtcaaaccgccgcctcgctgc

aatccttcaagaagcgcaacgatgacgcccgccgtaaggtccagcttctttccgagcagccccaggccgtcgacttctcg

cactaccgcaaggtcctgaagaaccaggccatcgtcgatgagatcgagaaccacttcaagaacttcaagcctgcctccta

cgacgtttcccgccagcttaaggccatcgatgctttcgaggcccaggctgtttccaacgctgagcagactaagggcaaga

ttgactctgagctggttaacctccagaagactctggagaacatcgagactgcccgtcctttcgaggacctcactgtcgac

gaggttgcttccgcccagcccgagatcgacgagaagacctcttcccttgtctccaagggcaagtggatgccacctggcta

caaggagcgcttcggcgatctgtccgctgtttaagcgtggaatctttcgattcttttatcccccgcttcatttcgctgca

agatgtatttgttcgatctcccgcatatggcttcgtacttgggtatggcttgtgccgcccgtccggtgtgatgaagcgag

gg

>Hypothenemus_hampei_contig_10540|c0_g2_i1 ATP synthase D

tcgcagcgccgctctcaagatcgactgggccaaggtgtccacctccctcggcctccgcggtcaaaccgccgcctcgctgc

aatccttcaagaagcgcaacgatgacgcccgccgcaaggtccagatcctgtccgagcagcctcaggccgttgacttcgcc

cactaccgcaaaaccctcaagaaccaggctatcgttgacgagatcgagaaccacttcaagaacttcaagcccgctagcta

cgatatctcccgccagctgaaggccattgatgctttcgaggcccaggccgtccagaacgctgagcagaccaagggcaagg

ttgaggccgagctggtcaacctccagaagacc

>Hypothenemus_hampei_contig_1055|c0_g1_i1 phenylalanyl-tRNA synthetase subunit beta

catccggtcggatgttctccacaaatgcgacctgattgaggacattgctattgcacacgggttcaacaacttctcgagac

agctgccgccatggctcaccgctgggtccgaagttcctctgaataggttttccgacaagctccgagcagagctcacaacc

atggggtttgacgagtcgctaacgctgacgcttctgtcgcgggaggagaacatgattgacggggagcaagcagtggttct

tatgaacccaaagtcttctgggtacgaggtctgcaggacatcgctgattcctgggttgatgaaggcggtggcgtcgaacc

tgcatgcaaagatcccattcagggtgtttgaggtctcggacgttgttctgctgtgtaaagagaaccagtgcggagcaagg

aacttgaggatgcttggcgccgtgtactgcggacatacaccatgtctcgaggaggtccaaggggctctgtctcttctcct

ggaaaagtgcgggatcatggactactcgtacaatccgcacgacgacctgtcgagataccttaagaaccagagcgccctgg

tggctgttaaaggcagcacgattggatcgataggcgtatgtaatccagaaatatgccgggcgctcaaggtcccgtatgca

gcatcgttcctagagatagatgtcgagaggctcttctcgatgtatacggaacacaaaagaagtaggcca

>Hypothenemus_hampei_contig_10556|c0_g1_i1 H2B1_DEBHA ame: Full=Histone

ggtatttcccaaaaggctatgtctatcatgaactctttcgttaacgatattttcgaaagaattgctggtgaagcttctaa

gttagctgcttacaacaagaagtccactatctcctcccgagagatccagacttccgtccgtctgatcctccccggtgagc

ttgctaagcacgctgtgtctgagggtaccaaggctgtcaccaaatactcttcgtctgccaaataagcgggctctgggttt

cttttatttctgcgggattgctctttattttattactgcctctgatcttgttttttcctcctgggttctgaggcggtcgg

ttatgggtgttttgcgggttttctaatgtgtcatgggatggtttcgattttcttttcccccgatgggcgacttttttctg

atggttactactcgatgcgcgcggtgtaacataactcaagtctggggccgggttgcatggtctaacctcccc

>Hypothenemus_hampei_contig_10556|c0_g1_i2 H2B1_DEBHA ame: Full=Histone

ggtatttcccaaaaggctatgtctatcatgaactctttcgttaacgatattttcgaaagaattgctggtgaagcttctaa

gttagctgcttacaacaagaagtccactatctcctcccgagagatccagacttccgtccgtctgatcctccccggtgagc

ttgctaagcacgctgtgtctgagggtaccaaggctgtcaccaaatactcttcgtctgccaaataagcgggctctgggttt

cttttatttctgcgggattgctctttattttattactgcctctgatcttgttttttcctcctgggttctgaggcggtcgg

ttatgggtgttttgcgggttttctaatgtgtcatgggatggtctcaatttctttttttcccgatgggcgactttttctga

tggttactgctccaattcgtgcggtgtaacataactcgggtctgggggctggttgcacggttgaacctc

>Hypothenemus_hampei_contig_10572|c0_g1_i1 Ribosomal L13e

gccgtggtttcaccctggctgagctgaaggaggctggcattcccaagaagctcgcccccaccgtcggtatctccgtcgac

caccgccgtgtcaactactccaaggagtctctggccgccaatgtcgctcgtctccaggactacaaggcccgtctgatcct

cttcccccgcaagagcggtcagttcaagaagctcgactcctcccccgaggaggtcaaggccgtcaaggcttccctggcca

acggtacccgtgacggtgtcgccacctacgttggtgctactttccccatcaccaacaccactcccgaggaggccatcact

gaggtcaagcgtgactccctccccaagggtgaggaggctgcttaccgcaagctccgtgactctcgtgctgaggcccgcca

ccgcggcaagcgtgaggctcgcgccaaggctaaggccgaggaggaggccaacgccaagaaataaatgtgttaatttttct

gctcgtgctgatgatgatgatcgggtcggtttttcggatcgaggatgaagagtgtattgtggcatggcgtgg

>Hypothenemus_hampei_contig_10575|c0_g1_i1 60S ribosomal L27

ttcgccgctcaccgggaccttcagaatgaagttcatgaaagtgggccgtgtggccatcatcacccgtggccgttacgccg

gtaagaaggtcgtcattgtccagcctcaggacactggctctaaggcgcaccccttcgcctacgccattgttgccggtatc

gagcgttaccccctcaaggtcacccgtcgcatgggcaagaagaccgttgagaagcgcagcaaggtcaagcctttcatcaa

ggtcgtcaactacaaccacttgatgcccactcgctacactctcgagctcgagggtctcaagggtgccgtttccgccgaca

ccttcaaggaggtctcccagcgtgaggacgccaagaagaccgtcaagaaggctcttgaggaccgctacaccagcggcaag

aaccgttggttcttcactcctctgcgtttctaaacggggtatttaagggcgttatgtcgtttgagcgcgcaaggtcttag

atgcggcaaagggaataaaaaggcatcgag

>Hypothenemus_hampei_contig_10575|c0_g2_i1 60S ribosomal L27e

gccaaccgggaccttcacaatgaagttcatgaaagtcggccgtgtggccatcatcacccgtggccgttacgccggtaaga

aggtcgtcattgtccagcctcaggacactggctccaaggcccaccccttcccttacgccattgtcgccggtatcgagcgt

taccccctgaaggtcacccgccgcatgggtaagaagcttgtcgaccgccgctcccgcatcaagcccttcatcaaggtcgt

caactacaaccacctgatgcccacccgttacactctggagcttgagggtcttaagggtgttgtttcccaggacaccttca

aggaggtctcccagcgcgaggacgccaagaagaccatcaagaaggctctggaggaccgctacacttccggaaagaaccgc

tggttcttcactcctctgcgcttctaaacggttttaatcgggcgtttgtcgttttgaacgcggttcaggtgaaggcgagg

gggaaaaaagcatcgagcacattcggtccctgatttgggataggaaggcatataccatagctggaaacagaatcgggaat

gtatcggtgaactccagttttga

>Hypothenemus_hampei_contig_10604|c0_g1_i1 signal peptidase

ataccacccgggcagcatctggagcgagacaaacgagaagtggaacgaggcagacggggggtggagggccgttggggttg

ctccaagcaggttcgtcacggccggcgagcgcagcgaggagaaggaggccccggtgagctttgcagccatgctccccagg

aaatggtactctgtggtcgagtttcccagcggaaggctcgaggtcgggcaccacctgagcatgctcgaggtgggcacata

tgtgattctggaggcagaccgcggcgaggactgtggcaggattgtctggaacatgagcgagagcgagttcgagggctctg

tgccgcaggggcgcgacgcaaagagcgagttcgagccgaagaagatcctgcggagggcgtctgcagacgacctcaacaag

ctgaggcagaggaaggagatagaggcagcgtctctgcagcgatgcagggagcttgttgcggccaggaggctgagcatgga

gatcctgagctgcgagtaccagtgggacatgaggaagataacgttctacttcaagagcgacaagcggatagacttcaggg

acctgctgaaggagctgttcaagctgttcaaggtgaggatctggatgtgtgccgagaggaggaccaataacgacgtgatt

aaaaagatcgg

>Hypothenemus_hampei_contig_10609|c0_g1_i1 hypothetical protein CANTEDRAFT_112491

gcaaatttcatcagaactgcttcttccgttccagcagtaattagttctttggcccttcaagtcggtacctacaaagatga

ttcttataccaccatgttagatgatgatggaatcgatgtcgactccttggaagactttgctaccgaattaccatggtact

catcaagactcttaaaagctaaaggaggatccgaaactgattctgattctgaatccggttcagactccaaaacttcttca

agtgaatcttcttctgcatctagctctgacagtggaaatggtgctggtagtattgttgctccagctggtgctatgttagg

tgctattgccgttgcattattgtaattttgaatag

>Hypothenemus_hampei_contig_10624|c0_g1_i1 hypothetical protein ECU11_1890

ctggaggcttaggacgttgaaaagcgaaccccatagaatagagaacatgacgaaaacgatcaaggcaagcacggtgaatg

ggatgctgattgcgctgattgttgttcttggacttgtaattatatatctctcggaatggtacaagatattctcgctgttt

tccgaagacgcaaggagcatcgttctgatgaacaaggaggacattgagaggagtgtatatggggacgggatgaagatgaa

gatccagaagaagaagctgtctggggaactgtggaaggactacgagcggctgaggaccaaggcgattatggttttttctg

atggcggatatgtcaagatcttcgacattggatctggagacgtgaagcagtacaagcacgacgagtatttggactacttg

catggaatcggcagctacctgctccctttgaattcgctgaagtacgacgtgcggaactttctgagggatactcttgagga

caaggaacgcgacgaggttgaggatgcgcccatggtgtatggatgcttgatcatcccaaacacggacaaggggcttaagt

tcatgtcgaggtcgaggtcgatggatgtcaattgtaactggagcgacattagttatatctgcctcaaggaaggaagtttg

agcgaaaaataaa

>Hypothenemus_hampei_contig_10634|c0_g1_i1 hypothetical protein ECU08_0480

gctctgatcccccttgccctggccatgctctggacaaatgtaaatgtcgtccagttgcttgttggcatcaggtcgccaaa

gcctccaaaggccagggtcttggctgccaacgcctcgtccttcctcgacgtgtttgtcctgaaatacctcacggggatcc

gcaacttctactatgttacggattctgggttcgttgacgtgcacacaggccagtttcatcagagggtggccgagccttgc

gtcctgtttcccgaaggctgtcggaccaacaaccgcgccattctccagttcactagggatgtcagggtcgaccatgtctg

ttgtctgaggtactctggggaatgtataaacatgtacggcgggctcctgggcttcgtcccaaggttccttgcatccaggt

gctcagtggatgtgaggttcaggaaaagcactgacccacacgatatttgtgagcttggaggccttccacaggtgagatgg

acatccaaagacag

>Hypothenemus_hampei_contig_10638|c0_g1_i1 conserved hypothetical protein

ctctactgtatcttcatcaaaaacctcccaatcaacaacaccacaaccaacaaaccaaaatgtctcctcaacaccagcgc

tcctcctccccccaccagggcggctctctctccgacatggccccaacaggaacctccatccccaacgacgccggcatcca

aagaacgatcccctcggtgccgcgccccgaccaaagatccgagaacgcgcagttcaacaaccaaggcatcgccgagccca

cgaccgcctttgccgctgacaacgccgccagcatgccccgtggccctggcgatctgggcgagacgggcgaggtgttgact

ggcacggggaattctttcccggctagtggtgaggcgaagaggaatgagaactcggctgggtatcctggggggaagtacta

ggccctttttgtttgagggataaagtgttatgatatgatttttgggatgttatggatgagtgacttgtgtggttactctt

tttgttctcttactattactgccagtactactctcactactattgcatttctaaagcccatcgataccacgaccgaaatc

ttcgtgaatgtcatgtataatacgtactgaatatgcgatgttgtcaaaatgttt

>Hypothenemus_hampei_contig_10653|c0_g1_i1 Golgi-to-ER retrieval

agcagatctatctggatagacttgcgccgagaccagacatacgatggggagtgacgggggctctgttcgtgttctacgtc

atacggatatggacgactggggcgttctacttgatcacatattgcctggggatctatctgctgcatgcgctgatcctgtt

tctaacgccgaagggagagaccattccggatccgtttgagaacatcgaggaggacgactacattccggaggccatagaca

acgagttcaagccgttcatcaggaacctgccggagtttgacttctggatgtttgtgacgaagatccttgggatggcgctt

gtggggacgtgcttcggcaagctggacattcctgtgtacacgccgatccttgtgatttattttattttcatggttgggta

cactgcaaagcggctgattgcacacatgaagaagtacaactacaacccgtttgtgc

>Hypothenemus_hampei_contig_10683|c0_g1_i1 Protein of unknown function DUF1687, fungi

cacaaccgataacccaacaacagatcagcttcgttctatcctggattacatctcgcctacttccggaacaggcggtctgg

gaaataaggagacatacgccgtttccgaactcattcgtgatgcgaaggatgccgaggatgctattaagagatttaaggct

gatggcaatagctttgttaagcctgttactgttgattgggtgaatggtcgtgctgttattggtgatggcgagtctgagat

tcttcgtatggttcgtcaattgcccgagaactaaagcacggcatagcttcattgtcggggcgttgaaatgaatacactgt

atggcattgtacaactattgc

>Hypothenemus_hampei_contig_10707|c0_g1_i1 glutamyl tRNA synthetase

cttgaaaacaggccgcggatccacgacttctcgaggctcaacttcgagaacacggttctcagcaagaggaagctcaagta

ctatgtcgacaacgggtttgtgtgtggatgggacgacccgcggctggcaactgttgcggggatccggaggcttgggatga

gcatggaggctctgagagagtacattctgatgcagggcgtgtcgcagaagacatgcacgatttcctgggacaaggtgtgg

gcccttaacaagaagaggatcgacccgggtgcgcccaggtacttctgcgtgcggcagaaggacgccgtcgaggtgacgat

tgataacacctcggagtatgtgatggatgtgcccaggcacaagaagaacggggctcttgggacaaaggccgtgctgtatt

ccgatcggatactgctctcgcaggaggatgcacaggctctgcaggacggcgaggagttcacgctgatgaactggggaaat

ggcattgtcaagagcagggcggtttctggcggcgtggtggtcgggatggaggtggccctgaacccagggggggacttcaa

gctgacgaagaacaagatttcctgggtctcgaggcgaggatctgttgctgtcgagcttgcggagtacgggagcttgatga

acgacgaggacacagaggacctggcgcggaagttcaacagggactctgtctcgaaggagtactggtatgcagagtctggg

atcatggatgtgcgggagggcgaggtgatacagtttgaaagatgcgggttttactactgcgacgggtttcttgtgttcaa

cctggttccctttacaaagcaaaagagaacggccaattaaacttgttccataccttgctgggcggtccttgtgcttggag

attggcccatggggctccggcgtcctccgggctctgcctcggcctctccctgtgatttagtatctgcgacgggtactagt

agatccggggcctgctatgccgtaga

>Hypothenemus_hampei_contig_10728|c0_g1_i1 unnamed protein product

ctttctttgttagttgtaatgagcaacctcaccagacgactggaagtacgacaaggcaagcgtgcacacgatcaacagca

gacagcatacgcaagagaacaacaagaacgagcagaccgacaagaacgacaaacagggcacaaacaagcatacgacgtgc

cagataacgcgagacgaattgggagtccaaccgaacaaccgcattactatacaacgtccgagagtgactcagtacagtgg

actccagcgggatttgagtcagcatttcccgagacacgaaccgattacccccagcaagccccattagcagacatgagtaa

agcacccctattttacggaaaaccagggcaatacgactcagtaaagacgtggtgtgacatcacttttatcaccaacgacg

agctttcacaagacaaacaaaaacaagccgctttctt

>Hypothenemus_hampei_contig_10738|c0_g1_i1 minichromosome maintenance

tgtcaaggtttgatctatactttgtgctgattgacgatgcgaatccggagaacgacaggaatgttgcaacacatgtgctg

aacaaccacgccttgattaccgactctggggcgctgggcagctatttcacgaaggagcaggtgatgctgtatctaaggta

tgctcgtggaaagacgccccggatgaccgaggaggcaaaggaggtgcttatcaagaagtacattgggataaggcaggaca

gcctcgtccacagcaacaactatatgatgacggttcggcacctcgaaagcctgatcaggctcagcgaggcacttgcaaag

atccacgacaatgaggttgtgacgaaggagtatgtcgaggaggcacacaggctggtcaagagcagcatcatcgaggtcaa

gggggaggacatagagatagttccgaggacagatgacgactgaggggtcatgatcaacagaaatgactacatacggatca

caaactctttcatctatctcataaagacgcgggagccgatggagaggcacgagctgatagaggcgtttctctccgagaac

gagtcatcgatcgagagcgaaagggctcttgttgaggagcagtccaaggcagagaacgtgctgtcgtttctgataggcaa

ggagggcatcctgtttgtcagtgagggaaggatccacatccatcccagctacgatgtatgaagtaaaacccttcgatttc

caaaaaaaagatcg

>Hypothenemus_hampei_contig_10746|c0_g1_i1 major facilitator superfamily

tacgccgaaggtcctgtactttcttgtgaatcttcagtactactcccttcatcaattccggggggtttttgtgaagaaga

aattcggtgccaccgacggtgaccttgcaaactacatgggccctatgctcggagccatcttcttcacaaacatctttatt

ggcacgatgaacgacaagtttgggaagtctcacctgttcatagtgggcgctctccttctcacatgcgcacttctccagat

gttctacgtcgatttctgcatgggcctctttccaggcatgttctggatcaaccttcttctctacctgaccttcaacaacg

gaatcccccctctgcttgacaaagccgtgctggactatctgagcgggattccagaggcgggtgcccgtgcatatggaagg

cagaagctgtggggcacagcagggtacgggctctcctgcaaggtcatagaaaagtgcatcaagttcggagacgacttcaa

gttcgaaaacctaaggtactactccctgg

>Hypothenemus_hampei_contig_1075|c0_g1_i1 hypothetical protein ECU04_1420

aagagctcctgtccgagaagctactccagccctcggaggttcgggatgtaaacatcgtcatcagggattccatgatcagc

agccttctggcaaaggatgcgtacacacgcttttccgtcaagaagaagggcgattctctgtacgtgtgctttgaagacgt

ttacatcagagaaatcaagtactatgactatctgagtagatatggagaggaggtcatcaggtccctaaagtcctctgagg

tcaggatatcctcgggcccggcatctaccgttgttgtgtattcgtcaacactgttccgcagctcggccggggtccagagg

ctgttcagttgaatcgatgtcgaaaagtcgattgagacagtcgtgaatatcgaagacgtggcagtggtggccagctccgg

ggcgctggggagggaactgaaggctcttgccggaattagcttcgaagagggcgaatacatcttcaacgatcgaaaagtat

acatagtagatccggcgggacaaaaactttcatttggcggggttgtgtttaccgacttcatggacgcccaggagccggca

agcacgtgatttatttgggctttgttgtgactcgaatggaaaacaaagtgaaactggtgtcttcagatggtaaagagttc

cttctcgaatacgacattgctatccaaagccaaacgctgcagtcgttctttggacgcccaacaatgttcatggagtccat

ttcgagagaggtcaggcttccaataaatgcagtgcatctgaagagagttgtcgagtttcttgaattcaagcatgccttgg

atccaagcaaggagccggaggagttcaagatcgaggactcagaggccttggagctcctggacattgctgcatatctcaaa

atctagaggtctggccaagcgctttatttcttgctacacggcatccatcagcggtctccggcttccgaaagagaccacct

atctctaagggatggccttgtgtcccccg

>Hypothenemus_hampei_contig_10752|c0_g1_i1 hypothetical protein EHEL_060530

cccgagcaacatgaacctagctgtcttcgttccgttgatctccatggccctctgtgcagccgacgaaaagcctgccgtcc

atgcagacacccggggaagccttgcacttgcaaaccccatatacgccgagaccgcgtcgtccccgatcacaaatgccgcg

tatgcagtggggctgctgctggccatagcattcatcaacatcccgaagaagtcattcgagtcgctccagtactgcaccgt

ctcgctcatcggagtgtatttcggcaccacgcacatcctgcagtctgcagggtgggggctcgactacctcatccccgtcg

cagggcttggcgctgtcgttgctgccgtcctgtcgtacaacaaggagttccgcgacctgttcttctctgcaattgctgcg

tacggaacaacgtacttcatcgtcctgatctcgaggatggagaacttcatctatgcagcaatcatcggcgccatcctctt

ctttgcatacctcttcgtcgggaggatgaaggaggactcgcggctgctggttgcaatggcaaaggcagacgtcacaagcc

tgggcttcgtcaccttcgtgaacgtctggggcgtcctcgacctgtttggcgggatgcacggcctcaatgcaaccgagggg

ctggtgtctgggttcctgatcggaggcctgctcatcctcgtcgtcttcaccgttgttttcgtggcaaactacttccagga

gtggaccgaggagaggatgaacagcatcaaggggtagtgggccacggcctgcccgcaggcgcctttgagcgagcggaaat

aaaccctgttctgtactgtgtatttcgaccctgcagcccaagccccgatgctggagccgagggtcgagcg

>Hypothenemus_hampei_contig_10803|c0_g1_i1 ribosomal S6e

aaattttttccggggcctacccttacggatgaaggtaagtgacttggagtagcgctaactttagttgaacatagcatacc

cgacgaacggtacgcagaggatgtttgaggttgataggagggttgagacgaagctgtacgacaagatgataggagaccag

tttgacggggggattctgggagcagactttgaggggacgatcatggagatcaccgggggagacgaccaccagggcttccc

gatggtcaaggggtatctgacgaagaagaggatgaggcctttgctgtcgaagggcgatgcaggatacaggtgcaggcgca

agggggtgaggtgccgtaagagcgtccgagggtcgattgtatctgaggagacgtctgtgctgaacctgataatcctgcga

tcgggcgagaaggagatagacggcctcacaactgtcgtcaacgacgtatcccatctcccaaggacggataagaagctcag

gaagatgtttgatgttccggagtccgagacgaaccccgtgaggtacatcaggaagattctgaaggccgagtgcgaggatc

caaagaaggcacccaagatcaagcacaacggaaagagaatgaagaaggagcaggagaggaaggaaagcgaaacgaagatc

agagcagagaggaagaggattctggaggaggagaggaaggcttatcttgagaagtacttcaataaagcct

>Hypothenemus_hampei_contig_10813|c0_g1_i1 40S ribosomal S2

ccgctatcaaggctgccattgttatcgccaaattatctatcatcccaatcagaagaggttactggggttctaacttaggt

gctcctcactctttaccaactaaggttaccggtaaatgtggttccgttttagttagattaatcccagccccaagaggtaa

aggtattgtcgcttccccagttgttagaaaattaatgcaattagctggtgttgaagatgtttatactacttcttctggtt

ctactagaactaccgaaaacaccttaaaagctgctttcgttgccattggtaacacttacggttacttgactccaaactta

tgggctgctcaacctttagctccatctccattagatgtttacgctgaagaagctgctgctggtaaaagaagatactaagt

tatttaactaaatatttttggtaaatacttgtaatctatataaatctt

>Hypothenemus_hampei_contig_10846|c0_g1_i1 disulfide isomerase

cgacgtgctgagggccatgaagagtatcttctcatatgttcccgaggatgagaagttcgacggagagggtgtcgaggtca

agcacgaaggaagaatgtttgtagttgagaaggccgaggacatcgaaggcgcagtggatttgctgaggaagtacccagcc

cctacggaggagaccatccaggccatgaggtctgaagggagtgaggcggagaaaggccgtggaaacttcgtggtgtactt

cttgggcaggtcggactacaagacggacatacgtgacagtaagactgcaagcatgtttgtgtctacggatctgtcccttg

catctgagctgggtattcctgcaccgggaatatacggatacaacggaggcgacgggctgtcatacagctccgagctcagt

ggcgaacgcgccgagaagattgtctctctggcaagtctcccgatgttttggcttcacctcgatggagaatgtctccatgt

acagatcgctaagagcaaccatcttctacatcttctttgagcccaagagcagtctggagatcctgaaggagtatcccggg

actctggacgacttcaggtacgatgcaagggtgattctgattccgaatattgatgacacgcttgggatcgaggactacgg

gctgacgaggaaggaccttccgggatgcgtgtccatcaggagcgatggtggtaagtacgttctaaggagtgtgacaaagg

acaccatggccgggtttgtcagggatgtgctggagaagagggccgagatcttctacaagtctcaggaggagcccaaggat

aatgcaaccagaggcgtcaaggtgatcaccaggagcaactcaaagctgtatatcgacgatgcggacaaggacaggctgat

tgtgtttgggacggagaggtgtcctcactgcatccggatcaagcctgtgttggagaagctgggggagattgcacgagcta

acgcagacgacaagctggttgttgggtactgcgacgtcgatatgaatgacatgagcgactttgagatccggtttgttcca

accatcttactgttcaaggcagggggcaaggagagtgtccagcactctggcggggaaagaacacttcccaacctggtctc

gttcatccgcgagtccggggggctgcataccgacttgtctggatttgtgccttcggagctgcaggagaggagatttgagg

ctggcgacgacgtcaggcccgagctctaaggaattaaatgtaaa

>Hypothenemus_hampei_contig_10846|c0_g2_i1 disulfide isomerase

cgacgtgctgagggccatgaagagtatcttctcatatgttcccgaggatgagaagttcgacggagagggtgtcgaggtca

agcacgaaggaagaatgtttgtagttgagaaggccgaggacatcgaaggcgcagtggatttgctgaggaagtacccagcc

cctacggaggagaccatccaggccatgaggtctgaagggagtgaggcggagaaaggccgtggaaacttcgtggtgtactt

cttgggcaggtcggactacaagacggacatacgtgacagtaagactgcaagcatgtttgtgtctacggatctgtcccttg

catctgagctgggtattcctgcaccgggaatatacggatacaacggaggcgacgggctgtcatacagctccgagctcagt

ggcgaacgcgccgagaagattgtctctctggcaagtctcccgatgtttggcttcacctcgatggagaatgtctccatgta

cagatcgctaagagcaaccatcttctacatcttctttgagcccaagagcagtctggagatcctgaaggagtatcccggga

ctctggacgacttcaggtacgatgcaagggtgattctgattccgaatattgatgacacgcttgggatcgaggactacggg

ctgacgaggaaggaccttccgggatgcgtgtccatcaggagcgatggtggtaagtacgttctaaggagtgtgacaaagga

caccatggccgggtttgtcagggatgtgctggagaagagggccgagatcttctacaagtctcaggaggagcccaaggata

atgcaaccagaggcgtcaaggtgatcaccaggagcaactcaaagctgtatatcgacgatgcggacaaggacaggctgatt

gtgtttgggacggagaggtgtcctcactgcatccggatcaagcctgtgttggagaagctgggggagattgcacgagctaa

cgcagacgacaagctggttgttgggtactgcgacgtcgatatgaatgacatgagcgactttgagatccggtttgttccaa

ccatcttactgttcaaggcagggggcaaggagagtgtccagcactctggcggggaaagaacacttcccaacctggtctcg

ttcatccgcgagtccggggggctgcataccgacttgtctggatttgtgccttcggagctgcaggagaggagatttgaggc

tggcgacgacgtcaggcccgagctctaaggaattaaatgtaaa

>Hypothenemus_hampei_contig_10988|c0_g1_i1 60S ribosomal L12

tgtcggtggtgaaattggtgcttcatccgctttagccccaaagattggtcctttaggtttatcaccaaagaaagttggtg

aagatattgccaaagctactaaagatttcaaaggtatcaaggttactgtccaattgaaaattcaaaacagacaagctgcc

gcctctgttgttccatctgcttcttctttggtcatcaccgctttgaaggaagccccaagagacagaaaaaaggttaagaa

tgtcaagcactctggtaacatcccattagaacaaatctatgaaattgccagacaaatgcaagaaaaatctttcggtaaga

acttggcttctgttaccaaggaaattttgggaactgctcaatccgttggatgtcgtgttgaaggtactaaccctcatgac

attattgaagctatcaataacaacgaaattgaagttccagaaaactaaaccttgtaagaatagattgcaattt

>Hypothenemus_hampei_contig_10988|c0_g2_i1 60S ribosomal L12

tgtcggtggtgaaattggtgcttcatccgctttagccccaaagattggtcctttaggtttatcaccaaagaaagttggtg

aagatattgccaaagctactaaagatttcaaaggtattaaagttaccgttcaattaagaatccaaaacagacaagccact

gcttctgttgtcccatctgcctcatctttagtcattactgccttaaaagaagcaccaagagacagaaagaaggaaaaaaa

tgttaaacactctggtaacattccattagaagaaatctttgaaatcgccagacaaatgcaacacaaatctttcggtaaaa

acttagcttccgttaccaaagaaatcttaggtactgctcaatccgttggttgtcgtgttaacttcaaaaaccctcatgac

attattgaagccatcaacgatggtgaaattg

>Hypothenemus_hampei_contig_11022|c0_g1_i2 ankyrin repeat

aggctgttgaatctggtaacttgaagatggttgaattgctgctgtctaaaggtgccaatgttgatgctgcgaacaaggat

gacacgacagttttgcacaaggctgtcgaagctggtaactttgagatgattgaatttctgatgtttagaggtgccaacat

taatgctacaaacaaatatggtaatgcagttttacatagggctgccgaatcgcgtaacctggagatgtttgaatttctgt

cttgcacaaggccgccgaatcaggtaacttagagatggtcgaatttctggtgactagaggtgccgacgttgatgctacga

ataaacatggcaatacagtcatgcacaaggctgccgaatctggtaaatgggagatggttaaatttctagtgatacaaggt

gccaatgttcatgctaccaacaaagatggcatgacagttttgcacaaggctgtcgaatctgataacgtgatgatggttga

atttctggtgattcaaggtgcc

>Hypothenemus_hampei_contig_11022|c0_g1_i3 ankyrin repeat

aggctgttgaatctggtaacttgaagatggttgaattgctgctgtctaaaggtgccaatgttgatgctgcgaacaaggat

gacacgacagttttgcacaaggctgtcgaagctggtaactttgagatgattgaatttctgatgtttagaggtgccaacat

taatgctacaaacaaatatggtaatgcagttttacatagggctgccgaatcgcgtaacctggagatgtttgaatttctgg

tgagtagaggtgccaacgttaatgctccaaataatcatggcaatacagtcttgcacaaggccgccgaatcaggtaactta

gagatggtcgaatttctggtgactagaggtgccgacgttgatgctacgaataaacatggcaatacagtcatgcacaaggc

tgccgaatctggtaaatgggagatggttaaatttctagtgatacaaggtgccaatgttcatgctaccaacaaagatggca

tgacagttttgcacaaggctgtcgaatctgataacgtgatgatggttgaatttctggtgattcaaggtgcc

>Hypothenemus_hampei_contig_11026|c0_g1_i1 Heat shock 70 family

tcacaacaccgtcttcgacgccaagcgtctcatcggtcgtcgttttcaggacgccgaggtccaggctgatatgaagcact

ggcctttcaaggtcattgagaaggctaccaagcccgtcattgaggttgaattcaagggtgagaacaagcagttcactccg

gaggagatttccgctatgatcctggtcaagatgcgtgagaccgctgaggcctacctcggtggcaccgtcaacaacgccgt

catcactgtccccgcctacttcaacgactctcagcgtcaggccaccaaggactctggtctgatcgccggtctgaacgtcc

tccgtatcatcaac

>Hypothenemus_hampei_contig_11048|c0_g1_i1 Glucose-repressible Grg1

atcactacaaacacatattcacaatggagaccgtcaagcaagctgccaactacgtcgccgagagcgtccagggtgctgga

gctggtaccgagaaggaggccaacaagaacaccgcaaagaactccgatgccaacgttagcactcgtgcccaggccgctaa

ggatgctgccgtcaacaaggtcgacgagaagaagcacgacactaaggctgatgtccacaaggaggccgcgaagcagtaaa

atgctgtgaacaatacttaaataatatccttaacggcgatatacgatatcacatggcaataatattatgatcacattatt

cacaaacttttatttcaaatttccaagcacttcgaataagattttacagatcg

>Hypothenemus_hampei_contig_11048|c0_g2_i1 Glucose-repressible Grg1

atcactacaaacacatattcacaatggagaccgtcaagcaagctgccaactacgtcgccgagagcgtccagggtactgga

gctggtgccgagaaggaggccaacaagaacaccgcaaagaactccgatgccaacgttagcactcgtgcccaggccgctaa

ggatgctgccgtcaacaaggtcgacgagaagaagcacgacactaaggctgatgtccacaaggaggccgcgaagcagtaaa

atgctgtgaacaatacttaaataatatccttaacggcgatatacgatatcacatggcaataatattatgatcacattatt

cacaaacttttatttcaaatttccaagcacttcgaataagattttacagatcg

>Hypothenemus_hampei_contig_11048|c0_g3_i1 Glucose-repressible Grg1

atcactacaaacacatattcacaatggagaccgtcaagcaagctgccaactacgtcgccgagagcgtccagggtgctgga

gctggtgccgagaaggaggccaacaagaacaccgcaaagaactccgatgccaacgttagcactcgtgcccaggccgctaa

ggatgctgccgtcaacaaggtcgacgagaagaagcacgacactaaggctgatgtccacaaggaggccgcgaagcagtaaa

atgctgtgaacaatacttaaataatatccttaacggcgatatacgatatcacatggcaataatattatgatcacattatt

cacaaacttttatttcaaatttccaagcacttcgaataagattttacagatcg

>Hypothenemus_hampei_contig_11075|c0_g1_i1 hypothetical protein M970_090110

tgagaagaggggggcggtgcacaaggatttcgtccgcaagatgtgcgagctggaggatgagctggatcttgcctccctcg

aggaggagaacggggcacccgacgggaacaagagaaggagaaggtcgacgacgctagacgtggacgatataatgtgcact

cctgttgaagaggcaggaaaggatcttcaagagatactgggtggcgaggcacctgatgtttttgttgaaaacagcactct

taatgtgaagggaaagacgttctccaaaggagacaaggttagaatcacaataaacaaagaagaggttgtgggaacgatcc

tctccgtaggggagggggacgttgttctgaggacaaaggatcaccgaaggctgaaggtcttgctagaggacatgcggtcg

accaggtcatctatagctgcgaccgatgaagacagcacttagtccttgagaacagcggaactgtagaatgaaaagtgaat

aaagtagat

>Hypothenemus_hampei_contig_11083|c0_g1_i1 hypothetical protein EROM_031470

cttgaccctgatagacaatatcgagaagagctacgaccccacgtccctcaactacaagttcaagtatgtgttctataaca

aggtagacggcccgttcatgcgtccccccgactttccggagacgctgtggaatatctccctgacatctgacccaaccatg

atgcctgtgctgctgaaaggcgacgagatcgagcacaggaaatctctccagatggaggtgtgcaagaagatcaacgactc

gtacgacttcctgcggaagaagataggggggctacggatgaggtctgagaagctcaagtcgaggatagacgggtgtgtgc

agatttacaggaaggtcttcagaggcgtctacaacaagctaaagaaagaggggggaaggtgcaccttgctcgaccacata

tacagggcatacattcccctggacaagagaaacaagctctgtgttgcagaaagaaagggcgaagttattgatttcctgat

ggacctgaaaacaacgggggagaaaatcctcaaggatgccgaaaatgctctgcaggaacgccagaagcagactgttatac

tgaacgagctggataaggtttagtctttgagctgtggtcgatgaattaaactaaa

>Hypothenemus_hampei_contig_11090|c0_g1_i1 Psp1

tttgagagcaagaggctcgacgttgcgtttgtggacgtcaacatcggtgcgcggaagaactcgtacgtgatcctggaggc

cgaccggggcgaggactgtggggttgtggtcggggtgaccacgaaggacaagctcgagaggctggtgaagcggcacgagg

agatggcgagcgaggtgcagccaaagaggatatacaggctggcgacgctgcttgacctggaggcgctggagcgcaagaga

agcatgcaggcaagggcgctggagttctgcagggagcgggccatggcaggggggctggagatggaggttgttggatgcga

gtaccagtgggacctcaacaagatcacgttctactttgccagcgaggagagggtggacttccgcgagcttgtgaaggagc

tctacagggtgtacaagacaaggatatggatgtgtgccatcgagaagtcgaagaacaagtacctgaaggagcttgtggac

tcgcaaggaat

>Hypothenemus_hampei_contig_11091|c0_g1_i1 60S ribosomal L10

ctctccggagggttgtcgggaaagctcttgtatcggcagggtatgtgtttgcgtcccgggatgcgctggaggtgttcagc

gatgttctgcagtggaaggcaattgagatcatacgaagagaggctgtggcaagggaggagccgggtagccagcaggagtt

ttaagtcagcaaattttttagccctcaaatggggagaaggcctggaaggtgctaccgatatctatcgaagaaggcgtacc

cgaagtcgaggttcaacaggggggtccctgactcgaagatccagatcttcgaccttgggcggcgtaaggcaggggttctt

gagctgccgctgctggtgaactgtgtgtctcacgagagggagcacctgtctgcagaggcgcttgaggcggcgcgtatctg

tgcaaacaagtacatggtgaagcatgtgggcaaggacaacttccacctgcgcgtgcgtgtgtatccgttccatgtgctca

ggatcaacaagatgttgagctgtgcaggggcggatagacttcagacggggatgagggggtcgtttgggaagtcgtatggg

cgggcagcaagggttgtgttcaaccagccgatcctcagcatacggacgaaggaggcgttcaaggatgcagcgatggaggc

gctgaggagggcaaagaacaagttcccggggcaccagaagatccaggtgagctcgaagtttgggttcacgaacctgttcc

acgacgagttcaacaagctgcactcggagggccggattgtgctgaggggagggtcgttctctgtggtccgagagaagggg

agtgtcgacgcgttccttcagaggcttgaggaggcagcggataattaaagatcg

>Hypothenemus_hampei_contig_11092|c0_g1_i1 hypothetical protein ECU01_1100

aatagaccatgaatccgggatgggagagcagaaaaacgatgtgatgttgagcattcagagagagtttgaggaattgaagg

aggcccataggaagacgctgaaggctcgtggaaagcttataagggccatggagaaggaggtgtctgagcttaggcgggca

atggaggacaagaggaaagcagagatagaagatctccgccggcagtacgaggaggagtttctggaaagaaagagggtgta

caaagaggcgtggaagaagaaggtcctggagtacaagagaaagctggacgaggtatatagatcaaagatcataagtttca

ggaagaagtgtgaggaggctgttaggaaagcgcgggagtcatggggtcggaagaggccatgagtctgaggacacgtgtat

ggacaaaggtgtttattaaactctat

>Hypothenemus_hampei_contig_11093|c0_g1_i1 cleavage and polyadenylation specific factor

ccaagccgatcaggctgcatctgataagctcgagcgagccgctccgcaacgtgacgtcgacagagcttcttgctgcaggc

acggagctgtcgatgctgtgctgcgactccaccggcactgtccatgcatatacgtactctccaaacaacatcataagcat

ggacggggcaaagcttgtcaagcgagcagagatgaagacaggcctagggaggctggcgtcgagcagcacggggctcagga

aaggcagcgtcatgctgtactcgaggacgaacatgctggtgcacgtgagcggggtcgacgactcgaagtacctccggctc

atggggatacaggcatcgatcatggcacatctgccgacgctctttgggctgaaccggagggactacctggactctgacat

ccacctgcacgggctgtcgctcaagggccccgttgtcctccatgtcctgaacatgtttgggtacttcgacctggagacgc

aggatgccatctcgtcgtctgcagcaatgggcaggggagaggtgtcgggggtcattgcgtctctgaacctgttctgatac

gattaaagcttgttgccacagacgg

>Hypothenemus_hampei_contig_1110|c0_g1_i1 dihydrofolate synthase

gatcaaagaaagctgggccgaagatgacgttaggacggagaggtgtttctactttgtagggtttaggactcctgagtcaa

tgaagtgggtgaagtgtacgccgcctcaaaggctcggggcgatttttgccgaggttatgaagaaggagacaaataagcaa

gtctctgttgtgaaagacatgaacctagaggaagcgcttgtggactgcatcaagaacagagaagggaagattgtgattat

tatttgcggctccctttatttgatggccgaattttaccgtctgaaagggggtccggcaagtcctacttattgccgctaac

cagctcgtgaagctttgca

>Hypothenemus_hampei_contig_11112|c0_g1_i1 hypothetical protein ECU01_0440

cttgatgttctgtctgccgtggatgggtgtgtggacgctggacgttcagtctggcggggaggccgtgaggggcaggatgc

gtgactcttgcgagtctgcggcacgcctggggcgtgagtatcttgggaaggcgtgcgagctgtttgagaaggggcggagc

cttgtgtacacgcgggtgcagagcatggtgtctgggatcaggaggagatgccaccgcagcaaggagatagtggggcccgt

ggacggagagaggtctggggacgaggaggcgtttgacctgtcgcccgaggacatcgagaggctgattgaggagatcaaga

ggaagcttgccgggtacatggatatggagacgccgggcgaggagaagggcggcgaggagatgaaggaggagagctctgag

aagaagggggagaaggaggaagagaagaaggaggagctttgagttggtcctgctgtttgtttacggtggccggtaaataa

accttaa

>Hypothenemus_hampei_contig_11115|c0_g1_i1 hypothetical protein ECU08_1210

ggcagcacgggccgaagaaggcgcagaggtcaagaaagaggagaagcaggaagggaggtacaacgtcgacttccaggagc

tcaacgaggagctgtctggcattgacctgaaggaggaggagcctcgggtgcctgaggccgacgagccaagctgtgccggc

gacgtctgtatagaggaagggggtgtgtccggggagaagatcaacgtgcagaggataacaaacgaggacaggttccggat

tgacgagggggccggggagcccaaggacggggcgaggcctcggacggcatatccgatgactccaaatgtgctgacggctg

ttgtgccgcagacgccagggaggagggtgctccatgaggagggtagcaacctgg

>Hypothenemus_hampei_contig_11146|c0_g1_i1 Rodlet peptide (Fragment)

ctctttctgaagtacagtcttctctctttcttataaacctaaactccgagaacacctttcgttcaaacccaaacccacag

acacaatgcagttcactctctccactgtcctggccctcgctgccaccgttgttgctctccctccctccggcccctctgct

ggtggtgttggtaacggcaatggtgtcggaaacaagggcaacaccaacgtccgcttccctgtccctgatgacatgaccgt

caagcaggcccaggccaagtgcggtgaccaggctcagctctcttgctgcaacaaggccacctacgctggtgacaccaccg

atgtcaactctggcatgctcggcggcaccctcagcaacctgatcggtgctggctccggctctgacggtcttggtctcttc

gaccagtgctctaagctggacctccagatccccatcctcattgccgtccccatccaggatctggtcaaccagaagtgcaa

gcagaacattgcctgctgccagaactctccctccagcgccaactccgacctcgtcggtgtcggtctgccctgtgtcgccc

tcggctccatcatctaagcgctttcctacgctctctggcatcgtgcgagaagcattggcctccgtgtcgcttctggtcgg

ccttcgggctatctggatttcgacaccagcgctaaagtgaactgttatcgattctcggggaaggaaagcatgagttgctg

gcatttgatttgaatagttaggcgatttgatcttttcacg

>Hypothenemus_hampei_contig_11146|c0_g2_i1 Rodlet peptide (Fragment)

ctctttctgaagtacagtcttctctctttcttataaacctaaactccgagaacacctttcgttcaaacccaaacccacag

acacaatgcagttcactctctccactgtcctggccctcgctgccaccgttgttgctctccctccctccggcccctctgct

ggtggtgttggtaacggcaatggtgtcggaaacaagggcaacaccaacgtccgcttccctgtccctgatgacatgaccgt

caagcaggcccaggccaagtgcggtgaccaggctcagctctcttgctgcaacaaggccacctacgccggtgacaccaccg

atgtcaactccggcc

>Hypothenemus_hampei_contig_11147|c0_g1_i1 similarity to HYPOTHETICAL TRANSMEMBRANE PROTEIN YSV4_CAEEL

gccaatcaggccccggcaactggacctgacaggtacattgttttgcagggggcaagggcatgccccgggctttgtgtttt

taaaatgcaaatggacgagccctgaaatggtcgggcagttggtcaatgtctctgtggcactgctggtgctttacaacgtg

ctccaggcattcaaggcgtccagggaggacaggaagctgcataagagatacaacaagtacttcatggtcattgcggtgtt

ccttgtgctggacaacctgctttcgtttatactcgacttcgtgccgttctaccagctcttcaagctcatggttgttgcat

ggatgtcgatcccggcctgcacgggggctgtgtttgtctacaagttctacatccacggggtcatggccaggtacgaaggg

gaccttgaggagatggttgagaggataaggtcgatggtgtctggatacttcaacgagtactacgagaaggcacagaagaa

gtaccgggatagcaggtcctctgtcgcaggcgaggggctggttgggatgagggctgttgaggaggcccgcaaggcgtctc

cggccgatctgaacatggaggaggcgtctgactctgggttttcgaccatcgactcgacggccatcaacgaggagaagcac

agcccgggacttgactcgcgatggtcgaacaagccctcgaacgacgtcgagggatgaataaaaggtttc

>Hypothenemus_hampei_contig_11160|c0_g1_i1 Y854_ENCCU ame: Full=Uncharacterized membrane ECU08_0540

atctaaagtatcgaagatcaaggcttttggagagaagttcaagtcgatcaagtcaaagatgacgctcaacgcggtctttg

tgacccttgggttcatcggggccattgtcaagggagtcaacttcattgcctacaagaagttctttgcagatgggggtaat

gtttggggcctcgatagcggttcttcttggaagaagacggtcgtgttcaccgacctcacgctgggtgggcttgtagcagt

ggcagggctggttgcagcgtgggatatgctggatgggatatgcaggacagtgaggtctaacaagccgagggacaagagct

tctatacgaaggttgtggcggcatcgatacagtttgtgctgtttctgatgaccatgaagatgctgttgttctatccgtat

caggacaccaaacatatccaaggtagtccatctcagacaacgcctcgtgtgaccgcaaaggaattcctccatgataggaa

catgattgggatgcttggagggatctttgcggtctacatgttctacattggaaacagccttattatgagctacaacagga

actcgaagcgagatattgcgaagcttgtgcttgcgggaatagggattttggcagttggatctacgcttgtgttttcgctc

atgaagaagaatgatcccagcgatctcagcaagaactttgtttctcttgcgcttgtggctgttggtatagtgatgatggc

tgtgcaggcgttccttggcgacaaccagggcctggacagtgagatgcctgagtcgccactgtaccagatagtgtcgtgtg

tggttgcagtgttgctcggagttgcggtgctctgtggggcatggaaggttctatataacgatatcaagcttaaggaccta

atcaagagctttgcgttccttggaatgaacaaggccttcaagcagaagctgggcccacttgctcctccggctcaaacaac

ggtcgaagaggattgacgactcgggggggggtagtacaagggcctctctttggcttagttaaccaatctaattgaggcgc

>Hypothenemus_hampei_contig_11181|c0_g1_i1 20S proteasome sububit alpha

agtgtaaacacatacagctcggagggcaggatccaccagatcgagtatgccatgaaggcaatgaacctggggacgacgac

gataggagtgagggccaaggactttgttgtgctgtgttccgagaagaaggtgctctcgacgctgcagaacccgaggagca

ttgtcaagcactacaagatctacgaccacgttgcgcttgggttttctgggataagcggcgacacaaagacgattgtcaag

aaggcacgtgacttctgcatctcgcacacgcacatgtacggggaaaacgccagtgtggagaggctgctgaagtacctgtc

gagcctgagcctgcggtttggggaggaggacgaggcaaagatgatcttccgcaggccgtttggggtgtcgctgctgatag

cggggttcgacacgcagcccaggctgtactcgctggacccgtctgggtcgtacatcagctacaaggcaaaggccattggg

tctgggcacgaggtggttagcggggttcttgaggacgagtacgaggaggactgcgacgtcgacacgctgctgcagaggac

gctgcacaacctgtcgaaggtgatgaaggacaagataagcaaggacaacgtggaggctgcggttgtgaccaaggagggcg

tcaggttcctaactccggaggaggtgtaaggatatctaatgaaatgaactttcagg

>Hypothenemus_hampei_contig_11240|c0_g1_i1 HIRA-interacting 5

attagcattgaaattatttagtgttgatggaatcaggtcggttatgtttggaagtaatttcattaccattgaaaaatcta

gtcaagatttgaattggtcattattaaagccggagatattttcaattttaactgaatatttaactaatggaactccaata

ataagtgaaggacatgaattaagtaatgatatggaaatcagtgaggaagacgatgaagttatatcgatgattaaagagtt

aatcttcactagaatcagaccggcaattcaagatgatggaggagatattgaatttgttaattttcgagaagatgatggaa

cggtttatttaagattgaaaggggcatgtagatcatgcgactcactgtcagtaacattgaaaaatggaattgaaagtatg

ttaaagtattatattgaagaagtacaaaacgttgaacaagttgatgaagattttgaaatcaatacatctacga

>Hypothenemus_hampei_contig_11242|c0_g1_i1 hypothetical protein ECU07_0120

aacagtgtgcaggagtactcgaacttgcccatagaggaggtggaccagatgacggttggggaccggctctgcgagcgatg

caggccggcatactctggaaagagggttgtcccgtgcacgacgttccgcgagtacttgcggaagctcccgctgtgctcgg

ggtgcaagaagagcaacgagagatgcataaagaatgcgtttttcaagagcttcatcctccacaggacatacgaaagggtg

ttttccatgcggtcgattgcactgcacacggcatggttctatctgatgggcgggtctgtggcctacagactcgttgtggt

caatctgattgcctggagga

>Hypothenemus_hampei_contig_11248|c0_g1_i1 aspartyl-tRNA synthetase

tgcgaaaagcctgcggagggcaagggagacctgacgcagcaggagttcagtgcgctgaagaaggtgacgcacgagtcgta

catcgaggttcgcgggagggtggccagacaggcaacggagatctctggatgcagcaagagggacattgagatccggatcg

ttgggttcagcgtgctgagcacggcagacaagagcctgccgttcagcatgaaggacgtgtctgcaactgcagaggagagg

gagaagaacccgacgctgcagagcgttgcataccacctccggctggacaacagggcaatggacctgcgtgcgcctcagac

gcgggccacgttccggctggtcgacggggtgatgttcttgttcaggacgtacttgcggagacatgggtttgtggagatca

ag

>Hypothenemus_hampei_contig_11273|c0_g1_i1 HRD ubiquitin ligase complex ER membrane

tccctgctctacttcctgaccgacttccttcttgtcgtgtccacgttcgacagcgacgttagcttcaagaacggcctgct

ctttgtcatgctgctatgtgtgaagtctcttagctggcttctcggggcaaggatcaagagggacgtccatccctctctgt

acacgcttgcatacggcatatcgttgttttccggggcaatgggggttgtgtttgccctgtcctgcatgtcctcgatcgac

ggacagatcctctttctcttcgagtacacgctgcttgtgattgcatctgcaaagaacatctttgttatgaacctacttct

gtctgaagacgatga

>Hypothenemus_hampei_contig_1130|c0_g1_i1 chromosome segregation ATPase

cgagttcatggagggcttttcgcagatctcgagggggctcaaggagatctacaaggcaatcacatacggggggaatgccg

agctggagcttgtggactatctggacccgttcagcgagggcgttgttctgagtgtgatgccaccaaagaagagctggaag

aacgtgggccacctgagcggcggggagaagacgctcagcagccttgcacttatctttgctctgcacaggtacaagccatc

gccgttctatgtgatggacgagatagacgcggcgctggactacaggaacgtcggggtgatctcggcgtacataaaggaga

tgtcttccacggcccagttcctggtgataagcctgaggagcgacatgttcgagcttagcgagacgctccttggggtgtac

aagacggacaatgtgtccaggtcgcttgttgtgaatataggcag

>Hypothenemus_hampei_contig_11340|c0_g1_i1 hypothetical protein AUD_9179

ttatagtataaattgtcctatcatctaaaatatctattattatcgaccagattacatctttcacggaaatggatctcgcc

cagaagcacggacacctcaagaacgagccccgacaccccttagagtcgcagcagccccacgtggattctcaaagtgaggt

caacaacctcttaggcagcttcggccatccccagaacgaccaccccgatcggagatctagtggcgccggcaacatgggaa

acgtcgtgcaacaggcgcgagagatgactgggcgcaactccggcgagctatctgatcgaaagaactcacaatctgatgcg

tttaccgacgggattagggagc

>Hypothenemus_hampei_contig_11357|c0_g1_i1 hypothetical protein EROM_021440

ttgtgggggatgcctggggcgtggagtacctgaacaaggagctgttcatgtttgcgtctggtagggcgcagtactttcgg

cgctacgagaggctcaaggagttcaaggaccgggaggccaggtttgttctggtgcggggcgactcaatggtgtcctgcgg

gcggttcaacatagatgtgtatgcgatgggcaagtacgagcttgagaaggaggaccaggaggagagcgagtcgtctgagg

tgatgagggttgcgcgtgtgatgaacagcggggtgtacgaccggtttctgtgcgggctggagcatctgccgccgggagtg

tctggcgaaggggc

>Hypothenemus_hampei_contig_11383|c0_g1_i1 ribosomal S13p S18e

tctgacaattaacgcggtcccgtcgcgccatcgcccatcatgtcgctcgtgtccggcgagaagacgaacttccagtacat

tctgcgtttgctcaacaccaacgtcgatggcaagcagaagatcatgtacgccttgacccagatcaagggtgtcggtcgcc

gctactccaaccttgtctgcaagaaggccgatgtcgacctcagcaagcgtgctggtgagctcaccaccgaagagctggag

cgtatcgttaccatccttcagactcccacccagtacaagatccctacctggttcctgaacagacagcgcgacatcaccga

tggcaaggaccaccagatggtttccaacactctggacagcaagcttcgtgaggacctcgagcgcctcaagaagat

>Hypothenemus_hampei_contig_11383|c0_g2_i1 ribosomal S13p S18e

caaccgcccatcatgtcgctcgtctccggggagaagagcaacttccagtacatcctccggttgctgaacaccaacgtcga

tggcaaggagaagatcatgttcgccttgacccagatcaagggtgttggtcgtcgttactccaacctggtctgcaagaagg

ccgatgttgacctgagcaagcgtgctggtgagctcaccaccgaagagctcgagcgcatcgtcaccattctccagacccct

acccagtacaagatcccctcctggttcctgaacagacagcgcgacatcaccgatggcaaggactcccaggccgtttccaa

cgctctggactccaagctccgtgacgacctcgagcgcctcaagaagattcgctcccaccgtggtctccgtcactactggg

gtctgcgtgtccgtggtcagcacaccaagaccactggccgccgcggacgcaccgtcggtgtcagcaagaagaagggctaa

acgatctgagagggttcaacttattttttttcttgctcgctggaagggagagggaaagcgttggtgtggtactttacgaa

accgggacc

>Hypothenemus_hampei_contig_11383|c0_g2_i2 40S ribosomal S18

tctgacaattaacgcggtcccgtcgcgccatcgcccatcatgtcgctcgtgtccggcgagaagacgaacttccagtacat

cttgcgtctgctcaacaccaatgttgacggcaagcagaagatcatgtacgccctgactcaggtcaagggtgtcggtcgcc

gttactccaacttggtctgcaagaaggccgatgttgaccttaacaagcgtgccggtgagctcaccaccgaagagctcgag

cgcatcgtcaccattctccagacccctacccagtacaagatcccctcctggttcctgaacagacagcgcgacatcaccga

tggcaaggactcccaggccgtttccaacgctctggactccaagctccgtgacgacctcgagcgcctcaagaagattcgct

cccaccgtggtctccgtcactactggggtctgcgtgtccgtggtcagcacaccaagaccactggccgccgcggacgcacc

gtcggtgtcagcaagaagaagggctaaacgatctgagagggttcaacttattttttttcttgctcgctggaagggagagg

gaaagcgttggtgtggtactttacgaaaccgggacc

>Hypothenemus_hampei_contig_11404|c0_g1_i1 histone H3

aactgcaagaaaatccactggtggtaaagccccaagaaaacaattagcttccaaagctgccagaaaatctgccccatcta

ctggtggtgttaaaaaacctcacagatataagccaggtaccgttgctttaagagaaattagaagattccaaaaatctact

gaattattgattagaaaattacctttccaaagattggttagagaaattgctcaagatttcaagtctgatttaagattcca

atcttctgctatcggtgctttacaagaatccgttgaagcttacttagtctctttattcgaagacaccaacttgtgtgcta

tccacgccaagagagttactatccaaaagaaggatatccaattg

>Hypothenemus_hampei_contig_11404|c0_g1_i2 histone H3

aactgcaagaaaatccactggtggtaaagccccaagaaaacaattagcttccaaagctgccagaaaatctgccccatcta

ctggtggtgttaaaaaacctcacagatataagccaggtaccgttgctttaagagaaattagaagattccaaaaatctact

gaattattgattagaaaattacctttccaaagattggttagagaaattgctcaagatttcaagtctgatttaagattcca

atcttctgctatcggtgctttacaagaagccgttgaagcttatttggtttccttatttgaagatactaacttgtgtgcta

ttcacgctaagagagtcaccattcaaaagaaggatattcaattagccagaagattaagaggtgaaagatcataaatttaa

gagtctttt

>Hypothenemus_hampei_contig_11404|c0_g1_i3 histone H3

cagaaaatctaccggtggtaaagccccaagaaagcagttggcttctaaagctgctagaaagtctgctccagtctctggag

gtgttaagaaacctcacagatataaaccaggtaccgttgctttaagagaaattagaagattccaaaaatctactgaatta

ttgatcagaaaattgccattccaaagattggttagagaaatcgctcaagatttcaagtctgatttaagattccaatcttc

tgctatcggtgctttgcaagaatccgtcgaagcttacttggtctccttgtttgaagacaccaacttgtgtgctatccacg

ccaagagagttactatccaaaagaaggatatccaattg

>Hypothenemus_hampei_contig_11404|c0_g1_i4 histone

cagaaaatctaccggtggtaaagccccaagaaagcagttggcttctaaagctgctagaaagtctgctccagtctctggag

gtgttaagaaacctcacagatataaaccaggtaccgttgctttaagagaaattagaagattccaaaaatctactgaatta

ttgatcagaaaattgccattccaaagattggttagagaaatcgctcaagatttcaagtctgatttaagattccaatcttc

tgctatcggtgctttacaagaagccgttgaagcttatttggtttccttatttgaagatactaacttgtgtgctattcacg

ctaagagagtcaccattcaaaagaaggatattcaattagccagaagattaagaggtgaaagatcataaatttaagagtct

ttt

>Hypothenemus_hampei_contig_11443|c0_g1_i1 hypothetical protein AC631_03645

acaattcaaaaacaaccagccgtcttttacgttaatgccgctttattcgattgcgatggtaccttagttaactctaccgg

tgctatctctgaattctggagagattttggtaagaccagacctcatgttaacccagaagaaatcatcagaacttctcacg

gttgtagaacctttgatgttatcgccaaatggtctccagaagatgccattgaagaacaagttactcaatgggaaggtgat

attcctgattcttttggtcaatacgctaaaccaattcctggtgctgttgaattagttaaatcttttgataatttttccaa

agaagaaactgaaaatgctaaacaaagatgggccattgttacttctggtactttacctttagccaccaaatggttaaaat

tattaactatccaaaaaccagactgttttatcactgctgaaaaagttactaaaggtaaacctcatccaatgggttaccaa

tccgctagaaacactcttggttacgaagattcaaataaaaaagttgttgtttttgaagatgctccagctggtatcactgc

cggtaagggtgctggtgccatgattgttggtatctgttccacttacgatccagaaaaggtcagaaaatctggtgctgata

tcgttgtcgatgacttgtcatcattcaagattgattcttacaatcctgaatccaacgaattcaaggttgtggtcagtgac

tatcattacgccaacgatgaatacttgcaagaagttgcttaagttattgg

>Hypothenemus_hampei_contig_11443|c0_g1_i2 HAD-like protein

acaattcaaaaacaaccagccgtcttttacgttaatgccgctttattcgattgcgatggtaccttagttaactctaccgg

tgctatctctgaattctggagagattttggtaagaccagacctcatgttaacccagaagaaatcatcagaacttctcacg

gttgtagaacctttgatgttatcgccaaatggtctccagaagatgccattgtcgaacaagtcactgaatgggaaggatct

attccagactcatttggtcaattcgctaaaccaattccaggtgctgtggaattagtcaaatcttttgacaagttctccaa

agaacaaacccaagatcaaaaacaaagatgggccattgtcacttctggtactttaccattagctaccaaatggttaacat

tgttaactattcaaaaaccagactgtttcatcactgctgaaaaagtcaccaaaggtaagccacatccacaaggttaccaa

agtgccagaaacactttgggattcgaaaacccagccgctaaggttgttgttttcgaagatgctccagctggtattaccgc

cggtaagggtgctggtgccatgattgttggtatctgttccacttacgatccagaaaaggtcagaaaatctggtgctgata

tcgttgtcgatgacttgtcatcattcaagattgattcttacaatcctgaatccaacgaattcaaggttgtggtcagtgac

taccattacgccaacgatgaatacttacaagaagt

>Hypothenemus_hampei_contig_11467|c0_g1_i1 hypothetical protein EROM_081330

cgagtgcggccacaagatcacgatgcactcgtgatgggggctggggtgtctgggacaacaagtggttgaacttgggatct

ggagagccctgcaaatggagaggatcgagaggatgtcgatgacaaggcagctgcttgtgctgatggttgcatgcacggtg

ctgaccaggatgtatgggccggtacctctaatggcgtatctgggtgtgcagtgtgtggtcgcatgtctgtttatcctggg

agccactctccacggggcgcttcgcataaggagctgcagcaaggagaacaaggagtacatggagaggatcaggcgccatg

acgacgcccttcatgaggcgcatgcgggacaacagggggat

>Hypothenemus_hampei_contig_11478|c0_g1_i1 S-adenosylmethionine synthetase

cgacaggagcggggcatacatggcaaggtggatagcaaagtcgcttgttgcgagcgggatctgcaggcgggtgcttgtgc

aggtgtcgtatgcaattggggtttgcaggcccttgtctgtgcacgtcgacacgtacggaacgggggttgttgacgacgag

acggtgatggggctggttgtggagaacttcgacatgcgtcccggggcaatctcgctggagctggggctggacaagccgat

cttcaggaggacggcggtgtacgggcactttggaaggcctgagtttgcatgggaggtgccgaaggacctgaggctcgtgg

gctctgccaagccggggaaataaagc

>Hypothenemus_hampei_contig_11481|c0_g1_i1 RNA-binding

cctcgagggccgatgctctggaaagccgccggggatttgttctgacagcaaggaagtctctgcggcactggggaagacct

tgcagcgcgacatgggaaaggcgatgcgcaaggggccggttgggaagagcatagtgcggaggggaagctcgggggccctc

agctttttccaggaggtcgagagagaggtgtgtgcccagtggggagacaacgtgttctactgagaaggccgtgcagagag

cctctgagcagcagtggataattttcgccagattcatagccccatggaggcccagacgagagtggttcccattcgtcctg

caaggatgaagagcatgaaggacgagtggatgaagatatacaccccggttgtcgagatatgcaaggtgcagatccgcatg

aacatccggggaagggccgtggagatgaggacgtgtgagcacacagaggacccctcgcacttggagcggtctgctcagta

catcgaggcgatcaacgtcgggtttcctgtcgaggatgcaattgcggtgctaaagttcagcgacgtgtttctggaccacc

tcgagatctccgaggtcaagacgctgaaggggctgcatgtcgagagggccattgggcgtatcataggaagagaggggaag

acaaagagcgcgatcgaggagttcagcaggtcgaaggttgttgtcaaggaccagaggatccatctgcttgggactgtcga

gaacacccggatagcaaaggacgcggtgtgcaggttgatcatggggtcgcagccgggaagcgtcttcaacaggctgcgga

tcatcaactcgaggctcaaggagaagtacggcggggtccagacggtatacaacaacctagagaagaagcagtaggagggc

cctgg

>Hypothenemus_hampei_contig_11498|c0_g1_i1 hypothetical protein EURHEDRAFT_413184

tcttatatattctgcaatgaatccctctcaacctagacagtatcagacattctccaatcctctacattctaaacttatca

tttacctatcctaatttttctgtgaattttccgggtcaacccaaatcctagcaaaatgtccgacaagatgcatttggctg

ctcgcgctgagcaggatctcaacagctaccaggccaaacagggtcttggccctaagagtgactccacccttgagagtggc

gtcaacgagatggtcgataagaaattcgaagaatccacaggcgtgaagaccggccgagctgcaggcgctaccggaagcaa

ccgcaagcctatccccgaagatgaaggtggtactcgtgacgaccgcggccgtttggcacaggccggagagtacgaaggca

agggcggacccgaggacaaggtgaagcttgagtctgagcgtcgtcctggtgatcaggacacgctgaaccttcaagatatg

aagcgtgagggaattgc

>Hypothenemus_hampei_contig_11510|c0_g1_i1 hypothetical protein ECU11_1480

cggtgacgatagggaggacatccctgttcaagttggtcgagaacatcctagaagaagggaaagtggatgctctggcgaac

accatcacagagtatttgatgggcaatagatacagcagggaccaggcgctcgagtacatatcggactacttggagagcga

gctcgagaaggccggaatagacctagaagacgatatcgacggcgtgtcgttggcagtcctgtttgtatatgaggagatgc

ttgaaaacaagcaggagttcttttcgaagatccaagagaggtctgcccatgtggccccgctcgcagactcagactcggag

gaatagctcggcccgtcaaaactt

>Hypothenemus_hampei_contig_1155|c0_g1_i1 hypothetical protein ECU04_0770

gaggcaacgttgccccgaacaagctgaagggcaagtaccccatgagaaaggccctgggaatgggagccaagctccgaagc

cccccagagacagatgaccagggctctcttgcaagccattacagaatggttattaagagaattgccgggaagacagggag

cagtggcgacggtgaggtctggaggagggagctcaaggtcatcacccagtaccacatgaggaaaatagggatgtcgagcg

agctggaaaaggtcggtgcattaatagacagcagcagcatggcacacgtgctgatgaacatcctgatgcttctccaagaa

acagtcaacaggtcgaagagtgcagaggtggtgtctgactgcatctccttcaagaaggacctcattctgtttggcgacca

ctacagagatcgg

>Hypothenemus_hampei_contig_11570|c0_g1_i1 MRP-like ATP binding

cagaacgtctctctcggcgactcctcgagagcaatcgacttctgcacgtcgaacggcatcccaatcgttggactcgtcga

gaacatgagcgggtactcctgcgagtgctgtggagatgtcagtaacatctttggggcaaagggcggagaaaggcttgcca

aggagctggagatcccctttgtgtaccggctaggcatcgacccgctgctgtgcgaagcactcgacgaaggggcgtttgta

aagacctgcgggtcgatggaaacatacattgggttcaggaaggccatgctcgaggtgatgggccttccaaagatcccgga

ataaagcgct

>Hypothenemus_hampei_contig_11571|c0_g1_i1 symplekin domain-containing

ctggctgatcaagagggacatctggaagcaggaggaggttgtggagggggttgcacggtgcctcgaggcgattggggcgc

cggccgtcgacatgattgtacatatggcgcccgacgggatgcacagggtgcttggcaagagcaaggggctgcagaggctg

tgccgcgagtacctcaagcggggaggaggcgacaggcgcagggacgctgagctcagggctgcggttgagcgggcaggcaa

taaatgacattggtataaatacttctgctgagcagacccccgaagagtgatgatatttgccgccccagactgacgactga

cgaca

>Hypothenemus_hampei_contig_11572|c0_g1_i1 Acyl-coenzyme A oxidase

tacttccttatttacaaagtattgtccaattatactctgctagtgttgttttggataaattcagtggtgatttcttggct

tatggggtattaccaactactttattaagcggattaacttctaaacatatcccaagtttattgaaagccgttagacctca

cgttgttgctctcactgattctttccaatatcctgatatgattattaattctgctattggtaattacgatggtaatgttt

acgaaaattatttcaaagtcgttaaagaaagtaatcctccttatatctccaaagctccttattctgctgatttagaagct

atgttgaatagacccgctttggctgaaagagaaagatttgaaaaaactccggaagttgctaaaatcttattcaaataatt

gatttgatccgacttactttgcttttgccct

>Hypothenemus_hampei_contig_11576|c0_g1_i1 hypothetical protein EROM_010960

acaggaagaggctgtcgcatctgatcaatggatatatatcgaccaaactggttgtgccattctggaacgaatacaacaac

atggctctaatgggatatgtgatactccacaaccacttctgctccgtagagatcaaaatctcgcggattctcgagctgat

agacggcgggggtattcaggcttctgacgagggtatggttgataggcagaaaatacacgaggaaatatcgaggctcattc

cagacctcggggcgtatgagctggacattccgatcgacatcgaagacatcttttggaggaccataaagctcttcgtgagc

ttttcctccattctgctgcggtcaaggtttgtccagatcacaaaggtgtatggaatcaggacgttccgggatctgattga

cgtgtttaggaacgtggctgtggattgctatctgtacaggataaaggacaggctcaaggaggaggtgagcagaatcataa

cccagaagaagataaggcgaagatccactaaggagctgatgacaggggggtgttccggctgacaaaggcccctgccgtcg

aaactagggtttc

>Hypothenemus_hampei_contig_11579|c0_g1_i1 serine palmitoyltransferase 2

aggagcgcagaaaggtcaagagcagtgcatactacgagcgcaagaaggccgctcgccgccaactcacccacgctcagaag

tcggcgggtgttaacgagaagaccaagagccagctcgctgagtacggatattagatacctagttgcgcggccggttgctt

gtgtctgtgatgggatgggttgggatcgcgagcggttttctttcagcggagtggtggacggcgaaccgatcgaccttttt

ctcaccgtccctaccctgtcatgaaaatccacaatactcatgttgtgacgcacatggggggcttggagagaccgatttct

gtttataaaaaaaaaactacggagtcgcttgggatgaaaggttgttcaaaatagctgaccgttattt

>Hypothenemus_hampei_contig_11582|c0_g1_i1 Formate dehydrogenase

agctgctctactacgactaccagccgctgagccccgaggtcgagaaggagatcggctgccgccgtgtcgatgacctcgag

gagatgctcgctcagtgtgacattgtcaccatcaattgccccttgcacgagaagacccgtggtctgttcaacaaggaact

cattgccaagatgaagaagggctcttggctcatcaacaccgctcgtggtgccattgttgtcaaggaggacgttgccgatg

ccgtcaagtccggtcacttgcgcggctacggaggtgatgtctggtttccccagcccgctcctcaggaccaccctctccgc

tacgcccagcacccgtggggcggcggcaatgccatgg

>Hypothenemus_hampei_contig_11583|c0_g1_i1 Histone H2B

ccactggcgagaagaagaagcgtggcaagacccgcaaggagacctactcctcgtacatctacaaggtcctcaagcaagtc

caccccgacactggtatctcgactcgtgcaatgtcgattctgaactcgtttgtcaacgatatcttcgagcgcgttgctac

tgaagcctccaagcttgcctcatacaacaagaagtccaccatctcttcccgggaaatccagacctccgtccgtcttatcc

tgcccggtgaattggccaagcacgccgtctcggaaggtacaaaggccgtcaccaagtactcttcctctgccaaatagatg

ggttggtggtgacttttgttttacttttgctcttttttattgtactttttagctgcttcgggctgtcatatcgaggcagc

gggt

>Hypothenemus_hampei_contig_11624|c0_g1_i1 Thiamine thiazole synthase

tgtatggacccaaacacaatcaattgtaatgttgtgttatcagcagctggtcatgatggtccttttggtgcaggaactgc

taagagaatggccgaggtatcccaaatcgaacttggtcacatgagaggtcttgacatgaacactgctgaggatgctatag

tcaagggtacaagagagattactcctggtctagttgtcaccggtatggaattggcagaagtcgatgggttcaatcggatg

ggtcccacattcggtgcaatggctttagctggagtcaaagccgcagaagagactttgagagtttacgaccaaagaaagca

gcaaaatcttcaagatattaagaatttgaatcacaatgctccaaaatcttatta

>Hypothenemus_hampei_contig_11627|c0_g1_i1 ribosomal cytosolic

gaccgtgctaagctcattggcctcgagttcatgtcaactgaggatttgaagaagcttaacaagaacaagaagctcgtcaa

gaagttggccaagtcacaagacgccttcatcgcctctgaagccctcatcaagcaaattcctcgtttgcttggtccaggtc

tctcaaaggctggtaaattcccaactccaatctcccactcagatgacatcgaaaagaagatcaacgatgtcaagtcaacc

atcaagttccaacttaagaaggttctctgtatgggtgttgctgtcggtaacattgacatggctgaagacgaaatcgtcgc

caacgttgtcct

>Hypothenemus_hampei_contig_11630|c0_g1_i1 Sporulation SPS19

atgtaggtgcagccaaggctggtgttgatgcgttatctaatgctttggctgtagaattgggaccattgggaatcacttct

aattgtattgctcctggagccattggaggaactgaaggtatgtccagattaacacctccaggggttaaaccagtacaaga

aagagttccattgcaaagagaaggaaccaccagagatattgccgatgccactgtttatttgttctctccagctgctgatt

acgttacaggaaccgttcaagtcgttgatgggggattatggcatatcggtggatttatgggtgatatgtaccctctggtg

attaagagccaaggtgaagacgatccaaagttatgaagtat

>Hypothenemus_hampei_contig_11657|c0_g1_i1 proton-dependent oligopeptide transport

cctggctcctgccgtgtttttctggatgctctacgaccagcagtcgtcttcgtgggtcgaccagggctcgaagatgggca

cccgccactcgctgctcgggctctcggtcgacgtcctgccgtcgcagatgcaggcattcaactcggtcttcatcctgctc

ttcatccctcttttctccagggtcgtctatccgtcgatggccaggctcggggtgcttgcgtcgccaacagacaagatggg

ggttgggatcgtgcttgcgtcgctgagcttcctctgctcggcgtgcctcgagcacaggatcgctgcgcttgccccttccg

gcgagagcctgtcgatcctgtggcagctgccgcagtatgtgctgctgactgccggcgagatc

>Hypothenemus_hampei_contig_11672|c0_g1_i1 hypothetical protein ECU03_0160

caatgcaaggcagaagaggctctcgctgataacgatggagagctatgtggaggactcgtacacttggtatgtgcgagcac

tcgagtttgtcgaaaacgcctttgggatccgccttgtcactgtcgagcaggagagcaacgagatgtacatgcggttcgat

gcctccgggtgcgaggtcgggatatttatcagggacgggcagatggtcgactcgaagctgtacaacgcctccgatggcaa

tgcagccctgtcgtttgaggcgttgagccggcttgcaactcttgtcaacgatccccgcatccttctgacgcaggttgcaa

gcaga

>Hypothenemus_hampei_contig_11687|c0_g1_i1 hypothetical protein EROM_081010

tgcggggaagggtgatgggcaaggcagccgagatgcggagggctgaagacaaggagaacgaggctgagggggagtttgat

ggggacgtgtttgagcaggagagcctgattgcagggcacattccggttgtgaagagcagagacgaggccgaaatgccgag

ggccaagaaggcctctgaggttgagagtcttgggactgtcgctgcaaaaagaagggaggttgtgcctgaggccatggcgg

cagaggcctgtgggaaggaggtggagatgagcaagaatgcggatcaaatgagggggagaggctcaagtgcaaacagcaag

ctgaacatggccttgcagc

>Hypothenemus_hampei_contig_11690|c0_g1_i1 hypothetical protein CANTEDRAFT_117236

ttgaacatccaaagatttccaagatttctttcactggttctactgctgttggtaagcacatcatgaagagtgctgctgaa

tctttgaagaaggtcaccttggaattgggtggtaaatctccaaatattgttttcgccgacgctgacattccaaagactgt

tgacaacattgttacctctattttctacaacactggtgaagtttgttgtgctggttccagattatacattcacgaagaca

tctacgaagaactcatggaagctttggtcactaaggttaactccatcaaggttggtaacccattcgaagaagacaccgtt

atgggtgctcaaaactctaaggctcaattcgacaagatcttgaaatacattgacattggtgt

>Hypothenemus_hampei_contig_11712|c0_g1_i1 DNA-directed RNA polymerase subunit beta

gcacaggtgttctttggacccacctactaccagaggctgaagcatatggtcgatgacaagatacatgccagggcgcgagg

gcctctccaaatactcacgaggcagcctgtcgagggaaggtctagggacggaggtcttcgctttggagaaatggaaagag

actgtataatttcgcatggggcgtctgcatttctcaaagagaggctgatggatgtgagcgatgcatattcgtgctatgta

tgcgatatctgtgggcttcttgcaatgggcggaagccggacaaacgaatgcaaaggatgcagcaacactgcaaatgtaag

catggttgagattccgtatgccttcaagctcctcatccaggagctgatggggatgaacattgcgccgagaatccgctttg

acgag

>Hypothenemus_hampei_contig_11753|c0_g1_i1 hypothetical protein Eint_050790

tacgaaacatcctggctgtcttgccacgacatgggttgtgcagagaagcagggtcgatctggagtgatcgataagaagtt

atggtttccaagggcccatgaagaggccaagcaggaggaaggagcccgaggagaaggggaagaagaaggaagccacggag

gagaaaggcgtttctgagaccaggaggcttagaagggcgatgtgcgagaagatcctggggtcgatagacaagggaattgc

cgacgaggcgagagagcttgctcgatatgctctatacacagatgcacctgagtttgtgaacccttgggggtcaagcgaga

tccatggcgtttcgaacgacgtggccacgattc

>Hypothenemus_hampei_contig_11778|c0_g1_i1 triosephosphate isomerase

tgctttggaaagaggattatcggttattttctgtattggggaaactttggaagaacgtaaatctggtattactcttgaag

tctgtgctagacaattggatgctttatccaagattgtttctgattggtcccatgttgttgttgcttacgaaccggtttgg

gccattggtactggtttggctgctacttctgatgatgctcaagatactcacaaacaaattagagctcatcttgctaaaac

cattggtgaaaaacaagcctctgaagttagaatcttatacgggggttctgccaacggtaagaatgctccagacttcaaag

acaaggccgatgtcgatggtttcttggttg

>Hypothenemus_hampei_contig_11812|c0_g1_i1 Hsp70 chaperone ( )

caacacttcgcttggcgagttcactctggctcccatcccccccatgaaggccggagaagctgctttggaggttgtgttcg

aagtcgatgtcaacggaatcttgaaggttaccgccaccgagaagtcctccggccgtaccgccaacatcaccatctccaac

gctgtcggcaagctttccactactgagatcgaccagatgatcgatgatgctgccaagttcaagtccagcgacgaggcctt

caccaagagattcgagtcccgccagcagctcgagtcctacatctcgcgcgtggaggagatcgtctccgaccccaccatgt

ctctgaagctcaagcggggtaacaaggagaagatcgagtctgccctcagtgatgccatggcccagcttgagatcgaggac

tctacgcccgaggatctcaa

>Hypothenemus_hampei_contig_11814|c0_g1_i1 ATP-binding cassette sub-family F member 2

ggaagagcagggccccgacagcccggttcttatggacctgtacgacccagcgtattgacaaaatggacccctccaccttc

cacactcgtgcttccctgatcttgacgggtcttggtttcaacaaacagacgatcaacaagaagacgaaggatatgtccgg

tggttggcgtatgcgtgtggccctcggaaaggccctgttcgtcaagccgtccttgctcttgctggacgaccccactgctc

acttggatctcgaggcctgtgtgtggttggaagaatatatgaagaagtgggaccgtactctggtccttgtctctcactcc

atggatttcttgaacggtgtctgcacgaacatgattgacatgcgcatgaagcagctcatgtactacggtggtaattacga

ctcgtaccacaagacccgtagcgaacaggaggtcaaccagatgaaggcctacaccaagcagcaagaagaaatcgcccaca

tcaagaagttcattgcttccgctggtacctacgccaacttggtgcgtcaggccaagtcccgccaaaagattctcgacaag

atggaggccgatggtttcatccagcctgtcatccccgaccgtgtcttcactttccggttcgcggacgttgagaagctgcc

tcctcccgtcctgtccttcgacgacgtttccttctcctactccggcaactg

>Hypothenemus_hampei_contig_11814|c0_g2_i1 ATP-binding cassette sub-family F member 2

ggaagagcagggccccgacagcccggttcttatggacctgtacgacccagcgtattgacaaaatggacccctccaccttc

cacactcgtgcttccctgatcttgacgggtcttggtttcaacaaacagacgatcaacaagaagacgaaggatatgtccgg

tggttggcgtatgcgtgtggccctcggaaaggccctgttcgtcaagccgtccttgctcttgctggacgaccccactgctc

acttggatctcgaggcctgtgtgtggttggaagaatatatgaagaagtgggaccgtactctggtccttgtctctcactcc

atggatttcttgaacggtgtctgcacgaacatgattgacatgcgcatgaagcagctcatgtactacggtggtaattacga

ctcgtaccacaagacccgtagcgaacaggaggtcaaccagatgaaggcctacaccaagcagcaagaagaaatcgcccaca

tcaagaagttcattgcttccgccggtacctacgccaacttggtccgtcaggccaagtcccgtcagaagatcctcgacaag

atggaggccgatggtttcatccagcctgtcatccccgaccgtgtcttcactttccggttcgcggacgttgagaagctgcc

tcctcccgtcctgtccttcgacgacgtttccttctcctactccggcaactg

>Hypothenemus_hampei_contig_11815|c0_g1_i1 hin1 like

agttcggttgggtgaagtctccgaagtttaagcccaagatcaagtgcgatttggagattcctttgaactccaatggcaca

gcgtccgggacattcgagtccaaacgctgcggtcttgattggtagccttcttcgcccgatgcttgattgattcatggcct

acacacttgcttctttttcccacccccgcttcttagggtcatagcagtattactctattcttttttgttattattatact

tagaatttgatccgctgcgattcctttttccccagcgttgttcgtattcatcttctactgcatattttttgtgttttttt

ccccgtgttgtttcggagtagtatatgatgtgtgatcatcggcattcacactactgtttc

>Hypothenemus_hampei_contig_11817|c0_g1_i1 ankyrin repeat

aatagctgaataatcaaaagttacttaaatagaaagtcaagaatataaagaagaaaatggacaattattgatgcatgtat

tttatatattaaattcgttacttattaagttgtgtaaccaagtaatagattctgagaataaatataacgcaataatcagt

aagacatcatacatagataatatatctattcgattagagtatcacagtaatttaaaaaaagaaatggttcaatttctcgt

ggctgaaggtgctaagaatgccaagaagaatgcagttttgcacaaagctgctttatgtggaagcttggttataattcaat

ttctggtggctacaggtactaatattgaagttacaaacaatgataccaatacagttttacattgggctgtcgaatctggt

aacttaaagatagttgaatttctggtcactaaaggtgccaatgttgacgctaaaaataaatatggcgatacaatttcgca

cacagctgttatttatagtaggtgggatataattcagtatctagtgactaaaggtgccaacattaatgctacaaacaatt

atggtaatacagttttgcatttggctgttgaatctggtaatttggagatggttgaatttttggtgaccaaaggtgccaa

>Hypothenemus_hampei_contig_11826|c0_g1_i1 Ecm33 domain-containing

tcacctccgctggtgatgtctacatctatgacaccaagctgggctccctggacggtatctccctcgagaccgtcggtgct

ttcgacattgagaacaaccgttacctcaagaccgtcaacgtcaacaacctgaagaacgccactggtgacatcaccttcgc

ttccaactacgacgctctcgagattgaactccccaacctcggcgatggcaagaacctgacttgccgtaacatcagcagca

tctccgttccctccctcaagaagctgtccggctccctcggtttctacggcactgactttaagagcttcattgcccccaac

ctgaccagcactggtgaccttgtcttcatgaacaacaagaagctgtccaacatctccatggcccgccttgagaaggtcaa

cggtggtttccagattgcccgtaacgacaagcttcaggagatcgacctccccaagcttgagcgtgtcactggagctattg

acttcagcggcaagttcaacactgtcgagttcggttcccttaaggaggtctctggtggcttcaacctgcagtctactgac

ggcaagttcaactgcgacaccttcgacaagatgaagaacgacatcatccacggcaagtacaactgtgatgccaagaagga

caaccctaccacctccaacggcaagtctggcaccagctcttccagctccagctcctctggctccagcagtacctcctccg

gtgccgcc

>Hypothenemus_hampei_contig_11828|c0_g1_i1 ---NA---

cacaaccccgagcccgggtttgatgtcgactgggaagccaactgcagtcctggattcatggtcgcgagattcaaggctct

cggccccaatgactgtttttaaacgcgcatttcgtttttgattttctcaagcctgtatactcgattgtcatcagctggaa

cgaagctgagactttcgggtacgaaaagtatataccacatttgatttttcagcgtcactacgagaaccagaggaccacta

cgcaatgttgattagcacattgtagttggtagggatctgctatgcatcataatttttgtaatagggtcttctattcagaa

gccagaagccagaagccagcaatgacaagt

>Hypothenemus_hampei_contig_11831|c0_g1_i1 Superoxide dismutase [Cu-Zn]

aacgctgagcgtgctttccacgttcaccagttcggtgataacaccaacggctgcacttctgctggccctcacttcaaccc

tcacggcaagccccacggtgctcccactgatgccgaccgtcacgttggtgacctgggcaacttcaagaccgatgctgagg

gtaactctaccggcaacatgcaggacaagcttgtcaagctgattggtgccgagagcgttctgggccgtacccttgtcgtc

cacgctggcactgatgacctcggccgtggtggtaacgaggagtccaagaagactggtaacgctggtctccgtcctgcttg

cggtgtcattggcattgctgcttaaatttggagcaatctttgagataaccccattagtatttgggttatgatgatattcc

ccatctactgagatgaaaagttcaggatagttaccctggctaggatgcaatgacaat

>Hypothenemus_hampei_contig_11832|c0_g1_i1 synaptobrevin VAMP

aggatgggcatccgggcgaggtgcggagatgcctggaggagtccctgtgacccacgcggggttgcgatgggcctggggtc

ggcaaatgtatttaaaacaggggatggatagccctgcgatggctgttctgtacacacagataataaagcttccggactac

aggatcctgtctggagagtactctccgcaaagcaactcgctcaagcccgggaacgagatagtcaaggagctgcgggacgt

tgtcaagtcgattccggggggcagcgcgacgtcgttctacacattcaactcgtcggacacgaggtttgtgttctacttca

aggtgggtgctgggcttgtgtttgcagtgataagcgacaagtacacctcgcagaagcttgcctccggatacatggaccag

gttgtcgacagctttgggaagatctacgtggacgatccaaagacaacgtactacacgtttgacccgacgctcaaggcgat

gtccgacaggttcaacagggagtcgaactatgcgcaggggatggctgttgtcgaggagaccaaggggctgcttgcagagt

ctctcaacatgataatcaagagggacgagaacatcaacaacctcaaggggcttgcaagccggatgacgatcgaggcgcag

atgatgcagaagaacgtgcagaggatgcatctcaagagcatgctgaacgactactggatctacatgatctttgctgtgct

ggtcgtgctgttcctgtactatataacccattaa

>Hypothenemus_hampei_contig_11832|c0_g2_i1 synaptobrevin VAMP

tttataaaagacgagaagaccctatagagttttatcaacgtactacacgtttgacccgacgctcaaggcgatgtccgaca

ggttcaacagggagtcgaactatgcgcaggggatggctgttgtcgaggagaccaaggggctgcttgcagagtctctcaac

atgataatcaagagggacgagaacatcaacaacctcaaggggcttgcaagccggatgacgatcgaggcgcagatgatgca

gaagaacgtgcagaggatgcatctcaagagcatgctgaacgactactggatctacatgatctttgctgtgctggtcgtgc

tgttcctgtactatataacccattaa

>Hypothenemus_hampei_contig_11838|c0_g1_i1 F-type H-transporting ATPase subunit J

cgatctccgaaaccacctcgcattcgcaccatctgtcaatatgtctctcctcggaaagaagttccccggtctcctgggca

agcccatgactcccttcttcgccgccggtgctatcgtcctctacggtgtcaactctctgcagaacgctctctccaacact

gccgagttcaagaacgaccctcgcaaccccaacgccaagtccggtaacgctggccactaaattcttgaccgatgacgaag

cgctgtgagaaaccacctagagtggcgtgagacaggagcggattgggttgttatgacaaggaaggggaggaatttatctc

tgtacctcggtcgcaactcgccattgttatgtttaaagttcgcgggcagtttgccctctgtccaagcatatagaagatac

cctgttgggtgacttggatcaattt

>Hypothenemus_hampei_contig_11838|c0_g2_i1 F-type H-transporting ATPase subunit J

cgatctccgaaaccacctcgcattcgcaccatctgtcaatatgtctctcctcggaaagaagtttcccggccttctgggca

agcccatgactcccttcttcgccgccggtgctatcgtcctctacggtgtcaactctctgcagaacgctctctccaacact

gccgagttcaagaacgaccctcgcaaccccaacgccaagtccggtaacgctggccactaaattcttgaccgatgacgaag

cgctgtgagaaaccacctagagtggcgtgagacaggagcggattgggttgttatgacaaggaaggggaggaatttatctc

tgtacctcggtcgcaactcgccattgttatgtttaaagttcgcgggcagtttgccctctgtccaagcatatagaagatac

cctgttgggtgacttggatcaattt

>Hypothenemus_hampei_contig_11858|c0_g1_i1 cytosolic class I small heat shock type 2

cgggtggaacgcagcagcggccaattcatgaggaggttcaggctgcccgaaaatgccaagatggatcagatcaaagctgc

tatggagaatggggtccttaccatcaccatccccaaagaagaggccaagaagactgatgtcaaagccattcagatttctg

gttaactaattatttggtagtaattgtatagttatgcaataaatgtagagttgagagcggtttaagtttgatgaattgcc

ttggtatatgagggcgtctgttgtaatggctactgttactatcctactgtgaactaaagtatgtattccgtgttttgtga

atgaaatgtttttcct

>Hypothenemus_hampei_contig_11912|c0_g1_i1 diphthamide synthase subunit DPH2

atgggcttgaggccaaggacaggatgcagtaccttgggtccaagtgcaacaagggagagaagatccggggcctacagata

tttgcaattgtgttcacgtcgagggagtacgagccgcttgcaaaggccataagggctgcacttgtttcgcggcacaagaa

cgcgtacctgctgcttctgaaggacctttcgtacgagaggatgataacgatcgaggggaccgagtgcattgtggttgtgg

actgcccgttcttcgagtgctctctggatctgcacattcccattgtgacgccgttcgaggttgagtatgcgctgtgcggg

cagtggggcaggttcgacaagaactcgttctgcgtgcctgacgcgcctgcaagcacgtgcacggcgctggagcgcattgg

gagggcggggagtgtcctgcttcggctggcggagcagagcgtgccgttctgccgtgaggacgaggacgacgtcgaggtgc

attgcgggcagagcgggacggcgtgcag

>Hypothenemus_hampei_contig_11953|c0_g1_i1 40S ribosomal S15A

cagtgctcagatcacaaagaccagtgaagcctggccaacatgtgcaaagccatcaataatgccagtagggcagggaagag

gcagcttattctgaagtcatccacaaaggagtcgcgcgtatttcttctccagatgctgaagcacggatacatctccgggt

tttcgcacatccacgacaagaggagtgggaagaacatcatcgacctaaacgggaggctcaacaggtgcgggataatttcg

ccccactacatagtcaagctcggcggaatcgagaacttccgagcaaggctcctacctgcaaggcagtttggccatgttct

gttcaacacctccaagggtgttctggaccacaaggaatgcctggcagaaaacgttggtggccaagtccttggatttttct

attaaaagccattctccaacaatgcctttaattaaagataaccagt

>Hypothenemus_hampei_contig_11976|c0_g1_i1 alcohol-dehydrogenase adhA

tcctgtattcaacacggtggtgcgcatgatcaacatcaaaggaagctatgtcggtaaccgccaggacggtgaggaagcgg

ttgacttctttgctcgtggcttgatcaaggcaccattcaagacggctccgttgagtgatcttcccaagatctttgacttg

atgaaacaaggcaaaattgccggtcgttacgtcctcgacgtgccacagtaaggcgtgggagtgcatggagtttcgtggga

aatttggcgttgggctcgcatatggcattctctattactttgctctctaacccgctatttacattaacgtgtaactgtgc

ataagataacgaaagcttagtcgttctaatacactgcatacagc

>Hypothenemus_hampei_contig_11988|c0_g1_i1 Pck1 phosphoenolpyruvate carboxykinase

attccctgctaatgttcatactaaaggtatgacttcatcaacttcggttgaaattaatttcaaagatatggaaatggtta

ttcttggtactgaatatgctggtgaaatgaagaagggtattttcaccgttatgttttatttaatgccaattaaatatcaa

gttttaactcttcattcttcagctaatcaaggtactgataagggtgatgtcactttattctttggtttatcaggtactgg

taaaaccactttatctgcagatccaaatcgtaaattgattggtgatgatgaacactgttggtccgaccatggtgttttca

atattgaaggtggatgttatgctaaatgtcttgatttatctgctgaaaaagaaccagaaatttttaattctattaaattc

ggttcaattttggaaaatgttgtttatgacccattgagtaaagttgtcaattataatgattcaagcattactgaaaatac

cagatgtgcttacc

>Hypothenemus_hampei_contig_11993|c0_g1_i1 ATP ADP translocase

cgctctaccataccatgaaaaggaagcattattttcgcaggtgttcgttgtgacgtccgtaatcacactggcgtctgcag

ccacaagcttcctcgcagatcggtttgtgccgaaagaagacctgaggcgggccgggaaaaaggaacataaggcccagaag

atgggatttgccaagtctctgaagatgatgcagaagtctaggtttcttatggcaatgacggggacggttgtggcgtcctc

tgtatgctcgggcatatttgaggcaacttacaggggaggaatagtgttgggtgcagtgcaggcctctgcatcaaagccct

cgtacatgaacaagttaaatgccatggcacagataataacctctgcctttctgcttgtgatgttctttaagcctgcagtt

catctcatagaaaagaaaggatggatcctggtggcgatggtcgggcctattgtt

>Hypothenemus_hampei_contig_11996|c0_g1_i1 vacuolar ATP synthase catalytic subunit A

gggcttgacaagaagcttgcgcagaggaagcacttcccgtcgatcaactggaacctgagctactcgaagtatctgtcgaa

cctggacgcgtactacgagggcgaggacccgggctttgttgtaaacaggacgaagtgccgggagatcctgcagctggatg

acgacctgtcggagattgtgcagcttgtgggcaagaacgcgctgtccgagacggagaagctgattctggacgtgtcgaag

ctgatcaaggaggacttcctgcagcagaacgggtactcgaagtacgacagcttctgtccgtttgtgaagacaaggctgat

gctgcggaacatcatcctgtacttcgacaactgcaagaatgccatagcgaactacaagctgagctggtcgacggtgaaga

aggagacggaggaggtgttctatgggctgatgaagatgaagtttgtgatggcggatggggagatggaggcgcggctggag

aagcttcggcgggagatagacgaggtgttcctgaggatgcttgggcaaat

>Hypothenemus_hampei_contig_12012|c0_g1_i1 ATP synthase subunit 9 (mitochondrion)

tcagagttgtcattatatacatcaaatttatgatatacaagaaagaggtaacgaaataagtgaaaatttaagaagcaata

ttagtgatcatataatcgaatgtcatgatgcaactataaatagagatcgtgatacatcaaatatgaatataccatatata

gatcaaagattagttaaagaattagaagaattaagagataattcatctaattcttaattcagctaaaataataggaacag

ggttagctacatcaggtttaataggtgctggagttggaataggaataggaattggagcattaattttaggtgtagcaaga

aatccatcattaagaggacaattattctcatatgctatattaggttttgcttttgctgaagtaactggattatttgcatt

aatgatgg

>Hypothenemus_hampei_contig_12016|c0_g1_i1 glycoside family 17

ggtcctgccgcagaagatgccggtttccaagtcatgtttggtgtttggccaaccgatgactctcatttcgaagccgaaaa

acaagctttgaaagattacttgccaaacatttcggtcgataccgtcaaggtcattagtgttggttccgaagccttatata

gagatgatttatctgcttctgctttggcctccaaaatctccgaagttaaagatttattaaaagatatcaaggataaaaat

ggtaaatcttactcctcggttcaagttggttttgttgattcttggaatgtcttggttgatgatggttctcaaccagcaat

caaaag

>Hypothenemus_hampei_contig_12024|c0_g1_i1 dynein heavy chain

gggaggtgcatgggattctgaacctggtgtttggcaggaagaacagggacttctttgaggcgtacggagtctccttgtgc

tttggagtgtatagggaatatgtggaaaagacgttgtatgcggcgctagcacgagacttgtgtgggggaagcgtggagag

gatgtgggaggggtctgaaagcatagatatggatgcagtgagggatgcagttggcgagggtgtggatcgattctattcgg

cgtcgttccctgggtctcaggccggatacatggaggttgtgagcgagattgagacgtatgggtttgactacacgcttgtg

actgccagcgacgacgcggcatatgtgattgaggagaagattgccgtgacgtctgcaatatcggccgggccggcggaaag

caacgaagagatctgcaggatgctggcagaaaacggggaaaagggaaggacgtatctgatcaggaacatccacttccttg

gaagcgtccggaagaccgggaacagccggctga

>Hypothenemus_hampei_contig_12025|c0_g1_i1 hypothetical protein EROM_111540

gacatggcggcggggaggaccaaggctctcacgaaggcctgtatggcgagagcaacatcatccaggaggagagaaacacg

atccgcgtgaaggttcagcagaagaaggggtcgaagaaggtcacgatcatagagaatctccctgcagacgtccgggagaa

gctcctgtcctctcttaagaaggaaatggggtgtggaggcatacttcttgacgaaaagtcatcgatccagctccagggag

acaagacgaacatggggattgtcggctacctcaagaggtacatcaagggctgcaaggtcgagctgaatggaagggtcagc

tagcctctcctgcgccctgtggggttggaatttattaaacaagcctcgtgtctttgtgcgccgggtgcagaagtcaaaaa

aa

>Hypothenemus_hampei_contig_12058|c0_g1_i1 40S ribosomal S0

gttagtcgctagagaagtcttgagattaagaggtgtcattccagacagagctaccgaatggagtgtcatgccagatttat

atttctacagagacccagaagagattgaacaaacctctgctgaagaatctaagaccgaagaaactgaagaaccagctact

gctgaagctgaaactgaatggactggtgaaactgaagaagttgactgggctgaatccggtgctaacccagctgccgaaga

agctgccgctcaaaactggtaaatgtatagccataataactaattcatcgatcatgggatctgaaatcaagagaaaactc

ataaacaccctttttatatataagaataagatatctgtttagattctgttttgtatattgtatcttaagaatgacataat

ataaattattg

>Hypothenemus_hampei_contig_12074|c0_g1_i1 lysyl-tRNA synthetase

gggaggatcatgagcatgaggctgcatgcgcggttctgcttcttcctggttgtgtctggagggtctgtgctgcagcttgt

tgtggatgtcaaggagacggggcaggcggaggtggcgaagtttctcaagagaggagatgtcgttgggtttacggggagcc

ctgggcggacgcggacgctcgagccgtctgtgtttgtcaaggaggtggttgttcttgcgccgtgcctgcggacgattccc

acagagcactttgggctgaaggatgcagagacgatctacaggcggcggtacatggacctactgatcaacagggagtcgaa

ggaaaggttccaccggcggacgcagatcatcaggtacatccgccacttcctggatggcaggggctttgtggaggttgaga

cgcctatgatgaacctgattccagggggagcggtggcgcggccgtttgtgacgcaccacaacgagctgaagctggacctg

tacatgagaatctctcccgagctgtatctgaagaagctggttgtggggggtcttgagcgggtgtacgagatagggaagca

gttccggaacgaggggattgaccttacccacaatcccgagttcacgtcctgcgagttctacatggcatatgcggactaca

acgacatcatggcgatgacggaggagctgttgcacgggatggtgctcgagatgtttggggcggagactgttgtgtacatg

ccccggaagagggaggaggagacgaaggccgttgagatatcgtttgcgaggccgttccgggcgatcagcatcatcgagga

gctcaacagcaaacttgggctgagcctgagtggcgagacgcttgagagcgaggagacgctggagagccttctggatgcat

gccggcgggaggggctgtcgacagaggggcctcggacgctatcccgggtgcttgacaagctgattgggcatgttattgag

ccgcagtgtgtgaacccgacgtttatcaaggactatcctacggtgatgtcgccgctggcaaagagccacaggtcgaaggc

ggggctgacggagaggtttgagctgttcatcaactgcaaggaggtgtgcaatgcatacaccgagctgaacaacccgtttg

agcagaggaagaggttccagcagcaggcccaggaccagagggcaggggacgacgaggcgatgatgaacgacgaggacttc

tgcatggcgcttgagtacgggctgcctccgacggggggctggggcatggggatcgacaggcttgtgatgtatctgacgaa

tgcagccaacatccgggacgtgattttcttcccgacgatg

>Hypothenemus_hampei_contig_12077|c0_g1_i1 ribitol kinase

ccaactgctactacttcaagtactaatatgagtaccttggtttacgctagtggccatgcaggatcccatcatcaaatgaa

taccatgactccattgggtgaagaacaagttgattatttcggtggtgaaggccaaaagaatcaagatagtgattctgatg

aagaagaacaaaccttatcctttggttccaaacaaaatgtccaacaagatgttcaaaaaggtatccatactaaattacaa

ggattaaacttgaaaccattaactccttctaaagctaaaaaggctgaatccaatcctggtgataaattatggaagattat

ggaaaatatgaccggtccaggtagagtcattttacctagtgacgaacaacatccagatcgtaaattattgaatgccaaat

ataaaatctttttagaacaatgttataaacaacaagaatatagaaatttggttgaccaagttgaagaagaaaatttaaaa

tctttgaagaaacactaatttaatcaaatgttaataatttattagagttaaacgataaataa

>Hypothenemus_hampei_contig_12081|c0_g1_i1 ATP-dependent RNA helicase

ggagcggcagttgctgctatactctgcaacgttcccatacttcatcacggggttcatcaagagatacatgaaggatcccc

tgtgcattaacctagtcaaggagcttgcgcccgtgggtgtcaagcagttctacacacatgtcaagccgtgtgaaaagctg

ctgtgcttgaagtcgctgcttgtgaagtcaagcatcaagcagtgtgtgatattctgcaacaatatcaagaccgtggagct

cctcgcaatgaagattacagagatggggatgtcttcgtacttcatccactcgaagatgacgcaggaggacaggaacgtcg

tgttccataactttgtgaaggggaagtgcaagatcctggttgcaacagacctcatcacgcgcggagtcgacgtccccaat

acaaactacgtgatcaacttcgacgtccccaagtccccggagtcgtatctccacaggattgggagagccgggaggttcgg

gacccctggggttgcaatcagcttggtcaccacgggagagagggagttgctgatggacatagaggcaaagctgggtaagg

agatttctcccctctccgacggggggctaacccgcctgtatgaaaacaacatagacgaagattaagaaagtaataaagca

tcttc

>Hypothenemus_hampei_contig_12083|c0_g1_i1 ECU06_1215

agtcaagctgggaagagtggtgacgcctgcgctgaaggacaggagacacatgcatgcaatcatcgtggggatcatcgacg

caacgttcgtgcttctgcagaggaaggacggcgagagggaggtgtgcgcgattgccgccctgcacctcgaggaggaggcg

ttcgacatcaagggaatgagtgccgaggagatcggaaggctcattccagacgacgtgtgtgccgaggacacggccggcga

tttcgacagattcaaggcgaagctcaggaaggctgttgagaacgaggttctgagagagaagggcctggcatgattaaagc

agat

>Hypothenemus_hampei_contig_12129|c0_g1_i1 40S ribosomal S23

ccagggttattttcggccaacaatctggtaaagagtagaaaggccaagaggctagcagacatcacgtacaggaagcgggc

gctcgggacgaagtataagcacagtgtgctcgggcgagcaccccaggccaaagccattgtccttgagaagattggtgtgg

aggccaagcagccaaactcggccataagaaaggccgttagatgccagctgattgcaactgggaagaagataaccgccttc

gtgccatacgacgggtccgtgacatacatcgagagcaacgacgaggtgactgtcgagggctttggtaagaaggggcggag

cgtgggagacatccccgggattaggttcaaggtgtgtaaggtgcagaacgtgtccctacacgcgatatttacaggaaaga

aggagaagccttcgaggtaatattaaatgctcctggca

>Hypothenemus_hampei_contig_12130|c0_g1_i1 1-acyl-sn-glycerol-3-phosphate acyltransferase

aaaaacatggctcgagcgatgaagagagtctggaacgccatgtgctttgcagcagtgatgggcgtgtacttctcgtacat

tgtttttgggtacgtggttatttttgcagtgaggcttgtgcttcctggggaggctgtacacaacacattggcaagggtgg

tgaaggttgggtggctccacctcacgagagcagtgctctacggatactttccgcggaaggtgtttatcaggtacgacccc

atagtgcttgagaagagcaggaacgtcgtgatctcaaaccacctgacggagtacgactggctgtttgtctcgaccgtact

gcaccatttcgggaggttcgaggacatatgcatcatcctcaagatgtctttgagggacattccg

>Hypothenemus_hampei_contig_12131|c0_g1_i1 CTP synthase

ctgtgcgtttgtgtccgtcgactacctcgtgacgctgcccagcggcgagcagaagacaaagggcatccagatggggtgca

ggaacttccgcgggttcgggctcaagtacgacgtcgtggtgtgcaggggctgcagcgaggcaagctcagagacgcgcagg

aagattgcggcgacgtgctggatgcgcgaggacgacgtgttcagcctgccggacatggcgtctgtgtatcttgtgccgca

gtacctggaggcacacgggctggtcgactcgctcaacagggtgctggggatcagcgacaggtctgcagacaggacgatgc

tggacgtgtttgcacgcgttgctgcgcggcacagggacagcgtgcgcattgcgattgtgggcaagtactcgcccgagctc

gacagctatgcgtcgcttgtgcatgcgctgcggttctcgggggcagccgtgggcgtcaacgtggatgttgcgtgggtcga

tgcagaggcgtggggcgcaggcagcctggacggcgtcgacgggatgatcattccgggcggctttggggcgcggggcgtcg

agggcaaggtggcggcgatccggcatgcgcgcgagagcggtgttccgctgctggggatctgcctcgggtaccagcttgcg

attgtcgagatgtgcaggaacgtgctgggggtctgcgacgcgtcgtcggaggagtttgaccccgagggccgcaaccttgt

gatccggttcatcagcgacgagaacggggctgttgaccggcggctgcgg

>Hypothenemus_hampei_contig_12134|c0_g1_i1 hypothetical protein M970_080830

ctgtatcctgtgcgggcagtgctcaacgtcatcttcctggacccgcagctgctccatccagagatatcgaagacaacacc

atacacaaccgtgctctccccaccagtcaacctgctcattgcaatgtcgtacttccgcatagcaccggggcacttcgggc

ttcttctatgcttcatcggatcgttccttgtgggtctgctgctgtatgttgctgtgaggaggagaatactgcccggaatc

tcatgtctctacaccctagctgcctccgcagtcttcttcctgatgatgacagagagccttgtcgggctgacagcatacat

aggcaaggcaacgggactagggatccagttcctatccggcacgttcctttcgctgcagtccaacattgctgagatcatga

cgtgcctcgagtacagagatcg

>Hypothenemus_hampei_contig_12168|c0_g1_i1 plant senescence-associated partial

gagtaactatgactctcttaaggtagccaaatgcctcgtcatctaattagtgacgcgcatgaatggattaacgagattcc

cactgtccctatctactatctagcgaaaccacagccaagggaacgggcttggcagaatcagcggggaaagaagaccctgt

tgagcttgactctagtttgacattgtgaaaagacatatggggtgtagaataggtgggagctccggcgccagtgaaatacc

actacctttatcgtttttttacttattcaatgaagcggaactgggcttcaccgcccatcttctggcgttaaggtccttcg

cgggccgatccgggttgaagacattgtcaggtggggagtttggctggggcggcacatctgtaaaacaacaacgcaggtgt

cctaagggggactcatggagaacagaaatctccagtagaacaaaagggtaaaagtccccttgattttgattttcagtgtg

aatacaaaccatgaaagtgtggcctatcgatcctttagtccctcgaaatttgaggctagaggtgccagaaaagttaccac

agggataactggcttgtggcagccaagcgttcatagcgacgttgctttttgatccttcgatgtcggctcttcctatcata

ccgaagcagaattcggtaagcgttggattgttcacc

>Hypothenemus_hampei_contig_12168|c0_g1_i2 hypothetical protein GLYMA_13G0132002, partial

gagtaactatgactctcttaaggtagccaaatgcctcgtcatctaattagtgacgcgcatgaatggattaacgagattcc

cactgtccctatctactatctagcgaaaccacagccaagggaacgggcttggcagaatcagcggggaaagaagaccctgt

tgagcttgactctagtttgacattgtgaaaagacatagagggtgtagaataagtgggagcttcggcgccggtgaaatacc

actacctctatagtttttttacttattcaattaagcggagctggacttcatcgtccacgttctagcattaaggtctcatt

agaggctgatccgggttgaagacattgtcaggtggggagtttggctggggcggcacatctgttaaaagataacgcaggtg

tcctaagatgagctcaacgagaacagaaatctcgtgtggaacagaagggtaaaagctcgtttgattctgatttccagtac

gaatacgaaccgtgaaagcgtggcctaacgatcctttagaccttcggaatttgaagctagaggtgtcagaaaagttacca

cagggataactggcttgtggcagccaagcgttcatagcgacgttgctttttgatccttcgatgtcggctcttcctatcat

tgtgaagcagaattcaccaagtgttggattgttcacccacca

>Hypothenemus_hampei_contig_12168|c0_g1_i3 hypothetical protein BPMI_01608

gagtaactatgactctcttaaggtagccaaatgcctcgtcatctaattagtgacgcgcatgaatggattaacgagattcc

cactgtccctgtctactatccagcgaaaccacagccaagggaacgggcttggcagaatcagcggggaaagaagaccctgt

tgagcttgactctagtccgactttgtgaaatgacttgagaggtgtaggataagtgggagccgaaaggcgaaagtgaaata

ccactacttttaacgttattttacttattccgtgaatcggaggcggggctctgccccttcttttggacccaaggctcgct

tcggcggaccgatccgggcggaagacattgtcaggtggggagtttggctggggcggcacatctgttaaaagataacgcag

gtgtcctaagatgagctcaacgagaacagaaatctcgtgtggaacagaagggtaaaagctcgtttgattctgatttccag

tacgaatacgaaccgtgaaagcgtggcctaacgatcctttagaccttcggaatttgaagctagaggtgtcagaaaagtta

ccacagggataactggcttgtggcagccaagcgttcatagcgacgttgctttttgatccttcgatgtcggctcttcctat

cattgtgaagcagaattcaccaagtgttggattgttcacccacca

>Hypothenemus_hampei_contig_12168|c0_g1_i4 hypothetical protein ASPNIDRAFT_143688

gagtaactatgactctcttaaggtagccaaatgcctcgtcatctaattagtgacgcgcatgaatggattaacgagattcc

cactgtccctatctactatctagcgaaaccacagccaagggaacgggcttggcagaatcagcggggaaagaagaccctgt

tgagcttgactctagtttgacattgtgaaaagacatagagggtgtagaataagtgggagcttcggcgccggtgaaatacc

actacctctatagtttttttacttattcaattaagcggagctggactttgcggtccacgttctagttttaaggtcctttt

gggctgatccgggttgaagacattgtcaggtggggagtttggctggggcggcacatctgtaaaacaacaacgcaggtgtc

ctaagggggactcatggagaacagaaatctccagtagaacaaaagggtaaaagtccccttgattttgattttcagtgtga

atacaaaccatgaaagtgtggcctatcgatcctttagtccctcgaaatttgaggctagaggtgccagaaaagttaccaca

gggataactggcttgtggcagccaagcgttcatagcgacgttgctttttgatccttcgatgtcggctcttcctatcatac

cgaagcagaattcggtaagcgttggattgttcacc

>Hypothenemus_hampei_contig_12168|c0_g2_i1 hypothetical protein OXYTRI_13058

gagtaactatgactctcttaaggtagccaaatgcctcgtcatctaattagtgacgcgcatgaatggagcaacgagattcc

cactgtccctacggcgggcattgcgaacctacagccaagggaacgggcttgggcaatcagcggggaaagaagaccctgtt

gagcttgactctagtatgggggaagggctgtgcggcgcgatgcagcgaggtgggggcttcggcgcatgtgcaagaccact

attgcgcgcgcacagtgctttggtgtccatatggggagtttggctggggcggcacgactgctagagaccaacgcagacgt

cctaaggcaggatcagcggggagggaaaccacgcgttgagcacaagggcacaagcctgcttgaggctgcccctgttgcgg

ggcggcttgggggaaacccgggcctagagatcccgtgtcctatctggcgtgggacacgggtgacagaaaagttaccacag

ggataactggcttgtggcag

>Hypothenemus_hampei_contig_1222|c0_g1_i1 similarity to HYPOTHETICAL PROTEIN YDED_SCHPO

cgacctgaacaggctggtgctggactacctggtgcatgaagggctgggacacattgcagccgagtttgcaagggacgtgg

gcatccctttcaacacatcctcgttcctggaccacagaacagggataaggagggcaatcgaggagggcaacatcgatgtt

gcaatgtcccgcataaacgacctcaactcggaaatagtggacagcagcatcgagctgtactacttcctaatggagcagaa

ggcatgcgagcaggcacaggcaataagggaagagaacggggccatggacgagcagaaggtgtttattctgcttgaggagg

tgcttgagtttgtgcgctcggagctgtcgtcgcttgtggaggagaatccatctctggagtcgcacttcgaggaccttctt

gagtttgtggtcttcaactcgaagaaggaggccgttgtcgagaggaggaggggccttgccgagtacgtcaacaagtgcat

cctggagaagtacgaagttgcagagaacgacctgaagcgggtcctgaacggaatagtgagtggcgagaggc

>Hypothenemus_hampei_contig_12249|c0_g1_i1 DEHA2C14058p

aaccggtcaaggggttattgccattgactggatctccggtaccaagggtggttgggcctctgttatgaatgaagatgggg

aaacctcttctacttgtaaagacggttactactgttcttatgcttgtcaagctggtatgtccaagacccaatggccttct

gaacaaccttcgtctggtatttctgttggtggtttacaatgtaaaaacggtaagttatacaagtccaactcggattctga

ttatttatgtgcttggggtaaagatactgccgaatttaaatcaaacattaaaaaagatgttgctatctgtagaaccgatt

atctaggttctgaaaacatgaatattccaaccttattagaagctggttctaccgctccagtttcggttgttgattcttct

aaatactacacctggaaatccggtaaaacttccactcaatactacgttaacaatgccggtgtttctgttgaagatggttg

tatctggggtactgatggttctggtgttggtaactgggctccagttgtcttgggtgctggtattaccgatggtaaaactt

acttatccttgattccaaatccaaataataaagatactccaaactataacattaaaatcaaaggaactgatggtgctacc

attaatggtgattgttcttatgaagatggtcaatacaacggtcacggtactgacggttgtacttctaccctttcttctgg

taaagctcaatttgttttttactaaattaatccctaatgtgtttctacctataccatattaaagtttctgttatattaat

ttatctctacttaaaaatgcacctttttctgcagctctgattctctcttactctccccttttttttttaactaat

>Hypothenemus_hampei_contig_12249|c0_g2_i1 SUN-family beta-

ctcttccggatcttctggatattacggtaacttaggtcttttcgaagacccaaccgaaaaattcgaagatggtgtttacg

attgtgatgaattaccaaaagccgaaggtgttgttccaattacttggatctctggtttgaatggtggattctcttctatc

atggatcaaaatggagacacttcttctacttgtaaagacggattctactgttcttacgcttgtcaagctggtatgtccaa

gacccaatggccatctgaacaaccatctaacggtatttctgttggtggtttattatgtaagaatggtaaattatacagat

ccaacaaggatgaagattacttatgtaaatggggtgctaagaaagct

>Hypothenemus_hampei_contig_12249|c0_g3_i1 SUN-family beta-

ctcttccggatcttctggatattacggtaacttaggtcttttcgaagacccaaccgaaaaattcgaagatggtgtttacg

attgtgatgaattaccaaaagccgaaggtgttgttccaattacttggatctctggtttgaatggtggattctcttctatc

atggatcaaaatggagacacttcttctacttgtaaagacggattctactgttcttacgcttgtcaagctggtatgtctaa

gacccaatggccatctgaacaaccatctaacggtatttctgttggtggtttattatgtaagaatggtaaattatacagat

ccaacaaggatgaagattacttatgtaaatggggtgctaagaaagct

>Hypothenemus_hampei_contig_12283|c0_g1_i1 extracellular thaumatin domain

tatagttctgtctttctctttttgattcattcgaatcgtccttcttttttcttctttcttgactatcagtctttttgtct

gttgtacagacttcattctctacactccactttctctatacttaagtctcttcagacttcactatccttctttcaagttc

aagaaagaacaacacattctaaaaatcaaaatgatgttcaagtccctccctctcatggccactctcgccacggcccttcc

ctactacctgaacgccaccaccaacggcaccgctgctagcaccatcactcgtcgcgacggaggcggcggcggcggcgtca

acatcgtcaacaacctggattccactgtctacgcgtggtctgtgtctgatggagtcagtcacatgcataccctctctgcg

ggcggcggcaactaccaggaatcgtggcaaagcaacgacaacggcggcggcatctccatcaagctgtccatgtccgagga

ccagtccgacgtgctgcagttcgagtacactcagtccggcgacaccatcttctgggacatgtcctgcatcgatatgggcg

ccgactccagcttcaccaagaacggtttctccgtcgagcccagccagactagcaccgactgccccagcgtcaactgcggt

gctggcgacacggcttgcgccgaggcttacctccagcctaaggatgaccatgctacgcacggctgccctatcgatacctc

tttccagctgagcttgggtaactagattagtgttccctcacgaggaacgagcaaaaaaacaagaaccagaaaagatgcgc

tctagatgagtggctcacaggaaatgatccggatgatgtcaaacctcaaatcatcctacaatatctttcctccttccttt

atgaacatgacttgacgctacgacggaaaatggatgcaaacggaaacggaaacggaaatgggaaaattggaaaattgggc

tgtgatttgagatcacctttgtggagttgggtggaatttttttgcgaaagactttcgatttacaagagatagacttttgt

ccttcctttgttgctttctcagatatacttttttcttcac

>Hypothenemus_hampei_contig_12307|c0_g1_i1 hypothetical protein CANTEDRAFT_112847

atcgttaataatgcaattcgctaccgtcgccttcttattctccgctgttgtttctgctgctaccctcactgaaactgatg

ttgaatccaccttggtcaccatcacttcttgtggtccagaagtcactgaatgtccagctgaaacttcttctgctgctaac

accactgctgccaacgtttccacttgggaaggtgctgccaacaagcaatacgctgttggtgctgctgctttggccgctgg

tgctttattggctttgtaagtcattggtttttaatttcatttttgggttaaatattttgcatcggttttctgtgtaggtt

aatacatccggct

>Hypothenemus_hampei_contig_12307|c0_g1_i2 hypothetical protein CANTEDRAFT_112847

atcgttaataatgcaattcgctaccgtcgccttcttattctccgctgttgtttctgctgccaccttaactgaaactgatg

tccaatctactttggtcaccatcacttcttgtggtccagaagttactgactgtccagctgaaacttcttctgctgctaac

accactgctgctaacgtctccacctgggaaggtgctgctaacaagcaatacgctgttggtgctgctgctttggccgctgg

tgctttattggctttgtaagtcattggtttttaatttcatttttgggttaaatattttgcatcggttttctgtgtaggtt

aatacatccggct

>Hypothenemus_hampei_contig_12333|c0_g1_i1 3-ketoacyl thiolase

atgcccagggagtacgggttgagctctggaggagggagcccggacgcagcagactgcttgctggcgatgggggaggtttc

cgagatgctggccaggaagcatggggtgtcgcgcgaagctgcagatgcgtatgctgcagcaagccaggcgaaggcgctgg

atgcctcgaggagaggctgctttgccaaggagacgatcccgatcgatgtcgagggagagacggttggatgcgacgaggga

ataagagagacgtctgtcgaggcaatccgcaacctgaagcccgtcttcatggcagaaggggtctgcacatctgccaacag

ctcgcagctgtccgacggggcgtctgcagtgctcctgatgaagaggagcaaggcctgcgagctcgggctgccggtggctg

cagagttcgtcgactttgttgctgtgggagtcaagcctcgggacatggggcttggcccggcagttgcgatcaagaagctg

ctgcagagaaacgggctggacaaggaccaggtctcgtacttcgagatcaacgaggcatttgcatcccaggtgctttgctg

cctgtcggagcttgggatcgattgccacaaggtcaacaggtatggagggtccatagccctgggacatccgcttggggctt

ctggcgcaaggattgtctgcaccttgctgagcgtcatggagaacgagtctctggacgggtacggggtggcttccctgtgc

gtgggcggtgggcatggggttgctgccctgctcaggagatgtcgctagacgtcgaggcctacgggcggtcagtaaagaac

aga

>Hypothenemus_hampei_contig_12334|c0_g1_i1 similarity to D-ribose transport binding

tcttgaacttcttgcttgcgcggaagaagaggctgctgtcgtctgaggagaccgaccgcctgatggagctgattgcgcag

tcgatggagatggacgaggggtgtcgggagcagggtgacaatgcggagttgatcaggaagatccttgacaacgtggaggt

tgaggagatagggtctgatgagagcgagagtgtcggagatgcgattggcgagctgttttttgaggaggacagcgacggga

gcgggagccattctagcgacggaatgtgcgggtctgacgacaggagcgagcctctgtccgagaggaagttccggcggggg

gtgtttggaggccgaggagatggaaagcaggcagggccgaagaagaggatggttgggatcaaggatgcagggggctcgga

gctgccgaaagatgtggaggatggggaggatatgcttgagacaagggacttgaaggcggatgagcaaaggcttggagatg

atggcaatggggatatatctggaaaggcccgagggggctttgagaagaggttcaagcttcctgggctcggaggagagcgg

aaggccggggcggtcgaagggggctgctcggaggaatgtgaggaggagttccggtttgagattggggatctcaagagagt

gagcggggcgggcccgaatgtcaagttctttgacgaggacgggcaggagctctgagccattctgcagagatgtagagcag

taatggtt

>Hypothenemus_hampei_contig_12360|c0_g1_i1 hypothetical protein ALT_0287

tatgcacggcaaatcttcagcatgcctaaagtatattttgaccacgaccctataaccctacaagaaggaggccacatcgg

tgctcaaatcggtggcaaaaggattaagcctgatgaagaggaatttgttactggagaagttgagggggtcattatctata

gatccccagaaaccggttgagctgaactgcacgcaagatgctcatttcttgcctggagaacacgttatcttgcagcagct

agatccgactacttacgctgcgataggtatgagaagtggcaaggaagtagaatttaaggagtgaaaagttcatgtcgtgc

ttaaaataatttgagaatatgtattgctgctgcgttaaaagtacgggaaattttgtcctggattaggggcttggtcactc

aattgttagataacgacgagtcgggatttgatagttattcccgccactgggcaactgttgacctgacctttataagcccg

ctaaaaatctccgggc

>Hypothenemus_hampei_contig_12374|c0_g1_i1 Rab GTPase interacting factor Golgi membrane

ggagttcaacctgagggcggcgattctagggaggctgtctggagacgggcctctgcttgaggagctggggatcgacttcg

ggacgatccggaaggagagccggctgatcttcggggttctgcagaggacgcctgtggacttctcgtttgtcaagaacgcg

gacctttcggggccgattgtgtttgttgctctgtacacccttgggctcattctgaactataagatccactttgggtacat

atactttatctcgcttctgacggcgtcgtccacatacttcctgctgaacgtcctggacacaaagcaaatagggttcctgg

aatgctgcagcgtgcttggatactcgttcctgcccgttgttttcttttcgttcctgaacatcctgctgaggaggcttgcg

gtttggccgcggatgctgtacgggcttgggttt

>Hypothenemus_hampei_contig_12378|c0_g1_i1 bZIP transcription factor

agaaccttatgatcaatatccctccttctccgataatcttttcaattcttatcttccgcaatcgtgttcctcggattcgc

ctttcaatctacccggtgatgacttcgccggcctcgaatccttcgacacatgggccccggtaactgacggtccataccag

ccactgcctgctatttcaactccgttctacccagcaccctacacatctgccgttgaagcgcagcctagtcccttgcagaa

tgagctttctttcccggatatggatgttccgaacgaccttttcccctcgcacgagcctcccttgacatattctgaaggct

cccaatcccaaccacctcctatgtcccttccaacctcgaccacctcgtctcccaactcgtccactccgagtgatacgggt

acggctcggtccaaacccgcagtttcgcgaattgaaaagcgccaattgaacacgatggcggcgcgccgctatcgccaaag

acgagtcgaccaggtgaatcagttggaggctgagctcaagaaggtcaaggaggagcgggatgcgttgaaaatgcgcgtct

>Hypothenemus_hampei_contig_12394|c0_g1_i1 hypothetical protein EROM_010870

gcatcctggatggcaggcaggggcctccgcgccaacgagacgagttgtcgagcatgagagggaatccagacaggtacaac

ggggatagatacaatggaaagtgctctccaggagaccgaagtgaacggtgcccggcggaaagggatttgcgggatcgtcg

gaggtatagagacgaccagggcattgaggacgacagagagggaagaggcggcccttcaaacgactggagagatgacacgg

ttggctcaagccgtggaagacctcctagggactatgatccgccatgcacgaaagagatgtgcacggagatggacagatac

gactccccagacatgagggaggagtgtgtccggtactgtaaggactacaggagcaggaatagcaagagacccaacgaaaa

gagagaaaaggaggaggaggtaagcgacctgacaagcgactcgtgctatgaggaaaagaagtgattatacccgtgtatcg

aaggtttcatagagtatgagcagctggagcgcctcggggttacagatgcctgtaggcaggatgttctggc

>Hypothenemus_hampei_contig_12409|c0_g1_i1 hypothetical protein PICST_4493

ccgtgggggcctctgataatgccgacaacaaggcttctttctccaactggggccgttgtgtggatatctttgctcctggg

gtggatatcttgtctacttttgtctggtctgataccgtccaaatgtctggtacctcgatggcctctcctcatattgccgg

tttgttatcctactttttatctttatacccagatatcaactcggaatacgccaccggtgaaagtggcttactcgatcctc

aagctttgaaatctaaggttatcaagtacgctactaaaggtgtcattcaaggcctcactgacggtcaatctccaaacctt

cttgcct

>Hypothenemus_hampei_contig_12424|c1_g1_i1 translation elongation factor EF-1 alpha subunit

ccagcttgggtttcctctcttccttcttcttctttcttcattcatcctcttctaacttacctctttgctcagtacctcta

cgcgagaagccgtagtatctgagcaagttttcagaactcttctagtatcttacaaagaactacaaaagttcagcacaacc

agccaaaatgggtaaggaggacaagactcacatcaacatcgtcgttatcggccacgtcgattccggcaagtcgaccacca

ctggtcacttgatctacaagtgcggtggtatcgaccagcgtaccatcgagaagttcgagaaggaagccgccgagctcgga

aagggttccttcaagtacgcttgggttcttgacaagctgaagtccgagcgtgagcgtggtatcaccattgatattgccct

ctggaagttccagacctccaagtatgaggtcactgtcattgatgcccccggtcaccgtgacttcatcaagaacatgatca

ctggtacctcccaggccgattgcgctatcctcatcattgcctccggtactggtgagttcgaggctggtatctccaaggat

ggccagacccgtgagcacgctctgcttgctttcaccctcggtgtcaagcagctcatcgttgccctcaacaagatggacac

ctgcaagtgggctgaggaccgttacaacgaaatcgtgaaggagacctccaacttcatcaagaaggtcggctacaacccca

aggccgttcccttcgtccccatctccggtttcaacggtgacaacatgcttgagccttcccccaactgcccctggtacaag

ggttgggagaaggagggcaagtccggcaaggtcaccggtaagaccctgcttgaggccatcgacgccatcgaggcccccgt

ccgtccctccaacaagcccctccgtcttcccctccaggatgtctacaagatctccggtattggaactgtgcccgttggcc

gtgtcgagaccggtatcatctcccccggcatggtcgtcaccttcgctccctccaacgtcaccactgaagtcaagtccgtt

gagatgcaccaccagcagctcaaggagggtgtccccggtgacaacgttggtttcaacgtcaagaacgtttccgtcaagga

agtccgccgtggaaacgtcgcctccgactccaagaacgaccccgcctccggctgtgccaacttcaccgcccaggtcatcg

tccttaaccaccccggtcaggtcggcgctggttacgctcccgtcctcgactgccacactgcccacattgcttgcaagttc

gctgagctgcttgagaagattgaccgccgtaccggaaagtccgtcgagtcctctcccaagttcatcaagtctggtgatgc

tgccatcgttaagatgattccctccaagcccatgtgtgttgagtccttcaccgagtacccccctctcggtcgtttcgctg

tccgtgacatgcgtcagaccgtcgccgtcggtgtcatcaagtccgtcgacaagtccgctggtggtgccggtaaggtcacc

aaggccgcccagaaggctggcaagaaataagcgatttgcggatgaattcagaaatgccgtgatgaatttttttcttaaaa

at

>Hypothenemus_hampei_contig_12424|c1_g1_i2 translation elongation factor EF-1 alpha subunit

ccagcttgggtttcctctcttccttcttcttctttcttcattcatcctcttctaacttacctctttgctcagtacctcta

cgcgagaagccgtagtatctgagcaagttttcagaactcttctagtatcttacaaagaactacaaaagttcagcacaacc

agccaaaatgggtaaggaggacaagactcacatcaacatcgtcgttatcggccacgtcgattccggcaagtcgaccacca

ctggtcacttgatctacaagtgcggtggtatcgaccagcgtaccatcgagaagttcgagaaggaagccgccgagctcgga

aagggttccttcaagtacgcttgggttcttgacaagctcaagtccgagcgtgagcgtggtatcaccattgacattgccct

ctggaagttccagacctccaagtatgaggtcactgtcattgatgcccccggtcaccgtgacttcatcaagaacatgatca

ctggtacctcccaggccgattgcgctatcctcatcattgcctccggtactggtgagttcgaggctggtatctccaaggat

ggccagacccgtgagcacgctctgcttgctttcaccctcggtgtcaagcagctcatcgttgccctcaacaagatggacac

ctgcaagtgggctgaggaccgttacaacgaaatcgtgaaggagacctccaacttcatcaagaaggtcggcttcaacccca

aggccgttcccttcgtccccatctccggtttcaacggtgacaacatgcttgagccttccaccaactgcccctggtacaag

ggctggaagaaggagggcaagtccggtgaggtcaccggtaagactcttctggaggccatcgatgccatcgagccccccgt

ccgtcccgccaacaagcccctccgtctgcccctccaggatgtctacaagatctccggtattggcacggttcccgtcggtc

gtgtcgagactggtaagatcgttcccggcatggttgtcaccttcgctcccgccaacgtcaccactgaggtcaagtccgtt

gagatgcaccaccagcagctcaaggagggtctccccggtgacaacgttggtttcaacgtcaagaacgtctccgtcaagga

ggttcgccgtggtaacgtcgcctctgactccaagaacgaccccgcctccggctgtgcttccttcaacgcccaggtcatcg

tcctgaaccaccccggtcaggtcggtgctggttacgctcccgttctggactgccacactgcccacattgcctgcaagttc

tctgagcttctggagaagattgatcgccgtaccggtaagtccgttgaggactctcccaagttcatcaagtccggtgatgc

cgccatcgtcaagatgattccctccaagcccatgtgtgttgaggctttcactgagtacccccctctgggccgtttcgccg

tccgtgacatgcgtcaaactgtcgccgtcggtgtcatcaagtccgttgacaagtccgctggtggtgccggtaaggtcacc

aaggccgcccagaaggctggcaagaaataagcgatttcacatcatgcagagatgccgtgatgattttcttacaaaaacgc

ttatggatggagctagattttgctgtgtagtccaaaaatgctactcacaggtttat

>Hypothenemus_hampei_contig_12424|c1_g1_i3 elongation factor 1-alpha

agctgtagaatctgtgaaaataaaccgccgccatgggtaaagaagacaagactcacattaacatcgtcgttatcggccac

gtcgattccggcaagtccaccaccactggtcacttgatctacaagtgcggtggtatcgactctcgtaccatcgagaagtt

cgagaaggaagccgccgagctcggaaagggttccttcaagtacgcttgggttcttgacaagctcaagtccgagcgtgagc

gtggtatcaccattgacattgccctctggaagttccagacctccaagtatgaggtcactgtcattgatgcccccggtcac

cgtgacttcatcaagaacatgatcactggtacctcccaggccgattgcgctatcctcatcattgcctccggtactggtga

gttcgaggctggtatctccaaggatggccagacccgtgagcacgctctgcttgctttcaccctcggtgtcaagcagctca

tcgttgccctcaacaagatggacacctgcaagtgggctgaggaccgttacaacgaaatcgtgaaggagacctccaacttc

atcaagaaggtcggctacaaccccaaggccgttcccttcgtccccatctccggtttcaacggtgacaacatgcttgagcc

ttcccccaactgcccctggtacaagggttgggagaaggagggcaagtccggcaaggtcaccggtaagaccctgcttgagg

ccatcgacgccatcgaggcccccgtccgtccctccaacaagcccctccgtcttcccctccaggatgtctacaagatctcc

ggtattggaactgtgcccgttggccgtgtcgagaccggtatcatctcccccggcatggtcgtcaccttcgctccctccaa

cgtcaccactgaagtcaagtccgttgagatgcaccaccagcagctcaaggagggtgtccccggtgacaacgttggtttca

acgtcaagaacgtttccgtcaaggaagtccgccgtggaaacgtcgcctccgactccaagaacgaccccgcctccggctgt

gccaacttcaccgcccaggtcatcgtccttaaccaccccggtcaggtcggcgctggttacgctcccgtcctcgactgcca

cactgcccacattgcttgcaagttcgctgagctgcttgagaagattgaccgccgtaccggaaagtccgtcgagtcctctc

ccaagttcatcaagtctggtgatgctgccatcgttaagatgattccctccaagcccatgtgtgttgagtccttcaccgag

tacccccctctcggtcgtttcgctgtccgtgacatgcgtcagaccgtcgccgtcggtgtcatcaagtccgtcgacaagtc

cgctggtggtgccggtaaggtcaccaaggccgcccagaaggctggcaagaaataagcgatttgcggatgaattcagaaat

gccgtgatgaatttttttcttaaaaat

>Hypothenemus_hampei_contig_12424|c1_g1_i4 translation elongation factor EF-1 alpha subunit

ccagcttgggtttcctctcttccttcttcttctttcttcattcatcctcttctaacttacctctttgctcagtacctcta

cgcgagaagccgtagtatctgagcaagttttcagaactcttctagtatcttacaaagaactacaaaagttcagcacaacc

agccaaaatgggtaaggaggacaagactcacatcaacatcgtcgttatcggccacgtcgattccggcaagtcgaccacca

ctggtcacttgatctacaagtgcggtggtatcgaccagcgtaccatcgagaagttcgagaaggaagccgccgagctcgga

aagggttccttcaagtacgcttgggttcttgacaagctcaagtccgagcgtgagcgtggtatcaccattgacattgccct

ctggaagttccagacctccaagtatgaggtcactgtcattgatgcccccggtcaccgtgacttcatcaagaacatgatca

ctggtacctcccaggccgattgcgctattctcatcattgcctccggtactggtgagttcgaggctggtatctccaaggac

ggtcagacccgtgagcacgctctgcttgccttcacccttggtgtccgccagctcatcgtcgctctgaacaagatggacac

tgccaagtgggccgaggagcgttacaacgagattgtcaaggagacctccaacttcatcaagaaggtcggctacaacccca

agaccgtccccttcgtccccatctccggtttcaacggtgacaacatgcttgagccttccaccaactgcccctggtacaag

ggctggaagaaggagggcaagtccggtgaggtcaccggtaagactcttctggaggccatcgatgccatcgagccccccgt

ccgtcccgccaacaagcccctccgtctgcccctccaggatgtctacaagatctccggtattggcacagttcccgtcggtc

gtgtcgagactggtgttatctcccccggcatggtcgttaccttcgctcccgccaacgtcaccactgaggtcaagtccgtt

gagatgcaccaccagcagctcaaggagggtctccccggtgacaacgtcggtttcaacgtcaagaacgtttccgtcaagga

agtccgccgtggaaacgtcgcctccgactccaagaacgaccccgc

>Hypothenemus_hampei_contig_12424|c1_g1_i5 elongation factor 1-alpha

catcgagaagttcgagaaggaggccgctgagctcggtaagggttccttcaagtacgcttgggttcttgacaagctcaagg

ccgagcgtgagcgtggtatcaccatcgatattgccctctggaagttccagacctccaagtatgaggttaccgtcattgat

gcccccggtcaccgtgacttcatcaagaacatgatcactggtacctcccaggctgactgcgccattctcatcattgcctc

cggtactggtgagttcgaggctggtatctccaaggatggccagacccgtgagcacgctctgcttgccttcacccttggtg

tccgccagctcatcgtcgctctgaacaagatggacactgccaagtgggccgaggagcgttacaacgagattgtcaaggag

acctccaacttcatcaagaaggtcggctacaaccccaagaccgtccccttcgtccccatctccggtttcaacggtgacaa

catgcttgagccttccaccaactgcccctggtacaagggctggaagaaggagggtaagtccggtgaggtcaccggt

>Hypothenemus_hampei_contig_12612|c0_g1_i1 Brefeldin A resistance

ttggctgtattggctgaatcctaccacttatctggttggtagtatgcttactttcaccatctttgacgccgatgttgaat

gtgtcaacgaggaactctcgttatttgacccgcctagtaacttgacttgtggcgaatatctggcctcctacctcaccact

tcaggtgcgaacctcttgaacccagacgattcgctcggttgcagggtttgtccttactctaagggaagtgattacttgcg

aacactcaacatcaatgactggtattttggatggagagatgtaggtatcacggtcatctttgtcctcagttcatacgctc

tggtatatgtattcatggcactgaaaacgaagccttccaagaaggctcaatagtttctcaccacatgaaaagaacttgcc

aaagatacgctacgctgtctaaatcctcgattctgtcgctttctcccatgttttcagtttctatttctactactattctc

tgttattcgctttggtgtttgttatcagttctttctctacttcatgttcc

>Hypothenemus_hampei_contig_12620|c0_g1_i1 26S proteasome subunit alpha-4

tgagcgaacgagaagccttttcctgctctgaagggcgcggactggtgggcacaccttggaaaataaggcgattgggatag

cccctcaatggggtttgaggagcagcttgcggtgttttcgccggacgggaggctgatccaggtcgagtatgcccagcagg

caagcgagcacgggtctcttgttgtgtttgggactgggcgcgagcagatatccgtgagcatcgagaggaagtctgggaac

aagcttctaatggaggaggaaaaggtgtttctgatcagcgagccccaggggatttggatgagctactctgggttcaagcc

ggatgcctatcttgtgatgaacatggcgcgaatgatatgcttttcgtacaagaactcgactggggaggacatgacgattg

accagctggcaaggcagctgagcgagtacaagcagaggtacactgttgtgtaccaccagaggccgtttggggtgcggaca

gtgctgtttgggcttgtgccttcgccggcagcatatgtgcttgagcccgacggaaacttctctgagttcagcagtggggc

cattgggcagaagagccagaaggtgtgtgagtatcttgagaaggaggggagcgaggatcttgtgcgggggacgattcttg

ggctgatggaggttgtgcagtcggacccgaacaagatatcgtgctttgtgataacccgaggggggtgtgagcgggtgcct

gagtccacgatcatggaggtggtcaggtcgtctg

>Hypothenemus_hampei_contig_12626|c0_g1_i1 DHAL_EMENI Aldehyde dehydrogenase (ALDDH)

cgtggcccacggtggtgagcgccacggtgacgagggctacttcatccagcctaccgtcttcaccaacgtcactagcgaca

tgaagatttcgcaggaggagatcttcggacctgtcgtcaccgtccagaagttcaaggacgaggccgaggctatcaagatt

ggtaacaacaccagctacggtctggctgctgccgtccacacgaagaacgtcaacaccgccatccgtgtctccaacgccct

caaggctggtaccgtctggatcaacagctacaacatgatctcctaccaggctcccttcggtggcttcaaggagtccggtc

tgggccgtgagcttggctcctacgccctggagaactacaccc

>Hypothenemus_hampei_contig_12626|c0_g2_i1 Aldehyde dehydrogenase

cgtggcccacggtggtgagcgccacggtgacgagggctacttcatccagcctaccgtcttcaccaacgtcactagcgaca

tgaagatttcgcaggaggagatcttcggacctgtcgtcaccgtccagaagttcaaggacgaggccgaggctatcaagatt

ggcaacagcaccgcctacggtctcgccgctgccgtccacaccaagaacgtcaacactgccatccgtgtctccaacgccct

gcgcgccggaaccgtttggatcaacaactacaacctcatctcgtggcaggctcccttcggtggcttcaaggagtccggtc

tgggccgtgagcttggctcctacgccctggagaactacaccc

>Hypothenemus_hampei_contig_12664|c0_g1_i1 40S ribosomal S8

accacaagcgcaggaagacaggggcaaaggcggtgatcagctccaagaagcgcaacaacagggcggggtcgcagccaagc

gcaacgaagataggggagaagagggtgaggcctgtcagggtgcgaggaggcaacaagaagtacagggccttgaggctgga

cgtggggcatttcaagtttgcaacgacggggcacacgcgggcatcgaagcttatgcaggttgtgtaccatcccagctcga

acgagctggtgcgcaccaacacgctgaccaagagctcagttgtcaagatctctgcagagccgttttcggaggacgtcaag

agtgtggagaaggaggttgatcccgagctccatgaaggctttgagaaggggtatctatacgcaatcatcacgtccaggcc

cgggcagattggaatggcacaggggcatgttctacagggagacgagctgaagttctactcggataagttcaacaagaaga

ccaaggaggttcagaaataaacgaattggctgcttttcaaaaaaaaaga

>Hypothenemus_hampei_contig_12680|c0_g1_i1 Peroxisomal membrane

cctctccgaccccgaggccaagttctccaagtccattggctgggccgatgctgagggccgtacctaccgttacgctctcg

tcattgacaacggcaaggtcacctacgccgctaaggaggccgagaagaacgtcctcgaggtcacctccgccgaccacgtc

tacaagcagctgtaagcgtgagtggatcatcaaaacaatgcattcactttttattttagaccggcgcggcgcaatacata

ttacgggtcagcgagtgataggtaatagttcgagttgatacttgagaaaagttatctgctttgagttttgctttcaatcg

ctcctgttccttttagcttgtgtcaacatagaggaaacatatcctaatg

>Hypothenemus_hampei_contig_12705|c0_g1_i1 40S ribosomal S12

gatctgaaaaattttaccctccgatgaacgagatgcaggactcgatgatagagactgagatgactctccaggaggcgctc

accaaggtgtgcaaggtgtccaggacgtactgtaagctaagcaagggggcgaaggagacgacgaagaagatccttgcggg

gacgatgaggtttgtgatgctcacaaaggacgccgagccgcgggtcgagaagcttgtcacgctgctggcgaagaagaagg

acatcccgatcatctcgatcgagacccggcaggagctcgggcgcattgttggcgtggagaatgtcagctcgacaggcaag

gtgaggggcaaggggtgcagcgtagccgggatccaggactactgcgagcagacttccgaggcaatttttgtccaggcagc

tctgctaaggggcgtgtcggcctaataaacccgatttgagttaattgtccagttgtcaggcaaggctctcctgcgagagt

gtgattggatggaggagggacttaaaggagatgcataaggaggtttcggtagatatagaggggtctctgcaaagcgcaat

aggatggagtcgcctgatagaagg

>Hypothenemus_hampei_contig_12736|c0_g1_i1 polar tube ptp2

cgttgctcttgttagcaccacaggtgtccagcagcccccggctgttgccccccaggcacctattacagtcctcccggcac

agccccagggctctgtggtcaatgcatgcacggccaagaagctggacggcgcagagatagtgaggaggaatatgatggaa

tgccagagaaagagcgcagaggcaacacggacgatggtcgagagggcaaacgaaaaggctgtagagtcgttcaacaagga

gatcagcaagggtcccaaccagaaggaaagcggccagtgtatagaaaaggccgtgcaaggcaccgacaggtgcattcttg

cggggatcatcgacaaggcggtcaagcggcgcaagtacaggatctccgacgtcgaaaacagcacgtcactctacaggggg

gacaagctgattgccatgatcatcaacgtcgactacgggttccagccaatcaacagaccgaagaagaggaagtcgaagat

aatggccaaccttcctctgccgaagagagagatgtacttcaaccagatcgggcagcttgtgggagcaaaggggacgttcc

ctcaggagaacaaggagaactgcaagccttgcgacgacaagaagaccattgaaaccgtgtccgagaagtgcaatctcggg

tgccagcttaagggaacgtctgccctgataaacaaggccatccagaagaaggaccccaaggaaacaaaggaggctgagaa

ggaagaagcaaaccaggactctaacgacggcgaggccgagaaggccgacaaggccgaggacgcagaaggacaggcgtctg

cagacggcgagggtctagagtaattcttaaattaaaatctccctgg

>Hypothenemus_hampei_contig_12746|c0_g1_i1 COATOMER BETA PRIME SUBUNIT

gatctggggacggagcactatgtagtttcgtcgtggtgtgtgagggacgacgtgctctactttgtgtctggcggaaaggg

atactacctgatcatgggggacaggccgtatgtataccacttttcgtctgtcgaggggacgattgccggggtttccgggg

acttggtgttctacctgcacgagaaggccattgagagcaagaagatagacggcgagttccttgagttccagcgggcagtg

atagccgggagggagtgcaaggcaggcgacgggatccgcagcaaggcgattgtgttttttgagtctctggggatgcatga

gagggcgcttgagctgtgtgtggacgacaaccagaggtttgagatcctgctgaagctggacaggtacgacgaggcgtttt

cgaaggcaaacagcattgtcaagtacgacaagcttgggaagtacttcctcaaggcgggggagcttggaaaggcatccgag

tgctttttcaggtcgaggaactgggtgagcctgctgcttgcagacgtgctgtctgggaagacgcggcttgcagaagccgg

ggcgtcgtgcaggaaggaggggcggctcaaccacgcgttctttgcgtacctgaagagcgggatgtatgcggagtgcgggc

gcctgcttgagggaacgccgtttctgccgctgttttcgaggagcaagat

>Hypothenemus_hampei_contig_12756|c0_g1_i1 3-ketoacyl- thiolase peroxisomal

ttcttctaaacaatctcttttacagaaaaaccccgatgatgtagtcattgttgctgcttatagaacagcattaactaaag

ggggccgtggtaaattgaaggatgttggttctgattatgttttgaccaaatttttggagcaatttttatccaagaccaaa

gttgatgcttcattgattgaagacgttgctgttggtaatgttttaaaccgtgctgctggtgcctctgaacatcgtggtgc

atgtttggctgccggtattccaaattctgctgcttttattgctcttaatagacaatgttcttctgggttaatggctatca

gtgaaattgccaataaaatcaaatgtggggaaattgattgtggtttagctgctggggttgaatcaatgagtgctaactat

ggtccggatgctattccaaaagttgataatcatttaaaagaaaatgctgaaatggccaaatgtttaattccaatggggat

tactaacgaaaatgttgccgccaagtactctattcctagaaatgttcaagatgagtttgctgctgattcttatgctaagg

ctgccaaagccattgaatccggggcttt

>Hypothenemus_hampei_contig_12759|c0_g1_i1 eukaryotic translation initiation factor eIF2A

acgagaacgtcgtggtgtttgcagggtttgacaacctgtctggggacatcgaggtgtttgatgtagcttcgcgcaagctg

ctggccaagttcaacgtgcttggggcgtcgctggttgactggaaggaggacgggtcgcatttctacgtgtcgaccacaag

ctacttccaggaggacaacgggattgtggtgtacgactactacggaaggcgggtgtccgagaggaggtttgagtccttgg

tgtctgcccgtggatatggagagacagaagcgtttgtgtgcctggagaagcccgagaagccgatcatcgaggtgcagcag

agatatgttcccccgtctgtgcataacgctgtagcaaggcgggccggggcaaagcccttgcacaagaagggagt

>Hypothenemus_hampei_contig_12800|c0_g1_i1 Phosphoenolpyruvate carboxykinase

gctaagattccatgtcttgctgatactcacccaaccaacattattcttttaacttgtgatgcttctggtgttttaccacc

agtttctaaattgactaatgcccaagtgatgtatcattttatctctggttatacttctaaaatggctggtactgaagaag

gtgtaactgaaccccaagctactttctctgcttgttttggtcaaccatttttggttttacatccaatgaagtatgctcaa

caactttctgataaaatttctaaatacaatgccaatgcttggttattgaacactggttgggttggtgcaagtgctgctag

aggtggtaaacgttgttctcttaaatacactcgtgctattttgaatgcaattcattctggggaattgagtaaggttcaat

acgatactttcccaacttttaacttgaatattccaaccagttgtcctggtgttccaagtgaaattttaaatccaactaaa

gc

>Hypothenemus_hampei_contig_12801|c0_g1_i1 1,3-beta-glucanosyltransferase Gel1

gcggtaccgatgacgagcgctccgacttcttcgctttcaacgactactcctggtgcgacccctcctccttcaccacctct

ggctgggaccagaaggtcaagaacttcactggctacggtcttcctctgttcctttccgagtacggttgcaacaccaacac

ccgtaagttccaggaggttgaggctctttactccaccaagatgaccggtgtctactccggtggtctcgtctacgagtatt

ctgaggagggcagcaactacggtttggtcaagatctccgacaacagtgtcaagaccctgaaggactacgatgctctgaag

gaggctttcgacaagaccgccaaccccaagggcaacggtgactac

>Hypothenemus_hampei_contig_12802|c0_g1_i1 glucan endo-1,3-beta-glucosidase eglC

agacattcacttgccactttttcataatgcagatgatccagcttctcgcccttgccctctccgtggccaccgccgatgcc

gtctccaagggcttcaactacggtgccaccaaggctgatggcagcagcaagtaccaggccgacttcaagaaggatttcgc

cgccgccaaggttctggtcggtaactccggcttcaccagtgcccggctctacaccatgatccagggtggtaccaccaaca

cccccattgaggctatccctgccgccattgaggagaagaccgagctgctgctgggtctctgggcctctggtggtaacatg

gacaatgagattgctgctcttaagtccgccatcagccagtatggtgaggatttcgctaagctcg

>Hypothenemus_hampei_contig_12838|c0_g1_i1 tyrosine phosphatase

cgaggtgtacggtgtccggggccgcagcataaggaggctccggtacatcaactgggtggacttctctgtgatctccaagg

acgagatggagctgttccactcgtacttcgacagggtgcgcacggagacggttcttgtgcactgcattgccggggtcggg

aggacggggacattcatcatgtacgacatcctcaagaaaatgggcgtgccgacgctggacatgtttgtaggcgtcttcct

ggagctccggagcaagagggcacacctcgtgacaaatagagtccagctcgagttcctgaagagcgtcttcctcgacaggc

cagaggcgtcttg

>Hypothenemus_hampei_contig_12853|c0_g1_i1 sugar transporter STL1

agagtaactggggttgttaccttttctttgctgctatgaactttgcctttgttccaatcattttcttcttctacccagaa

actgccggtagacaattagaagaaatcgatatcatcttcgctaaggctcatgttgatggtagacaaccatggagagttgc

tgctaccatgcctcgtttatctttgaatgaaatcgaagaacaaggtaaccaattaggattatacgagggagacttcgaaa

agcttcaatctgaagttaaggaagacaactcctctatcgtaaagtctaatgatgaatctgacaccccaggtgaaaatact

gaaaaagctgaaggtgttttaggtaatgacagtgctaacaaggtttaatagatcg

>Hypothenemus_hampei_contig_12870|c0_g1_i1 hypothetical protein EHEL_030730

atacatatttgagatggatctccacagagggtcggaagataagaatcaagaacttcaaggacccgatcaaccaggagcaa

atccatgagatcagcttcttcaaactgaggtacgactcagacacaccgcttagggcagagtacgtcggcaaccaagcaaa

ctttctcgagaacaactgcttccgcagcaagattttttatcaggaaaatgcgctggacgcactgtccgtcaacttccagt

tcggctctccccagaaaataccaatcatcaacaggagaaagatcctcactgtactcgtgatgatcgtggccgtcctgggg

ataggaacgctggttgccatgctggcagagaaggattctccaagggcccctcccccgctcaagtttcgcctaagcggaag

aaaaagcat

>Hypothenemus_hampei_contig_12870|c0_g2_i1 hypothetical protein EHEL_030730

aaggcaaacgggtcccgctgtgtgcttgaggccgtcaaagtcgagccgttcacacagaggaagtttgcagacatgataaa

ggagtcccatgcgagaacacaggactattacctcgcaagggtccagtgcaaggatggcaatggggatgggaagaacacgt

actactgctacgacgcaagacagctctgtaaatacatatttgagatggtgatctccacagagggtcggaagataagaatc

aagaacttcaaggacccgatcaaccaggagcaaatccatgagatcagcttcttcaaactgaggtacgactcagacacacc

gcttagggcagagtacgtcggcaaccaagcaaactttctcgagaacaactgcttccgcagcaagattttttatcaggaaa

atgcgctggacgcactgtccgtcaacttccagttcggctctccccagaaaataccaatcatcaacaggagaaagatcctc

actgtactcgtgatgatcgtggccgtcctggggataggaacgctggttgccatgctggcagagaaggattctccaagggc

ccctcccccgctcaagtttcgcctaagcggaagaaaaagcat

>Hypothenemus_hampei_contig_12875|c0_g1_i1 PI3 PI4 kinase

gagaagtatactaggaacctaaggcagaacagaatcacgtgcctggggtgcgacggaaagggagaaggcatcgaggcatg

tacatacagatatgagatttcaaagcagcagaggtacgctgcatacgaaagggtttacaacacgttcaagacagagaggt

atgtggacaggtactttgggctggcctatggaagccttggaaagtactttcggttcaaaagccgtgttctttcgagctac

tccaccaacagcgcctttctatacatgctttctgttgtggacaggaggcccgggaacattgttgtagcacgggacacggg

atacttcatgaacaggactgtctgttgcgaggagggagaaggcccaggccgagcaaagggagtcggggggattataaccc

cgggcatgcaaaagctgtttggaaaggaggggatagaggggataatggtctcgatcatgtaccactatgcagacatgctc

aattcgggggattggcataaagacctgttgagggttatactggagggcaggtttgcaggcatggcggggtctgggctcaa

ggggatgcacagagaagtgttgagcaggatttctgggatggttggaaagggagacgacgggggttacagtataatcccga

tggtcagtgagtggatggacgtgttcaagctggcacaagccgacccaagaaacgt

>Hypothenemus_hampei_contig_12902|c0_g1_i1 60S ribosomal L25

aggatcaatattaaccaaagacaagatggctccaaactctaaagctactaatgctaaaaaatcagtcctcaaaggtacta

acgccaaaaaggctttaaaagttagaaccgatactactttccacttaccaaaaactttaaaattagctagatccccaaaa

tacgctagaacttcagttcctcactatactagattagatgcttacaaaattattgtttctccaatcgccactgaaactgc

tattaaaaaagttgaagatggtaacattttagttttccaagttgacttaaaagctaacaaacatcaaatcaaatctgccg

tcaaagaattatacgatgttgatgttgaatacgttaatactttagtcagaccaaacggtaccaaaaaagcttacatcaga

ttaaccgctgaccacgatgctttagatgttgccaacagaattggttacatttgatt

>Hypothenemus_hampei_contig_12902|c0_g2_i1 60S ribosomal L25

aggatcaatattaaccaaagacaagatggctccaaactctaaagctactaatgctaaaaaatcagtcctcaaaggtacta

acgccaaaaaggctttaaaagttagaaccgatactactttccacttaccaaaaactttaaaattagctagatccccaaaa

tacgctagaacttcagttcctcactatactagattagatgcttacaaaattattgtttctccaatcgccactgaaactgc

tattaaaaaaagttgaagatggtaacattttagttttccaagttgacttaaaagctaacaaacatcaaatcaaatctgcc

gtcaaagaattatacgatgttgatgttgaatacgttaatactttagtcagaccaaacggtaccaaaaaagcttacatcag

attaaccgctgaccacgatgctttagatgttgccaacagaattggttacatttgatt

>Hypothenemus_hampei_contig_12912|c0_g1_i1 translation elongation factor 2

agattgccgtcaagctgccgtcgcctctgcagagccagaagttccggtacgactatctgtacgaggggcctgtggaggac

agcgttgcaacggcaatcaaggcatgtgatgccggggaggatgcgcctgtgacgatgtatgtgtcgaagatgattccttc

gaacgacaacaggttcattgcgtttggaagggtgttttccgggaagatttatcctgggatgaagatccgtgtgcaggagc

cggggtactcgccgacgtctgaggagctgtcgaacacgtcgctgatccacacgaagtctgttttgaggactgttgtgatg

atggggcgcgggtacaaggatgtgccgaactgcccggcaggaaacatcatagggattgttgggatcgacgactgcctgaa

gaagacggggacgatcacgaacaaggagggggcatacaacatcaagtcgatgaagttctctgtgtctcctgttgtcaagg

tggcggtgtctgcaaagaggcctgaggatcttgggaagctccaggaggggctgaacaagcttgcacagtcggaccccttg

tgcctggtcgagagaaacgacaagggccagaacacgattgcatgtgcagggtcgcttcaccttgagatctgcctcaagga

ccttgaggagcagtatgccaaggtgccgatcgttgcagacgacccactggtgacgtacttcgagggcgtgacgtcgtctg

tctccgagtcgaagatgacgaagtctgccaacaagcacaacaggatctacatgactgccgagccgctcgacgagaagatt

gtcgacaacctgaaggacgtcaagtccgaccaggccaagacgatggcaacgaacttccgtgagaagctggacatccggga

cgactggatcaagaagatatggtgctatgcgcccgagttgaacccgcagaacctgctcgtggatgggaccaaggggatct

cgatcatcaacgagatcaaggagcacgtcaacacggggttccgggctgcagtcaacgacgggccattgatcggcgaggtc

atgcgcggggtcaagttcgagctgagggacgcggtcctgcacgcagacgcaatccaccgaggaatcaaccagctgctgca

gccggtcaagaacctctgcaaggggctcctgcttgcagccggccccgtgctctatgagccgatatacgaggtcgagatca

ccacgcccaacgactactcgggagccgtgacgacgatcctgctttcgaagagggggtctgccgaggacttcaagacgctt

cctggaaacgacacgaccatgatcacggggacgctgcccgtgaaggagtcgtttacgttcaacgaggacctcaagtccgg

gtccaggggaaaggccagtgcgtcgatgaggttctcccactacagcatcctgcctgggagcatggaggacccaaactcct

tgatgttcaagaccgtcgagacagtcaggaagctcaagaagatgaatcctgctcctccgactgcagactcgttcttcgat

a

>Hypothenemus_hampei_contig_12932|c0_g1_i1 basic 7S globulin-like

cgaggtgtgcgtcagctcaaagaacgttttcagcacacgattgggtccatcggttccccaaattgatcttgttttgcaga

gtcaaagcgtggtttggagcgtcactggctcaaactccatggtgtacgtgaacgataatgtattgtgccttggattcgtg

aatggtggagtgaatcccaggacctcaattgtgatcggcgggtaccaattggaggacttgctcttgcaattcgatttggc

tacttcaagactgggcttcacctctacgcttttgggctctcgaaccacatgcgcaaactttaacttcacctccacagcct

aaactctctgtcaaagagtggattgcttctcaagtcacaactcatgctgctgctgctgctttttgtgtgtgtctgtctat

acatgattacttttgttgttatttagcaccgaataatggccttatggtcggtgtctttggattactatactattccttgg

aataaaag

>Hypothenemus_hampei_contig_12939|c0_g1_i1 hypothetical protein

gatctgataatccgggaggacacgttcaagctgtttacaaacaggtctgggtccgggatattcgagaggcttgaggagca

gccgcggatggagacgatggaggacttggaggcgcggatcaaggggtacttccgcgggattcttgacacgagcgcagcgg

actcgctgtacttcagcgaggcgtcgcggatgctcagctttgtggagcgagttttcaaggagcttcgggagctcaacgag

tctgcgatggagaagagcataaggagcccgatggacgatgtgatccggctctactcggagtccctgcgcagatttgttga

gggcatggacattcctgttctgcaggagcacgtgaggttcattgtgtcgtaaagacaggcgcaaataaacag

>Hypothenemus_hampei_contig_12955|c0_g1_i1 similarity to HYPOTHETICAL PROTEIN YXIF_BACSU

gtgccatgcgggccctcaggggcctcacaagcctgtctaccttgcccaccaggtcgagccttctgtatatctcatccgag

cgtccggggctgcccacgatcttctccatctggaatagctgtgcggcactgccaaagtcggacgggtcgatgcagggaag

ggcctcgaggccgctgcctgtgtatgtcaggcaccggtcctcatatgacacaacggtccccagcggcctgtcgtatatgt

cccagtggaactcgtctgccccaatggctagctcgtcgtttgcagagagatacgagtttcctattgcaattgcatatcgg

ccgccgctctcctcaatgcttgtgttgaccaggggaatgcccttgaccctcaggtccaggtccgggcgaagagacagctt

gatctcaacaagatactttgtatg

>Hypothenemus_hampei_contig_12966|c0_g1_i1 hypothetical protein ECU04_0770

gacaaggtacatcgcaatgatgtatggatacatccacccggagcggtggaaagagatttacaaggaggtgctctgtgcat

acaaggagcatgggtattcgtcgcccgtggacttctgcgaggacctcaggtttgatgcagcaaggaggctgtacgtgcag

aaggtcgagctaaggaccaggctcttggagggggaggacgtggagtaccccgaggagcatcccaagcacaactggaacat

cggggacatcgatagaatatccaggcttgggaaaggctcctccgagccggccttggacaccaggcagctgtatcccaccg

tagacccacgggtcatcatacagcatgtggaggccatgaacaggcagcaagcagacaccgcgctgctgggtgcacaaaac

aaggtgcttgaagccagagaggccctttcaatgggcacagagaaggagaggaagaggttctatacccagatgtcggacga

ggagagggagatcgacgacttcctggatagaatggaggaggaggactacggcg

>Hypothenemus_hampei_contig_12969|c0_g1_i1 M2 dsRNA satellite propagation

tccggggttctacgagaacctgtttatgaataaggtttcttgcgacgtgctgcaaatcatggggaggatcaaggggatag

acaaggagtcacagatgactaaaacggttgagtctgtgctggcgagaatgaaggatgggaaggggctccgggacggggct

ggcttacaagggttgtttatgaacattgtgtgtccaggcatggttctccccaagggtgcggatccgtctgttggggttgt

gctcctgacatctatccaacttggggagcttgaggcgcccttctgccagaaaatactgtgtctgcatggacaagcagccg

gcaaagagcatcttgagatctgccacgagaccttgaagtactatgggagtgtgaccagggtgtttaactatgttaaagag

atgaaggaagtgtacgagacaacggtgggaagagatctgccggggctgtttttgcagcctccaacgctcttcagctcatg

cttccacgaggcgtttggattttctggagtgacggggaagagtaaccgagggtttctgaggaacgtgctggagctgctag

tagcaaacagaaggctttatccggccattcctgggattat

>Hypothenemus_hampei_contig_12987|c0_g1_i1 DNA-directed RNA polymerase subunit beta

gtgctaaggatgaagaacctaaactacaacaagctggacgaggacgggctcataagccccggaacaagggtcactgggga

cgatgtgctcattgggaagataacaccgatattggatccggagagaagtaccaaggaagcaccggtttatgtgtacaagg

acagtagcacggccatgcgtaggaccgagacggggattgttgacacagccatcatcacgaacaaagatggatacaggttt

tcgaaggttaaggtgaggtctgggcgggttcctcagatgggagacaagtttgcgtcaagacatgcacagaagggaacgat

aggaatcacgctgaggcaagaagatatgccgtttactgcagacggaatagttccggatataattatcaatccgcatgcaa

ttcccagtcgtatgacgatagggcatctgatcgagtgtcttcttggcaaggtcagtgcgatgagcggggaggagggagat

gcaactccgttcagcgacgtgactgttaacga

>Hypothenemus_hampei_contig_13007|c0_g1_i1 hypothetical protein EHEL_081420

cctggccggggtctctgcgccgctgtatgcaagcaggaagacagtggatctgtttgtctacaagacaatggtcgtcacga

tctcgtcgatctactcgtgtgcttgggacgtgttcatggactggggcattgtcagggacaagatggtctaccctcgccac

acatatgcctggggcgttgtgttcaacgtggcgtgcaggtttgcgtgggtggtggccgataggttcaaggtccctgcgtt

ctggatggcgtttctagagatcaccaggaggtttgtgtggactgtgttccgcgtggagttcgagcacctcaacaactgca

gcgagttcaagtcgaagggctcgatgcgcctgacgtccagggagctcttctacaagaaggactacgaggcaaacacaagg

cccaacgacaccgagacagagaacgactcgagcgttgcatagcaggccc

>Hypothenemus_hampei_contig_13022|c0_g1_i1 predicted protein

ccccccggtgcaactacaccttcccgtccgcgtcggacggcgagacctgggtgatgaacgcgctcaagttggagcaaagc

atcaccggcgccttgatcggcctgtcgggctacactcagtcgcccgaggtttccttcctgctggcgcgtctgtcggccga

gcacagcggccacgcctactatatcgccagccagcagcagggcaccgtcttcccgaccaacagctcctcgttggtccctg

cgtacaaccccgactgggtcctgcagaatggaacccagcccggaaagctgggctcgtacctcaagaactgcgtctcgcct

ccctcgtcgccatgcggtgagaagctgaacatcgggcctctgatcggcactttcggatcgaactcaactactaacagcac

caccaactccactgcctctgcctcgccttcggctgggcttttcgcaaaggtgttctagagaagttctagggttctttggc

ccatgaaatcgagcgccgctgccttgcatgcaataaagtcaagatcttacggcacataaagg

>Hypothenemus_hampei_contig_13099|c0_g1_i1 60S acidic ribosomal P2

gcacagcaacagcgagaggatatgcctgccgcacattgctgcaaggcttggggaggagcttccggggtacaaggtggttg

tgtcgaccagggacagggatcctgtgacgatagtgtagggccttgtgggcctgggacaggcgacgatattttgggaaaat

ttttatccccgaagatggaacatgttgctgcttacatcatgcttaacaaggttgggcgagaggccaatgcggacgggatg

agggcgcttttcaaggcgatagacgcagaggtcgaggacgagacgatggacctgtttctgtccaaggtttctggcaagag

catggaggaggtgatggcgaagggcggcgagctgatggcgtcgcttgcggtttcctctgggccggccgtgtccgaggcag

ctgcgccgacccaggccgcagagagcaagaacgaggagaaggaagaggacgaggacttcgacatctttgctgcgttttaa

ttaaaatgacct

>Hypothenemus_hampei_contig_13103|c0_g1_i1 nucleosome binding (Nhp6a)

tggtctgtccgcgtacatgttcttcgccaacaacaaccgtgacaaggtccgcgaggagaaccctggtatctctttcggcc

aggtcggcaagatgcttggcgacaagtggaaggccctttccgagtctgagcgcaagccctacgatgagaaggctgccgct

gacaagaagcgttacgaggaagagaaggccaagtaccaagctcagcaaaatgaggaggatgatgaggagtcatcttaagc

gcctagccgcaatatccaattgcgcggttcatggcgtttgtgtctttaccttgttttcgctgatctctggttctcgtcgt

tactgcatcacttaagggctttatggtatggttctacggtggtttctttctgatttttatgaacaccccccgggaaggat

ggggaggtattgtttaatagggaatgtctaccgaaacttcgggcttcgtcctgccttgtcaactgtttcatttcgtgttg

accaaagctgcactgaagccgggtggccgg

>Hypothenemus_hampei_contig_13141|c0_g1_i1 hsp75

gctgaccaccaaactaccgttcaattcccagtctaccaaggtgaaagagttaactgtaccgaaaacaccttattgggtga

attcgacttaaagaacatcccaccaatggctgctggtgaaccagttttggaatctatcttcgaagtcgatgctaacggta

tcttgaaggttactgctgttgaaaagtccaccggtagatctgctaacatcaccatctccaactctattggtagattatct

tctgaagaaatcgaaaagatgattgaagatgccgaaaagttcaagtcttctgacgatgctttcgccaagagacacgaaca

aaagcaaaaattggaagcttacgttgcctccgttgaatccaccgtcactgacccagtcttgtctgctaaaatgaagaaat

ctgctaaggacaagatcgaatctgctttgtctgaagctttagctgctttggaaatcgaagattcttctgctgatgactac

agaaaggctgaattgac

>Hypothenemus_hampei_contig_13160|c0_g1_i1 40S ribosomal S29

cttgccgtgtctgctcccaccgcgctggtctgatccgcaagtacggtatgaacatctgccgtcagtgcttccgcgagaag

tccgccgacatcggtttcaacaagtaccgttaaacaaaaataaaaccaatgtccacctttgacgccctctcgatccattc

gcgttccacaatccgttcaacgtcccctggcactcgatagaggtgcggggtgggagaaatgtttcgaatcgccgggaaat

tgactgcgcccgcgttccgttttttcgcatgacggctcccaaaaatcttctctttacgtattttttttgcttggtgtctc

gggtgtgaatctgatggacgagatggggactggggttgttgtgtgatgagtttttgaacgaggatggatggaacctgcat

ccttttatttcctttaacc

>Hypothenemus_hampei_contig_13195|c0_g1_i1 60S ribosomal L21-A

cggttacagatcccgtactcgttacgccttccaacgtgatttcaaaaaacatggtgtgattccattatccacctacttga

agacttacaaggttggtgatatcgttgacatcaaagtcaatggtgctgtccaaaagggtatgccacacaaattttaccac

ggtaaaactggtattgtctacaacgtcaccaaatcttccgttggtatcatcatcaacaaggttgttggaaacagatacat

agaaaagagagttaacattagagttgaacacatcaagcactctgcttgtcgtcaagaattcttaaacagagttaaaacta

acgctgctttaaagagagaagctaaagctaaaggtgaacaagttta

>Hypothenemus_hampei_contig_13195|c0_g2_i1 60S ribosomal L21-A

tatattgaaaaatgggtaaatcacacggttacagatctggtactcgttacgctttccaacgtgacttcaaaaagcacggt

actattgctttatccacctacttaaagacttacaaagttggtgatattgttgatattaaagctaatggtgctgtccaaaa

gggtatgcctcacaaatactaccacggtaaaactggtatcgtttacaacattaccaaatcttccgtcggtgttatcatta

acaaagttgtcggtaacagatacattgaaaaaagagttaacttaagaattgaacacgttaaacactctgcttgtcgtcaa

gaattcttaaacagagttaaaactaacgctgctttaaagagagaagctaaagctaaaggtgaacaagtttacttaaaaag

acaaccagctaaaccaagagaagctagagttatttctactgaaggtaatgttccacaaactttagccccagttccttacg

aaactttcatttaatctatttggatgtattttattggaaaattgt

>Hypothenemus_hampei_contig_13220|c0_g1_i1 lac partial

ggactcggtaatggcgcgcattgcgcccagcgccatctgatcgttggcaaccagcatcgcagtgggaacgatgccctcat

tcagcatttgcatggtttgttgaaaaccggacatggcactccagtcgccttcccgttccgctatcggctgaatttgattg

cgagtgagatatttatgccagccagccagacgcagacgcgccgagacagaacttaatgggcccgctaacagcgcgatttg

ctggtgacccaatgcgaccagatgctccacgcccagtcgcgtaccgtcttcatgggagaaaataatactgttgatgggtg

tctggtcagagaca

>Hypothenemus_hampei_contig_13227|c0_g1_i1 elongation factor 1-gamma

aggttgactataaatataatgacgaattaaccttgacctttatgtctaacaacttggttggtggtttctttgctagatta

tctgcctccaccaaattcatgtttggttccatggttgttttcggtgaaaacaacaacaacggtatcaccgggttcttttt

aatcagaggtcaagatcatgttcctgcctttgatgttgctccagattgggaatcctatgatttcaaaaaattggacgcta

aaaacgatgaagatgttaaattcatcaacaacatgttggcctgggatgaaccagttgtcgtcaacggtgaaaagaaagaa

atcgccga

>Hypothenemus_hampei_contig_13253|c0_g1_i1 Pyruvate dehydrogenase E1 component subunit mitochondrial

ttatatatgagtggtggtaaacaaccatgtaacattacttttagaggtccaaatggtgctgccgctggtgttgctgctca

acattctcaagattacgcatcttggtacggttcaattccaggtttaaaggttgtttctccatactctgctgaggattata

aagggttaattaaagctgccatcagagatccaaatccagttgttttcttggaaaatgatcgcttatggtgaatcttttga

agtgagtgaagaagctttaagtagtgattacgtcttgcctttcggtaaagctaagattgaaagagaaggtaccgatatca

ccatg

>Hypothenemus_hampei_contig_13261|c0_g1_i1 endo-1,3-beta-glucanase eglC

tggacaacgagattgctgctctgaagtctgctatcagccagtatggtgatgacttcgccaagctggtcgttggtatctcc

gtcggcagtgaggatatgtaccgtaactccgtcactggctccaagtccaacgccggtcctggtgttgagcccgaggagct

cgtctcctacatccagcaggttcgctccactatctctggcaccggcctgagtgatgcttccatcggtcacgtcgatacct

gggactcttggaccaactcctccaacgccgatgttgtcaaccacctcgactggctcggtttcgacggatacccttactac

cagctgaccatggagaacggtatcgaaaacgccaagaagctcttcgatgagtccgttgagaagac

>Hypothenemus_hampei_contig_13274|c0_g1_i1 alpha,alpha-trehalose-phosphate synthase

taaagagaggcgagaatggctttgagtacacaagaacaagcggggggttggtgactgggctcaagagcatcaacgacaag

atcaggttcaagtggctcggaaacataagcggggtcgggttgaacgaggaggagaaggaggtgataaggaaggactgctg

ggaaaggttccactcgatccctgtgttcatcgaccctgtgctgaacgacaacagctacaacgggttctgcaacgccatcc

tgtggccgatgatccactcgttcaaggacgacgtttcattcaccatcaagagctacgaggcatacatggagtacaacagg

atcttctgcgaaaaggtgtgtgagattgtcgaggacggggacattgtgtgggtccacgactaccacctgatggtgctccc

cgagatgctgaggaggaggtcggacaagcagttcaagatcatgtttttcctacacactccgtttgcgcccgcagagatca

tggaggcacttacctgcaggcgggagatagcaagtgggatgatgcacagcgacctcgttgcattccactcgttcgagtat

gcactcaacttccaggagacttgtgttgccaaccagatagagataagtgccaagctggatgcaattcccatcggaatcga

cccggagatgttcagagacgtggtcagggaggagaagacggccaagaggatcagggagctcaaggagacgttcaagggca

agaagatcctcctgggggttgaccgaacagactacgtaaaggggatgccgcaccgcgtcaagggcttccagaggtttctt

gagaagcacccggagtttgttgggaaggttgtgttcctgcaggtcggggttcctagtaggacaaatgtcaaggagtactc

ttcgtacatcacaaagatgaacgagcttgtgtccgaggcaaacagcacgatagggccggttgaggccatgcacttctact

tcctgaacaagagcgtggcctttgacgagctgtgtgcaatgtacgcggccagtgatgtgctgcttgtgacgtcgctcaag

gacgggatgaaccttgttgcgctggagtatgtttcatgccaagacgagaattgcggcgtccttgttctgagcgagtgtgc

cggggcatccacaacactgcccggggcactagagatgaactcgtggaacactgaggagattgcagatattatccacgatg

caattgcgatgactcctgaggagagggcagagaggcacgatattaacagaaaggctgttgacacattcacttctgtccga

tgggccgagagaaacctcgacggactc

>Hypothenemus_hampei_contig_13284|c0_g1_i1 similarity to HYPOTHETICAL GTP-BINDING PROTEIN YN8U_yeast

caagctcggaaggtgagtgaggcgaaggtgaaggtgcggaaggagtacgtgaagggccaaagccacaggatctggctcga

gctatacaaagtgctagacagtagcgacgttatcatccatgttctcgatgcaagggacccgctggggacaatgtgcgaga

aggtagccagctacatcaaggaggaggcgcctcacaagcatctgatgtacgtgttgaataaggtggatcttgttccaaca

ggagtgactgcaaagtggttgcgatacttgtcaaggacacacccgaccatagcataccactcgagctcgataacaaacaa

ctatgggaaggcgaatctgataaacctgcttcggcagctgagcaagctctacaagaaaagccatttgagcgttggattcg

ttgggtatccgaacatagggaagagcagcataatcaacacgctgcggagcaaggcggtgtgcaaggttgcgcctgttccg

ggggaaaccaaggtatggcagtacatagccctcacaagaggaatctacctcatcgactgccccgggattgtcccgatctc

cgactacgaccaggccgtgctccgaggagcggtccggattgagaatatcgaggagcccgaggagtatgtggacatgatag

ttgaaaaggctcgggactcgatggcaaagacatacgggatcagcttcaacagctccgaagacctgcttgaaaggcttgca

gtcaagtttggcaagctgcagaaggggggagagcctaacataaatgcagtctcgaagatggttcttcacgactggataag

gggaaagatcccctactttgtgcccccgcaagcggaggatgacgagcaaaaagaatagacaagcaatcttggtgtcaagg

caaggaagatcg

>Hypothenemus_hampei_contig_13297|c0_g1_i1 -like pseudouridine synthase

cgggggctccggagcaaggggcttgtggggaaccttgagaagggctttctgaactactttgggcagcagaggtttggaaa

caagctggccaaccacgaggtagggcgcgacatgctggagaggaactatacaggagccgtggacaagatcatgcaggccg

ggaggtgttatgggctgtatggtgccgggaagtatgaggaggccatgtgccttggagacagcacggagaagtacatcctg

aggtgcaaggccaagggaatgggggacaaggacataatctacgggctgcggcgggagctccggatgctctatctgcactc

gtaccagggcctgaagttcaacgacgccatcaactgcaggctggaacgcgggatggaggttgtcgagggagacatcgtgt

gtgtggacggggagatcaaggtcgtggaatgcccggagggcctcagcctgttcgacgtggtactgccgcttgagaggctg

gacagcaagatgctgaagggagggtatagg

>Hypothenemus_hampei_contig_13308|c0_g1_i1 transcription initiation factor TFIID subunit TAF9

tacagctgctggagttcgcctacaagtacaccacagacgttcttgaagatgcgctgctatttgcaaagcacacaggaagg

gcacagattaacacaagcgacgtgaagctcgccttgcagacaaaggtcgggaggcacttcgtccctcctcccccaagaca

gtacctgacagagatagccatgacagtgaactctaagccactaactattcccgacggggaaaacctcatcagggttccgc

ctccatcatccgcactgctaaaccttgattacgaggttctccgcaaggacagtgacaagaaaagaagaatttactaagat

ccagaggccgataacctgctaattaaacatccgctcatgccccggtcggatgttcctggactttgcgctctgaaaacagg

ccgtatagggactcccttgcagacttttgatcatgtccaagcagattgatatatgcctca

>Hypothenemus_hampei_contig_13320|c0_g1_i1 Piso0_001894

ggttctattgttgctaacagacaattccaattcgatggtccaccacctcaagctggtgctatctatgctgctggttggtc

tttagttccagtcgattacactgaagattctgaaactaaagataaagctgatgatggtaaaaaagatgataaatctaaga

gagaagaaggtaatggttacaaattagccttgggtaaacaaaccgttttctacaagtgtgcttctggtgacttttacaac

ttatatgatgaatcaattggtgatcaatgttctgaaattgaaattgatatcttgaagattgtcgattgttaaatttcaaa

ctaaatcacttttcatgaactttttctttctatgagttttttttatcaaaggttttcttattaaaggaacaaaattgtct

ggttttgtacctgaataattttcatttaggattatttt

>Hypothenemus_hampei_contig_13335|c0_g1_i1 PTP3_ENCCU ame: Full=Polar tube 3 Flags: Precursor

cgaacctcgacgacttgaacaggaagtacgaggaggaggttgcgcaccagtcgaacatcgaggcgctgctgagggagact

gcaacgtcgaacctggatgtcaaggaggtgattccgccaacaaccagccggatcgggattgtcggggggcacatgaagct

tccgctgaagggcttcagcaacgaggaggcgaagaccgaggaggaggcgattgtgcagagcaggcagaagaacaggagga

accaggtccgcaacaaggtcattgggacgtctgttgagagcgggctgaatgtgattgagacggagaacggatacacacac

gtctccgaggcggccaaggcgctgaagatcaacaaaggacgggtgttctacccggagaatgcaaaggggctgatggagaa

gcagaagctggacacgtcgctgctggagcacaggttcaacactggggagaggc

>Hypothenemus_hampei_contig_13343|c0_g1_i1 hypothetical protein CANTEDRAFT_115146

agaagtcgataaccaattgaacaaggctcaagattcttggtctaagactaagattttacctgaaaccgacaagaacccag

ttagatctggggtcagttcttaccttggatacgtcaaccatcttggtgatgtgatttctggtaaggccaaggaagctgaa

aaggaggctacaagaaagcaagagaaatatcaaaactcttggtttagttggggtactggccataaggaaagcttagaaaa

agcatacgatgttaataaggccagagccattaagaaatatcaattggctaaggaccaattggaagaagccaagaatgaat

ggtctaacgctcaaagaaagacccccgatgttgaaactagattgactgatgcacaaaaagattttgaaagttccatgaag

catttgaaatcttacggacaagaattcttcgatcaagccaacaaggacttggaagcttctaagaaatcctggtttaactg

gggtctgaagcaacaagataagttagaagc

>Hypothenemus_hampei_contig_13351|c0_g1_i1 Triosephosphate isomerase

cacgctttcctttcaatttttcagatacccccttcttaaaaatcaatcatcatgcctcgtcaattcttcgtcggtggtaa

cttcaagatgaacggtgttgtcaagagcatcaccgacatcgtgaaaaacctcaactctgctaagctcgactccaacaccg

aggttgtcatctctcctcccgccctgtacctcaccctcacccgcagcctggccgacaagaagatcggcgttgctgcccag

aacgtcttcgacaagcccaacggtgctttcactggtgaaatcagcgttgagcagctgaaggacatcgatatcaactgggt

tgtcattggtcacagcgagcgccgtgtcatcctcaaggagtccga

>Hypothenemus_hampei_contig_13379|c0_g1_i1 stearoyl- desaturase

gtctcgttgccatcgctggagtcgtccacgacgtcactgatttcatcaaggaccaccctggtggtaaggctatgattagc

tctggtattggcaaggacgccactgccatgtttaacggtggtatttactaccactccaacgccgcccacaaccttttgtc

caccatgcgtgttggtgtgattcgtggcggctgcgaggtcgagatctggaagcgttcccacaaggagaactccgagtacg

ttcgtgatgagagtggccagcgtattatccgtgctggtgctcaagtgaccaagattcctgagcccgtccccactgcggat

gctgcttgatttcggttctgtctatgattctgttt

>Hypothenemus_hampei_contig_13379|c0_g2_i1 stearoyl- desaturase

gtctcgttgccatcgctggagtcgtccacgacgtcactgatttcatcaaggaccaccctggtggtaaggctatgattagc

tctggtattggcaaggacgccactgccatgtttaacggtggtatttactaccactccaacgccgcccacaaccttttgtc

caccatgcgtgttggtgtgattcgtggcggctgcgaggtcgagatctggaagcgttcccacaaggagaactccgagtacg

ttcgtgatgagagtggtcagcgtatcattcgtgccggtgctcaagtaaccaagattcctgagcccgtccctactgcggat

gctgcttgatttcggttctgtctatgattctgttt

>Hypothenemus_hampei_contig_13391|c0_g1_i1 AAA+ ATPase

tcgatcatcttcattgacgagatcgacgcccttgcgcccaaaagggagaagtcgcaaggggaggttgagagacggatcgt

gagccagctgctgacgctcatggacgggatgaaagcaaggagcaacgtgattgttctgggagcaaccaacaggcccaact

cgattgatccggcgctcaggagatatgggaggtttgacagggagattgagatcggggttcctggggagatgggaagactt

gagatcctacggatccacacgaagaacatgaagatggccgaggacgtggacttggtggcgataaacaaggagctgcatgg

attcacggggagtgacctggcgtcgctgtgcagcgaggcggcgctgcagcaaatccgggagaagctgccccagatagacc

tggactgtgacaagatcgatgcaaaggtgctggctgctctgaaggtcagcaacgacaacttcaagtatgccattgagcat

acggatccgagctctcttagagagactgttatccagtcgccgaacgttaagtggtccgacattggggggcttgagcatgt

caagctggagcttcgggagactgtgcagtatcccgtggagtatcccgagaagttcctgaagtttgggatgacaccggcca

agggagttctgttctacgggcctcctgggtgtggtaagacccttctcgccaaggcggtggcaacggaatgcaaggccaac

ttcatatctataaagggcccggagcttctttccatgtgggttggagagtcggagtcgaacatcagagacctgtttgcacg

tgcaaggggagctgcgccctgtgtcctgttctttgacgaggtcgactcgatagccaaggcaaggtctggaaacgatggta

gtagcggggtcacggataggatgctgaatcaattgctgtcggaaatggacggaataaaccagaagaagaacgtgtttgtg

attggggcgacaaacaggcctgaccagctggacagtgctctcatgaggcctgggaggcttgaccaactggtctacatccc

gctgcctgatcttgagagcagggtgtccatcttgcaggccacgctgaagaagacgcccctctcgccggaaattgatctgc

ggcagcttgcagaggccacggacaagttttcgggggccgatctctctgagatctgccagcgggcatgcaagcttgcgata

agggaaacgattgagtacgagcttgagcagaagaagaagggatctgagatgatggatctcgaggaccctgttccgtacct

gaggcctgaccaccttgtgcaagccctgaagacggcgagacgcagtgtttccgagaaggaggtcgagcgctacgaagcgt

ttgccaggtccatgaaggtcgacatgaggaagtttgagaagaagaagaaggacttcaatgatgacggcctctatgaataa

agatcg

>Hypothenemus_hampei_contig_13397|c0_g1_i1 fructose-bisphosphate aldolase

ctccacaccgaccactgcgctaagaagctcctgccctggttggacggtatgctcgatgaggacgagcgctacttcaagct

ccacggcgagcctctcttctcctcccacatgattgatctgtccgaggagcccgtcgactacaacatcaacaccaccgccg

cctaccttaagcgcgctgcccccatgaagcagtggctcgagatggagatcggtatcaccggtggtgaagaggacggggtc

aacaacgagcacgtgcaaaaggagtcgttgtacaccttgcccgaaaccgtgtacaaggtgtacgaggctctttccccaat

tgggcccaacttttccattgccgctgcctttggtaacgtgcacggtgtgtacaagcctggtaacgtgcaattgaagccag

agatcttgggcgag

>Hypothenemus_hampei_contig_13428|c0_g1_i1 Ran GTPase binding

ttctaatccagggcgtggctattttttagtccaaaattcctccctcaacaaagtctgatggtcgaggtggagaagaagga

agaccgtacaaagatagaggctaattgtggaaagcagagggaagaggacgtgtgcagcgggggggaccgaggcggtgaag

gaggcgattccgaggcagatacgaagaaggcgcaggcaagtccgtttctgacgaatgcggtgcctcggaaggaggaggcc

aagaatgcggatggaaaggaggagattgatgcagaggagatagtcgagaagcagaagaagcatcttgaggaaaaccagag

cgacgagatgctgttcaaggcaaggtgcaagctctactacttttccgaggag

>Hypothenemus_hampei_contig_13428|c0_g2_i1 Ran GTPase binding

gaggaggccaagaatgcggatggaaaggaggagattgatgcagaggagatagtcgagaagcagaagaagcatcttgagga

aaaccagagcgacgagatgctgttcaaggcaaggtgcaagctctactacttttccgaggagaccaaggcgctcgaggagc

gtgccgagggaacgatggttattgggatgcatgccaagaacaaccttgtgaagataacagtgtttagagatcaaacgggc

aggctaggatgcaatcactttatcaatccaaggttcaaggcgcagccacacgggaaggtcagcaacggatggatgtggat

gtctacggaggacacggttgaggaggatgcgctccgcaagaaggtccaattgtttgttgttaagttttactctgaggagg

attatacaaagtttggggaggaatacgacctaggccgggtccacaacgaaaaggttctcaaggccaagccaaataaagg

>Hypothenemus_hampei_contig_13435|c0_g1_i1 Triosephosphate isomerase

gacggtggtctctccgttatcttctgcattggtgagactcttgaggagcgtgaggccaacaagaccattgaggtcgttac

ccgccagctcaacgctgccgccaaggagctctccaaggagcagtgggctaaggttgtcatcgcctatgagcccgtctggg

ccatcggtaccggcaaggtcgctaccaccgagcaggcccaggaggtccacgccgctatccgcaagtggctcggcgagtct

atctcctctgaggctcaggagaacgtccgtatcatctacggtggctccgtcagcgagaagaactgccgtgagctcgccca

ggagcccgatgtcgatggtttcctggttggcggtgccagcttgaagcctgccttcgtcgacatcatcaacgcccgtctgt

aaacaacccaaaagtcggacttattagtattcaatgagcaaacctattgccagcagatattagccggctgatggtcgagt

cttgaaagacagatttcgtttttaccgagacatcc

>Hypothenemus_hampei_contig_13435|c0_g1_i2 Triosephosphate isomerase

acgttgtcattgcctacgagcccgtctgggctattggtaccggcaaggtcgctaccaccgagcaggcccaggaggtccac

gccgctatccgcaagtggctcggcgagtctatctcctctgaggctcaggagaacgtccgtatcatctacggtggctccgt

cagcgagaagaactgccgcgagctcgctacccaacccgatgttgacggtttccttgttggcggtgccagcttgaagcctg

ccttcgtcgacatcatcaacgcccgtctgtaaacaacccaaaagtcggacttattagtattcaatgagcaaacctattgc

cagcagatattagccggctgatggtcgagtcttgaaagacagatttcgtttttaccgagacatcc

>Hypothenemus_hampei_contig_13522|c0_g1_i1 NUCLEAR PORE COMPLEX PROTEIN (NUCLEOPORIN NUP116)

gggctgcaagcctccccagcagcaccccccccacaggaacaggttggggagtcgaagggcccagggctcgccagccccaa

ggaaagaacgtccgcgtctgtgttcacacaacctgcgggggagaagagtgtgagcttcatgcagatgacgctaggggaga

tcgtccagcagcagactcggaagcttgaggaaaacataaaagttttcaaggagaaggcaaaggaggtttttgagcaggat

gagaggataatccgatcactgaacaactacaagctgattcggagcaggattgaggaggaggagcggataattgccgagac

ggaggagaacgtcgagttttttgagaggtggctttgctctttccagaaggaggttcccgagggggcggcagacgagcttc

tgacatgtattaaggagtttgagaggatatgcgacaagtacaacaagacgctcgagacacttaaggacgaggaggacgag

gtgatgtgcctggtcaatgagaactacaacctgatcaacacgattgacgagaagcttgatgttcttgagaaata

>Hypothenemus_hampei_contig_13522|c0_g2_i1 NUCLEAR PORE COMPLEX PROTEIN (NUCLEOPORIN NUP116)

gggctgcaagcctccccagcagcaccccccccacaggaacaggttggggagtcgaagggcccagggctcgccagccccaa

ggaaagaacgtccgcgtctgtgttcacacaacctgcgggggagaagagtgtgagcttcatgcagatgacgctaggggaga

tcgtccagcagcagactcggaagcttgaggaaaacataaaagttttcaaggagaaggcaaaggaggtttttgagcaggat

gagaggataatccgatcactgaacaactacaagctgattcggagcaggattgaggaggaggagcggataattgccgagac

ggaggagaacgtcgagtttttttgagaggtggctttgctctttccagaaggaggttcccgagggggcggcagacgagctt

ctgacatgtattaaggagtttgagaggatatgcgacaagtacaacaagacgctcgagacacttaaggacgaggaggacga

ggtgatgtgcctggtcaatgagaactacaacctgatcaacacgattgacgagaagcttgatgttcttgagaaata

>Hypothenemus_hampei_contig_13581|c0_g1_i1 40S ribosomal S22

tcaagacttccgtcctcaacgatacgcttaacgctatcaacaatgccgagaaggctggcaagcgccaggtcctcatccgt

ccttcctccaaggtcatcgtcaagttcctcactgtcatgcagaagcacggctacattggcgagttcgaggaggtcgatga

ccaccgctccggcaagatcgtcatccagctgaacggccgtctcaacaagtgcggtgtcatcaacccccgctaccccgtcc

agctccgtgacctcgagaagtgggctgtccagcttcttccctcccgtcagttcggcttcgtcgtcctgaccacctccgcc

ggtatcatggatcacgaggaggctcgccgcaagcacgttgctggcaagctcctcggcttcttctactagatggagtctca

aaaaaattccaaactcaatgaaaagttgttgttttgaccggttctagtgcttgggctgtgccctgggaatgtaatctaga

taagcggatatacc

>Hypothenemus_hampei_contig_13581|c0_g1_i2 40S ribosomal S22

atatcccacctctttgatttgagaatctcgaattctataccgccaaaatggtcaagacttccgtcctcaacgatgccctc

aacgccatgaacaacgccgagaaggctggcaagcgccaagtcctgatccgtccttcctccaaggtcatcgtcaagttcct

cactgtcatgcagaagcacggctacattggcgagttcgaggaggttgacgaccaccgctccggcaagatcgtcatccagc

tgaacggccgtctcaacaagtgcggtgtcatcaacccccgctaccccgtccagctccgtgacctcgagaagtgggctgtc

cagcttcttccctcccgtcagttcggcttcgtcgtcctgaccacctccgccggtatcatggatcacgaggaggctcgccg

caagcacgttgctggcaagctcctcggcttcttctactagatggagtctcaaaaaaattccaaactcaatgaaaagttgt

tgttttgaccggttctagtgcttgggctgtgccctgggaatgtaatctagataagcggatatacc

>Hypothenemus_hampei_contig_13642|c0_g1_i1 Per1-like membrane

gaccaacaggattccaaggcacgtgccatcaagtaaaacctcaagcaagagactccattggtcaggagacattgcatttg

tttaaaatcatctcgatcccttgggatatgaagtactgcttcggcaaatgttttgaggagtcgagagagcttgtgagagc

aggagtgatcgaccggatcttcaaccggggcaccgacgaaaaggcaaagagcatgtgccacctcctctgcctgaagctca

caaacgggggaaaccttaagaggaacgggagatggggattccagcccattcttggaatgacggagttcttctcggcgctg

ttttcgtttgcaaacctcataataaacatcatctgcttccacaggctgctgagaaagcatctgcaggtgacaaggctcgg

aaggctctatcacgtccagtactacatatgcaacctggcattcatcgccagcaccctgttccacatacacgaaaacacgt

tcacaaggaactgcgattacttccttgcgtttctgacgattctgttcggattctacatggcttttgtcagggtcatcctc

atagcaagcccctcgctcgagatggccacaaggaagccgctccaggcgattttcgtcgcattctacgcataccatgtcta

caggatgtcgaacatcgagttcgactacgtctacaacaaggtctcctgcgcaatcatcgtcgtcctcaccctgctgtccc

accttgtgaccttccttagatacaggaagatgccacactccagaaacatccttctcttcacattcttcttcttcct

>Hypothenemus_hampei_contig_13650|c0_g1_i1 Ankyrin repeats-containing

ttagaagtgataagaaatattgattttagtaacagtaatcaaaaaacagaaatagtaaataaaattacagaatttttatc

acaaattgataaaacattaagtaatagttttgcgaagagtttattaggtatggcgctggtaaatgagcattttactaagc

ctttgttaatagcgtttgacggatttgatgaacttctggataaagcggacagagataaaataatctcattattgaaaagt

ttaaaggatacaactaacgccaaattttggattaccactcgtttgcattatgaagatattttagaagatgcactatcaac

atttgctataaagcttgacccaatggatgatttaacaacaaagaaattcattaaaaa

>Hypothenemus_hampei_contig_13652|c0_g1_i1 hypothetical protein EN45_113680

gatagaacgttctttctgccctgatacattagaggagatcaacgaacttatcgacctcttcagcaacatggaacacggtg

atatctacgacaaacaaggtaatccgatacatcaagaagactatgtgttcactagaatacgcggaggaactcatgaaggc

aaggtcaaagagattgtttcagatgaagatcgagccgcggaaaagtctgtgaaacaccctcctaaggtcttatttaaaaa

taaagatggtaggattgtggctcacaaccccgaaacgttggaaatatctagtgaacactcttaaaagtataccgcaatga

tgctttgttgcttggcgttggaagcccagagtggcatttagtttcattgcggcccacctaaaatccaatattgg

>Hypothenemus_hampei_contig_13666|c0_g1_i1 similarity to HYPOTHETICAL PROTEIN YDOA_SCHPO

atgacttctcgacctcgtcgtcatgcctgttcacaagcaggtggcagctggtgaggtccgacacaatgcagcctcccggc

acgccgtcgctgaagtccaggctggggcatacaatgctgttcatccctcccgggcacatcgaaggcagcatttataataa

ctgccttgtggtcatagggggacagaggaacaaagactcgatacgagagatggttttctacagcctggacacggacacgg

ttttccagaacgtggcgttccccataaagggggacgggaaggttgtgcaaaggggccttctgtattctgcagaaatagtg

gttctgttctcctacggaagggagaaggaggggaagttcgagagcatagatgtgtacacctactcgttgctcagggggaa

gtggaccaaggtggatgtga

>Hypothenemus_hampei_contig_13674|c0_g1_i1 hyphally-regulated cell wall

aaaacttagaagcggtgtttctggtgctggtcaaagcattttaagtaaatttactggatctgttccaagagcttcattca

ctgtttctgatgttgcttgggttgaaggtgatttatttaaagtgactattaacttcgaaactgttgaagctgctaaactt

tttgctaaattccaagatgaattgaaatttctttctgttggtggtgtcggtaatgatggttctgaccaactcttatggga

tggtagttccaacagtaaaattgacaactttgctaaatggtcttctactgttttagttaaagcatctaaacacaacaatc

tttactgtttaccagatgactttgccatcaaatttgattggactgcccaagatgcaagtgaattacattctctctggagt

aaatactttgataccacttatgaatacaccttctcaaaagactttggtac

>Hypothenemus_hampei_contig_13688|c0_g1_i1 adenylate kinase

ccggatgcggcaaggggacgcagtcgcggctgctgtccgagcggtatgggattccacacgtgagctctggggacattgtg

agggaggagcttcggaagaacaccgaggcagcagcaacgatcaggaggatggtggactctgggaggctggcgcccgacga

gctggtgaacgagcttgtgctcaggaaggtcgagaggatgtccgggtacattctggacgggtatcctcggcggcccgagc

aggcggagatgctgggggatggcgtcgacctggtggtgttcatcgacgtcgacgagggcacgtgcatccggcggatctgc

gggcgcaacgagggaaggaaggacgacagcgaggaggttgggcgaaagcggcacggggtgtacaagaaggagacggcgcc

tgtggttgagttctacaagagacaggggaagctggtggcgattgacgggggaaaggaccccaggacggtgttttccgaga

tctgcaaggtcattggat

>Hypothenemus_hampei_contig_1369|c0_g1_i1 40s ribosomal s27

tctacaatcaccaccgtcttctcacacgcccagaccgtcgtcatctgcgccggctgctcgaccgtcctgtgccagcccac

cggtggtaaggcccgtctgactgagggctgctccttccgccggaagtaaacgagacgcgcaaatgaaatttcttttttat

tgtcttgttgaaacgcagaggaaatagcatggagcaatctgggatgaaaataccactcctctttgggttcggttattaaa

tggggaccgccggcttttcaaaatgtcacatacaacaattacccttttttctctctctctcttcctctcttccctcctat

atcttcctactcgcttttttcacgggatgatacgccttttccgttcaacttcttcttccagacaatgtcttaggttcgaa

agttttgaggatgttggttgaggttgaagttgtggaatggaaaatgtgcaaaatgatatgatacgatccataccactttt

ttttatcttccctgaaaacaaaaaaacaattgaaatgtgacgttctgaaacccgtctctttcgtcca

>Hypothenemus_hampei_contig_1369|c0_g2_i1 40S ribosomal S27

tcatcaaccttctcgctgatcatacatcgtcaagatggttctcgctgtcgatctcctcaaccccacgcctcaggctgagg

cccgcaagcacaagcttaagacccttgtgccccagccccgctccttcttcatggacgtcaagtgccccggctgcttcacc

atcaccaccgtcttctctcacgctcagaccgtcgtcatctgtgccggctgctctaacgtcctgtgccagcccaccggtgg

taaggcccgtctgactgagggctgctccttccgccggaagtaaacgagacgcgcaaatgaaatttcttttttattgtctt

gttgaaacgcagaggaaatagcatggagcaatctgggatgaaaataccactcctctttgggttcggttattaaatgggga

ccgccggcttttcaaaatgtcacatacaacaattacccttttttctctctctctcttcctctcttccctcctatatcttc

ctactcgcttttttcacgggatgatacgccttttccgttcaacttcttcttccagacaatgtcttaggttcgaaagtttt

gaggatgttggttgaggttgaagttgtggaatggaaaatgtgcaaaatgatatgatacgatccataccacttttttttat

cttccctgaaaacaaaaaaacaattgaaatgtgacgttctgaaacccgtctctttcgtcca

>Hypothenemus_hampei_contig_1369|c0_g3_i1 40S ribosomal S27

tcatcaaccttctcgctgatcatacatcgtcaagatggttctcgctgtcgatctcctcaaccccacgcctcaggctgagg

cccgcaagcacaagcttaagacccttgtgcctgcccctcgctcgttcttcatggacgtcaagtgccccggctgcttcacc

atcaccaccgtcttctcccacgcccagaccgtcgtcgtctgcgccggctgctcgaccgtcctctgccagcccaccggtgg

taaggccagacttaccgagggatgctctttccggagaaagtagatgtctcgacgcaaatcaggccgaagcgatgaatgaa

agaa

>Hypothenemus_hampei_contig_13710|c0_g1_i1 hypothetical protein Eint_111740

tcgttatcctcggccctgtcgctgcaattgcagagtactacaaccagcatgtgctcccatacgacgtcgatgggggggcc

aatgaggcctactcagactacaacgtcgagtcctcgatgatatccatgagcgagtcgaatatgctcgagggcgagcccaa

ggcccacggccccgcaagagaggttgcttccggagcaagcggggagaccgagcactacgtcttgggcccttcggagtcga

ggtctcctgccggcgccatcaagtccctgaggatcggcaagtttaagaccaatccagaaagcataagcgagctgctccac

cgtgaaatggccgacatgagccccgacctccacagcaagctgacgaccacgctggagcacaggggcgttgaccacaggaa

gtatagagacctcttcctcataaagaagagagtcatcgacgacggccggagaaagataatcaacttcaacatcaaggaga

agataatcccgagagaggggccggaggacctctccatcagcaaggactttggaacaagggccctgctcgggggaatcatg

ttcctgctggcc

>Hypothenemus_hampei_contig_13714|c0_g1_i1 hypothetical protein EHEL_091070

aagccggcattgaggagcgcagcaagaagcaggagagaaaggcagagagggaggagaatatgaggagagccatcaagtcc

caggagactgacttaagaagtaaagaccaggagattcttctccttcggcagaagatatcaaggcttgagtcggccatgga

gataaagggaaagagatacatcaagaagtgcatcgaaaggggagagactgctgtccggatgctggaagaggaaatgaaga

gggagaggaatatgctcttgaaaagaattgaggaggagatagagacctcgaagatgctgatgaagaggaacgaggggctt

aagaagatcatcaacgagctgg

>Hypothenemus_hampei_contig_13719|c0_g1_i1 translationally controlled tumor

gtcaagttagttgacggtgtcgtctacgaagctgactgtgccatggtcaaagttggtggtggtgacattgacattggtgc

caacccatctgctgaagatggtggagatgatgtcgatgaagctgttgaaaccgtcaacaacgtcgtccactctttcagat

tacaagaaaccgctttcgacaagaaatctttcatgacctacatcaagggttacatgaagaaggtcaaatctcacttggct

gaaactaacccagatgctgttgaaacttttgaaaagggtgctcaagcttacgtcaagaaggtcattggttctttcaagga

ctgggaattctacaccggtgaatccatggacccagatgccatggtcgttttgttgaactaccgtgaagacggtactactc

catacgtctgtatctggaagcacggtgctaaggaagaaaagatctaagatggttcgaacgtatgtagtgtataattctta

gttaatt

>Hypothenemus_hampei_contig_13911|c0_g1_i1 hypothetical protein

ggtcgaacttcaagaggataggggcgaacccggtcatcaacgaggccttcaagaggagggagcagttcaacgcaatcagg

aggtttataatcatgttcctgtgtaatgagatagctagcttcaatatcaatattagggactatacagaccattacttttt

ggtactaggaaccatgacagcagcatttgttgggctgagcatcctaccgaacgacgagagtgtcaacaagaggaggtttt

tcctgctgttctcgggcgtctgtgcgttgggcggcgttgcgtatactgttatcgacatcgttagggtctcggccgatgga

agtataagagggtaagtgaagcctataattttagaggattgattcttgagatgatattctacacgtttgcaatggggatt

ctgtttggcggggcgctccgggactaccagatgtttggcagcgggctgaaggagtactaccacgggactctttctgtgag

gaagaacctcagcgtgatgtgacgcgggctcga

>Hypothenemus_hampei_contig_13911|c0_g2_i1 hypothetical protein

ggtcgaacttcaagaggataggggcgaacccggtcatcaacgaggccttcaagaggagggagcagttcaacgcaatcagg

aggcttataatcatgttcctgtgtagtgagatagctagcttcaatatcaatattagggactatacagaccattacttttt

ggtactaggaaccatgacagcagcatttgttgggctgagcatcctaccgaacgacgagagtgtcaacaagaggaggtttt

tcctgctgttctcgggcgtctgtgcgttgggcggcgttgcgtatactgttatcgacatcgttagggtctcggccgatgga

agtataagagggtaagtgaagcctataattttagaggattgattcttgagatgatattctacacgtttgcaatggggatt

ctgtttggcggggcgctccgggactaccagatgtttggcagcgggctgaaggagtactaccacgggactctttctgtgag

gaagaacctcagcgtgatgtgacgcgggctcga

>Hypothenemus_hampei_contig_13930|c0_g1_i1 calponin H2-like calcium-binding

ccatgttccttttgtttctagacacacttattcaaaggcctgaggcgcaaagcctttaatgagagttctcggcagctatt

ccaattatctctttttggacgcacactacctgaaccttaattttgtttgctcccaggtctgtaaatcatagaaaaataga

cgccccgagatggacgagcagcagaaggaggtgacgctttgggttacggcgattctcggggagaagccagaagcagggtc

gttccacgacttgttcaaagacggggtgaggctctgcaagctccttggcaaggtttcatctctcgacgtcaagtataaag

agtccaagcagatattgtacaaagggaaaacatctgt

>Hypothenemus_hampei_contig_13930|c0_g2_i1 calponin H2-like calcium-binding

ccatgttccttttgtttctagacacacttattcaaaggcctgaggcgcaaagcctttaatgagagttctcggcagctatt

ccaattatctctttttggacgcacactacctgaaccttaattttgtttgctcccaggtctgtaaatcatagaaaaataga

cgccccgagatggacgagcagcagaaggaggtgacgctttgggttacggcgattctcggggagaagccagaagcagggtc

gttccacgacttgttcaaagacggggtgaggctctgcaagctccttggcaaggtttcatctctcgacgtcaagtataaag

agtccaagcagatatttgtacaaagggaaaacatctgttcattcatcaacgggcttaagatgctcaagatgaatgagtac

gagctgttccagaccaacgacctgttcgaagcaaaggacctgaagcaagttgtcatctgcctgtatgcactgagcaggca

gctgcagaaagaaaaggtgtttccagggccattcatcggccctaagcttgccacaaagtcccggatcgagttcagccagg

aggttcttgacagaagcaagtgcgctgttcacctccagatggggtattcgggtcaggagatggttgagaacgcccgaagc

aaggctgcttccagcgccgtaaaggatttaaggacagagcccctttaatattaaactcctagtatc

>Hypothenemus_hampei_contig_13942|c0_g1_i1 thioredoxin peroxidase

acccccatatggaaaacttgtgattttccaggtttctagaaacggaagtaccccaggcatgtttcctaagaagatcagtg

actgcaagtacaaggcatttgtcgatggagagatccgggacgtttccctacaggactatgcaggaagatatgttgtgctt

gtgttctacccgctagatttcacttttgtatgccccacggaaatcaacaggatcagcgacctcaaggaggaattccagaa

gagaaacgccgtggtgctacttgtttcctgcgactctgtctacacgcacaaggcatggtccctcacacccagggggtcga

acgggattgaaggtgttgcctggcctatggtgtgggatgcaaggagggagctctgcaaccagttcgggatgtttgatgac

gagagtgggcatcccatgcgaggaacggtgattctctcgaaggacctttccgtcaagcatgtgagtgccaactaccatgc

gattggaaggtccgtcgatgagatcctacgcatgcttgatgcacttgcgttcaacgacgagcacggggaggtttgccctg

tagagtggaagaaggatagcaagagttattaaagtctatcccgtgaaaaa

>Hypothenemus_hampei_contig_13942|c0_g2_i1 thioredoxin peroxidase

acccccatatggaaaacttgtgattttccaggtttctagaaacggaagtaccccaggcatgtttcctaagaagatcagtg

actgcaagtacaaggcatttgtcgatggagagatccgggacgtttccctacaggactatgcaggaagatatgttgtgctt

gtgttctacccgctagatttcacttttgtatgccccacggaaatcaacaggatcagcgacctcaaggaggaattccagaa

gagaaacgccgtggtgctacttgtttcctgcgactctgtctacacgcacaaggcatggtccctcacacccagggggtcga

acgggattgaaggtgttgcctggcctatggtgtgggatgcaaggagggagctctgcaaccagttcgggatgtttgatgac

gagagtgggcatcccatgcgaggaacggtgattctctcgaaggacctttccgtcaagcatgtgagtgccaactaccatgc

gattggaaggtccgttgatgagatcctacgcatgcttga

>Hypothenemus_hampei_contig_13987|c0_g1_i1 60S ribosomal L15

tgcgggagatacacaggcgcaagcagtcggacgtgatgaggtacctgcttcggatccgggtgtgcgagtacaggcagagg

ggcgagtgcttccgggtggagaagccgacgtttcttgagagggccaggacgcttgggtacaaggcaaagcagggatatgt

gctgtacattgcgcgtgtgaagaaggggaacatgaagaggaactacaacaacgggaacacgcgggggaagtgcgtcaatg

cggggatcaaccagatcaagccgtcgatccgcaagcaggcgtcagcggagatggtggcgggcaagaagtgctcgaatctc

ggggtgctgaactcgtactgggttgggcatgatgcgggatacaagtactacgaggtgattatggttgacaggcaccacgc

ggcgatccggaacgacccgaagatcaactggatatgcaagagcacgatgaagcaccgggagtgccgtgggctgacgtctg

cgtcgcgcaagagccgggggcttggaaagggcgttagattcaaccagtcgaagggtgggtcgacgcgagcgtgctggagg

cgaaggaacacgctggtgctgcagaagtaccgctgagattaaaccgaga

>Hypothenemus_hampei_contig_14095|c0_g1_i1 Fe(II)-dependent sulfonate alpha-ketoglutarate dioxygenase

ggtggtgacaccatctttgctgatgtcaaggaagcctacagaagactttctccagagtttcaaaagagattagagggcct

tcatgtattgcacagctccgaagaacaagccagtaattctagaactcaaggtggtgttgaaagaagaaagccagtatcta

acattcacccattgatcagaactcatcctgccaccaaggataaatacatctatttgaacagagctttcagtagaagaatc

gtcgagttaaaggaacaggagtctaatttcttgatggaattcttgtacaaacacattgaatctgctcatgacttgcaatt

gagagctaaatgggaaccacgtactgttgtcgtttgggataatagagtggtccaacattcagctattatcgattgggata

ctccagtttccagacatgctttcagaatcaccccacaagccgaaagaccagttgccaacttagaagacttaaacaaagaa

gagtatgatgttggtgatgtggctgaagctttaaaaaatgctt

>Hypothenemus_hampei_contig_14095|c0_g2_i1 Fe(II)-dependent sulfonate alpha-ketoglutarate dioxygenase

ggtggtgacaccatctttgctgatgtcaaggaagcctacagaagactttctccagagtttcaaaagagattagagggcct

tcatgtattgcacagctccgaagaacaagccagtaattctagaactcaaggtggtgttgaaagaagaaagccagtatcta

acattcacccattgatcagaactcatcctgccaccaaggataaatacatctatttgaacagagctttcagtagaagaatc

gtcgagttaaaggaacaggagtctaatttcttgatggaattcttgtacaaacacattgaatctgctcatgacttgcaatt

gagagctaaatgggaaccacgtactgttgtcgtttgggataatagagtggtccaacattcagctatcattgattgggata

ctccagtttccagacatgctttcagaatcaccccacaagccgaaagaccagttgccaacttagaagacttaaacaaagaa

gagtatgatgttggtgatgtggctgaagctttaaaaaatgctt

>Hypothenemus_hampei_contig_14134|c0_g1_i1 predicted protein

acagatctatcccataaagaaccatctacaaaggctcttgtcgaaaagtccaaggagaagttagaaaatgctcaaggcgg

tctaaatgatccagatcccgagcttagagccggggttagtgttgacaagagcaaggccccggttcagcaggacttgagca

aggggagcacgcaacgagcgcaatactcgggaggaggctcagctagttcgactggtcatattgccgactatactggtatt

cattgattatacttcatgaggttgaccatgtcgttcaatagctttttcccggaatgtgttaaattaaatcattgtcattg

attctagagaaa

>Hypothenemus_hampei_contig_14145|c0_g1_i1 hypothetical protein EROM_081670

cttgcagaggactttgtccgggacatcgagcagggcggcaggatcttctacgagagggcagagtccttcaagctgtgagc

cctgtgcccggattcttttggcaagccccagcacccccgatggaaaaaattgatcttgaggacgtcgacgacgccatacg

cagggaaaagctgtttgtccgcgccgcaaccaacctgctgcagaccctggagttcaagagggttgaggagggctacgacg

gcgagctgatgcggccgcccaaggagatcgaggatgcagagaaagacaggcagaggaaggccgagaagatggcaaggcgg

cacagcgagatcaggatgaggaggagcaaggccagggagctggattccgaagagcttgagacggagggcgttacagcgcc

tgtgctgagcaacagggagacgcgaaggaagaacaagagcaagcaccccgtcaacacgtcgctcaagaggcggcggatga

agtcgaggcagaagagacacaggcactgatgggcgtgggctgcggcccggggctgaaataaagaggttcaagatgtgttt

tgtaacgggtgctctggccacaaggcaattggttgcaggccccgaaacgcagacggcagggacggtcacgaatgggatgc

aggcgcccccttaaaacaggtgataaggatgtcttggcaggcgaggtcat

>Hypothenemus_hampei_contig_14165|c0_g1_i1 1,3-beta-glucan synthase component FKS1

agatttcttcattgattaccgtgattttattagatggttatctcgtggtaatactaaatggcacagaaattcttggattg

gttacgttcgtctttccagatctagaattactggtttcaaacgtaagttgactggtgatatttctgaaaaagctgccggt

gatgcttccagagctcacagatctaatgttatttttgctgatttcattccatgtcttatttacactgctggtttgtatgt

tgcttatactttcatctgtgctcaaactggtgttaccaagaattcttacacctttaatggatcaactgaaccaattcatg

ttaactctactgtcagagttgttatttgtgctttagccccagttcttattgatatgggtgttcttggtttctgtcttggt

ttagcatgttgtgccggtccattattaggtttatgttgtaagaagactggtgctgtcattgctggtgttgcccatggttt

tgctgttgtcattcatttgattttcttcattgtca

>Hypothenemus_hampei_contig_14202|c0_g1_i1 hypothetical protein ECU09_0520

gatggagagatatgcggactaccagcactctgtgttttgcctccggatcataaaatctgttgcggggtcgaagttctatc

tcccgctgtcgttctacctcatccggatcctgaggaatgcaatggcagccaggaatatcactgcaagcggcaggaaggtg

gactacgacatgataacgccatcctccgagagagcaaggagcgaagagcaccagatgtttgtgataggagaggtgaatgc

cctgctgatgcagcacatgtcggcgttctcgcggaacattgggtttccggagcttgcagcagccgtcacgggagagctga

gaaagctgagggtagggatctacaaggagatcgttggggacatgatgtcgatcattgacaggcagagagagtatgtgctt

gagaagagaaaca

>Hypothenemus_hampei_contig_14228|c0_g1_i1 RSC chromatin remodeling complex subunit RSC8

atcacagagtctctttttggaagcatgtggtccaagaaggaggagtttcttttgctggaggcaatcaataggtttggaga

cgagtggagccttgtcagccagcatgtgcagacaaagacgaaggagcagtgcatactccacttcctccgcctgccgatac

tagagaacacattctcgaaggcggatttttctgtgggaaggccgttcgaaacggccgagaacccaatcatgtgcaccatt

gtgttcatatgcgggattgtccatccaaaagttgcgtctgaatgcgcaagagtggccatcaaacacattggcaaatgctc

ccaggaaacagttgtccatcacatactggaggcagctcgggaaaaggccgccgagcaaagaagcctggagaaggcaaaga

tagaaagaattaggaatgtgatgtgcgagggcctactgaacaagatcaagatgaaggtgggtacttgcaaggacttgtat

ctatcgacacagcgagtgcgcagtgagctagtggggctgagaaggagtcttgtcgaagagctgggccttg

>Hypothenemus_hampei_contig_14262|c0_g1_i1 Aldo keto reductase

acaacgagattccaattgtgggattaggaacatggaaagccaaggatgaagaagtatatctttccttgatacatgctttg

gaagctggatacagacacatcgataccgcagccgcatacggtaacgaagaacctattggcagagcaatcaaggattcagg

agtcccaagagaagagctctttgttactaccaaattgtggtccaatcgtcaccatgatccagttggagcaatcaaagagt

ccttagacaaattacaaatggattacgtggatttatacttattacactggcctgtatttttgaatccacaaggttctcct

gctagagttcctcttttaccaaatggtcaaagggatattgttatggattggtcctttgttaaaacttatagcttgatgca

tgaagtgcaagcacaaggattagcaaagtccattggtgtttctaacttttcagtaaagaatttgaaaatcttgttgagag

caccagaaactaaagtcactccagttgttaaccaagtggaattgcacccacatttaccacaatttgccttgggtgagttt

tgtgcaaagaatgacattaaattgcaagcctactctcctttaggatcggtgggatctccattattaaaggatgagacttt

actggctatcggtcaaaagtatggagtttcaccagccactgttatctttagttggatgacggaaagagatattgtttact

tacccaagtcaatcaacaaagacagattaagtaagaatttatctgttgtgaaacttgatgaagaagatgttgcaactatt

aacaacatccacaaaaccttctctaagagatacaatgatcaagattggtctcctatgaagccatttgaagaagattaaac

aaagggtatatatatttataaatagtgtgaattagtactggttt

>Hypothenemus_hampei_contig_14319|c0_g1_i1 40S ribosomal S8

cgacaaccccaacgacccttgcccgctgcgttcctggtgaagggctgcaggattagagtccatacatcgacaacatgggt

atttctcgcgactcccgtcacaagcgctcggctaccggtgcgaagagggccacctaccgcaagaagagggccttcgagaa

gggtcgtcagccctccaacacccgtatcggtaccaagagaatccatctcgtccgtacccgcggtggtaaccgcaagttcc

gtggtctccgtctcgaggccggtaacttctcgtggggatccgagggaatctcccgcaagacccgtgtcatcgtcgtggcc

taccacccctccaacaacgagctggtccgtaccaacaccttgaccaagtccgccgttgtccagatcgatgccgctccctt

ccgtcaatggtacgaggcccactacggccagcccatcggccgtcgccgccagcagaagtccgacgtccccgaggagaaga

agagcaacagcgtgaccaagaagcaggctgctcgcttcgccgacaacggcaaggtcgagtccgccatcgagcgacagttc

gaggccggtcgtctgtacgccgtcatcgcctcgcgtcccggccagagcggccgtgtggacggttacatcctggagggtga

tgagctggctttctaccagcgtgcgatccggaagtaaacgaacgagaagaaaaaaaaagagaatcagga

>Hypothenemus_hampei_contig_14319|c0_g2_i1 40S ribosomal S8-B

ccgcacccgtggtggtaaccagaagttccgtgctctccgtctcgagtccggtaacttctcctggggttccgagggcattg

cccgcaagacccgtgtcatcggtgtctctttccacccctccaacaacgagttggtccgcacaaacaccttgaccaagtcc

gctgtcgtccaaattgacgctgcccccttccgtcaatggtacgaggcccactacggccagtctatcggtcgccgtcgcca

ggccaagaacgccgatgccaccgaggagaagaagagcaagtctgtcaccaagaagcaggctgagcgctacgccgagcagg

gcaaggttgagggtgccatcgagcgccagttcgagtccggccgtctgtacgctgttgtctcttcccgtcccggccagtcc

ggtcgtgttgacggctacattctggagggtgaggagctggctttctaccagcgtgcgatccgcaagtaaacgaaaaaaca

aaagggaaaaccatacggttgggtgccgcatgggttgtcgt

>Hypothenemus_hampei_contig_14319|c0_g2_i2 40S ribosomal S8

ccgcacccgtggtggtaaccagaagttccgtgctctccgtctcgagtccggtaacttctcctggggttccgagggcattg

cccgcaagacccgtgtcatcggtgtctctttccacccctccaacaacgagttggtccgcacaaacaccttgaccaagtcc

gctgtcgtccaaattgacgctgcccccttccgtcaatggtacgaggcccactacggccagtctatcggtcgccgtcgcca

ggccaagaacgccgatgccaccgaggagaagaagagcaagtctgtcaccaagaagcaggctgagcgctacgccgagcagg

gcaaggttgagggtgccatcgagcgccagttcgagtccggccgtctgtacgctgttgtctcttcccgccccggccagtct

ggccgctgtgacggctacattctggagggtgaggagctcgctttctaccagcgtgctatccgcaagtaaacgaaaaacaa

aaatggaaagccattcggttaaaggaccgcgtgggttgtcgatgcacgggtggttatcggagtttcagctatgtttacca

gaaaacttgtcatgatctgggcatccgcgggtgcaatgtctgtag

>Hypothenemus_hampei_contig_14339|c2_g1_i1 ubiquitin C

ggaattccaccagaccaacaaagattgatttttgctggtaagcaattggaagatggtagaactttggctgattacaacat

tcaaaaagaatccactcttcacttggttctcagattgagaggtggtatgcaaattttcgtcaagactttaaccggtaaga

ccattaccttagaagttgaatcttctgacaccatcgacaacgtcaaatctaaaatccaagacaaagaaggtattccacca

gatcaacaaagattgatttttgctggtaagcaattggaagacggtagaactttggctgattacaacatccaaaaggaatc

cactcttcacttggtccttagattgagaggtggtatgcaaattttcgtcaagactttgactggtaaaactatcactttag

aagtcgaatcctctgacaccattgacaacgtcaaatctaaaatccaagacaaggaaggtattccaccagatcaacaaaga

ttgatttttgctggtaagcaattggaaga

>Hypothenemus_hampei_contig_14339|c2_g1_i2 ubiquitin C

tctaaaatccaagacaaagaaggtattccaccagatcaacaaagattgatttttgctggtaagcaattggaagacggtag

aactttggctgattacaacattcaaaaagaatccactcttcacttggttctcagattgagaggtggtatgcaaattttcg

tcaagactttaaccggtaagaccattaccttagaagttgaatcttctgacaccatcgacaacgtcaaatctaaaatccaa

gacaaagaaggtattccaccagatcaacaaagattgatttttgctggtaagcaattggaagacggtagaactttggctga

ttacaacatccaaaaggaatccactcttcacttggtccttagattgagaggtggtatgcaaattttcgtcaagactttaa

ccggtaagaccattaccttagaagtcgaatcttctgacaccattgacaacgtcaaatctaaaatccaagacaaagaaggt

attccaccagatcaacaaagattgatttttgctggtaagcaattggaagacggtagaacttt

>Hypothenemus_hampei_contig_14339|c2_g2_i1 Ubiquitin-40S ribosomal S27a

tctaaaatccaagacaaagaaggtattccaccagatcaacaaagattaatctttgctggtaagcaattggaagacggtag

aactttagctgactacaacatccaaaaggaatccactcttcacttggttcttagattaagaggtggtggtaaaaagagaa

aaaaagaaggtctacaccaccccaaagaagatcaagcacaagcacagaaagcacaaattggctgtcttgacttactacaa

ggttgacaacgatggaaaggttgaaagattaagaaaggaatgtccatctgaaacttgtggtgctggtatcttcatggcta

acatgggtgacagacaatactgtggtaaatgtcacttaacttacaaatctaactaagtcctttagttgagtttgttaagt

ttggttt

>Hypothenemus_hampei_contig_14339|c2_g3_i1 ubiquitin-40S ribosomal S31 fusion

ttgaggaagacaagtctgtcgaggccttcatcaagatgcagatcttcgtcaagaccctcacgggtaagactatcaccctt

gaggtggagtcctcggacaccattgacaatgtcaagtccaagatccaggacaaggagggaatcccccctgaccagcagcg

tctgatcttcgctggtaagcagctggaggacggccgtactctttcggactacaacatccagaaggagtcgaccctccacc

tggtgctgcgcctgcgtggtggtggtaagaagagaaagaagaaggtgtacaccacccccaagaagatcaagcacaagcgc

aagaagaccaagctcgccgtcctgaagt

>Hypothenemus_hampei_contig_14339|c2_g3_i2 ubiquitin-40S ribosomal S31 fusion

ttgaggaagacaagtctgtcgaggccttcatcaagatgcagatcttcgtcaagaccctcacgggtaagactatcaccctt

gaggtggagtcctcggacaccattgacaatgtcaagtccaagatccaggacaaggagggaatcccccctgaccagcagcg

tctgatcttcgctggtaagcagctggaggacggccgcactctctctgactacaacattcagaaggagtcgaccctccacc

tggtgctgcgcctgcgtggtggtggtaagaagagaaagaagaaggtgtacaccaccccaaagaagatcaagcacaagcac

agaaagcacaagttggctgtgttgacctactaca

>Hypothenemus_hampei_contig_14339|c2_g4_i1 ubiquitin-60S ribosomal L40 fusion

ttgaggaagacaagtctgtcgaggccttcatcaagatgcagatcttcgtcaagaccctcacgggtaagaccatcaccctt

gaggttgagtcctcggacaccatcgacaatgtcaagtccaagatccaggacaaggagggtatcccccctgaccaacagcg

cctgatcttcgctggcaagcagctcgaggatggccgcactctgagcgactacaacatccagaaggagtccacccttcacc

ttgtcctccgtctgcgtggtggtatcattgagccttccctgaaggcccttgcctccaagtacaactgcgagaagtccatc

tgccgcaagtgctacgcccgtctccctccccgtgccaccaactgccgtaagaagaagtgcggtcacaccaaccagctccg

ccccaagaagaagctcaaatagacgactcgcttattggtgttgtgtgctcggtgtgctggggtgcagaatccaggtctgg

tgtatggctggaacgcgacacgcagaggtttttacatggcgttagaaatgatgaaacaatcacagccagtcatactgttt

ttagttggcacctctcaatcaggtcgcaatccatgaattgaagtaaaagttc

>Hypothenemus_hampei_contig_14339|c2_g4_i2 ubiquitin-60S ribosomal L40 fusion

gtcaagaccctcacgggtaagaccatcacccttgaggttgagtcctcggacaccatcgacaatgtcaagtccaagatcca

ggacaaggagggcatcccccccgaccagcagcgtcttatcttcgctggtaagcagcttgaggatggccgcaccctgagcg

actacaacatccagaaggagtccacccttcaccttgtgctccgtctgcgtggtggtatcatcgagccctcccttaaggcc

cttgcctccaagtacaactgcgagaagtccatctgccgcaagtgctacgctcgtctgccccctcgtgccaccaactgccg

taagaagaagtgcggtcacaccaaccagctccgccccaagaagaagctcaaatagacgactcgcttattggtgttgtgtg

ctcggtgtgctggggtgcagaatccaggtctggtgtatggctggaacgcgacacgcagaggtttttacatggcgttagaa

atgatgaaacaatcacagccagtcatactgtttttagttggcacctctcaatcaggtcgcaatccatgaattgaagtaaa

agttc

>Hypothenemus_hampei_contig_14339|c2_g5_i1 ubiquitin

tctaaaatccaagacaaagaaggtattccaccagatcaacaaagattgattttcgctggtaagcaattagaagacggtag

aactttatctgactacaacattcaaaaagaatccaccttgcacttggtcttaagattgagaggtggtatgcaaatcttcg

ttaaaaccttgactggtaaaaccatcaccttggaagtcgaatcttctgacactattgacaatgtcaagtctaaaatccaa

gacaaagaaggtattccaccagatcaacaaagattgatcttcgctggtaagcaattagaagacggtagaactttatctga

ctacaacattcaaaaagaatccaccttgcacttggtcttaagattgagaggtggtatgcaaatcttcgttaaaaccttga

ctggtaaaaccatcaccttggaagtcgaatcttctgacactattgacaatgtcaagtctaaaatccaagacaaagaagg

>Hypothenemus_hampei_contig_14339|c3_g1_i1 polyubiquitin containing 7 ubiquitin partial

gagaggtggtatgcaaatcttcgtcaagaccctcactggaaagactattaccctggaggtggagtcttctgataccatcg

acaacgtgaagagcaagattcaagacaaggagggtattcccccagaccagcagcgtctcatcttcgctggtaagcagttg

gaagatggtcgcacactgtccgactacaacatccagaaggagtctaccctccacctggtgctccgtctgcgtggtggcca

gtaatcttgttctgctttttatgatttcgactacgataggttctatatgacctgcatggcgtttggtcggttttgaagct

gaggcttctgttctctttttcccatttat

>Hypothenemus_hampei_contig_14454|c0_g1_i1 60S ribosomal

gacggcaagggacatctccttggtcgcctggccagcactgtcgctaagcagctgctcaacggccagaagatcgtcgttgt

gagatgtgaagccctcaacatctccggcgagttcttccgcgcgaagctcaagtaccacgcctaccttcgcaagatgactc

gtttcaaccccacccgtggtggtcccttccacttccgcgctccttcccgcatcttctacaaggctgtccgcggcatgatt

ccccacaagaccgcccgtggtgccgccgccatggagcgcctcaaggtcttcgagggtgtcccccctccctacgacaagaa

gaagcgcgttgtcgttccccaggctctgcgcgttctgcgtctccgccccggccgcaagtactgcaccgtcggccgtctca

gccacgaggttggctggaagtaccaggacgtcgtctccagacttgaggagcgtcggaaggttaagagcaaggcttactac

gagcgcaagaaggccgctcgccgtaaccttgctaaggcggagcagagcgcaaacgttgacagcaagaccaaggcccagct

tgctgagtacggctactagatacctgataaagttcggccggttgttgtgaagtgacgaaggtctgggttgggatcgcgga

cggttttgtttcagcgaattgaacggcaagccgatcgacctttttccatggcccccggtgatctcttttccttttccttg

gccacttcatgaataccatgacctcgccggttgtgacggacatgggtggccgagggcggcggagattctgggtggcttcg

gaagtttttcacaaaaagcagaaaaaacggagtcgcgggatgaa

>Hypothenemus_hampei_contig_14454|c0_g1_i2 60S ribosomal L16

cgcaagatgactcgtttcaaccccacccgtggtggtcccttccacttccgcgctccttcccgcatcttctacaaggctgt

ccgcggcatgattccccacaagaccgcccgtggtaccgccgccatggagaggctgaaggtcttcgagggtgttccccctc

cctacgacaagaagaagcgcgtcgtcgttccccaggctctgcgtgtcctccgcctccgccccggccgcaagtactgcacc

gtcggccgtctcagccacgaggttggctggaagtaccaggatgttgttgctagactcgaggagcgcagaaaggtcaagag

cagtgcatactacgagcgcaagaaggccgctcgccgtaaccttgctaaggcggagcagagcgcaaacgttgacagcaaga

ccaaggcccagcttgctgagtacggctactagatacctgataaagttcggccggttgttgtgaagtgacgaaggtctggg

ttgggatcgcggacggttttgtttcagcgaattgaacggcaagccgatcgacctttttccatggcccccggtgatctctt

ttccttttccttggccacttcatgaataccatgacctcgccggttgtgacggacatgggtggccgagggcggcggagatt

ctgggtggcttcggaagtttttcacaaaaagcagaaaaaacggagtcgcgggatgaa

>Hypothenemus_hampei_contig_14524|c0_g1_i1 hypothetical protein PITC_063460

acgtccgcattggcgagtctgctatgccctggcacagcgagtctgaggatggcaaggtctacaagtaccagtaccaccct

cacggcgacaagagccagcccctccgtgctgctcccagtgccatgaacactgtcattgtccccaatgtcaccctgcccga

ggacctccacgagaagttcaacaagtacggcaaggaggagtgggactactaaacagttcactttgagctttccaaaacat

attgtgagggcaaaccgccgtttatgcggtgacctattgtatactaagaccacggcaaggtggaggtagattgcgtggac

ttatgattgcgattaccggggatt

>Hypothenemus_hampei_contig_14577|c0_g1_i1 Ribosome biogenesis SLX9

gatctaccactgcatcaccatggctcccattcgatccgtcaagaagcaggcgaccgcaagaaagccactgagggccggca

ccgcaacccagtccggcatcttcaacgatgacttccgaacgacaaagaaggacaaacgtcagatcaagcacaacagcttc

atgtcgaagattgagaagaattcgcaaagatcagccaagcgtcgcatgtcgtccaagaacctggccgccaatcttagctc

gttggccgatgccctgcccgaaaccgaagaattcaatgacccagataaccaggtgaaggtgatcaagcaaacgactctca

gacataaaccggg

>Hypothenemus_hampei_contig_14609|c0_g1_i1 hypothetical protein PGUG_04923

gtttcgaggaaacctgtgtgtctgccgactcgttgactgtgactctcaaggacggagttttgaaggacagcaagggccgt

attggctcgattgttgccaaccgtcaattccagttcgatggtcctccacctcaagccggtgccatctacgccgcaggctg

gtcgatcgttcctttggactacaaggaagacaagaaaaccaagcaattggccgacgaccagcaaaagcaagaaaagtcca

agtccaaggacaagaaatccaagaacaagagggatgataccaagtggggcaagttggccattggcaagcaaaccactttc

tataagtgcttgtcgggagacttctacaacttgtacgacgaaagtattggcggccaatgttcagaggtcgaacttgtggt

gttgaaggcagttgagtgttaactttaaaaattgcattacgttccttagcatttgtttttcttcatcgacggtttttttc

gagctacttgcatgcaatagctatagcatcattt

>Hypothenemus_hampei_contig_14624|c0_g1_i1 hypothetical protein ECU10_0230

tgttgcaaagatggcacttgaaccttgtagctgacaatgttgacccataaagcgcagatgcttttcatgcaagaaagcat

caaagagaagatcgactccattgatgcgcttatgaggcgggtggacgagaacacaaacatctctgttgttgacatcctca

aggaggagattgtcaagctccggaggctcaatgaggaatacagaaagatattggaggataagaaggtcgtgcacaaggat

caggctcaaggcaagaccaggtattaccttcgggacggatccacatatgtcgtcaagggaaaccagtacaggtatctata

cgacgcaaagaccaaggtgattacctacgagttctcaaacggacaggtcgaaaagacgtttcccctggggttgaaggaaa

tcagacatccggacggcagcatcacaataaaaagtgggtcccgggaccatgagtacat

>Hypothenemus_hampei_contig_14689|c0_g1_i1 molybdopterin and thiamine biosynthesis E1

gatcttgaaggagctcaagagtgggcattcacaagaaaaccctcttgcccatgcgtgtagccgtatggttgccgctgccc

cccaggagacagatgtccacgtccccacagtgggatggagagatgtactaggtgatcttgagtcctaccttcttatcgat

gtccggccaccgggtcaatacggggtgttcaacgtcaagggatctgtgaacattccccttggagacctgcctgagagggt

caagtccatagagccgttcgggaggaaggtgggggttgtctgcaagagaggtgtgtcttccaaaaagggaacccagatac

ttggggagagcggcctg

>Hypothenemus_hampei_contig_14725|c0_g1_i1 Ham1 nucleoside triphosphatase

ttgcaacaacgaatgtgaagaagttgaatgagatcaggatgactctcaagatggaggtggaacacttgaacgtggagatg

gatgagctccagggaagcatggaggacattgtgagccacaagctcgaccaagtgattccgttcataagcgaggacgatgc

tgtgatcatcgatgacacaagcctctcgctgaagggcttggagggtttccgggagtgtatatcaaggactttctcaagat

agggtccaggaagatccttgagattgttaccaagatgggcgagaacggcgcaacagccatgtgtggcctaggaattgccc

actacaaggacggaaagatagtcaagaaagtgttttccggatgccttgagggcacgattgtgggatgcaaagatgatgcc

ttgaagggatttgactacatcttccttcccaaaggatctgatgtctgcatcggggagctctcgcccgaagagaagaacag

aataagccatcggagaattgcaagcgacaaggtggccagctacatgatttctgcgggaatagttaattaaaccccatggc

ttctaaaaaaaa

>Hypothenemus_hampei_contig_14725|c0_g2_i1 Ham1 nucleoside triphosphatase

ttgcaacaacgaatgtgaagaagttgaatgagatcaggatgactctcaagatggaggtggaacacttgaacgtggagatg

gatgagctccagggaagcatggaggacattgtgagccacaagctcgaccaagtgattccgttcataagcgaggacgatgc

tgtgatcatcgatgacacaagcctctcgctgaaggcgcttggagggtttccgggagtgtatatcaaggactttctcaaga

tagggtccaggaagatccttgagattgttaccaagatgggcgagaacggcgcaacagccatgtgtggcctaggaattgcc

cactacaaggacggaaagatagtcaagaaagtgttttccggatgccttgagggcacgattgtgggatgcaaagatgatgc

cttgaagggatttgactacatcttccttcccaaaggatctgatgtctgcatcggggagctctcgcccgaagagaagaaca

gaataagccatcggagaattgcaagcgacaaggtggccagctacatgatttctgcgggaatagttaattaaaccccatgg

cttctaaaaaaaa

>Hypothenemus_hampei_contig_14728|c0_g1_i1 DEHA2C13398p

aatacaaatgtttcgtcaattagctttacgtcaatccaatgttttaaaatccactcaagttagatacttgagtgctagcg

caccaagatcctctatccttggaggtgttaaggaaactctacaaaaagttaataagaagactggggaagttttagctgaa

ggtatggagaaggcagaagataagactccaaatgcatccagtgtcggtgatgctgcttcgaagattaataagaagaccgg

agaagtgttggctgatggtatgcaaaaagttgaagataaagcaccaaatgttgatagtgttggagatgctgcatctaaag

caaacaagaagactggagaagtattagctgatggtatggaaaaggctgagaaggttgttccaactgccagtaaggccgat

gctaagaagaaggttgatgaaaatgtcaaagggtatgacaatttacaacataaaggaagtagagttgagactgagcaaaa

tcgtcctgatgacgccgt

>Hypothenemus_hampei_contig_14756|c0_g1_i1 stem-specific TSJT1-like

agactatttggttcaagcttcagatagaggaaaagatgctggcaatctttcacaaggcattcgctcatccaccagatgag

cttaatagccctgcatctcaccatggtagcaagaagccaaagatgccagaggagaccctcaaagagtttctcgcttctaa

ccccagtgatgctttttctttgagctttggagatgctgctgtgctttcttatgttcgacaagaccaaccttccctcctac

acaagaaccaaaggttgttctgtgggcatgatgagatatactgcctcttcatgggaagcttgaacaatttgtgtgctcaa

atcaagcagtatgggctatcaaaaggc

>Hypothenemus_hampei_contig_14761|c0_g1_i1 beta tubulin

aggaggccgaatcgagcgactgcctgcaggggttccagatcacgcactcgcttgggggaggaaccggggcagggatgggg

acgctgctgctgtcgaagatcagggaggactttccagacaggatgatatgtacattttctgttgttccgtcgccaaaggt

gagcgacacggttgttgagccgtacaatgcaacgctctcgatccaccagctggtcgagaatgcagatgagacgttctgca

tcgacaacgaagcgctgtacgacatatgcttcaggacgctgaagctgagcaaccctgggtacggggacctgaaccatctt

gtttcactggtgatgagcggggtgacgacgtgccttaggtttcctgggcagctgaacgcagacctgcggaagcttgcagt

caacatgattccgttcccgcggctgcacttctttgtggttgggtttgcgcctctgactgcgaccaggacgcagaagttcg

agacgcactctgtgtctgagcttacacagcaaatgtttgactcgaagaacatgatgactgcgtgcgacccgaagaagggc

aggtatcttactgttgcagcggtgttcagggggaagatttcgatgaaggatgtcgacgagcagatgtcgctggtgcagtc

gaagaacagctctctgtttgtggagtggattccaagcaacgtgaagaccgcggtgtgcgacattgcgcctcagggccttc

agatgtctgcgacgtttgttgggaacacaacatcgatccaggagctcttcaagagggtgtctgaccagttcactgtgatg

ttcaggaggaaggcgttcttgcattggtataccggagaggggatggacgagatggaattttccgaggcagagtcgaacat

gaacgacctgctttccgagtaccagcagtaccaggatgcaacggtcgaggataccgaagagtttctagttaattaaagga

ttg

>Hypothenemus_hampei_contig_14783|c0_g1_i1 with calponin domain

gcgctcctcgaggagtccgactacgggtctgaggagcgagacgaggacggtacggacaagtacctgctgtctgtcgatgg

ggagaggaagagctttgagctgaggttttactctcctctgtacccgttctttgcaaagtgccgcgagcggtaccagggga

tcctgagcaagctgatagggatctcgacactcgaccactacttccagttcgagtcgatgaacgagcttgggaagctcaag

agggcaacggtgcatctcccaagcaccacggccaaccagctgatcaccctgctgctgaacaacgccaagctcctggactc

cgagctatgcgagatcctggaggcattgcccctgttccctgacgaccagaacaagacgctgtcgttcacattcttcgggc

ccgagtggatatgctcgcaagaccccgagaagattgcgctggacaacttcatcagggggctgaagaagaagctgatatat

gcgatctcggtgtgtaagggcaggaacctggtggagatgcttgtccgggagtctgacgacgaggagagagggctctacct

ggacatcaagaggaatggtgtgtacagcaagggccacgaggactcgcagtcggtgacctctccgcccgcacagtacgaga

caatcgatctgctcaaggaagcaatcatcgacgacctcaacttcctggaagacaagaagatcaccagcagacacaacctg

tactcggagctgctgttcatgctggcgcaggacattgtcatcctgaggttcatgtccgaggagaggagcaaggagctccg

gatcaacgagctcagctacgacaacctgtgctatagagacgactacctgtccagcaagatcgagctctaccagaactacc

tcaactcgtttgtcgcaaagctggccgtcaagaagagaggcctgtttgggtttgtggcagacgacattgccagagactcg

aagtacggcacgcacaagtacagtgcagacaggctgatgagcatgggggtgctggtgcagatctacgagtcgcccgacgt

ctccgagatcttcttcctgttcctctccgactcgccgctgctcttcagcgtggagatctatgtccagaacatccttgtgt

cccaccccgtttcgttccgcttcgacgacctcctcaagctcaagaagaacggaaacagcgtttgcgacattgcaggcatc

tgctcgttcagcgtgtgtagattcattgaccttgtcaactccaagtacattgggagtgaaggatgacctgccttgcatta

aaggtg

>Hypothenemus_hampei_contig_14788|c0_g1_i1 phosphoenolpyruvate carboxykinase

gcccatgactcctgatgtctggcggatcaaccgtgagcgtgctgtcgactacctcaacacccgtaaccgcatctatgtca

tcgatggtttcgctggctgggacgagagataccgcatcagtgtccgtgttgtctgcgcccgtgcctaccatgctctcttc

atgcgcaacatgctcatccgcccctcggcggaggagctgcagcacttccaccccgactacgtgatctacaacgccggatc

tttccctgccaaccgcttcaccgagggtatgacctctgccacctcggtcgccatcaacttcgccgagaaggagatggtca

tcctgggtactgagtacgccggagagatg

>Hypothenemus_hampei_contig_14793|c0_g1_i1 Glutathione-dependent formaldehyde dehydrogenase

gctggtgagtacaccaagggatgggctgattattttgagagactgttgggtttggaaacagatacagctgaacttgtcaa

cgagatgattacttcggtacgaggattcgggcgctgtggaattacaggtgtctatgccggttattgtaatcacttcaata

tcggatccttaatggagactgggattcgtcttatcggcaatggacaagcccctgtacacaaatactggaaagatctcttg

aagtatattcagcaggaaaagatccatcccttgcacatggtcacccatcgcttcaagcttgaagacatggagaaaatcta

cgatttattcaacaagcgtgagctgggcatgcagaagattttcgtacagacaaaattttctgctcctccagcgtcagggg

cacctgcgttaacagaactttgagcttcatggagttat

>Hypothenemus_hampei_contig_14799|c0_g1_i1 ubiquitin conjugating enzyme E2

ctcctggagcccgcagacggctgcggagggaggaggatatgctgaaggaatccacgggcgacgagagcaagcttttcagg

gcatgcccacagggaccgaaggagaacaataactacaagacttgggacgtctatttcacgcttgggggtgattctctata

tgccgggagggttctgaaggccgtgatggtgttcccggactgctatcctctacagccgccgaccctcaggtttgtgtcga

agatgttccatccaaatatctatgaggacgggaggatgtgcatatcgattcttgaggaagacgtcccagaccccactggg

tatggcgactcaaaggacaagtgggcgcctgtgcaaaacattcggacgattctcctgtcgattgtggtgatccttaactc

cccaaacacgtcttccccagcaaacgtagatgcctctgtgatgcacagggacagccctgaggagtatgcaaagaaggtca

tcaagattgctcgggaggaggatgagaagcttagaagtgcagatccacaggccgagaaaattgcactggaggcagagacc

gg

>Hypothenemus_hampei_contig_14818|c0_g1_i1 hypothetical protein EHEL_020910

atgggcgctctgcagggcagaagactcgtccacaaagtcgacggacaatatcttcaagaagttcctggaggttgccgtga

gcgacgagcaccacaggctgcagcctgccaagggcaagaagcacgacgggcaggtgcccaagagccatcccgtgccgaag

aagaagacggtgattgttctggaggaggactccagcaagaaggacgggggtctgaatccggaggtcgggcagagggcagg

cagtgtcgcaaagagtattgagaagaagggcgaggcgcagccgcagaagcccaaggccggcgagcagccgcaggaggact

ctccaaagccagacgtgcagtcggagcttacgtttgagacgccgatctcggcatcgtttcttctcaaacccctaggggcc

aaggaggtcacgctcacacaagccgaggccaaggaagtgagagagagcttcaagaccctggagaggctttcgaaggacct

ggaggcgctgcgcagcaggctgcagtcggcgctcaaaaagctccttgggccggagagggatgagaagggctcgaagcccc

ctacagaccttctgaactacaacttcatagaggtcactgagacggggcaggcgacaaagaagtgac

>Hypothenemus_hampei_contig_14863|c0_g1_i1 ribosomal S3Ae

ggtttaaagggaagagttgtcgaagtctctttagctgacttacaaggttctgaagatcactcttacagaaacgttaagtt

gagagttgatgaagttcaaggtaagaacttgttgactaacttccacggattaaacttcacctctgataaattaagatctt

tggttagaaaatggcaatctttggttgaagctaacgttactgttaagactgctgatgactacgttttgagagttttcgct

attgctttcaccaagagacaaccaaaccaaattaagaagactacttacgctcaatcttctaaattaagagaaatcagaaa

aaagatgattgaaattatgcaaagagaagtttctaacgttactttggctcaattaacttctaaattgatcc

>Hypothenemus_hampei_contig_14913|c0_g1_i1 eukaryotic translation initiation factor 5A

aattggaagatttatctccatctacccacaatatggaagttccaaatgtcagaagagaagaataccaattgattgacatt

gatgatggtttcttatccttgatgtctcaagatggttctttgaaggatgatgtcaaggccccagaaggtgaattgggttc

taaattacaagaagaattcgatgaaggtaaagatttactcgttaccattttgatggctatgggtgaagaagctgctattt

cctacaaggaagctccaaagggtgcttaaatctccttgaattaattaaaactttcaaaatcagaatttttttttgaattt

tatattcttttttattagctggagatgaaatcttttatatatattgaaggatgtgtattaaattttttagatccaataaa

cactccgtt

>Hypothenemus_hampei_contig_14935|c0_g1_i1 heat shock SSA1

tgaagtcacctttgatgttgatgcaaatggtatcttgaatgtttctgcattagaaaaaggtactggtaaaactcaaaaga

ttaccattactaatgataaaggtagattatctaaagaagatatcgaaagaatggtttctgaagctgaaaagtttaaagaa

gaagacgaaaaagaggtggccagagttcaagctaagaacgggttggaatcttacacttatcaattaaagaactctatcaa

cgatggagaattgaaggataagatccctgacgacgataaagaaaaattgaacaaggccgtggaagaaaccattacctggt

tagatgaatctcaacaggcagctactgatgaatacactgataagcaaaaggaattagaagcaagtgctaatcctat

>Hypothenemus_hampei_contig_14935|c0_g2_i1 heat shock Hsp70 family (SSA2)

tgaagtcacctttgatgttgatgcaaatggtatcttgaatgtttctgcattagaaaaaggtactggtaaaactcaaaaga

ttaccattactaatgataaaggtagattatctaaagaagatatcgaaagaatggtttctgaagctgaaaaattcaaagaa

gaagatgaaaaggaagccgctagagttcaagccaagaatggtttggaatcttatgcttactctttgaaatcttctttaaa

tgaagaacaaattaaatctaaattagaagctagtgacattgaagaagttactaaggctgccgatgaaactatcgaatggt

tagatgctaaccaaaccgccaccgaagaagaatacgctgaccaccaaaaagaattagaaggtaaagctaacccagtcatg

gctaaagcttaccaagccggtgccgctccaggtggtgctgctccaggtggtttcccaggtgctgacggtg

>Hypothenemus_hampei_contig_14951|c0_g1_i1 60S ribosomal L25

gctgcatccgctaaaaaagctgtgttaaaaggtaccaacggtaagaaggccttaaaggttagaaccaacacttctttcag

attaccaaacaccttgaaattagctagaaacccaaaatatgttagaaagagtgttcctcactacaacagattggacaact

acaaaatcattgtctccccaattgccagtgaaactgccatgaagaaagttgaagatggtaacactttggttttccaagtt

gacttaaaggccaacaagcatcaaatcaagagagctgttaaagaattatacgaagttgatgttgatcatgttaacacttt

ggttagaccaaacggaactaagaaggcttatattagattaactgctgatcacgatgctttggatattgccaacagaatcg

gttacatttagtgtataaggttaatgagtc

>Hypothenemus_hampei_contig_14952|c0_g1_i1 hypothetical protein ECU11_0520

gccgtacagcgggtgggtgtctgacgtggagttccttatgaacagatacttcttcagttctgagttcaaccatgtcaacg

gattcaacatagaggagtcggtgtacgagggatacaggtttgtggagagcaacgacgggaagttccacctttcgtatctg

ccatatcccgggtactacgacgtaggcaagggagcgaaatgcttctgcccggagtgtgggctcatgctgcctcaatgcct

ggaggcagatggcacgtgccttctggacagggccgatggccgtgacgaaaaggagagcaaggagtcctaaggaggtacaa

gtctatgagtttagtacaaccgaggtggccattaaaagatcgg

>Hypothenemus_hampei_contig_14955|c0_g1_i1 Mitochondrial membrane fission

tattcagctctcattttatttttgaacatgggaagaagtccagaaatcgaggcagccttccggcctcttcgagccggtga

gctccaagtccttcgatcacaatatgagaaagaaggcatgtatgccggagttcagacaaaatttaactacgcctggggct

tgatctgcgaagatacccgatctgatcaacaagaaggcctccgcctgttagccgagatcttcagggctgccccggaacgt

caacgggaatgtctctggtatatggcgttggccaactacaagctcggtaactatggtgaatctcgacgactgaacgatgc

gctccttgagcatgaaggcaaggat

>Hypothenemus_hampei_contig_14985|c0_g1_i1 Zn finger

acagccggcttttcgacgagatcaggacacgccaggcccctctgtacagtgacaggatgcctcgaggggccgaaggcatg

tcctccgatcctgggttctccctcggaggatacggctttgggatgagcccagagggctctggcagatcccacggcgtagg

cgaactatacccatttaatcaccagtggacagataggatcatggatgggatactcaagagcatatacactagcgacggga

agcccgaggatcgagacgcaaggagagacgccatcgactcctcaagggcagcaaggggaaggcacatgaaatattcgggc

agcctgaaggaccgtccttttgtgtgcacatacagtgactgcaagcgcgcattcaagaggtacgagcatctcaagcgcca

caacctgatgcacaagatcgg

>Hypothenemus_hampei_contig_14990|c0_g1_i1 ATP ADP translocase

cagtcgatggccccggcgaaatactcggcggggaaaagcaccggcacttcgcacaggtcagctctcttagaaaggtaagg

cacatcagccaggacatcaaggcaaatctgctcttcaacctgctctacgcccttctttcgctcgtggatgcatttctgta

cattctgggcgatatggtcattatgaacacgcaaattcctagctcgatattgttcataaagtctgtgttagtcttaccgg

tgactttcttctccagcgccgttgtgcagaggggattgaagtttcttagccagcctcgcatgcttgaagccattctcatt

gtctcgtccgtgttctttctgttcttcggatttgtcatatggccctacagcaaaagactccagcctgactccttctggtc

gagagacatatttggcgatgggaagatgaactccaggcatctggacttcttctttccgatcttccttgtctttaacgagt

ggatctcgacggtgctctaccttgtggcagagctgtgggggtccttgatcatctctttc

>Hypothenemus_hampei_contig_15028|c0_g1_i1 ubiquitin-like domain containing

atattccagaagtacggcattcccatcgactcgcagcacatagtgttcaatggaaagcactatgtcctgggcatattcga

ctctccccttgttcccgccgctccaaacacatgcagcaacccctttggggcctttggatgcccagacaccctcttcaacg

cccttgagagcagcggaacgactattgaccaaatcagagcgtcggtattcccccacaaggaagagcagaagcaacaggag

gcgcctgaccagcctgttgtcgtgaattctgaggctatggcaacaagactctctacatcccagtacaaggagctcgggga

gatgtccttgagccttgccgaggacgagaaagctgcaatcatgaaaggcccaagcgagtacatctgtttaatcctgagag

ccatgggaaccctcaaggccttcccaccctacacattggagttgtctagtgacgagattgccagcgacacgatcgcgtc

>Hypothenemus_hampei_contig_15032|c0_g1_i1 transcriptional repressor

aacagcaacaacaacaacaacaacaatatcataaatcacttagttctactccaccaacatcaactaccagtataagcaat

tactatccaactcctcaatcgagtacatctagcgctagttcaaaccatttccaaccacaaccaattcattatatcccatt

acaaatacagccaattcctcaatattaccctcaaaaaaatacatccaatgtgagcgtatcacctaaaacagtacttgatc

aaagttttgaaatccccactccaccaccatcatcaacggattattcaatacgatcatttactccagaaacaccaactaca

aataaggataaagttaccaaaagaaaacatatttgtaaaacctgtactagatcatttaccactagtggacatttagcaag

acataatagaatccacactggggaaagaaaacacatatgtccctggccaacttgtgatgctagatttgcaagacaaga

>Hypothenemus_hampei_contig_15060|c0_g1_i1 60S ribosomal L3

agctggtcaaaacggttaccaccacagaacctctatcaaccacaaagtttacagagttggttccggtgctgatgaagcta

atggtgctactgaatttgacagaaccaagaaaactattaacccaatgggtggtttcgttagatacggtaacgttaaaaac

gatttcgttatggttaaaggttctatcccaggtaccaaaaagagagttgttactttaagaaaatccttatacgttgacac

ctctagaagagctaccgaaaaggttaacttgaaatggatcgataccgcctctaaattcggtaaaggtagattccaaaccc

cagctgaaaaacacgctttcatgggtactttaaagaaagacttagaaaactaaattagtcctgacttttagttggtttta

attttatctttctatatctatttttaaagtataaggggctctgtagttatctagtttag

>Hypothenemus_hampei_contig_1507|c0_g1_i1 hypothetical protein ECU08_0960

cccgggcaggagggttgcaaggcgtgccagagacattgtcgatgcagttacagagctccttaccactagagcagcccctt

ggaatatccctgaggccagaggcgtcgtgcctcggtgccccgacgaggcatttgtcgtcaaggaggcgcggaagataagg

ccgagcccgggatgctctggctgtgggtacatcgaaatgcccacgctcagctcgatagagggtgctcgtgactctctctt

gcaccaggatgtttacgagcctctggacagcacatcctatgacggcatggttgggtgcatggagcatgggcaggacttcg

gtgaatgtgcatccggaagcagggcgaggagggcaaggatgttctacagactgctcgagatgagctcgctgggagaggtc

tttcccatccaaagccgtccatacggcaggatagtccttgccatgcctgatgcacacacggacagccatgctgggtttgg

taggacttaatttatcagggtctgat

>Hypothenemus_hampei_contig_15090|c0_g1_i1 rad50-like DNA repair

gaggaaaagaaggtgaagctggttgcgcagaggtccctgctgctgggggagtgcaagcagattgcgcttgggatcaaggc

gtgcaagcaggagcttgagaaggaccacggagccactgtcgagaactataacaagtgctttattgaggtcaaggccctgg

agatgtcctgtatggacatcgacaagtgcatccaggcactcgacagagcaatcgtcgacttccacacctcgaagctggag

gaggtgaatgcaacgctcaaggacctgtggacaaacacgtacagaggaagcgacatcgactggatagagatcaggacaga

gagctgtgggctgaagacatacaactacaaggttgtgtttgtcaagggaggtgcagagctcgacatgagggggaggtgca

gtgctgggcagaagatggtggcaagcatcctcataaggcttgcgcttgcagactcctttgcaacaaactgcagcgtgctg

gcgcttgacgagccgaccacgaacctcgacagagacaacatagagagccttgcattcacgctgtcgcgagtgatatcgag

gcacaagaagaacaaggatttccagctgattgtcatcacccacgacgaggactttgtccagctgctgagcagggacgggc

ccgagtacttctacaggctgtccagagacaagaacgg

>Hypothenemus_hampei_contig_15117|c0_g1_i1 X-prolyl aminopeptidase 2

ggagcggttgtgcaccacaaggcaggcagcaggcggcttctgagagacgaggtcatcctgatcgacagcgggtcgcaata

catgtttggaaccacggataccacacggacattgcactttggagcccccagctccgaggagaaggagagctacacccgtg

tcctcaagggccagctaagggccatgagggccaggtttgggtcgcgcatgccggcatcaacgctggactcgctgtcgagg

atggacctatggagcaagaagctggactacgggcatgccactggacatggcgtggggcacttcctgtgtgtgcacgagtc

tccgccgtctgtgtcgtacttgaacgagtcgcctctgaagcccgggcaagtattttcgatcgagccggggttctacaagg

agggagagtacgggatccggatcgagaaccttgtgcatctgaaggatgccggagacaagttctacgagctggagaacctg

acgcttgtgccgtaccaccttaagcttgtggatatgtcgatgatggacgacgaggaaataagctacttcaacaaggtcaa

cagcaagatccgggcagcgctcgagcctctgatgaggggaggcgtggggtacagatacctgagatcg

>Hypothenemus_hampei_contig_1512|c0_g1_i1 hypothetical protein ECU02_0160

cagggaaggctgaggaaggagagtcgtgcgaatctgaggaaaaagaggcggacatacctcatggccagaggaggactagg

gacttagccaggtatttcgatagaagttcaagctcctatggggcctcggggctaggaaaggaggacgaggcgttgatgga

ggagaagattcgagagcagttgcaaaagatcatttcttacgaatcccacggattgagaagaaggcctgaggacttgccga

ttccagctaggatgtcttatgaggacgagagaggggcgggaagcaccccatccaagatcctgttttcaagcaagtcgata

ccagcaaagagcatgcctaagatggccaagagcctgtcgt

>Hypothenemus_hampei_contig_15121|c0_g1_i1 ribosomal 60S

tttactgcttaaaagtgattattctagaatattaaaaaatgaaatacttagctgcttacttattattggttcaaggtggt

aacactgctccaactgcttctgacatctctgacttgttgtctgccgttaactctgaagttgatgaaaccagagttgctaa

cttgttgaaagaattagaaggtaaagacgtccaagaattgattgccgaaggtaacaccaagttggcttccgtcccaactg

gtggtgccgctgctgcctcttccggtgctgctgctgccggtggtgaagctgctgctgaagaagctgctgctgaagaagaa

gaaa

>Hypothenemus_hampei_contig_15232|c0_g1_i1 40S ribosomal S17

gcggtcccgtccgcggtatctcgttcaagcttcaggaggaggagcgtgagcgtaaggaccagtacgttcctgaggtctcc

gctctggatgtctcccagaccgagtccggccagctcgatgtcgacaccgacaccaaggacctgctcaagtcccttggctt

cgacaacctcaaggtcaacgttgtcgccgtcagccagcagcagaacgccgagcgcccccgccgcttcggtccccgctaaa

ttccgcgctctggataacccccgacaaattgtcaaaaaagttctttgtctacggccgactcttggcgtttactacgacga

cacttattctggttggctctgttcccggctattacggttttagtctagattg

>Hypothenemus_hampei_contig_15232|c0_g2_i1 40S ribosomal S17

acaactataccgccaacatgggtcgcgttcgcaccaagaccgtcaagaggtccgctaaggtcgtcatcgagcgctactac

cccaagctcacccttgacttcgaggtcaacaagaagatctgcgatgaggtggccatcatcgccagcaagaggctgaggaa

caagatcgccggatatgccacgcacctgatgcgccgtatccagcgcggtcccgtccgcggtatctcgttcaagcttcagg

aggaggagcgtgagcgcaaggatcaatacgtccccgaagtctccgcccttgacttctcccagaactccgagactggcaag

cttgatgttgaccaggacaccaaggatctgcttaagagcattggcttcgactccgtccccgtcaacgtcgttgccgtcgc

ccagcagaatattgccgagcgcccccgccgcttcggtaaccgccagtaaacatctctttttttaatcgcacctgggaatg

aaaaaaagaaaaggatgatccggacgaaagcttgagcggatggaaagaagacgttgtgctagcgttga

>Hypothenemus_hampei_contig_1524|c0_g1_i1 60S ribosomal L19

ggcgccctgcctgtgtgcggagaggccgaggcacacaaaacaccgaaaaatttgcaccccgaaaatgtaagtgggcaccg

gtctaatttgaaggtcgaacgaggagagagtcaggcggctggcagcagatatcctgaggtgtggcaaggccaaggtgtgg

ctggacaacaacgaggtcgagaggctcaatgcagtgaccaccagggagcaggtgcggcagctgattggcgacaacgtgat

catcatgaagcaggacaagcacaacagccgggggcggttcagagagcgccttgcggcaaagaagaagggcaggcacatgg

gccctgggaagcggcacgggacggcaaatgcaaggatgcccgagaaggtcgtgtggatgaagaagatccgtgcgatgagg

gcgatgctcaaggagatgcgtgccagcggagagatcagcaaggaggacttcaggacgttctacatgcaggcaaaggggaa

cctgttcaagcacaggttcttcatgaaggactacatcatcaagaggaagacggaggagcagagggccaaggagcttgcgg

agcaggccaaggcactgaacctggcgggcagggcagcctcgtcctgaaataaagagtcttt

>Hypothenemus_hampei_contig_15261|c0_g1_i1 hypothetical protein PGUG_00423

gcctagatccttcccttataatggtttggctaagactttgggttcgggtagtgaagaagacgcaaaagtctaccgtgtca

agccagataccactgctgaacaagaattgggtgtcttgggtgctaatgacgaaattaaaaagcaacaagaggcttacttg

caagctaactctgattttatcaaagcttaactccttgaaaagaattatatcccctttgtacattacaaccactttgcaaa

agtagcatccattgccactcctttgcaaaacctttactttagcagaggctccatcaatataaaatgatcaaaaaaaaatt

gaataaaaaaaaattgaaaa

>Hypothenemus_hampei_contig_15300|c0_g1_i1 DNA lyase endonuclease 4

tcaactttggaaaccccgtgctgatcgacaagagcatggagtgcctggtcgacgacttggagaggtgtagggcgctgggg

atcccgatgtacaacatgcatcctggaagtaacaaggagaagagcaaggccgcatgtctgaggagcatcagcagacacat

aagcacggcgctctcgagggttggcggcgttaccatcctgatcgagaacatggctgggcagggaagcgttgttggaagca

catttgaggacctgagagacatcatctgcggggttgaggacaaggagagaataggggtctgcctggacacatgccacctg

tttggcgcggggtttgacatccggacagaggagggctttggcgaggtcatgcggaggtttgacgaggtggttgggctgag

gtacctgaaggccatgcacatcaacgacagcaaggagccgcttggttcaaggaaagacaggcatgagtccattggacgcg

ggcttataggcgaaagggcattcaggtttgtcatgaacagcagtgtgtttgacgacatccccctgatccttgagactccg

gacccagaaagatacaaggaggaggctatctttctaagagg

>Hypothenemus_hampei_contig_1537|c0_g1_i1 hypothetical protein ECU09_0040

gtgtttatgctctcttactggataaggagaaactggtgtgtgaatctttcctctggggattcaaagcagtatctgatcta

ctgcactctgattgggctatgcacagtggggctggttgcatgcctcgcagaaacgtttcattatggcctctatgggggct

acgttgatgttttgaaaacgtttgcgacattctcctctataagctacataaggttcagatgcctggggctcttgccaaga

cctgtttaccgatgcagtaaccttggaaatgcagtctcgatcattgcaatcattgtaataacctgtgcagtttgtgtgca

tgggaagaatagtgcagatgtggctgcatggagctttgtcgtgagtggggctatgctagc

>Hypothenemus_hampei_contig_15376|c0_g1_i1 60S ribosomal L8

ggttctagcaagaagggccccaagaaccctctcatcgagaagcgcccccgcaacttcggcattggccaggacatccagcc

caagcgtaacctgtcccgcttcgtcaagtggcccgagtatgtccgtcttcagcgccagaagaagatcctgaacatgcgcc

tgaaggtccccccttcgctggcccagttccagaacaccctggaccgcaacaccgctgcccagaccttcaagctcctctcc

aagtatcgccctgagaccaaggccgagaagaaggtgcgtctcgtccaggaggccaccgccgtctccgagggcaagaagaa

ggaggatgtctccaagaagccctaccacgtcaagtacggtctgaaccacgttgtcggccttgttgagaacaagaaggctt

ccctcgtcctgatcgctcacgacgttgaccccattgagctggttgtgttcctgcccgctctctgccgcaagatgggtgtc

ccctacgccatcgtcaagggcaaggcccgtctcggtaccgttgtccacaagaagacctccgccgttctcgctctcactga

gacccgctccgaggacaaggctgagttctccaagctcctctccgccatcaaggagggctacaccgacaagtacgaggagt

cccgccgccactggggtggtggtatcatgggcgccaaggccaacgcccgccaggagaagaagcgcaaggccgttgagaac

gccatccgcgtttaaagctttaaatagcttaggacgggaatccg

>Hypothenemus_hampei_contig_15376|c0_g1_i2 60S ribosomal L8

ggttctagcaagaagggccccaagaaccctctcatcgagaagcgcccccgcaacttcggcattggccaggacatccagcc

caagcgtaacctgtcccgcttcgtcaagtggcccgagtatgtccgtcttcagcgccagaagaagatcctgaacatgcgcc

tgaaggtccccccttcgctggcccagttccagaacaccctggaccgcaacaccgctgcccagaccttcaagctcctctcc

aagtatcgccctgagaccaaggccgagaagaaggtgcgtctcgtccaggaggccaccgccgtctccgagggcaagaagaa

ggaggatgtctccaagaagccctaccacgtcaagtacggtctgaaccacgttgtcggccttgttgagaacaagaaggctt

ccctcgtcctgatcgctcacgacgttgaccccattgagctggttgtgttcctgcccgctctctgccgcaagatgggtgtc

ccctatgccattgtcaagggcacggcccgtctcggcacagtcgttcacaagaagacctccgctgtcctcgctctcaccga

gacccgtgctgaggacaaggccgagttctccaagctcctgtccgccatcaaggagggttacaccgacaagtacgaggagt

cccgccgccactggggtggtggtatcatgggcgccaaggccaacgcccgccaggagaagaagcgcaa

>Hypothenemus_hampei_contig_15422|c0_g1_i1 60S ribosomal L11

agtcgccatgtcggacggaaaggacaagagcgcaaaccccatgcgcgagctgcgcatccagaagctcgtgcttaacatca

gtgtcggagagtctggtgatagactcacccgtgctgctaaggtgcttgagcagctgagcggtcagactccggtctacagc

aaggcccgttacaccgtccgtaccttcggtatccgtcgtaacgagaagatcgccgtccacgttaccgtccgtggccccaa

ggctgaggagatcctcgagcgtggcctcaaggtcaaggagtacgagctccgcaagcgcaacttctccgagaccggtaact

tcggtttcggtatctccgagcacatcgatctgggtatcaagtacgaccccggaatcggtatctacggcatggacttctac

tgctgcatgacccgccccggtgagcgtgtcgctaagcgtcgtcgtgcgaaggcccccattggtgtcaaccacaagatcac

caccaacgagaccatcaagtggttcaagaaccgcttcgacggcattgtccggtaaatgcttttaacgagtgttgaggaaa

tgggatggagatccggttgaaactgtgatttacgatgcatcgaagggagccaaaaattcttctccgaaacaaaaaatcgg

cccaggacggaagaccccgtcgaacttcggccgggctcccggaccctgcgtcggcaatggtgttcttgaaaccctttctc

ttattttttttttatct

>Hypothenemus_hampei_contig_15422|c0_g2_i1 60S ribosomal L11

agtcgccatgtcggacggaaaggacaagagcgcaaaccccatgcgcgagctgcgcatccagaagctcgtgcttaacatca

gtgtcggagagtctggtgatagactcacccgtgctgctaaggtgcttgagcagctgagcggtcagactccggtctacagc

aaggcccgttacaccgtccgtaccttcggtatccgtcgtaacgaaaagattgccgtccacgtcaccgtccgtggccccaa

ggctgaggagatcctggagcgtggcctcaaggtcaaggagtacgagctccgcaagcgcaacttctccgagaccggcaact

tcggcttcggtatcagcgagcacatcgatcttggtatcaagtacgaccccggcattggtatctacggcatggacttctac

tgctgcatgactcgccccggtgagcgtgtcgccaagcgtcgccgctgcaaggcccgcattggtacccagcaccgcatcaa

ccagaacgagaccatcaagtggttcaagaaccgcttcgacggcattgtccggtaaatgctttgaacgagtgttgaggaaa

cgggatggagatccggttgaaactgtgatttacgatgcatcgaagggagccaaaaattcttctccgaaacaaaaaatcgg

cccaggacggaagaccccgtcgaacttcggccgggctcccggaccctgcgtcggcaatggtgttcttgaaaccctttctc

ttattttttttttatct

>Hypothenemus_hampei_contig_15422|c0_g2_i2 60S ribosomal L11

agtcgccatgtcggacggaaaggacaagagcgcaaaccccatgcgcgagctgcgcatccagaagctcgtgcttaacatca

gtgtcggagagtctggtgatagactcacccgtgctgctaaggtgcttgagcagctgagcggtcagactccggtctacagc

aaggcccgttacaccgtccgtaccttcggtatccgtcgtaacgaaaagattgccgtccacgtcaccgtccgtggccccaa

ggctgaggagatcctggagcgtggcctcaaggtcaaggagtacgagctccgcaagcgcaacttctccgagaccggcaact

tcggcttcggtatcagcgagcacatcgatcttggtatcaagtacgaccccggcattggtatctacggcatggacttctac

tgctgcatgactcgccccggtgagcgtgtcgccaagcgtcgccgctgcaaggcccgcattggtacccagcaccgcatcaa

ccagaacgagaccatcaagtggttcaagaaccgcttcgacggtatcgtccggtaaatgttttttttcattgatggggttg

tcaaatgggggaaagccttcggcttgtgagtacgatgaatcgattaaaaacggcagaccggcgttctgcttcga

>Hypothenemus_hampei_contig_1548|c0_g1_i1 prolyl-tRNA synthetase

ggtatgtgcaggtgatcacaaagggcgagatgctggactactacgagatcaagggatgctatgtgatgcgcccacagggc

cagtttgtctggaggtgcatccagaagtggtttacagagaggatcgaggcgctaggcgtgcaggagtgctacttcccgat

gctgatccccaagtcgatgctggagagggagaagagccacatcgagaacttctctccggaggtggcgtggatcacaaagt

gcggggacgaggttctggaggaccctgttgcggtgcgcccgacgtccgagacgattatgtatccgtcgtttgcaaagtgg

ataaggagccaccggg

>Hypothenemus_hampei_contig_15540|c0_g1_i1 similarity to replication factor A 3

catttgcggttgggggtggtttttgcgatttcatatggtgtttggcggcccccatgtattctcaagatgtcaaggaccgc

gagggccaggacaccgtggtgatagggcggttcgagagggtcgaggacggcgttgttgttctgaactgcatggggaccga

ggtccaggtgaggcactgcaacgtcgagtcgtacaagtctgggctggtcagggtctgtgggcttgtggagaacggggtgc

ttgttgaaagcagcgtccatcccatcgagggagacttcgaccttgcgacgtacaagcggtttgtggctgctgcgtcgaga

tacccaggcctgttttgacgccgatggccctgttcagctgcatcctctactccagagccttat

>Hypothenemus_hampei_contig_15546|c0_g1_i1 DHHC zinc finger domain-containing

ttgaagctgttacactttctacagaaggcctttggagggcagcggtccagtaggcctacggcttctggatccgatttaac

acgacctctgtaagctatagagagggccagatagatcatagacagcacgcctgatgctagcagcaacacgacgtaggtct

gcttgaatgcaaagtcctctggcaggatgattttagatactggagcggtgtacgtgaatatgagcgaggaaatcagcaat

gtcattctaaaattcggccttattccacccatcccgagggtttatttgtttctggttttagagctggatgcatatgagca

agatgaaacctcatcggaaactcattaaacaaccagccttctagaagatgcgggtgtggaggcggacaagctctttgtgg

tcgtctgcccagaacgggcctactaactcgtaagaatgaagggtcctgctgccctctcttacagcatgcctcttcctcag

cttcacggagaacaaaattccgttttcaaacgacctctccgaaagaataaaagggatgcagagggatggggagaggggga

tgttg

>Hypothenemus_hampei_contig_15547|c0_g1_i1 hypothetical protein CANTEDRAFT_114342, partial

taatcctcatactgttgaagtagtaccagaaaacaaattgaaggagtttgaacaatctaaagaagataaagtgaaatcta

aagctaaagaacttggtaaagaattaaacaaagaagctgagcacttaaagaaggaaggtaatgatttatttcaagcgatt

tttgcatctttgaaaaaagggtttaaccaagctagtgattcaatccataaaatcggtttaaagacccgtgatactaccaa

agatttgatcaaggtatcatcaaccgaaattaaaaatcctgttgttgtaattcaagggttggtatttgctaccggtatcg

ctggagcttattttgcttatttggaaa

>Hypothenemus_hampei_contig_15548|c0_g1_i1 26S proteasome regulatory complex component

aggaagtacttcagcgaaagcggcattgtatccgagaaggtcaagttccacgagctgattgtgctgctccaccttgggca

gagaaagtactttgatgcatccgaggtctactacacccttggggaggttggggaaagcgccaccaagtacgttgtcttga

gctccttcttctgcatcctgacgacgtgcgagacagagatgtccgacgtggtgggcaaaaggacagagatgctccggaag

ctcagcgaggataagaacaacgacgaggctgccaggtcggttgtaaatagattcctgagcagggttgtgatagacaagtc

gatgatcaacgagatccaagagacgttttcgcctgtcctggacgtctcggcgtacttgaacgaccttgttgctgcaatcg

atgagcacaacttcaggattgtcgagaggttctactcgtcgatcagcatccaagagatctcaatggtgatgcaaagcgcc

ccggaggacgttgtcaagaagatctcgttcatggtcaactctgggttcacaggatgccggatcaaccagaacacagggat

catcgagctccagaagcggcaatggaacgacgacgtcgaggacgtgatggccaagctgatcaagtgcaaccacctcatac

acaaggagaggctggga

>Hypothenemus_hampei_contig_15569|c0_g1_i1 dienelactone hydrolase family

cgtcgacgaggccaacaagctcgcgcccgccgggaagcccttcgactggtccatcctcgggtactgctggggcggcaaga

tcgcggtgctggcgaccggccaggagaacaagacgttcaaggccgccgtgcagtgccacccggccatgctggcgccggat

gacgccaagtccgtgaatgtgcccatggcgctgctggcgtcgaaggacgaggacccgtccgatgtgacggcctttgggga

taatctgtcgaagccgcactatgtggagacgttcactaaccagatccatgggtggatggcggcacggtcggatttggaga

atgacgaggttcggaaggagtacgagaggggatatcgcactgcgttggacttcctgcaccagcatgcttaggtggacggt

ttcctgtctgaaaagtgcgatttctgttctatcgctaacagattgaacctgactcttgaggtctgaatagtagataggta

atgaattatgttat

>Hypothenemus_hampei_contig_15574|c0_g1_i1 deoxyhypusine synthase

cttgtgacaacaggagggggaatcgaagaagacctcatcaagacgttcaagccgacgctctgtgcagatttcgggcttga

tggagaggagctgcgaaataacggactgaacagagtaggaaatcttgttattccaagcgagaactacgaggattttgaga

cgtggctgaaccaagtcgtgaatgacgtgacagagggatacacggaggaccggccaaggatactgacgccctcgtcgttc

ataaggatcctgggggaaaggatagacaacgagtcatccatcctctactgggccgcaaagaacaacataccggtatactc

tccggcggtcgtcgatggctcgatcggagatatactatcgttccatccaaggagaaagctgctaaagctggacatcgtcg

acgacgtgtaccggatgaacatggaggccgtgtgttccgaaaagactggggccatcatacttggatgcggcgttgtaaag

caccatattctgaatgcaaatctgttcaggaacggggcagagtactgcgtcctgatcaacaatgcccaggagttcgacgg

cagcgacgccggtgccagcctagatgaggctgtcagctggggaaagatcaagcccgaagcaa

>Hypothenemus_hampei_contig_15603|c0_g1_i1 DNA replication licensing factor Mcm7

atatcaagaagattctattgctaatgcttgtcggagcgccgtcaaggacacgagaggacgggatgagaataagaggagac

ataaatgtgttgcttgtcggggatcctgggattgcaaagagccagctactgaaaacatgtgtcaagataagccgcagagg

agtgtatacgactggaaagggatcgagtggagttgggctgacggccagtgtcaccaaggatcaggtaactggagagatgg

ttcttgaggggggagcactggtccttgcagacgggggaatctgctgcatcgacgaactcgataagatgaatgaggttgac

agaatcagcatcca

>Hypothenemus_hampei_contig_15623|c0_g1_i1 60S ribosomal L10a

tggtgatgctttcgatgttgacagagctaaatctgttggtgttgatgccatgtctgttgatgacttgaaaaaattaaaca

agaacaaaaaattaattaaaaaattagctaagaaatacaatgctttcattgcttctgaagttttaattaaacaagttcca

agattattaggtcctcaattatctaaagctggtaaattcccaactccagtttctcataatgatgatttatactctaaagt

cactgatgttaaatccaccattaaattccaattgaaaaaagttttatgtttagccgttgctgttggtcacgttgaaatgg

aacaagatgtcattgttaaccaaatcatgatggctgctaacttcttggtttctttattgaaaaagaactggcaaaacgtt

ggttccttagttattaaatcaactatgggtccatctttcaga

>Hypothenemus_hampei_contig_15635|c0_g1_i1 mRNA decapping enzyme 2

ttcgtcttcaacgtcaaggccgactttccattccaggcacagactcggaaggaaatcgaggagatcagatggctctcgat

aaaaaggctgtcaagaggcgaatacaaaagagggtactcgatcgtgtctacggcattcaaaagagcctcttacctgctag

aggctatcaggaaggccaggttcaagttcaacacagagaagatcgtccagagaatagatggattgcttaatagtggcaag

cagaaggtacaaaccccctagagatgcagatgtcggagccaaaagaggtttaatgcaaaggtagccggaaacccgccatc

tcatcacagaaagaaccgtagcaaaaagaacagcataacaagcagaacaattcctcccataaagagcatcctgtccgata

aatatc

>Hypothenemus_hampei_contig_15636|c0_g1_i1 cyclin-dependent kinase

gtcatgcccaggttctcccccgtcgagaaggtcggcgagggctcgttcagcaccgtctacaaggccctagacacagacac

gggccgctacgtcgccctcaaggcaatcacacgcacaagctcgccggcacgggtcctggacgagctgacgttcctcaaga

cgcttggcggcaggagaaactgcatggggctggtggggtgcctgcgcagcgaggaccaggtcgttgccgtgctgccgtac

ttcgaggccatcgacttccgcgagttcatctcgaacgcaaccacggcagacgccaagcagtacctgtacagcctgctcgt

ggccatcgaccacgtccacagcaacggcgtgatccacagagacctcaagc

>Hypothenemus_hampei_contig_15640|c0_g1_i1 hypothetical protein ECU08_0430

ataaggaaggtcacaaagggagtgaaggtcaagggagtcgagataagcttcaaagggctgttccagacaatccttatctt

cattccaatgacgctggggtttgcaggccttggaaagacattgcctgctcaggtcatagcgataatcgtgtatctgatcc

caatagccatttcgtccatccagatcatccgaggggtcgtgaggacctatagagaatgcagaagggatggggcttcaaag

ctcctgagattcagaaacctgcttttgctgcttcagatgatcaccattctcctcctggcgtcgcacctcatctacatagg

aagtgagaatcgtgcaaagaagctgggaagctacagcctgatgatctccgtcgcatgtctttcccagctgcccgaaaccc

tgtcccggttctcgatcgagagcgacgacctccacaagatcctcattccgctgcttgcatctgccctgctgctcatcatg

accctcctcacccggaatagcgacaccacaggcccggcatacaccgtcatgtgcgccctgctttctgtcgtggtcatatt

gagcgatgccatcggaaagaagatctcctccgagccaagggacttagagccgctacctg

>Hypothenemus_hampei_contig_15676|c0_g1_i1 zinc finger A20 and AN1 domain-containing stress-associated 5-like

catattttcattttcgttcaaaacattttctctctaccttctcgaattagaaaggaaaaaaaaaagaaaagcaaaaagag

gggaaaaaaacgaaaaaggaaaaccttggccatggctcatcagaagagagagaaagaagagacggaactcaaagttccgg

aatctctaacactatgtaccccaacaatcccagtacctccaccgcagctcgatgatccgagatctaattctgcgagatcg

cctgagagctcagatccgaaactcttggtcaacggggacaacaatctaagatctagcccgttgtcaccggagagatcgga

tctgatctctgtcggagctgtcgatggaaaggcggtggaattaaatcggtcgttgaaaaggccgagggacgct

>Hypothenemus_hampei_contig_15677|c0_g1_i1 stearic acid desaturase ( )

attggattggttctcaaccattcgatgatcgcagaactcctcgtgaccatgtcatcactgccttggctacttttggtgag

ggataccacaacttccaccacgagttcccctccgactaccgcaatgccatcgaatggcaccagtatgaccctaccaagtg

gaccatctggtcctggaagcagctgggtctcgcctatgacctgaagcagttccgtgccaacgaaatcgagaagggccgcg

tccagcaactgcagaagaagattgaccagaagcgctccaagctggattggggtactcccctggagcagctccccgtcatg

gagtgggacgactatgtcgagcaggccaagaacggccgtggcctggttgccatcgctggtgttgtccacgatgtgactga

tttcatcaaggaccaccccggcggtaaggcc

>Hypothenemus_hampei_contig_15685|c0_g1_i1 pyrimidine binding domain partial

cgcactgatcctctcccgtcagaacctggcgcagcaggaactaactgaagagcaactggcaaacatcgcgcgcggtggtt

atgtgctgaaagactgcgccggtcagccggaactgattttcatcgctaccggttcagaagttgaactggctgttgctgcc

tacgaaaaactgactgccgaaggcgtgaaagcgcgcgtggtgtccatgccgtctaccgacgcatttgacaagcaggatgc

tgcttaccgtgaatccgtactgccgaaagcggttactgcacgcgttgctgtagaagcgggtattgctgactactggtaca

agta

>Hypothenemus_hampei_contig_15698|c0_g1_i1 alpha tubulin

cattggacaggcgggagtgcagataggaaatgcgtgctgggagctgtactgcaaggagcatgggatcctacctgacgggc

ggcttgagcagggcgggatgcacgacaagagcaccgagccttttttctctcagacgagcattgggacgtatgttccgcgg

acgctgatggttgatctggagcctggggtgattgagagtgtcaagacggggcagtaccgggacctgtaccaccctggaca

gcttgtttctgggaaggaggatgcggcaaacaactatgcacggggccactacactgttgggaaggagatcatcgaccagg

cgatggagcagataaggtggatggcggacaactgc

>Hypothenemus_hampei_contig_15711|c0_g1_i1 pathogenesis-related 1

tgctcataacgcagctcgtgcacaagtcggtgttggcccaatagcttgggacgacacggtggctgcctatgcacaaaact

acgccaatcagaggataaacgactgcaatctggtgcactctggcggccgttacggtgagaacttagctgaaggcagcgga

gacttaacgggcacggatgccgtcaatctgtgggtaagtgagaagcaatactacgattacaactccaacacatgtgctca

agggcagcagtgcgggcactacactcaggtggtttggcgcgactcggtccgtctgggctgtgctagggttcaatgcacaa

ataacggttggtggttcgtgatttgcagttattatcccccgggcaactacattggtcagcgtccatattttgcagttatg

accccccagtctggatgtgtt

>Hypothenemus_hampei_contig_15720|c0_g1_i1 hypothetical protein ECU04_0590

acaaagtgtgtctacaaggcccacagagacgcatggcgtggccacaggcatggcatctcaaagtacttggagatactcaa

ctgctgccaccaggagccaaggagccccctggactgcttgcggagggtcatgaggaagtccaacaagaaccactacatcc

tcgcaagcaactgcgccgagatcacgggcctcatcggcctcgagaggcgcatccccacaatcacatgccaaagaggctct

ttgctgatgactgccgacctccaggccgtcccaatgcacaaagggctcagcacagaagccggccaagaagagctcagtcg

tcttgaggccctgtttcccagcgaggacaaggaatagaccacatccacagac

>Hypothenemus_hampei_contig_15768|c0_g1_i1 DNA-directed RNA polymerase subunit beta partial

tgatggccgactccggtgcgcgtggttctgcggcacagattcgtcagcttgctggtatgcgtggtctgatggcgaagccg

gatggctccatcatcgaaacgccaatcaccgcgaacttccgtgaaggtctgaacgtactccagtacttcatctccaccca

cggtgctcgtaaaggtctggcggataccgcactgaaaactgcgaactccggttacctgactcgtcgtctggttgacgtgg

cgcaggacctggtggttaccgaagacgattgtggtacccatgaaggtatcatgatgactccggttatcgagggtggtgac

gttaaagagccgctgcgcgatcgcgtactgggtcgtgtaactgctgaagacgttctgaagccgggtactgctgatatcct

cgttccgcgcaacacgctgctgcacgaacagtggtgtgacctgctggaagagaactctgtcgacgcggttaaagtacgtt

ctgttgtatcttgtgacaccgactttggtgtatgtgcgcactgctacggtcgtgacctggcgcgtggccacatcatcaac

aagggtgaagcaatcggtgttatcgcggcacagtccatcggtgaaccgggtacacagctgaccatgcgtacgttccacat

cggtggtgcggcatctcgtgcggctgctgaatccagcatccaagtgaaaaacaaaggtagcatcaagctcagcaacgtga

agtcggttgtgaactccagcggtaaactggttatcacttcccgtaatactgaactgaaactgatcgacgaattcggtcgt

actaaagaaagctacaaagtaccttacggtgcggtactggcgaaagg

>Hypothenemus_hampei_contig_15770|c0_g1_i1 Mn superoxide dismutase

acactgccgtcgagcagctgcaggaggctcaggccaagaacgacatctcggcccagatcaacctcaagcccgccatcaac

ttccacggcggtggtcacctgaaccacaccctgttctgggagaacctggcccccaagagcgctggcggcggcgagccccc

ctcgggtgctttggccaaggctattgacgagagcttcggcagtttgggcgagttccagaacaagatgaacaccgccttgg

ctggtattcagggtagcggatgggcctggcttgtcaaggacaagcagaccggcaacatcggcatcaagacctacgccaac

caggaccccgttgtcggccagttccagcctcttctcggtatcgacgcctgggagcacgcttactacctccagtaccagaa

ccgcaaggccgagtacttcaaggccatctgggaggtgatcaactggaagaccgtcgagaagcggttctcgtaaagtgcag

accggacattgactgtacattgcctctgatttgagcgattgatttttggatgggatctg

>Hypothenemus_hampei_contig_15772|c0_g1_i1 eukaryotic translation initiation factor 2

atcggggacaggatcgagatcaggcccgggcttgtgaccaagaggggcggcaagtttgtctgcaggccttttgtctcaag

gattgtttctctgaaggcagagagcacggaccttgacgaggcgtatccgggagggctgataggggtcgggacgacgatgg

acccgtcgttctgcaaggcagacaagctggttgggcaggtgatggggaagctcgggtttctcccgtcgatattccagaga

ataaccgtggagtacagcctcttcccaaagacaacgatgcaagggagctcgaagctgagagagggcgagcacgtgctgct

gaacataggaagcacgacgaccgggagtgtgatagcaaggatcgacgatgcctgtggagacttcgatcttgtgaagcctg

cgtgttgcgagatcggcgagaggattgcaatctcgaggaagatcaacaaccactggcgactcatagggcacggggagatc

aaggacggggtgtgtgtcgagcccgactacgatgttggaatagacgacaagtagcgacgagg

>Hypothenemus_hampei_contig_15780|c0_g1_i1 phosphate transporter (Pho88)

gtcctcatcttgggactgtatctatactcgcaggccaagatcaacagcaagaaggacttgactacgctgaagtatgtcga

gcccgctcccatgggcagcaacgaggagccccgtccggtcacgaccaccaacatggagtacgacaagggacagttgcgcc

atctgattaagggccaactgatgggtgtcggtatgatgggcgtgatgcacctgtacttcaagtacaccaaccctctcctg

atccagtcgatcattcctctcaagagcgccatcgaatccaacctggtcaagatccacgtcttcggccgacccgccgccgg

cgatctccagcgccccttcaaggctgccaacagcttcatgaacc

>Hypothenemus_hampei_contig_15782|c0_g1_i1 hypothetical protein AC631_05088, partial

ccactactttgggtcatttatgtatgtctttcaatgaacttgctgataacgctgctaatttagctactttattattttct

atgtgcttaaatttctgtggtgttttagcaggaccagatttcttaccaggattttggattttcatgtatagagctaatcc

atttacctatttaattcaaggggttttaagtgctggtttagcaaatactaatattgaatgttctgatactgaagtcttaa

gatttgctcctgctcaaggcaaaacttgtggagaatatatggaagcatatatgaagcaagctggtggttatttgattgat

aagagtgctactgatgaatgtaaattctgtgcaatggcctcaaccaatgattatttagcaactattaattcatactatag

tgaaagatggagaaactttggtatctttgttgcttttattgctatcaacatgattcttactgttatcttatactgggctg

ctagagttccaaaaggtaatagagagaagaagaagacaaaaacaacgtaatcatttattcatttcaaattcttttacttc

ttctttaattttatgattaat

>Hypothenemus_hampei_contig_15815|c0_g1_i1 Mannitol-1-phosphate 5-dehydrogenase

cccagtcattgctaagggcatcgaccttcgcaccgcttctgttccattggctgtaattgcctgtgagaatatgattggcg

gcacggacaccttacatggcttcatcaaggacaacaccgattcatcacgagtagagacactcggcgaacgggcctcattt

gccaactctgccatcgatcgtattgtcccggcgcaagccccggatgctggcctcaatgtccgtatcgagaaattctacga

atgggttgttgagcaactcccctttggcaagtttggtcatcccgatattcccgcgatccactgggtcgatcatctagaac

cttatatcgagcgcaagctgttcaccgtcaagat

>Hypothenemus_hampei_contig_15819|c0_g1_i1 30S ribosomal S13P

ccggcagaggtccagtacatcatccgtatccacaacacaaacatcgacgggacaaagcgcattgcatttgctctgacgaa

gattgccgggatgggcgtgcggatctcgacggcaatctgcaagcgccttggaatcgacctgaggaagcgtgcgggagaga

tgtccgaggacgagctcaagcggatcagcgacgcagtggccgaccctgcaagcgtcgggatccccgagtcgtacatgaac

caccgcaaggacgtgcttgacggcacgacgtcgcacctcataggcaccaagctcgacgccgacctgaggatgatgatcga

gagaggcaagaagaacaagcgcattagagcatgcaggctcgacgtcggcctcaaggtgcgtgggcagaggacaaagtcga

acggcaggaacggcaagtccatgggcgtttccaggaagaagtaaaggct

>Hypothenemus_hampei_contig_15831|c0_g1_i1 hypothetical protein M970_040010

aggatgttcagcttccgaaacacacgtctccggaaccatgttctgcatcgcctggaggctgcaaaggcccagagagagag

ggacagaaaggagaaggaggagagaagacacagaaggcagagcagaggagggcatgggagcaagggagacttcctcgggt

actacaaggccatggggttcaaggagggggagaagccgagtgagaaggagatcaagagcgcataccgcaagacggttgtc

aagaacaagaagaggaagaagacagaggccgaggagaaggagtgggaggaggagttcaagaagctcaacaaggcgttcgg

agtgctttccgacaaggagaagcgcgagatgtacgacaagggaatcgacccagacaatccgcagcagttctctgggtttg

gggacgacccgttccaggacgtgttcaggggcttcggcaactttgggttcttcgacttcccagtgggcaggcaaggcggg

aggaggaccacgacgacatatttttatttctg

>Hypothenemus_hampei_contig_15838|c0_g1_i1 class I glutamine amidotransferase

tccaagaatcgttcttgtctggagatgataaagccgtttacgaagatgctaactccgcttacagtgttgcttttaagaac

atcaagaaggcatctgaagtcaatgctgaagattacgatattttctttgctgctggaggtcacggttgttgtttcgactt

ccctaaagccaaggatcttgttaaggctgctgaaaccatctatgctaaaggaggtgtagttgctgcagtctgtcacggac

cagctatttttgataccatgaaggatcctgctactggtaaatctattatcgccggtaaaaacgtcactgggttccttgat

gaaggtgaaaaggttatgggtttagagcctgctttcaccaagtacaaccttg

>Hypothenemus_hampei_contig_15879|c0_g1_i1 Ribosomal S5 eukaryotic archaeal

aatgtccgacaacggggaagttgaggtggctgtctgcccctccctgcctaaggagatcgctgccctcgagggtgaggtca

agcttttcggcaaatggagctacgatgttgaggtccgtgatatctctctgaccgactacatccaggtccgcgctccggtc

tacacctcccacactgccggtcgctatgccgccaagcgtttccgcaaggctcagtgccccatcgttgagcgtctcaccaa

ctccctcatgatgaacggccgtaacaacggtaagaaggtcaaggctgtccgcattgtcgcccacgccttcgagatcatcg

ccatcatgaccgatcagaaccccctccaggttctgtgcgacgccgttgtcaacagtggtgcccgtgaagactctacccgt

atcggttcccagggtaccgtccgtcgccaggccgtcgatgtctcccctctgcgccgtgtcaaccagtccatctcc

>Hypothenemus_hampei_contig_15939|c0_g1_i1 ATP synthase subunit mitochondrial

tccagctccagctactacttttgctcacttggatgctaccactgtgttgtctagaggtatctctgaattgggtatttacc

cagctgttgatccattagattccaaatctagattattggatgtttccgttgttggtcaagaacattatgatgttgcttct

ggtgttcaacaaaccttacaagcttacaaatctttacaagatatcattgctattttgggtatggatgaattgtcggaagc

tgataagttgactgtcgaaagagccagaaagattcaaagattcttgtctcaaccattcgccgtcgctgaagttttcactg

gtattccaggtagattagtcagattacaagacaccatcaaatctttcaaggatgttttggaaggtaaatacgatcactta

ccagaaaatgctttctatatggttggtggtatcgaa

>Hypothenemus_hampei_contig_1596|c0_g1_i1 unnamed protein product

atcgttgaacttctggcatcatgggggaatcatcctttacaattcagattagtaccaatttagttgaccagctagctgat

gatagtggaaagctgaaaaggaaaactaggaaagcaaagccaaagctgccacaggatgctaaaactcctcagcaatccct

ttctaagcaaaagcaggtctctgatgattctaagatccttaaagtgccacctgcagctggatggccgcttcagcctcctc

tatacttgccacctcctcctgtgcaacctgctaatgcagagttagaagcaattcagtctgtcctgaaagagagtgaaaat

gttgtggagaagctgcaaaagcaggagg

>Hypothenemus_hampei_contig_15962|c0_g1_i1 ADP-ribosylation factor

ggcagcttatttaaaggcctcttcggcaaacgggagatgcgtattctgatggttggattggatgctgccggaaagactac

gattttgtacaagttgaagctgggcgagattgtcacgactatcccaactattggattcaacgtggagactgtcgaataca

agaacatttcattcaccgtgtgggatgtcggaggtcaggacaagatccgtcctctgtggcgccactacttccagaacact

cagggtatcatcttcgtggttgacagcaacgatcgtgaccgtattgtcgaggcccgcgaggagctgcagcgcatgttgaa

cgaggatgagcttcgggatgcccttcttctggtctttgccaacaagcaagatttgcctaatgccatgagccctgctgaga

tcacccagcagcttggtcttcagagcctgacccgccgtgcttggttcatccaatccacctgcgccaccactggtgatggt

ctctacgagggtctggagtggctcgccgacactctccggaaaacgaaccgcgattaaacgcgtataatacgaaattgtga

tggggaggattgtgtacgtagcagagcaagagaaataccacgggaaatctgcaaatgatggaatgatgattatggcggga

gtttcttccaatgttcttctgcgaggccaaatatcccggcgatgaaaaagaattccctcaccggcatggcatggccatcc

>Hypothenemus_hampei_contig_15962|c0_g2_i1 ADP-ribosylation factor

ggcagcttatttaaaggcctcttcggcaaacgggagatgcgtattctgatggttggattggatgctgccggaaagactac

gattttgtacaagttgaagctgggcgagattgtcacgactatcccaactattggattcaacgtggagactgtcgaataca

agaacatttcattcaccgtgtgggatgtcggaggtcaggacaagatccgtcctctgtggcgccactacttccagaacact

cagggtatcatcttcgtggttgacagcaacgatcgtgaccgtattgtcgaggcccgcgaggagctgcagcgcatgttgaa

cgaggatgagcttcgggatgcccttcttctggtctttgccaacaagcaagatttgcctaatgccatgagccctgctgaga

tcacccagcagcttggtcttcagagcctgacccgccgtgcttggttcatccaatccacctgcgccaccactggtgatggt

ctctacgagggtctggagtggctcgccgatactctccggaaaacgaaccg

>Hypothenemus_hampei_contig_16001|c0_g1_i1 hypothetical protein QG37_02685

tcatttctataggtcacgtcccatctttgactcttctaaagagccactgaatccaacttggttgatgagtgagataccct

tataccctcaaagtgagaaacaatttagcttgtctgtgattcttagcagtccagtcatctatctttatttgccttttgtt

tatgcgagatgaacttgtttcgttttcgccttgaaaggagcgatgatctttaccctttgcttcatgcctcttcgggggct

ttagtattgggtgggtataagtggctgtctgacaagttgcttctaaacatgttttgtgtttagttgtgactatagttaac

ttgtgttgattcgtcgaccctcttttagaagag

>Hypothenemus_hampei_contig_16048|c0_g1_i1 isoleucyl-tRNA synthetase

gccgttcttcagcgagtactcgtaccaatcgctcagccccggggagagcgtgcacttccaggagtatcctgcatgcaagg

cggggacccatccattctcgacggccaaggacgtcatagctgcggtgaggcgccttagggaaatgaactcgatctcgctg

aagacacctctgaggagtgtgacggtcatcagcagtggcgagctgcacgacagcataaaggactacgaggatgtcattga

agcagagtgcaacgtgctagagctgctctacaaggaagaagagaggtcgatgttcgaggtggtggtgaagcccaatttcc

tgaacctgaggagggacaaggcctcgatgaagaagaagatggcagtgatccaaaagctctcggatgaccaggtgcatgcc

cttctggactcgtcgctggaggtcgacggagttgagattctgcaagaagacgttctggtggtgaagaagatcagatgcaa

gtctggaattgcccaggagttcggggccttcagcatagtgatcgacagcaccatggacgaggacatggttgggatgaagg

ttgcaagggagttccacagctatgtccagaagctgaggaagagcgtggggctgagcatgagcgacgacgtcctggtagat

gtcgagtgtccggccttgagggacgttgtaagcaagcacttcgacgtctcgtttggaagcctgggcgtcctgtctgggaa

gggctcctacgagtttgatggagctacgcacaccgtcaccctctacaagagggtctagccctgggattgccattaaaccg

actttattcaaaaaga

>Hypothenemus_hampei_contig_1619|c0_g1_i1 hypothetical protein Eint_101110

cttcttcacgtccagcaggtgcaagcaggagacacagctgacgatgaggtatggcgacatcgagaagtacaagctgtgcc

ttgggattgtgacgtgtgggatgggggcgctgaagattgcgccaacgccgcacctgattgtaggggtggtctccacgttc

tttgtcagcttcccgatctcgacggtggaccaagagtacctcaacatggccaggtacttcctcctcctgtcggtcaagga

aggcccaaacagccccaggagcacgatgaagtacttgaagatggggtgcaagggaagcaagagcaagatgatgagggaca

tgtctgccatcaacaagatcttccgcaaggagtattcgaaggcgtccgacgccgacaagaagctcattatcgacgtcctg

acagacttctacgagcagcatgccgacagaagcaacctgctcgacgtcgagatgctcaagaacatagcctgtagaatgcc

ctgatttattttgggctccaggcctcaca

>Hypothenemus_hampei_contig_16194|c0_g1_i1 hypothetical protein ECU10_1710

aaagaagccttgccggcggcgagggaaacatctacctgggcagcatccgaggagacaaggccatcttggtgttcccgaga

caatctgtccaggaagacgcactgcttgcaacccttgcgcttccaaaggaaaacacccagtcgaacgacgtgtactactc

ctacaaggtgtctggaacgatggacatccacttcaggatcatctaccctgcaaccaacgagcacatcagaaaatattgct

ctgctaagacatatgtccgggagacctacgaggagtacctggagttcatgacggcagcctcgcacataagctccaactgg

atggacaaccttgtcctccaggccggggagggcgtcaaggaggagatcctacaccacgacgacgaggtgattgtgattcc

ggactataagtgggatcgccggtcgactagctccctccatctgctggctgtgttcaaggacaccagcctccggacagtaa

gagacgtgggcaacccggacatcctggtcagggcgcgcaacagggtggaaggcatcctggcagccaggttc

>Hypothenemus_hampei_contig_16203|c0_g1_i1 mitochondrial ATPase inhibitor

ggtgctccccgatcgggtggccaggcgcaatctgactcgttccagcgtcgcgaagccgctcaggaaaacctgtacattta

cgagaaggaactcgagaagctccggaacttgaagaagtccatcaaagaccagcgcgaccacttggataagctcgaccagc

atattgacgagctcaccaagagccagggtggtgagcagaattaaggctcatggtccctttcattttcctctggttgaatg

gttgacgggatcataatcatgctcactttgatcataagtgcttgacgatgtcggatggacagtattctgcttaaggctct

cattccaatatcccttgacgaaagcataagttttttgagtgcatctttctatgccctttgtttcctagatgtaatctacc

tccttagctattttcctacatacctt

>Hypothenemus_hampei_contig_16209|c0_g1_i1 HAD-superfamily subfamily CECR5

ccttcaccatgccccgcatcgacgaacatgcggtggattccaccctcgtgcctccctacgcggagggtcagaagcgcgac

agatggtctcgcccttcgatcccccgggatcccaacaagattgtcgaggttccttcgacccgtgatgctatcagcgatgc

ctctgctctgatgcacaacctcagcttctcgccgtccatgaaggaccgtcgaggctcgaggaactccttcagcacttccc

tgcccattccccggtcgccgcgtctctcgcggctgtcgacggcctcccggcccagcacggtgaagcgggacatcattgct

tcccagatccaggatctccacaaggacaaggtcgccaaggtgaag

>Hypothenemus_hampei_contig_16216|c0_g1_i1 ribonucleoside diphosphate reductase

acgaagaggtatgtcaaccagggggcggagaggaggccggggtcgatagcgattttcctggagccatggcatctggagat

atttgactttcttgagctgcgcaagaacacggggccagaggacttccgggcgcgagacatcttcactgcgctgtggatca

acgacctttttatggagcgggtcaagaacaacgaggagtggtcgctgttctgtcccagcgaggctgttgggctgtctgat

gtgtggggggaagagttcaatgcgctatactgcaagtatgagaagacggttagcaggactgtggtgccggcacagaagct

gtggaaggcgattgtgg

>Hypothenemus_hampei_contig_1625|c0_g1_i1 DNA-directed RNA polymerase subunit partial

cacccagggcgacgaactggcaccgggcgtgctgaagattgttaaggtatatctggcggttaaacgccgtatccagcctg

gtgacaagatggcaggtcgtcacggtaacaagggtgtaatttctaagatcaacccgatcgaagatatgccttacgatgaa

aacggtacgccggtagacatcgtactgaacccgctgggcgtaccgtctcgtatgaacatcggtcagatcctcgaaaccca

cctgggtatggctgcgaaaggtatcggcgacaagatcaacgccatgctgaaacagcagcaagaagtcgcgaaactgcgcg

aattcatccagcgtgcgtacgatctgggcgctgacgttcgtcagaaagttgacctgagtaccttcagcgatgaagaagtt

atgcgtctggctgaaaacctgcgcaaaggtatgccaatcgcaacag

>Hypothenemus_hampei_contig_16266|c0_g1_i1 skp1 G2 allele suppressor

tttgtggagacaaaggacacggcaaccatctttctgtacaagaggtccctgcactgcgtcgacgtcagcccagcggtgga

tgggcccaggagactgagcttgtgcaaaggagtggccgtcgacctttacaaagacgtccatccggacatcagggtcgttg

agtcgccgtcgaggatagagatccaccttaggaaggtcgagccaggcaggtggggaggaatctgtggaaggccggctgca

caagccgacagcacctgcgaggggagggccgtcgaggactcggagtccgaggagggtctgtccgtgatggatctgttcag

caggatctaccagaggtctggagacgacgtgcgaaaggccatggagaagtccttctatgagtccggtggaacggtcctag

atcg

>Hypothenemus_hampei_contig_16283|c0_g1_i1 LEM3 CDC50 family

gatgaggagtttatggtttggatgcgaactgctggacttccctcatttagtaaactggcacgcagaaatgatgatactgc

catgtcatcaggaacgtatcagctggatattacagatcgatttaatgtcacggaatactctggaaccaaggcaattctaa

tctcgacccgcacagtcataggcgggaagaatcctttcatgggaatagcctacgtggttgtgggaggagtttgtgttctg

ctaggagctggtttcaccattgcatacttggtccgacctaggaaactgggtgatcatacctacttgacctgggatcaaaa

caaccagcctacgactgcaatggccacaggtcgagatgatcgaatgcgaccaagtgcgccttaaagcgagtaggcaaatg

aaacgaaacaagaaaagctgcaacggactattgcaaagcccagaatcaaagatcaaggaaaaaaaattcgaaaaaacaaa

cctcgcacaattccctttcttcctatctccctttcctactatttcgactcatcgccagtatgcaattgggtcgctgtgaa

gaaatcaggtggatcattc

>Hypothenemus_hampei_contig_16302|c0_g1_i1 hypothetical protein ALT_4995

tctctcaagcaggttccttcgttctgttcctacaaactctcgctactttctttccttgtccgtactctctacgagaccat

tctttcaccatgaagcttctctcttcccttgctctgctggtctccgccgtggcagccaacccgatggcgcgggcgaacaa

cgccagcgatactttccacctgaagacagccggggcatccgatgcgaagcacaacgatctctacgtgtatgcctatcaca

ccgccgccggattcaacgacgccgtcctgacctcggacaccgacacggccaacccggtcttcctgaacaacacgcacgcc

cagtttgccctagacacgcctttcccatggggacttaacatgcccccgcacaacaactatgccgcgtgggaaccggtcga

gatcaacaccggatacgggaccgacgggttcttcatcgatggcaccggattgcagtggtcgagcaaccttttcgggggtt

ggttggtgtgcgactggtaccacaacgaaccccagcttttctacctgtaccgcccatacgaagccaagctgccctcgtcc

tgcagcaaagttaacctggaagtcgagcacctttcctgagagaaccgactcggtgccacccgacgagggcaatggttcaa

tgacgcgcgtggcgcaagttgatgattcatacggacgatgtaccgctcgcttctcccagattcgtaccagagtcaatgaa

agtttattgataggccgtcggggagatgccgtgcgtgcatccggataatgataattttctgtaatgactgcgatggcctt

ggaaatattatgacggacggcatggttggcttcgaggtggataacgggtacttatgtattttttcttgatgtttgataat

attcgttgcatgtactgtgtatgtatctcga

>Hypothenemus_hampei_contig_16308|c0_g1_i1 1,3-beta-glucanosyltransferase gel3

agcatcgtcgcgggtgctgcattcttcgccagcagcgttctggccagtgatctggacccgatcattatcaagggttctaa

atttttctacaagagcaatgacactcaattttacatccgtggtgtcgcttatcagcaggaatactctggccccaagtctt

ccagcaacaacttcaaggacccgttggccgacgtcgacagctgcaagcgtgacattccctatatctcgaaactcaacgcc

aacaccatccgtgtttacgccatcgatcccaagtccgacgactctgagtgcatgaacctcctcagcgaacatggcatcta

tgtcattgc

>Hypothenemus_hampei_contig_16321|c0_g1_i1 Serine threonine kinase

actgcgggcactgtggagagcggatatgcctgacggaggaggagtgctggagatgcatgagctgtgggcagaggttccac

ggacattgccgggaggttgggttcaagtcgtgtggaatcgaccttgagctcaggaagggaatggccgagttccggccgcc

gtcgcctgcaaagaatcttgccagggcagtctcgatagaggactttactcttgtgcgtgtgctgggaagaggcaactttg

ggaaggttgtgctggccagacatgacagagagacagttgcaatcaaggtgctgcagaaggagaaggtcgtgaactccggc

gacgtgttctacatcgagcttgagaagaggattctcagggagatctcgctggcagcccatccgttcctcatggacatgaa

gttctgcttccaggacgcagagaacatgtttcttgtgac

>Hypothenemus_hampei_contig_16342|c0_g1_i1 glycyl-tRNA synthetase

acggagatcgggatagacgggcggctgctgaggtttaggcagcacaagaaggacgagatggcgcactatgccaaggactg

ctgggacgcagagatatacacgtcgtacggatgggtcgagtgtgtggggatagcagacaggtcgtgctactatctacggt

gccacgagagtgcgtcgaaggtcgatctgaggtgcaagaagagacttgccgagccgaaggaggttgaggagtgggttgtg

aaggtggacaagaagaagtggggtgccaagctcagggataggtttggggtgctgatggagacgatcggggggttctcgca

ggagtacattgagaagaatgccaagggagtgagtggcggcaaaggctgtctccagatcgaatttgatggagataccatcg

atgcggagtgctcgagggtcagcaagaaggtgtttgtcgagaacgtgattccggacgtgatcgagccgtcgtttggggtt

ggcaggatcctctatgcgcttctcgagcactcgttctacctgagggaggacatga

>Hypothenemus_hampei_contig_16351|c0_g1_i1 signal recognition particle Srp54

cgtggggtcgataatcctgacgaagatcgacgggagcacaaaggcagggggtgcaataagctctgtggctgcaacgaggt

gcccgatcgagtttgtggggacgggagagggcatggacgacctggaggtgtttgatgcgcggaggtttgtgtcgcggatg

cttgggatgggtgatgtcgagggcctgatggagaaggtcgggagccttgggattgacgagaaggaggttgttaggaagtt

gaagcaggggaggtttaccctgggagacttctacgaccagttccaaaagattctgagcctggggccgatttccaagctgc

ttgagatggtgcctgggttttccgggctctcgctgcccgacgagggcgcgttcaagaagctgatatatgtgtttgactcg

ttcagccgtagcgagctggactcgacgggacagatgtttgagaaggagccaagcagggtgatgcgagttgcccggggaag

cgggacgtctgtccagggcgtggttgagatccttacacagttcaagaaggtgcatgcaatgatgcagaagatcagctcga

tgcctgggctggaggggatgatgggaaacccgtcctccatgagcctggcgcagaaggcaaggatgagggagcaagcaaag

ggcatcttgccaaaggacctccttgaccagatgagctcgatgttctagctgtttggggacaggcgtttattactttgctt

tgccaataaagacat

>Hypothenemus_hampei_contig_16371|c0_g1_i1 enolase 1

aaggtttgttcagagccattgttccatctggtgcttccactggtatccacgaagctttggaattaagagatggtgacaaa

tctaaatggttaggtaagggtgttaccaaagccgtcgctaacgtcaatgaaatcattgctccaaaattcgtcgaagctaa

cttggatgtcaccgaccaagaaaaggttgatgaattcttaaacaacttggacggtactccaaacaagtctaagttgggtg

ctaacgccatcttgggtgtttctttagctgctgctaaggctggtgccgctgaaaagggtgttccattatacaagcacttg

gctgacctttctggttctaagcaagacaaatacgttttgccagttcctttccaaaacgtcttgaacggtggttctcacgc

tggtggttctttggctttccaagaattcatgattgttccaactgccgctccaactttctctgaaggtttaagaattggtt

ctgaagtttaccacaacttgaaatctttggccaagaagcaatacggtccatctgccggtaacgtcggtgacgaaggtggt

gttgctccagctatttctaccccaagagaagctttggatttgattgttgaatccattgacaaggccggttacaagggtca

agtcggtattgctttggatgttgcttcctctgaattctacaaagatggtttctacgacatggacttcaagaacccagact

ccgacaaatctaaatggttaactggtgaacaattggctaacttgtacgaagaattgatcaacgaatacccaattgtttct

attgaagatccattcgctgaagatgactgggatgcttggtcattattcttctccaaggtcgaaggtaaggtccaaattgt

cggtgatgatttgactgttaccaacccattgagaattaagaccgccatcgaaaagaagagtgccgatgctttgttattga

aggttaaccaaattggttctttgaccgaatccatcaaggctgctaacgactcttacgctgctggttggggtgtcatggtt

tcccacagatctggtgaaaccgaagataccaccattgctgacttgtctgttggtttaagatccggtcaaatcaagactgg

tgctccagcaagatccgaaagattggctaagttgaaccaaatcttgagaatcgaagaagaattaggtgacaaggccattt

acgctggtaaggacttccaca

>Hypothenemus_hampei_contig_1650|c0_g1_i1 60S ribosomal L10

ggtatgcgtggtgccttcggtaagccccagggtcttgtcgcccgtgtgaacattggccagattcttctgtccgtccgcac

ccgtgactccaaccgtgccgccgccatcgaggctctccgccgctccatgtacaagttccccggtcgccaaaagatcatcg

tctccaagaactggggtttcacccccgttcgccgtgaggagtacgtccagctccgccaggagggcaagctccgccaggac

ggtgcctacgttcagttcctccgtggccacggtctgatcgaggagaacatgaagcgcttccccgaggcctacgagaacct

ttctcaggcttagatggtta

>Hypothenemus_hampei_contig_1650|c0_g2_i1 60S ribosomal L10

ggtatgcgtggtgccttcggtaagccccagggtcttgtcgcccgtgtgaacattggccagattcttctgtccgtccgcac

ccgtgactccaaccgtgccgccgctatcgaggctctccgccgctccatgtacaagttccccggtcgccaaaagatcatcg

tctccaagaactggggtttcacccccgttcgccgtgaggagtacgtccagctccgccaggagggcaagctccgccaggac

ggtgcctacgttcagttcctccgtggccacggtctgatcgaggagaacatgaagcgcttccccgaggcctacgagaacct

ttctcaggcttagatggtta

>Hypothenemus_hampei_contig_16512|c0_g1_i1 translation initiation factor IF-2P

tacatggagaaagcggtgtttccagtcgagctgaccatcgttccaaagtgtatcttcaatgccaggagcccgctggtgat

tggggtgcagatccgggagggtgttctcaagataggaacacctctctgcgttcacaaggatggtgaggtcgttcggctcg

gggtggtgacctcgatcgagagcaacaaggaggcagtagaagaggccacaaagggacagaaggtggccatcaagatcgag

gccaaagccaacctacctccgagaatgtttggtcgacacttcgagcagagtgacattttgtactcggtgctgacgaggga

ctccattgacgtcctgaaaaagcatttctccgacgagctgacagaggaccttgcaaggctcc

>Hypothenemus_hampei_contig_16520|c0_g1_i1 Major facilitator superfamily general substrate transporter

attgcacctcttatcttcagttgcttgattgcaatggccaattacgccatctatatggcaacgatagactacatggttgc

tgcctacggcccttactctgcttctgcaacaggaggtaatggtttcgctcgtgattttctcgctggtatcgcgacaatgt

actccacgcccatgtacaccaatattggtaacaagtaccatctacaatgggcgagtacgattctgggccgcattggtttc

ctggttctcattcctatttatgtgttttattggaagggtcctgagattcgcaagaagagcaagtttgcgcaacaacttgc

tgcggatcgggagatgcatactgaaaggagacgcagtcagtttagatcgatttctgagaatgcttagatcttgggttttc

cttgta

>Hypothenemus_hampei_contig_16520|c0_g2_i1 Major facilitator superfamily general substrate transporter

attgcacctcttatcttcagttgcttgattgcaatggccaattacgccatctatatggcaacgatagactacatggttgc

tgcctacggcccttactctgcttctgcaacaggaggtaatggtttcgctcgtgattttctcgctggtatcgcgacaatgt

actccacgcccatgtacaccaatattggtaacaagtaccatctacaatgggcgagtacgattctgggccgcattggtttc

ctggttctcattcctatttatgtgttttattggaagggtcctgagattcgcaagaagagcaagtttgcgcaacaacttgc

tgcggatcgggagatgcatactgaaaggagacgaagtcagtttagatctatttctgagaatgcttagatcttgggttttc

cttgta

>Hypothenemus_hampei_contig_16521|c0_g1_i1 T-complex 1 subunit theta

gggagctcctggttgagcacgggatcgacttttcggacagggaaagcgtgtgcaaggccattcggggggttctgaaggac

agggggctggagaggattgtggcggaggcagtgatgcaaaccagaagctttgactgtgagagagtccgggtctgcaaggt

tggatgtgggtcgccggaggacagctatgttgtggaagggatggtgttcaacaggccgccagagggagaggttagggcga

tccgaggagggaggacgtcgatctacaactgcgccctggacatttcgagaacggagctgaaggggactgtgctgatgagg

acggcctccgagctgctgtcgttcagcaaggaggagaacaagaggaccaaggaggtggttgagtcgctgtgtggagacgt

gattatatgtagtggaaaggtggacaggatctacctggacttcctcaacagggcggggaagctggtgttcagaatcacaa

gcaagtacgacctgcggcggattagggagcttgtgggaggacacatttcgtcaacgctggtgccacagactcctgagtcg

atggggagcgtcgaggaggtttccacgttcatggagggcaatgccaggtatacgaagttcacctctggaagcaagagggt

gtacacgctagttcttaagaactccgtgcaggctgtgctggacgagcaggaaaggatggttcagaaggtgctaactgcgt

taagtaggaacgtctctgatgggaggattggcctggtggagggggcagga

>Hypothenemus_hampei_contig_16521|c0_g2_i1 T complex 1 theta subunit

aggatggttcagaaggtgctaactgcgttaagtaggaacgtctctgatgggaggattggcctggtggagggggcaggaag

gttcgaaaagaaactttcaaaggtctttgcagagaggtcgatggggtcgagcggggggaagagccttgtctacaagtgca

ttggaaaggcactcgggacgtttagatgttcggatgcggatgtctatgacatctacaacgtaaagacaaaggcgctgaag

tattcacttgagtttatctcgacgctgtttgaaacaagcgactatctgattggcaagcaggaagcactcaacattgcccc

caggagcaaccagcactgggacgaggatcactaggaggggccggcagatttgttttaccagct

>Hypothenemus_hampei_contig_16573|c0_g1_i1 Glycolipid anchored surface GAS1

tcttgccctactgtagacagcaactggatggctgctagcaagcttcctccttcgcccaactccgacctttgcgaatgcat

gtacgactccctcgagtgtgttcccgccgatgatatctccaacaaggacatcaagaacacctacaactacctctacggtc

tgaagcagaagggcaaggttgacgctgtctccggtgtctactccaacgctactgagggtacctacggtgcttactccatg

tgcaatgccaagcagcgtctcgcttgggccatgaaccgctacgccaagaaagccggtggttccgatgcctgtgacttcga

tggcaagggtaagagccgc

>Hypothenemus_hampei_contig_16606|c0_g1_i1 60S ribosomal L19- partial

ctcaaaagagattagcagcttctgttgttggtgttggtaagagaaaaatctggttggatcctaatgagaccaccgaactc

tccagcgctaactccagacaagccatcagaaagttatacagaaatggtaccattgtcaagaaaccagctgttgttcactc

cagatctagagctagagctttaaaagaatctaagagtttaggtagacacatgggttacggtaagagaaagggtaccaagg

acgctagaatgccatctcaagttttgtggatgagaagattaagagttttaagaagattattggctaaatacagagatgct

ggtaagattgacagacacttataccactccctttacaagagtgctaagggtaacactttcaagcataagagatcattggt

cgaacacattattcaagccaaggctgaagctttgagagagaaatctttgaaggatgaagctgaagctagaagattgaaga

acaaggctgctcgtgaaagaagacaacaaagagttgctgaaaagagagaagctttcttggccgacaactaatgc

>Hypothenemus_hampei_contig_16609|c0_g1_i1 hypothetical protein PMG11_07888

catacctgagcagcctttaatacaaacaacgttacctaatcaacatgcaattcacaactctcttcctcctcgccacagca

gggcttgccctcgcaaagccagcacctccaacatgtggcacttgtaaccccgtctcaggacaaaaccactgcgacatcac

tacctcgtgcatcaacacgggctcatccttccactgtgcctgtcgcgcgggatacaaggcatcgcagcatgacaagaaca

tcaaagagcagttcagactgaacatgccagactacaatttcctcgtcttcgttcctgagaacacggcctgcgacaccctg

tgcgataacccatttggcgcaccgaacaccatctgccaggaggtgccaaaatacgatcagtgcaaggtttaaaatcttga

ccggttgaagcggaattggttacggaattgaggaacgaacatattggttga

>Hypothenemus_hampei_contig_16622|c0_g1_i1 hypothetical protein ANI_1_24184

ctatctgatcttcataccaaacaatcgtatttcacactgactcactcgcacttatcgacattttcaatatgtctactatc

cagaagatggcttccattgaggaactcaacaagtacaactcccaatgggtggagtttcacatccgtgaccacctcgccaa

cggcgagatcaatgtccgccataccgtcatcgaaggcggcgaattccacaacgcggacacctccaaacccatgaccgaag

acgacgtcgacgagatgaccattccgtcctacggcgtcggcgagatctgcgcccgcggcagaagaggcagcgaaggccgt

ctggatcttttccacgacgaggaaaagatctgcgagctccactgggacaaccgccaggagaatggctttaatttagtcga

ggtgctggacggaagtaagtcgtacagagtggagcatgggggatggagcccagaggtaggacctctgggccatgtgtacg

ttgatatttgggagatgaagaagaagctcgaggagaagaagaagttggagcagaagatcgaggagatcgagaagaccaag

gtttgattttacggcgttttttgagacttaccagcgtgggtgggggtgttttattggctattttttcgggaattctgttt

acttgcattgtctatctacctcgcatcgacattaacgctgg

>Hypothenemus_hampei_contig_16634|c0_g1_i1 thioredoxin reductase

agtccgggcgcatacatgtgtgggatatacacccatactgcaaacctcatgcccctcatagtcaagggcagcaagggccc

cgaccttgagttctccgggtctgagaacgttgcaggggtcgccggagtccgtgcctctggagagttcatcgacctggtcg

agaggcagtcgagaaacatggggatagaggtgttggacgaggacgttctggagctctcgagaggcgatgggttcttggag

atcaagacaagctcaggaacgcacaggacaagggccttggtgatagacagtgcagccctggaggagaagtaccgctcgtc

cctagggagcagcggggtgttttacacaagcgacaagaccccatacagagaggcaatagtcattgcagggggaggatgca

aggtgtcgttcgacgtgaaggagttcctggaaaccgcgaggtaataagctcgctcgttctt

>Hypothenemus_hampei_contig_16687|c0_g1_i1 CCCH-type zinc finger-containing

ccggaaaccaagcagaagtcgacttgagatatgaagaaggagctggaggaaagggcattcgggttgaagaacaggaagca

gaaggcagccatcatgaagcagcttgaaagcctcaatctcaaggaacagcttgagaagaaggagaagatgaagaaggagg

agaggatgaacatgcctgtcaagcaggtgatccctgtgggagtcgacccaaagacggtccaatgcgtcaacttcctgaac

aagatatgtcccgacggagatgcatgcaagtttgcacatggagatgtcagaaagacggagacccctaaggaagaggcagc

agagaagggtcctagacgcatatgccagttcctgatcgacgctctgaacgccggagaatacaatggggattggaagtgtc

ctttccccaagtgtaacgacatccacaagcttgttgaaatcaaggacgatgctcaggtcgagctgagcctcgaggagtac

atcgagctatccagacaggcacttccggagaagctaacaccgctgactgaggaaacgttcaggcaatggaagctcaggaa

gcaaaaggaggagaaggagcatgccaggaaggtcaaggcgcttgcaacggggatgaagggagtggagctcttcgaggcaa

ggagagagcttttcaaggatgacgaggaggctggcgacatggactacacagaaagatgctactcagactcagatgataat

gag

>Hypothenemus_hampei_contig_16692|c0_g1_i1 1,3-beta-glucan synthase component FKS1

ctagaatgttattaggtgttatcaccatgatttacattcaaagattattattcaaattcttaaccttaacttgtttgact

agagaatttaagaatgataaggctaacactgctttctggactggtaaatggtatggttcaggtttaggttacatggcttt

aactcaaccagctcgtgaattctgtgctaaagttattgaaatgtctgagtttgctggtgatttcatcttgactcatctta

tcttgtttgttcaactcccaatcttgtgtgttccattaattgacagatggcattcaactatgttgttctggttgaaacct

tcaagattaatcagacctccaatttactctttaaaacaagctaaattaagaaagagaatggttagaaaatactgtacttt

atactttttgattttaatcttatttgttattgttattgctgctcctgccattgcatctaaatttattgctgaagatactg

gttctactctctctggtgttatgcaaggtcttttccaaccaagaaaagttgataacaacgatactggtaagaagattcct

tcatactataaaggtagttccgttacttactggagttacactccaaagagtaaaggttacactactaaaccttaagcaat

ttaatttataaactttttttctttatttacttgttcttttactttttatcttttatatatgtttggaaaaacataaattc

tctttcacatattctgtttctttctattttatatattatttcttttatttgaaaacgattctgttttatttgactttt

>Hypothenemus_hampei_contig_16776|c0_g1_i1 60S ribosomal L1

ttgatgacttgaagaaattgaacaagaacaagaaattgatcaagaaattagctaagaaatacaacgctttcgttgcttct

gaagttttgattaagcaagttccaagattattgggtcctcaattatctaaggctggtaagttcccaaccccagtttccca

caccgatgacttatactctaaggttcaagatgttaaatctaccatcaagttccaattgaagaaggtcttgtgtttggctg

ttgccgtcggtaacgttgaaatgtccgaagatgacttggttaaccaaatcttgttgtccaccaactttttggtttctttg

ttgaagaagcactggcaaaatgttggatctttggtcatca

>Hypothenemus_hampei_contig_16786|c0_g1_i1 inorganic phosphate transporter

gtggaccagtttgtaaactttgccctgtcgatagtgtcgatgaagatctcggggaagtcgtggttccagacgccgaatgc

gatctgggcactgcgggcagcgtatctggggtccaacttggtccagctgatattcttcttctacatcacgcagaagatca

agaagaccaacgacatgcgcaaggtgaagataaagaaggaggcgtctctgttccaggacagcaatggcgaggaggaggag

gagatgacatatgccagctacgaccaggcagagctcacgaagagcagccggatggcggtgatccagttcttgattgtgtg

tgtgctccacttgaagctaaaggtgatccagccgctgtttgtgcagtcgtttgcaccgataagaagcctcctgttcaatc

cgctgtacacggcatacatctggaacaagcccgtgcttaggccgttcgaggcaaacatgctgttccagaagatccctgcg

accccggaggtgaagaagagggccaaggaagactgattaaacaatccataagcatctgatctgctgtgttctgcaggctt

tgccgggcggcccgccgaggaaattaaaactttgggcttgcttttaatccccaatggaagatggtg

>Hypothenemus_hampei_contig_16793|c0_g1_i1 Ribosomal L38e

gcatcaaccgtcaccatgccttccgaagtctccgatatcaagcagttcattgagatctgccgccgcaaggacgcctcctc

tgcccgcatcaagcgcaaccgcaagtcccagcaggtcaagttcaaggttcgctgccaccgcaacctgtacacccttgtcc

tcaaggagtccgacaaggccgacaagctcaagcagtccctgcctcctgctctgaaggtcgtcgacgtcaccaagggtgac

aagaagaagtccctgtaaatacattaaaatggaatgaacagaggcccggagtgcaacgcgactggttgtcaattttcgca

tgaccaactgttagagggacacctgactggacttgatgtctctgcgagccgacacggggatggacggatcttgccgccgg

atatttcttaactgtggttgtttcggtttcccgcgtctcattcgatagggttatttttcgatcttttgagacgactcgct

tcggatgcactcgaggaatgctttgtcggagctttctagtctttctac

>Hypothenemus_hampei_contig_16840|c0_g1_i1 CCAAT box binding factor subunit C

ttggatgagagggtcaacggcttctggcaccaggcattcaaggctgcagcggacgaaaagatatttctgaaggacctgaa

cctgcctcttgcacggatcaaaaggttaatgaaggtcgaggagggggttaggatggttgcttcggaggtccctgttctgt

tttcgatgataacggagaagtttattgaggagcttacgctccgtgcatggataaacaccgaggagaacaagaggaggata

ctgcagaagtcggacctgactgcagccgtgaaaacatccgagatgttcgacttccttgtatacattgtcccgagaaacga

cctttcgaggccgtttaaccatcttgtcccaaacaaaatgcaccatggagacggctttggcccagggatgggagacgtgt

atgtcgaccagcatgcctttgacgagagggaggtgatcggggaccccgggaagaggcctgcaccaagttacaggagcttc

tatcctgaggagcacctgatgaaggccgaaccatccaagcctctgggcgacagcatgcacatggatgactatagaggggg

ccttgatatttccttcggtagggacatgcctgccgatatagctcctgagcccttcagctgagcatggtccggagggt

>Hypothenemus_hampei_contig_16844|c0_g1_i1 eukaryotic translation initiation factor 5A

tctgtccttatctcccccttcaataccatcaccatggccgacgaggtgcaagagactttcgaatccgctgacgctggtgc

gtccagcacctaccccatgcagtgttccgctctgcgcaagaacggtcacgttgtcatcaagggccgtccctgcaagatcg

tcgaaatgtccacctccaagactggcaagcacggccacgccaaggtccacatcgtcgccatcgacatcttcaccggcaag

aagctcgaggatctgtctccctccacccacaacatggacgtcccccacgtcacccgtcgggagtaccagctcatcgacat

cactgacgatgacttcctgtccctgatggacgacaa

>Hypothenemus_hampei_contig_16844|c0_g2_i1 eukaryotic translation initiation factor 5A

tctgtccttatctcccccttcaataccatcaccatggccgacgaggtgcaagagactttcgaatccgctgacgctggtgc

gtccagcacctaccccatgcagtgttccgctctgcgcaagaacggtcacgttgtcatcaagggccgtccctgcaagatcg

tcgaaatgtccacctccaagactggcaagcacggtcacgccaaggtccacatggtcgccatcgacatcttcaccggcaag

aagctcgaggatctgtctccctccacccacaacatggacgtcccccacgtcacccgtcgggagtaccagctcatcgacat

cactgacgatgacttcctgtccctgatggacgacaa

>Hypothenemus_hampei_contig_16965|c0_g1_i1 60S ribosomal L21

tctacaacggtaagaccggtgtcgtctacaacgtgaccaagtcctccgtcggtgtcctcctctacaaggttgtcggcaac

cgctacctcgagaagcgcgtcaacgtccgcatcgagcacgtcaagcactcccgctcccgtgaggacttcatcaagcgtgt

caaggagaacgcccagaagaagcgccaggccaaggagcagggcatccacctccaccttaagcgccaggccgtccagcccc

gtgaggcccacctcgttgagggcaccacccccgagaccatcactcctatcccctacgacactcacatctaaacgaatgga

atggtcgacgcggttgtttggtgttttctgggtttccgatccatgggtggattttgcattaagacgcagcgaaaaaattt

atagggttctcggatgtacgagcacatctcacag

>Hypothenemus_hampei_contig_16993|c0_g1_i1 hypothetical protein EROM_070940

tagggcttgtggcctgcaaaagcttcctggcaaccatacgcccggccgggaccgacaacttcctcaaggtggcagggaaa

agcatcgagctcactccaaacagggctgatgggcttgcaaccttccgggtgtctgtgaaggagaagaaggggttcaagct

gtttggactgaaggtcatggagcgtagatacctggttcttatatcgcctgcgtccactcccgggctgtatctggggtaca

gcaggtccaggggactccacgtgcacaagaagaagaagtactgggaatacaaggacgtgggcaatgggggatatgcactc

aagaccgccgaggatagatgtctgggggtcacggagaatggcactcttgacattgccaagtgcaataacgcaagcgagca

gatgtttctcttcgaggagaggatcgaggaccagtcgactaccacaagtgacgacgggcatctgcttgttacggcagatg

cgcaccaggctcccggcaaggatgcccgcatccccttggataacctttcttcgtcttgcgaaagcagcgactcagacagc

gacagcaacatcgcaaagcc

>Hypothenemus_hampei_contig_17024|c0_g1_i1 ankyrin repeat domain-containing 50-like

tcattcaaaagattcatataacagattagcatcagttcttttctgagctattcgtgtattacgagtgctttttggcatcc

agaaaaccaaccacctccaagttaccaaatttgtcaaccaaatgtaacactgtattgccatcctcgtttattgtatcaat

actggcaccattaatcaccagaaattcaactatatcccagtgactatataaaacagcccaatacaaaaccgtattgccct

ctatgtctgctgcatcaacattagcacctgtagtcaccaaaaactcagctaccttcaagttaccagaataggcagcccag

tgcaaaattgaaatgccatcctcgtttatagcatcaacattggcacctttattcactagaaacttaacaatgt

>Hypothenemus_hampei_contig_17034|c0_g1_i1 hypothetical protein M970_080830

cgtcatgatctcagcaatgttggactgcagcgaaaggaacgtgccggataggaactggatccctagtcccgttgccttgc

ctatgtatgctgtcagcccgacaaggctctctgtcatcatcaggaagaagactgcggaggcagctagggtgtagagacat

gagattccgggcagtattctcctcctcacagcaacatacagcagcagacccacaaggaacgatccgatgaagcatagaag

aagcccgaagtgccccggtgctatgcggaagtacgacattgcaatgagcaggttgactggtggggagagcacggttgtgt

atggtgttgt

>Hypothenemus_hampei_contig_17061|c0_g1_i1 transcription regulator

ggatcaaggaggttctgaagagcaagaacgtgcttggggactttctgaagtgcatcaactacttcaaccagaagttcatc

agcgagcaggatcttgtgtggctggtatcgccgctgcttggaaacgaggagcttgtcaaggggttcaagatgttcatcaa

ctacaaggagccaccgaaggaggtgccgaaggatgtggagaaatacaggaaggaggggtcgtacaggatcctgccggagg

agatgaggaaggggaagcaggatgcgattgccagggagacgctgaactttgcatgcatcagctgcccgacttttgagtct

gaggactcgaactacgtgtttctgaagcgcaatgtgcacgaggaggcgctgttccggattgaggacgagaggtctgaggc

ggacatgtgtttggagagggtgcagtcccttatcaattcacttgagcacgtgcttgagacgagcggcgacgtggagataa

gcatgagggacatcaggatgtcgcccgggatcatgaaggaggttctgaagagcatttacgacaagacagcaccggagata

cttgaaggaattctgatgaggccacagatcgcgattc

>Hypothenemus_hampei_contig_17064|c0_g1_i1 hypothetical protein M896_051000

aaaagtataaaagctcaggacacagatgccctaccaaactttgggctcgctatcgttaaattgcttagagtagagatgtc

gcaagaaagtatgataaacgagatttacccactagctgcgctgttcatccatgcaacgaaccaggagatcaccaaggaga

ggattgcctcagtgctccgggcacttgggatggagagccatccgaagatctgcgagttcttcgagatggatgcgctcaag

gtcaaggacctactgatgagcagcacgcaaggagccccggcacctgcgggcggagcccaggcagcgccagatgcgtcgaa

gggtgccgagaagaaggaggagcccgaagaggaggagatcgaaatcgacttcggaa

>Hypothenemus_hampei_contig_17086|c0_g1_i1 histone

tggtaaaggaaaagcttctacttctgaaaaagcttctacttctagatcttctaaggctggtttaactttcccagtcggaa

gagttcacagattattaagaaagggtaactacgctcaaagagtggttctggtgccccagtttacttaacttctgttttag

aatatttatctgctgaaatcttggaattggccggtaacgccgccagagataacaagaaatccagaattatcccaagacac

ttacaattggccatcagaaatgatgaagaattaaacaaattattgggtgatgttactattgctcaaggtggtgttttgcc

aaacattcaccaaaacttgttaccaaagaaatcaggtaa

>Hypothenemus_hampei_contig_17086|c0_g2_i1 histone

tggtaaaggaaaagcttctacttctgaaaaagcttctacttctagatcttctaaggctggtttaactttcccagtcggaa

gagttcacagattattaagaaagggtaactacgctcaaagagttggttctggtgccccagtttacttaacttctgtttta

gaatatttatctgctgaaatcttggaattggccggtaacgccgccagagataacaagaaatccagaattatcccaagaca

cttacaattggccatcagaaatgatgaagaattaaacaaattattgggtgatgttactattgctcaaggtggtgttttgc

caaacattcaccaaaacttgttaccaaagaaatcaggtaa

>Hypothenemus_hampei_contig_17134|c0_g1_i1 alpha-dioxygenase 1

tcacaaccaatttcaacgaggagacgtacaccaagaaaggtctggaacgggtgaacacaactgagagcctgaaagatgta

ttggaccgccactaccctgagatgacaaagaaatggatgaactccagcagtgcattttccgtttgggattcagctcccaa

tgctcgaaatcctatcccactttacctgcgtgttcctcagtgaaagcaaattaattatagcatcagttgtactgctttaa

gaagttttgcaggtgccattcgcgcacaaacgttgtaccgtgttcttcggtttgaaggaaactgtaaactctttcttggg

cctctgctgttctcattactgttaagtcggccatatacgtagttccaggccttgtttggattgcgttacctttagtcgct

g

>Hypothenemus_hampei_contig_17157|c0_g1_i1 ribosomal- -alanine acetyltransferase

caggatggacgagatacttgaggtggtcagtgggatttgagatggtgagaagaagatttgagccagtaaaatttaaccca

tttggatgtacaaggttgagcctatgctccccagtgatgtcttttcgcttgatcttgtgaatctagacgataagtcggaa

agcttccctctgtcctactacctctattacctgatcaaccatgcagaggattgcgtcgtggtctcgtcgccgctggaata

cgagacgtctttcacgtacaagagggacgtgcatggatacatgatagggaagctggaggagaaggacaatgccatatgcg

cacacatttc

>Hypothenemus_hampei_contig_17176|c0_g1_i1 CDP-alcohol phosphatidyltransferase

caaggcggcttgtgctgtactccacattcgtaaggattgcatcctttggggtttcgtttgtcattatgttcaacaccttg

catgcaagcagcgtgtggatacagtatctgaacatcctgatgtttgggcaggtgttctcgataaagtacatcagcgaggt

gtactcgtacatcatcaagaaggacacctttctgttcatgcctgtgtatctgatgtacctggcgctgtccggggccctgc

agctacagcacctctgggagcaccgcaaggtgcttgtatgtgcgtcgttctgcctttcctctgcatactacatcctggtt

gtttccaggatgatcgtcacgctgacagaggctctgggcatccgctttctgtcaattgcaaaggaggatcccaagg

>Hypothenemus_hampei_contig_17178|c0_g1_i1 Y215_ENCCU ame: Full=Spore wall ECU02_0150 Flags: Precursor

attcttctgatgggggtggcaatccactcgcacacgccgggcctgcggagagttccagtggaaatatacatctcagactc

tggccggaagctgttcctcgacaagggcaagagcattgaggacatcgtgggagacgttgcagagaagcttgagctgaaga

tgaacgagcatgtgtatgcagaccgagccaccatggacaggcgcaagttccactttgactggagcgtcagcggaatgacg

tccccggagatcgacctggacaagtgcgagaaggacacgagccgattccagtacgacctggtccaggcaaccagggatag

gccgggggcttcggtgattcttctgtacacctgcccgtcggaggggtacagcgaggactttatcctggcggggcagaaga

ccccccttgtcatccagagaaagtatccggagtgctcaaacgacgtggcaacgttcacagagacggagccaatgaagatc

gagtcgattattgcaaatgcactcttcgttgtggcagggtcgcccttgaacgacatggtagagtttgaggaggtggataa

cggacgggacgggttcaagagagatatacgcctaaagacaacggacttctcgctcttttcgggcggagtctgccactaac

atattaaatctacttctaatccagggcgtggct

>Hypothenemus_hampei_contig_17185|c0_g1_i1 Glycoside superfamily

tggtggtaccgactatgaggccgctaaggctggcaccaagaatgcggagaccttctggaagaagggtgtctgtggtatgc

tggactggggcgttgatctcttctacttcgaggcatttgatgagtcctggaagcctgacagcactggtgacaatggccag

cctcaaagtgagaagtactggggtttgttcaccgatgaccgcagttctaagtttgatacctcgtgcccgaaataagcggt

cgggataactatgcttgactggtagatacatattttgcctgttttctttttcgttttcttctcttccgtgtatttttgag

tttctttactgtat

>Hypothenemus_hampei_contig_17187|c0_g1_i1 hypothetical protein PGUG_00416

tagggtgataattaccccaattgaatatcgaagtttcagcttaataacaataacttattagttaactacttacttagata

cttgatattactactagttaattcgattcacaatggctcaacctcataaattattttggaaaggttctgattacgatttt

gttgtattcattgaagactccaatttggttcaaaaatacaaaggtggtgataccaccattcctttaatagacattgtttc

tatttacaaagtgtttatcaacagacaaggtggtgttgaaggtgtcttggatgaagcatccaaatctgaattagcaaatg

aatttggtagttctgacgttgacgccattattaagaagattttggtagaaggtaccgacaagaatagtgctggatctttc

cattccggtgaaaatggtaagaatgattccatgggagctggtgaagttgctcattaaatggtgccaattgctcaaaagta

tttagttttaatgaatcttacgaaatttatattacattcaccaagcatataaacc

>Hypothenemus_hampei_contig_17208|c0_g1_i1 glyceraldehyde-3-phosphate partial

ctctccaacgcctcttgcaccaccaactgcctggctcccctcgccaaggttatcaacgacaagtacggcctcgttgaggg

tctgatgaccaccatccactcctacaccgctatccagaagaccgtcgacggtccctccgctaaggactggcgtggtggcc

gcactgcggcccagaacatcatcccctcttccaccggtgccgccaaggctgtcggcaaggtcattccttccctcaacggc

aagctgaccggtatgtctatgcgtgtccccaccgccaacgtctccgttgtcgacttgacctgccgcctcgagaagggtgc

cacctacgaggagatcaaggctaccgtcaaggctgcctccgagggcgagctcaagg

>Hypothenemus_hampei_contig_17215|c0_g1_i1 glutaredoxin

gacgtggttctggtgcgaaagacgccccgagatgatattgtgtggaaggtggatgtgagcgatgagaacaggatggagta

caggctgttcgtggaggatgttgagaatgacaaggagatgattgagagcatctgcaccattgaggagaaagaaggaattg

aggaggaagtccgggtgtttggggtcagttgatgggattttctcggatagctttgttgtttcggacggcctgcaagggat

atatttgttttgaatgttgtcctggaaatggaggcccgggagttggagaaggctctgaacaagagcgagaacctgattgt

ttcgaagaaaggttgtccattctgcgacgatgctaggaagatcctgacggagtggaagatcgagtttctcgacgtcgata

agacggcgaacgaggagctctccaaggagatctcggagaagtatgggattacgacgtttccgaagatctttcttaagaaa

aagtttgttgggggagccagcgacctcaagaagtatgtgaaggggagggagtttctcgatgcctttggaggcctgaagaa

tgaagaataaaaagtct

>Hypothenemus_hampei_contig_17247|c0_g1_i1 class I chitinase

ttcaacccccagccccggcggtggtggttcaacccctggccccggcagtggtacaggcatttcctccctcatcagtcaag

ctctttttgatcaaatgctgaaataccgtaacgatccaagatgccccagcaatgggttctatagttacaacgcttttatc

actgctgcacaatccttcccgggctttgcaacaactggtaatgatgaaactcgtaaacgagaaattgctgcatttctggg

acagacttcacatgagactacaggtggatggcctagtgcaccagatggtccttatgcttggggatattgcttcataaccg

aaaatggcgcctctgggtctttctgcgattctccatcctggccgtgtcctgctggtaaacaatactacggcagaggacca

atacaattgactcacaattacaactacggtttagctggccaagcaatcggagctgacctcataaacaatccagacttggt

ggcaacggacgccgttatatccttcaagacagcaatttggttctggatgacaccacagtcaaacaagccctcatgccatg

acgtcatcactggggcatggaccccatctggtgctgacacgtcggctggccgagtcccaggatacggggtcatcaccaac

attattaacggtggactcgagtgtggcatgggtactaatgataaggtcgctgatcggattggattctacaagaggtactg

tgacctatttggaatcggctacgggaacaatttggattgctacaatcagagaccttttgcctaaagccaaacaggggact

accgaattaccgtcctcgtttaagcataaataactgttgtgtgtgacccatcaagaccaatacatgtatttgatgcgatt

tattattggatttgcactaataaaacaccgacaatgaatt

>Hypothenemus_hampei_contig_1735|c0_g1_i1 WD40 repeat-containing

tcacaaacataatagggattggggagcagggcggtctttctctatttgatgcacgggtcgggaagatgatccattcagtg

gagatgagaagcaaggtaaacgacatatcattcaacccgatgagcggacacatgtttgtgtctgcaaatgaggactgtgc

gatctatctgcacgacatgagatatctggacgagcctagcggggtgtacagaggacatgggaatgcagttgtctctgtgg

agttcgacccgctaggagccgaaatcgcctctgggagcttcgacaagacgattcgtatctttgaagtcgacgagagaaag

agtagagacacatattacaacagacgcatgcagaacgtatttggggtgaagtactcgcatgactcccagttcatcgtgtc

tggaagcgatgacgggtcgatcagggtgtggaagagccatgcctcgaagaagctggggccgcaaagcaggaaggaaaaag

acgccctcaagtactccgaggcactgatggagaagt

>Hypothenemus_hampei_contig_17474|c0_g1_i1 ribonucleoside-diphosphate reductase small subunit

cggtggatcgacgacgagagctccgactttgcaacgaggcttgtggcgtttgcgtgtgtggaggggattttcttttcggg

ggcgtttgcgtcgatcttctggctgaagaagaaggggctgatgcctgggctgacgttttcgaacgagctgatcagcaggg

acgaggggctgcactgcgactttgcatgtctgctgcacacgcatctgaggacgaagtgcaagaggacaagggaggttgtg

acggaggcggtgaggatcgagaagcggttcctgagcgagagcttgcctgtgaatctgattgggatgaactgcgacctgat

gtgcaagtacatagagtttgtggcggacaggctactggagaaccttggggaggacaaggagtaccacacggccaacccgt

ttgacttcatggagaacatctcgctgatgggcaagacgaacttctttgataagagggagtcgcagtaccagaaggcgttt

gttgggattgagaacgggaacgactcgttccggatagacgcagacttctgacgtgtgcgattaaagg

>Hypothenemus_hampei_contig_1751|c0_g1_i1 cytochrome c

aaaggttctgaaaaaaaaggtgccaacttatttaaaaccagatgtttacaatgtcacactgttgaaaaaggtggtgctca

taaagttggtccaaatttgaatggtgttttcggaagaaaatccggtcaagctgctggtttctcttataccgatgccaaca

agaaaaagggtgttgaatggtctgaacaaaccatgtctgattacttggaaaacccaaagaaatacattcctggtaccaag

atggcttttggtggtttgaaaaaaccaaaggatagaaatgatttgattacttatttggctaaagctactaaataagttgc

tcaactacgtggtcatgatatacttttagaaaagttcctttacatttttattcttttatttatttaaaattactcatata

attcttttatcttcttatattgctaatgaaggcttttttctttcattgattgaaaaggtgctcattggctgaaaaatttt

ccatttgtca

>Hypothenemus_hampei_contig_1753|c0_g1_i1 hypothetical protein EHEL_051270

gagattagcagcatccagacgagtataaccaagtatgagcatccagggttcttgctggcattttgcatcatgaagtcgat

ccgggagcttctggagtcgctctcgaccagtagcagggcgcagttcagcccgcccggagacatcgaggcgtaccatggtg

acgatgtcgtgttcgggaagattattcaggagacagtggaggagacggagcaaaacatcgacgatcttctggagaagatc

aaggcgtgggagaaggacaccaaggagacggtattctgcaagaagagcgagacgatcgagctttttagatttgcatcgca

agtgtaccacaggatgtacacgcacttcctgtccatataccatgagccgatgacgccggagacaagggactatgtatggg

aagccctgacgaatcttgccgagatagcgcgcagcagggtcgatgtgcatgggaagccggagatctggaagaagatgatg

gatgcaaggagcgaggtgtcgaaatcgaaggagatggagtatcttgtgcatggaaggttcttctccgtgcctgccgacag

acccatcgagctgatgacgatggtgatgaacgggatccgtggggagtgggttgaggagatgattccgtactacaagctat

gtgcgctgtgccaccttctggagatatccacagcgccctgaggacgcctccgccttgcccttgggaaatccatgggatat

gggccctgtccggcctgtttactggatgcaatcctgttgtagtccaataaacttagtgcaaa

>Hypothenemus_hampei_contig_17571|c0_g1_i1 translation elongation factor EF-1 alpha

agatcgaggatggcacaaaaccacgtttgaacgcttgctttatagggcatgtggactctggaaagagtacgacggttggg

atgcttgcgttccagctgggagcagttgacaagagggagatggagaagtacgagaaggaggcagcactgaacaacaagga

cacgttctatcttgcgtacttgacggacaagacggatgcggagaggaagagagggattacgatcacaacgactctgatca

acctgccgacggagaagttcgacatcaacatcctggactgccctgggcacaaggactttgtgaagaacatggtgacgggg

gcagcacaggcagacgttgcggttgtgattgttccggcatctgggtttgagtcgtgtgttgggactggagggatgctgaa

gacgcacatcatgatttctgggattcttggatgcgacaagctgattgtgtgcgtcaacaagatggacgagattgccgagg

acaagagggaggggaagttcaaggaggtgtctgacgagatgctgcggatcatcaagaggagccacaaggacaagaacccg

attgtgctgccgatctctgcgttcaagggcatcaacctgacgaagaagagcgagaagttcggatggttcaagggctggca

ggagaaggagggagcaccgacgatcatgactcttgaggaggcactggactaccagactgtgccaccccgacacaacgaca

agcctctgcgaatgccgatcaccaaggtgtgctcgatagctggagttgggaagatcctgactgggcgtgttgactacggg

acgatcacgccgaacctcaagattacgatccagccggctgggatcatagcagagaccaggtctgtggagatccacaacaa

gccgcgggcgatgattccgtgcggagagaactgtggagttgcactgaagggaggcgtgaccggagacatcaacaaggtgg

atgcagggcacgtgatctctgccaacgacgagaacaaggcggtggcataccctggagcagtgatcaggatgattgttgtt

ggcaggcccaaggggctgtctgtggggtacactccgcagatgaacttcgggaagtgccactcgccagcaaggattgccaa

gatcatgtcgaagatggttgggaaggaggtgcacaacaacccggagaacgttggcaacaacgagacccttgtgggcgtag

ttgttctgcagaagccccttgtcatcgacaagatggagaggttcccgaacctggcgaagtttgcgctgatggacagcaat

ggggtcgtagggattggtaacgtcatggagccgcttacgaaggagcagcttctcagcgaccatggcatcgacatcaacga

ggacccgaaggttgccaagaaggggacgtccaagaaggcagctgctgctgcttgagcagcccctttcgatagga

>Hypothenemus_hampei_contig_17574|c0_g1_i1 unnamed protein product

ggaccctccggaagtggcatcggcaacacagacagcatgggtgtagctcttcagcaaaacttgttcacatctttggctgc

aactcttgtgcttattggcttgatatgtccaagactctgatcagagtctgaggatgagaggttttcaagtttagtggggg

ttgaatatgtggatgatgttttgctaccagagaatgttggattggacttttggctatgtgattgtactgtggagctgttg

tgggaatcatgtaggaaactctcactaggacttcttttgttttattccccttgtttaagtgataaagttgggaatttgat

tcccttg

>Hypothenemus_hampei_contig_17588|c0_g1_i1 T complex 1 subunit alpha

ggaagaagatggcatcgatcatcctgagaggggccaactgccagctgctcgacgagatgcagaggtctgtgcacgacgcc

atctgcgtcctcaagcgcactctcgagtcagggagtgttgttcctggcggaggcaccgtcgaatgcgcattgtctctgat

gctcgagaagtttgcattcacagtcaactccaaggagcacgttgccatccaccgctatgcagagtcgcttctatcaatcc

ccaagatccttgccacaaacgccgggctcgattcaaacgagatcgtggcaagcctgctgtcttcccagagcaaggagagg

cacgacgggccaggagactcgaggttccttggcatcgacgtggcaaccggagaggtccaggacaacgccgagttcgggat

aatcgagccgagcatgagcaagatgaagtccctgagggcagcaacagaggctgcaatcagcatcctgcggatcaacgagg

tcatcgtcctgcctgccgacaaatccaagaattaaatatgctcaagcacccctcggg

>Hypothenemus_hampei_contig_17626|c0_g1_i1 DNA-directed RNA polymerase subunit beta

gatcgagagggcgctggggtacgagcagtcgaaggttgtggacaagagcacgatccgggtgtcggtacggaagatcaccg

accagagctacttctggctgcagcggatcaggaagaagatgctagggacgcgggtatgcggggcagcacaggtcagcagg

gtgatcataaacagcagcggagggctatacagccttgtgattgagggccttgagctgctggacgtcatgaacacggacgg

ggtcaagagcagcagcacaacaagcaacagcatatccgaggtggaggctgtgctcgggatagaggccgctcgggcgcaga

tcatccatgagatcgagtacacaataggcaaccatgggatcaagatcgaccctcggcacatcatgctgcttgcagacacg

atgacctacagaggcgaggtgtttgggatcacgaggtttgggatctcgaagatgtcgaggtcgacgctgatgctggcgtc

gtttgagcagacaagcgactacctgtttgaggcggctgtgcagagcaaatccgacgaggtttgcggggtcagcgagtcga

tcatccttgggattccgatc

>Hypothenemus_hampei_contig_17663|c0_g1_i1 hypothetical protein EROM_110440

tgatgcaagtctttgcagacaacttcgagattgcacggtgctcgttcgacacgctcaaggtgggagacacatgcatatcg

tcggcagggaacgacgggtcctggacgtcggtcaaggtgtcgtttccagggagcgtcaggtacgacgtgcgcgagtacga

gccgcgtaagctggaggtggtcgacctcacgcaatacttgggggacccgcaggaaatgcagttccgggtggacttcgaga

cgaagtattgggggctgatcccggacgcatggggcatgattgctgtgctggtggtctctggcattgccatgcttgccatc

cctgtgaaaggcttgcaat

>Hypothenemus_hampei_contig_17675|c0_g1_i1 SEC31-LIKE PROTEIN INVOLVED IN VESICULAR TRANSPORT FROM ER TO GOLGI

agctctcttggaaagccatcgatcccgggggtaagcgtagggggtcttggatacaggtctccaacacagcctctgtctcc

cccgggaattccccagcatggtctcaagacatcaaactctgttcctgagctgaacaggtcgacttcgtcagtctcgcctc

cggggtcacagcaaaggatgcagattcccaagcctggatacagggcagcgcctggcacgccttcgtatggcaatatatcg

ccggcagcaacaccaaggccgtatggttcgatgccgagcatgccgaaccttacgggatcatcttcagcggttttgacata

cggaatggcgtctcagggagcaggatctcatgggcatgctccccacactcctgcacaacatggcgcagcaacaaagccgt

atataccgaggcctggaatgccttcgagcccctctcagcgggctccggacttacagcccaagccaagcatccccactccg

caattgcaggccagggaaatggagccacagtctaaggctgcaacccatcctgctagcccgctgatggagaagagggatct

gaacggcgaggagatactcaacacgtttgagggggtgatcagggagctgatagaaaaggcgtctgtcaaggcgaatctga

ttgtgcggaataagctcaaggaggttacgaagaggctctcgatatacaacagcgtgacgcggagcacattcagccctgcg

atccttagcgggattgacaggataaacaacgagatcaaagacaatgggaacgccgacgcaatgaagcagaggatccggga

ggttattctcgagtgcacggagagcggagagaatagggcggacctgtggatgcccagtgtatacacgctgctacaaatag

tctatcactagtgattaaaaagatgtt

>Hypothenemus_hampei_contig_17682|c0_g1_i1 -like N-terminal domain-containing

tgtactgggtctatggagttgggccggagctaaggagtatgatatatggatgcatagaggttgtcatagtgaccatacac

ctactcagttatgcattgtatgttgacgaggttgtgagcaacatacttgtgttgtgtgcggggtttgcggttctggagac

gctgtcgacaatcaacatcgtgcattatcgacacagccttcaaagctcttttctgccaagcaagaagagtattgctgctg

gaaagatcgtcagggcagccgtatttgggttcctagtcataggtcccgttccatcgtttctgaagcccttccttgtattg

gtgcagattgtctggggagcatgtagcagaaagcatccgcttgtcgtcaatgcatgcatattacttcttcccctactgta

tataaccacactgtcttttacgctggccgggttttccgggacctttatatacattcccctattggtgtttgcattggtaa

tcatggttgctctgggcctggtgaccttgaacatcctgagcaacattccaggaagcaagtacaacaaccatgagacaaaa

acactaccatactacagcgaatcggtgtagatt

>Hypothenemus_hampei_contig_17683|c0_g1_i1 hypothetical protein EROM_010010

gctgctgacctccaagatgagtgcaaagggggctggacgctacttcgggagagactcgccggtggccgccctgatataca

tagtgataagctctttggggctcctgccgcgccctctgcgcataaatcgcaaggtcgaagtcccgatccaaatagcaatc

caggccgcgctggttgtgtctgtggtgggaacacatatatgtggctggagagaggtcgtcgtgccatgggttgtggttgg

atgcggcgccgtgctgagtgtcttctcgctgctgaaagacctgttcctgtcacggagatggagggtctggaagaagtaca

ggcgcgagtacgactggatcccgtttgtgaccgtgcttatctgcatgatcatcgtgccactggacatggctacattcaag

aactcgaagcgagagaccgagcccatccttctctccaccctcgagattccttcctggtagtagatctagaaagactgagg

gaagcgtgggatcgtgtgtcaagcaccaataaacacatacaat

>Hypothenemus_hampei_contig_1770|c0_g1_i1 hypothetical protein EROM_030970

cgaggggtcgagaaaaaagggagttcaagggcggctggacttcttcctgacaagaggataagtgtatgccttctgcctat

gtcatagagccctgtgcatcaggtttcttagatacataggcctcgtggtggacacgtgcttcttgaatgtggatgcatca

acaagtagaacgccttctttgtctatgatgcccgagtcaagaatgtcaaggaagtacaccaatatctccatgctccctgt

tttccggaactcgttgacatttgaggtgaatgccttgaactccgagacacagctgatatatcttattctcttcctcagtg

caatcacgatgaggactatgtgaaccgggttgaagaggctgtagagggtttctatcgagtactctggaaggctgtacttc

tgtatgattgctcttcgagacagggccgatatgctggggtccacctgatgctgacgcaatgcctcctcctccgaatttat

ccctgtgacttgcttgtacagggcactgtacggctcgagcggaagaaagccgatgaaaaagaccctgtggttgaatcttg

acttgacacgcttctctaaggcgtcgagagatgcacacgagtctgatacgaggaccaggcagcatggatggtccctggct

ttctccagatagtgataaaccaacgactggcgcgaggtaggcatcgagccatctatc

>Hypothenemus_hampei_contig_1772|c0_g1_i1 transaldolase

tgatgctgtcgcctgggcgaaacagcagagcaacgatcgcgcgcagcagatcgtggacgcgaccgacaaactggcagtaa

atattggtctggaaatcctgaaactggttccgggccgtatctcaactgaagttgatgcgcgtctttcctatgacaccgaa

gcgtcaattgcgaaagcaaaacgcctgatcaagctctacaacgatgctggtattagcaacgatcgtattctgatcaaacc

ggcttctacctggcagggtatccgtgctgcagaacagctggaaaaagaaggcatcaactgtaacctgaccctgctgttct

ccttcgctcaggctcgtgcttgtgcggaagcgggcgtgttcctgatctcgccgtttgttggccgtattcttgactggtac

aaagcgaataccgataagaaagagtacgctccggcagaagatccgggcgtggtttctgtatctgaaatctaccagtacta

caaagagcacggttatgaaaccgtggttatgggcgcaagcttccgtaacatcggcgaaattctggaactggcaggctgcg

accgtctgaccatcgcaccggcactgctgaaagagctggcggagagcgaaggggctatcgaacgtaaactgtcttacacc

ggcgaagtgaaagcgcgtccggcgcgtatcactgagtccgagttcctgtggcagcacaaccaggatccaatggcagtaga

taaactggcggaaggtatccgtaagtttgctattgaccaggaaaaactggaaaaaatgatcggcgatctgctgtaatcat

tct

>Hypothenemus_hampei_contig_17743|c0_g1_i1 60S ribosomal L5

gtcttcccctcgcaactaccacaagaacttccaggtgaagaggaggaggcgccgcgaggggaagacgaactacaagcaca

ggatcaacatgatccggcaggactcgaatgcagcgggcagtgtgaagcataggctggttgtgcggatcacgggcagcaag

gtgatctgccagattgtcggggcgtacatggacggggaccgggtgatggtgcaggcagactcgtccgagcttgtgcggta

cggggtgtcgtttgggctgacgaactactctgcggcgtatgcaactgggtttctgattggcaggcgggcgcttgcggcgc

tgtctctggacaaggtgtatgctccgaaggaggcgagcggggagtacagcatcaccgaggacgtcgacggggagaagaag

gcgccgcgggtgttcctggacattgggcttgcaaggtcgtcgaagggggcgcgtgtgtttggggcgatgaagggcgcgtc

cgatgccgggctgagcattccgcacagcgaggtgaagttctacgggtacaagggcaacggcaagttcgatgcgcaggagc

tgcacgacaggatcttcgggcacaacatcagcgagtacatgaagcagctgcaggagtccgatgcagagaagtacaaggtg

cagttttccgggtacatcaagaagggcattacgcccgagcagattccgcagatctacaaggatgcgctggacaggattgc

gtctgacccgtctcgggagcagaggaaggcgcgggactactctgggctgaagaagtacaagcaggcgcgattgacgtacg

aggagaggaaggagcgtgtgcgtgcaaagctgtctgc

>Hypothenemus_hampei_contig_17743|c0_g1_i2 60S ribosomal L5

gtcttcccctcgcaactaccacaagaacttccaggtgaagaggaggaggcgccgcgaggggaagacgaactacaagcaca

ggatcaacatgatccggcaggactcgaatgcagcgggcagtgtgaagcataggctggttgtgcggatcacgggcagcaag

gtgatctgccagattgtcggggcgtacatggacggggaccgggtgatggtgcaggcagactcgtccgagcttgtgcggta

cggggtgtcgtttgggctgacgaactactctgcggcgtatgcaactgggtttctgattggcaggcgggcgcttgcggcgc

tgtctctggacaaggtgtatgctccgaaggaggcgagcggggagtacagcatcaccgaggacgtcgacggggagaagaag

gcgccgcgggtgttcctggacattgggcttgcaaggtcgtcgaagggggcgcgtgtgtttggggcgatgaagggcgcgtc

cgatgccgggctgagcattccgcacagcgaggtgaagttctacgggtacaagggcaacggcaagttcgatgcgcaggagc

tgcacgacaggatcttcgggcacaacatcagcgagtacatgaagcagctgcaggagtccgatgcagagaagtacaaggtg

cagttttccgggtacatcaagaagggcattacgcccgagcagattccgcagatctacaaggatgcgctggacaggattgc

gtctgacccgtctcgggagcagaggaaggcgcgggactactctgggctgaagaagtacaagcaggcgcgattgacgtacg

aggagaggaaggcagattccgcagatctacaagg

>Hypothenemus_hampei_contig_17858|c0_g1_i1 translation elongation factor EF-1 alpha

actccaaaataccacgttaccgttattgatgctccaggtcacagagatttcatcaagaacatgatcactggtacttctca

agctgattgtgctattttgattatcgctggtggtgtcggtgaattcgaagccggtatctctaaggatggtcaaaccagag

aacacgctttgttggcttacaccttgggtgtccaacaattgattgttgctatcaacaagatggactctgttaagtgggat

caaaacagattcgaagaaatcgtcagagaaaccaccaacttcgttaagaaggtcggttacaaccctaagtctgttccatt

cgttccaatttctggatggaatggtgacaacatgttggaaccatcctctaactgtccatggtacaagggttgggaaaagg

aaaccaaggctggtaagtcctctggtaagactttgttagaagctattgacgctattgacccaccatctagaccaactgac

aagccattgagattgccattgcaagatgtctacaagattggtggtatcggaactgtgccagtcggtagagttgaaaccgg

tgtcattaagccaggtatggttgttacctttgccccagccggtgttaccactgaagtcaagtccgttgaaatgcatcacg

aacaattagaatctggtgttccaggtgacaatgtcggtttcaacgtcaagaacgtttccgttaaggaaatcagaagaggt

aacgtctgtggtgactccaagaacgatccaccaaagggttgtgactctttcgatgctcaagttattgtcttgaaccatcc

aggtcaaatctcctctggttacgctccagttttggattgtcacactgctcacattgcttgtaagtttgacactttgatca

agaagattgacagaagaaccggtaaggttttggaagaaaaccctaaattcatcaagtctggtgatgccgctatggttaag

atggtcccatctaagccaatgtgtgttgaaagtttcactgaatacccaccattaggtagattcgctgtcagagatatgag

acaaactgttgccgttggtgttatcaaggctgttgaaaagtctgacaaggccggtaaggttaccaaggctgctcaaaagg

ctgctaagaaataagttgattaatgtagaattttatattgactacttgttttatatttggttttttg

>Hypothenemus_hampei_contig_17858|c0_g1_i2 elongation factor 1-alpha

actccaaaataccacgttaccgttattgatgctccaggtcacagagatttcatcaagaacatgatcactggtacttctca

agctgattgtgctattttgattatcgctggtggtgtcggtgaattcgaagccggtatctctaaggatggtcaaaccagag

aacacgctttgttggcttacaccttgggtgtccaacaattgattgttgctatcaacaagatggactctgttaagtgggat

caaaacagattcgaagaaatcgtcagagaaaccaccaacttcgttaagaaggtcggttacaaccctaagtctgttccatt

cgttccaatttctggttggaatggtgataacatgattgaagcctccactaactgtccatggtacaaaggttgggaaaaag

aaaccaaagctggtaaatctaccggtaaaactttattagaagctattgatgctattgaaccaccaaccagaccaactgat

aaaccattaagattaccattacaagatgtttacaagattggtggtattggtactgtgccagtcggtagagttgaaaccgg

tgttatcaaaccaggtatggttgtcacttttgccccagccggtgttaccactgaagttaaatccgtcgaaatgcaccacg

aacaattagctgaaggtaacccaggtgacaatgttggtttcaacgttaaaaacgtttccgttaaagaaattagaagaggt

aacgtttgtggtgactctaaaaacgatccaccaaaaggttgtgactctttcgatgctcaagttattgtcttgaaccatcc

aggtcaaatctctgctggttactctccagttttagattgtcacactgctcacattgcttgtaaattcgacactttaattg

aaaaaattgacagaagaaccggtaaaaaattagaagaaaaccctaaattcgttaaatccggtgatgctgccattgttaaa

atggttccatctaaaccaatgtctgttgaagctttcaccgactacccaccattaggtagattcgctgtcagagatatgag

acaaaccgttgctgtcggtgttatcaaagctgttgaaaaatccgacaaagctggtaaagttaccaaagctgcccaaaaag

>Hypothenemus_hampei_contig_17858|c0_g1_i3 elongation factor 1-alpha

gggacaaaaacagattcgaagaaattatcaaggaaacctctaacttcgttaagaaggttggttacaaccctaagactgtt

ccattcgttccaatttctggttggaatggtgataacatgattgaagcctccactaactgtccatggtacaaaggttggga

aaaagaaaccaaagctggtaaatctaccggtaaaactttattagaagctattgatgctattgaaccaccaaccagaccaa

ctgataaaccattaagattaccattacaagatgtttacaagattggtggtattggtactgtgccagtcggtagagttgaa

accggtgttatcaaaccaggtatggttgtcacttttgccccagccggtgttaccactgaagttaaatccgtcgaaatgca

ccacgaacaattagctgaaggtaacccaggtgacaatgttggtttcaacgttaaaaacgtttccgttaaagaaattagaa

gaggtaacgtttgtggtgactctaaaaacgatccaccaaaaggttgtgactctttcgatgctcaagttattgtcttgaac

catccaggtcaaatctctgctggttactctccagttttagattgtcacactgctcacattgcttgtaagtttgacacttt

gatcaagaagattgacagaagaaccggtaaggttttggaagaaaaccctaaattcatcaagtctggtgatgccgctatgg

ttaagatggtcccatctaagccaatgtgtgttgaaagtttcactgaatacccaccattaggtagattcgctgtcagagat

atgagacaaactgttgccgttggtgttatcaaggctgttgaaaagtctgacaaggccggtaaggttaccaaggctgctca

aaaggctgctaagaaataagttgattaatgtagaattttatattgactacttgttttatatttggttttttg

>Hypothenemus_hampei_contig_1787|c0_g1_i1 hypothetical protein PMG11_05960

gtgataattgttctcccaatgccactaattgcaaagctccagctcccccgcaaacaaaaagtgggcatcatcctcgtctt

ttgcctgggcatattcgtcatcgcaacaagctcagcaagactttacgagctaagcataatggtttccggagaggatctca

cgagaacaaacgcggaagcagcagtctggtcttccctcgagactaacatttctattatctgcgcctgcatgccacccctt

cacccacttatctcccgcgtcttcaccctttgctttagacccgtccccctacattcctcaccagcctcaaaaatgcaatt

gaacacgacggttcttactgagtctcggaaaccatctgtatacgatcatccacctccaggcgcagatgggggtatctttt

tcaatgatttctttttcgctgggccggggacttactctgctagtattgcgaagatgaatgagaataacgaggtgaacgag

gagaatgagtcggggatcagagttgtgagggagttgaggatggtttctgattccaagcctcctagtcctcgat

>Hypothenemus_hampei_contig_1790|c0_g1_i1 replication factor A 2

ctcatcgactccgcagaggtcacgaacgtccaggcaatcggctgggtcgtgtcctcaaagacatcggcaacaggctccgt

gttcgtcctggaagatggaaccggcaggacagactgcacgttctggccaaacaacccgtacgaggaggagctgtgcaggg

ctctcgaggagggaaacctgctcaaggtcaacgggtcgctgcggacattcaacggcaagagaaacatctctgtgtcgcac

ctgtccaaggtcgaggacccaaacctcatcatctaccactttctcaactgcatccaccagcacctgttttacacaagaca

gctgcagagagaggaggtcaagccggacggcgccaggctcgagaggatccaggaggacatccttgagtgctacaggaaga

accaggacgaaaacgggctccacatcaacgtggtcatcaagatgctctcctctaagtatccggagaacgagatcagagaa

aacatcgatgccttgctgagggactgccacctgtacagcgtggatgggctagagtacaagacaacgatctgagagatc

>Hypothenemus_hampei_contig_17954|c0_g1_i1 Aldehyde C-terminal

gccagtgctgctgcgccggctcccgtatcctggtccaggaggacatccacgacaagttcgtcgctcgcttcaaggagcgt

gccgctaagaaccagctcggcaaccccttctccgccgataccttccagggtcctcaggtctcccagctccagttcgaccg

tattatggagtacattaaccatggcaagaacgagggtgctactgttgcttacggtggtgaacgccacggtgaccagggtt

acttcatccagcctacactcttcactgacgttacttccgaaatgaagattgccaaggaggagatcttcggccctgtcatc

gctgtcca

>Hypothenemus_hampei_contig_17968|c0_g1_i1 threonyl-tRNA synthetase

ggctgtcgccaaggcaggtggcagttataccaatgggaaatacggattatgttgagaaggtgaggtctgtgctctcaagg

ttccgggtggacgtgatcgacgacggaaacacgctgaacaagcgaataaggactgcggagacagggggatatgctcttgt

ttgcgtggtcgggaagaaggaggcggagaagaacgaagtgaacattcggttcaacaattcaaacagaaacatcgacctct

acgagcttcgagacatgctgaaccgaatgtctgacgagaagattgagcttgcaagcatacttcccattgaaaatatttcg

atcggcaga

>Hypothenemus_hampei_contig_17973|c0_g1_i1 4-hydroxy-3-methylbut-2-enyl diphosphate partial

atgctggtcattggagggtggaactcaagcaacacttcacatttacaagaaattgctgaagatcgtggaattccatctta

ttggattgacagcgagcagagaataggtcctggaaacagaatagcttacaagttaatgcatggggagttggttgagaaag

agaattttctacctgaaggtcccatcaccataggtgtaacatctggcgcatccacccctgataaggtcgttgaggatgtc

cttgtcaaggtatttgacatcaaacgggaagaagtcctgcagctggcatgaattagttttgcaggaaaacatggtagtat

tgcttgtttgtctgtaaaaccagctgaaactcaaagttacgagaggcaatatatgtagtataaacaggacgaccttgtag

atg

>Hypothenemus_hampei_contig_17985|c0_g1_i1 conserved hypothetical protein

cgatccggacagttcttaacaagctacaatgaggtcttctttgaattttctcttcgctcttccccttgctatgggttcca

tgatccatcctcgcgaggatggccgtggtagttacaccgtctctggtctgggatcgcgaaagcaagctgttctgaatgct

ggtggaaacaccatggacctggctattgctatgctggagaccgacacgatgaccaccgactacacctacggcgatggcaa

aaacggtgattccacgaactttggaatcttcaagcagaattggatgatgctacgcacctctgcttctgagttcgccggcc

agggagcggcagacgtgaacaatggggcgatcctcaataccgaattggacaaggacattcaagcccgccatgaaagtgag

gaccattacggtttcaatgtttgggttgctggtcataggaatggacaaagtggactggataatccgaacactgatgatat

cgccacctataagtctgccattcaatgggtccaggagcagattgaaagc

>Hypothenemus_hampei_contig_17985|c0_g2_i1 conserved hypothetical protein

cgatccggacagttcttaacaagctacaatgaggtcttctttgaattttctcttcgctcttccccttgctatgggttcca

tgatccatcctcgcgaggatggccgtggtagttacaccgtctctggtctgggatcgcgaaagcaagctgttctgaatgct

ggtggaaacaccatggacctggctattgctatgctggagaccgacacgatgaccaccgactacacctacggcgatggcaa

aaacggtgattccacgaactttggaatcttcaagcagaattggatgatgctacgcacctctgcttctgagttcgccggcc

agggagcggcagacgtgaacaatggggcgatcctcaataccgaattggacaaggacattcaagcccgccatgaaagtgag

gaccattacggtttcaatgtttgggttgctggtcataggaatggacaaagtggactggataatccgaacactgatgatat

cgccacctataagtctgccattcaatggatccaggagcagattgaaagccaggagacttattcaaccgacgatacacgct

tttgggtagatgtcacggccatctaattcgtctcctacggctcgccatagtgtgtgtggcatattgaagggtccactttg

ggaaacctttagaacccagtcgcctgtctagtt

>Hypothenemus_hampei_contig_17988|c0_g1_i1 methionyl-tRNA synthetase

gtggaaacaagcatattgagcttgatagcgttttatggactgtatcgcctaatggtcacagaggggaccttgaaaagcaa

ggtgtttattgaccagatggctgccatggtgtcgtcgtcgataagggttgatgtcgattttaggctcctggacatccagg

ttggggagatcgcttccatcgaagacgtgcctggccttgacaagctctactcagaagatgttatagcaagcgataggatg

agagtactgagcgggctaagggagcatgtagagaaggagcacatgctcggaaacaagtttcttttcgtcacaaacatgaa

gccggcaaaattcaaggggcagacctccgaggggatgattctgtgcgtgaaagacggagatgggaggatcgagccgatcc

agataccgagggaaaccaggaacgggcttcgcctggagctggaggggtgcaagacgcttctcaaggacttcagctccgga

aaggtcgacatgaagaagagtgggtatgcaaatgctcttgggtcgttccggattgtgaaccactttcttacgttcaaggg

gatgagggtcacctgtggcggggagtacgtgcgaaccaaggcggcagac

>Hypothenemus_hampei_contig_17993|c0_g1_i1 T complex 1 subunit epsilon

tcatctgagatggcatgctcgttgtcgctggagacgtttgccgagaagcacagcggggaggatcgcgaggccgtgcttgc

gtttggtagggcgcttgaggagatccctctctgccttgcgcggaacagcgggctggacccgattggatgctcgtcggagc

tgcgcaggcagcaggtggagagcaagagcccgtatctcggggttgactgccttggggctggggagcaggacatgaggaag

ctgggcgtgttcgacgcactgggcagcaagacaaggcagctgcagatggcgacacagcttgtcacaatgatccttaagat

cgacaatgttat

>Hypothenemus_hampei_contig_17993|c0_g2_i1 T complex 1 subunit epsilon

tcatctgagatggcatgctcgttgtcgctggagacgtttgccgagaagcacagcggggaggatcgcgaggccgtgcttgc

gtttggtagggcgcttgaggagatccctctctgccttgcgcggaacagcgggctggacccgattggatgctcgtcggagc

tgcgcaggcagcaggtggagagcaagagcccgtatctcggggtcgactgccttggggttggggagcaggacatgaggaag

ctgggcgtgttcgacgcactgggcagcaagacaaggcagctgcagatggcgacacagcttgtcacaatgatccttaagat

cgacaatgttat

>Hypothenemus_hampei_contig_18033|c0_g1_i1 hypothetical protein CANTEDRAFT_117236

gtgttaacgaaggtgcttctattgctaccggtggtgttagacacggtgacaagggttactttgttaagccaactattttc

actggtgttaccgaaaacatgcaaattgtcaaggacgaaattttcggtccagttgttgccgtctctaaattcagtagcgt

cgaagaagttatcgaattggctaacgactcggattacggtttagctgccggtgttcactccaaggaacttagcaacgtca

tcaaggttgctaactctcttaaggctggtaccgtctggtgtaacacctacaatgactttaacgcttctgttccattcggt

ggtgttaagcaatccggttttggtagagaaatgggtagagaatctttggactcttacactcaaaccaaggctgttaagat

cg

>Hypothenemus_hampei_contig_18034|c0_g1_i1 TUBULIN BETA CHAIN

caggacagtgcgggaaccaggtggggtgcaagttctgggagacgatcagcggggagcatgggattgaccaagaaggcagg

tatgttggggagagcgacaaccagctggagaggatcaacgtgtattacaatgaggcgtcgtcgaagaagtatgttcctcg

cgcagttctgattgaccttgagcccgggacgatggacgcagtcaggcagggggcgtttggggagcttttcaggccggaca

acttcgtgtttgggcagagcggggcagggaacaactgggcaaagggacactacacggagggggccgagctgatcgactct

gtgatggatgttgttcgaaaggaggcc

>Hypothenemus_hampei_contig_18072|c0_g1_i1 ethanolamine-phosphate cytidylytransferase

aactccgggtaccatgagaagctggttgggctgttcagagggtcgatggagaggccgcggcgtgggagggttgtgtttgt

ggacgggaacttcgacctgttccatgcaggccacgtggtgtcactgaggatagcgagggagatgggggactacctggttg

ttgggatccatgacgacgagacgaccaaggagtacacgaggagctacccggtgctggggatcaaggagaggatgcttgcg

ctgatgtcatgcaggtatgtcgacgaggttgttgtgtcgccgtatgttgttggaagggagtttgtcgagagacatggagt

cgatgttgtggggccttcgttcgactcgaaggacctgtcgcggtttgacggggtgcgggagctggtggagcacagctatg

cagagaacaggttcaactacctgtctgcagagcacattgtcaacaggatcatctcgaactaccaggagtacgagagcagg

cagaggaagaggatgcgggggaatt

>Hypothenemus_hampei_contig_18082|c0_g1_i1 40S ribosomal S9

ccgatctgagcttcagcaagagagcaaacacgcctcgggacccgtttgagaaggagcgcctgataagggagatccagctg

cttggggtgtatgggctgaagaacaagcacgagctgtgggtcatggagaagatatttgcaaaggacaaggagcgtgcgcg

tacgatcctgacttccaccaatcctgaggacatcccgattggcggcaggagtcttctgagcaagctcatgaagtacggga

tcatcggtggagtcgatcttacggacaggcacgaggtggtcatggggctcaacagggttctcgatcttactatcaaccac

tacctggaaaggcgtctccagttcagggtgtttgcagccgggcttgccaaatctgtgcaccacgccaggatcctgatcag

ggcaaggtgcatctcgatcaaggaccaggttgtcgacgtccccggattcatggtcagagcagacaaggagccactcatcg

aatacaacccatactcgtcctacggagagaagggcaagaagaaggcagctgccaaggccgatgaggaatagttcgtttaa

taaagtctctat

>Hypothenemus_hampei_contig_18134|c0_g1_i1 SWP12_ENCCU ame: Full=Spore wall 12

ccatggacaaggagacggtgaaggacatcaagaggtcgattgtgatgaagctcaacaaggtggactatgtcgaggtgcct

ctggtggacgggtactctgaggtggaggactgctaccgaaggttccgggatagcctgaaggagatctgcgacagcatcac

gtggctgatgacgtacgagcacgggggcagcaagatgaagtcgctgtactcgaagatgacgatgatcacgtcgacgtcgc

ggatcgggagcttcaagaaccatgacatctacgaggcgaatgggcttgttgggctggagctatcgcggatcgggcagtcg

tcggagctgagcaatattggcaagaagtactcgaaggcatacctggacatttcgcggtacaagagcgagatgaacagcaa

gcttgagcagcaggtcaagaagatctcagagctgcgggacgactcgagcgcaatcgacaagaagaggaagaaggtgtcga

acatgaggtatgacctggagatggagaagaggtccaaggacccgaagacgccggacgttgtgtcgaaggagaacgagatg

gagagggagttcaaggagacgtcccgaggggctctgaaggagatggagaggttcattgggacggagggtgtttccggggt

gctacagaaggttgccgagatgcatcgcgagttttctgagaagagcgcaaaggtgctaggggaggtgaaataaatgtttt

atacctggatg

>Hypothenemus_hampei_contig_18193|c0_g1_i1 26S PROTEASOME REGULATORY SUBUNIT 4

ggacgctggaggagtttgttgatgaccaccatgcaataatcacgacgggcgttgggctcgagtactatgtcaacatcatg

tcctttgtggacaaggaccttcttgagcctggatgcacagtgcttctgaactacaaggacaacagtgtcgtgggggttct

agagggcgagatggacccgatggtcaatgtgatgaagcttgagaaggcgccttccgagacgtacgcggacatcggggggc

ttgaggagcagatccaggagatcaaggagagtgtcgagctccccctgacgaatccggagctctatcaggagatggggata

aagcccccgaagggtgtgattctgtatgggctccccgggacggggaaga

>Hypothenemus_hampei_contig_18205|c0_g1_i1 hypothetical protein CANTEDRAFT_114023

gaaccggttttagagaagatcagtgaaagagtcacggaagcatcattcattagagttgatgtggatgaacaacaagaaat

cgccaaaaaatatggcatttccgccatgccaaccatcaagttcttcaatgaaggtgaagacagctcgactgttgttgggg

ccgatttgaagaagattgtagatttgatcaaggagcacactggagtggatttgatgaagaagtgaaagtaattggtcgaa

agtaattggccacaagaatctcacacaatccataaggctaactcaattacaacaaatataacttaagtatataaaacata

acgtacaagatataaatatacaaaa

>Hypothenemus_hampei_contig_18231|c0_g1_i1 multidrug resistance CDR1

tcgtttacacctccaccatgggtcaattgtgtatgtcgttctctgaacttgcagacaatgccgccaatcttgctaccatg

ttgtttaccatgtgtttgaatttctgtggtgttttggctactcccgatgctatgcctggattttggatcttcatgtacag

gtgtaatccgttcacgtacttgattcaaggtgttcttgccacaggattggccaactcggacgtggtttgtgctaaacaag

agcttctcaaattcaagccagccaatggccaaacttgcaaggagtacatggcaccctacatgaaaaaggcaggaggatac

ttgattgatgccgattc

>Hypothenemus_hampei_contig_18237|c0_g1_i1 TPR repeat

ctagggcatcacgtttaaggttgagccgttgaaacgcacgggggaggatttggctactatgagggcgagattgctttacc

aaagccgcaaacgcggcattctcgaatccgacctcctcctctcaaccttcgcggacgtctacctcgccaagatggacaaa

gcgcaactcgaggagttcgaccgcttcctagacgaaaacgactgggacatctactactgggcgacgcaggatccgccgtc

agaggaagagacctcgggcgtggcctcggacacgccgacggagacctggaagcgcaccggagccaagagcggggagtgga

cgcagacagtcggcgcgttcaag

>Hypothenemus_hampei_contig_18292|c0_g1_i1 ribosomal L21

ctcaagggatacagaagaggaaccaggtctctgttttcgcagggccacaggcagcacggcgttgcacgtgcgtcgaagta

cctcgagaggtacgagataggcgacatggtcgacatcattgtcaacccggccatgatgaaggggatgccccacaagtact

accacgggaggacaggacgtgtgtacgacgtgaagccgaggtctgtcaatgttgctctgtacaagagggttcgcgggcgg

tacgtggttaagaaggtgagtgtgcgaatcgagcatgtcagaaagagcagatgcgaggaggagagccgggaaaggatcct

caaggccagggaggaagcagcaaaggcagaggcagagggagttgtggtggctcctgtcaagagaagggttgagggcccac

gaggggcgatggagatatcgcttgagaacaaccagcccgtggaggttgggtacgagccctacatcgcgatcttctaataa

aagcgtt

>Hypothenemus_hampei_contig_18298|c0_g1_i1 similarity to HYPOTHETICAL INTEGRAL MEMBRANE PROTEIN YJJ1_yeast

agcttctgcagataacaggcataacagtcctgtcgctggttgtgtattcctccggatacaagagaagtgttctcaacgac

ttcctcttttgcatagttccccagtactgcgccataatctatccggacaggatatggaacctgtacatttgtttggcctt

gcttgtcagctactccgggtttcgaaggccggaggcagaggctaccgggagcgaaaagacaacagtcatcgactatctgc

gcttcagcatctccctgatggtggtgattgcgatatacgcagtggacttctcgatattcgaaaggaggcttgggaagtgc

cacttctttgggatctggctgatggacataggcgtagggagcttcatcctgaacgcggggatcgtggggtacaaaagcgg

gtcgaaaaga

>Hypothenemus_hampei_contig_1831|c0_g1_i1 GTP binding

tcaacgcggttttgggaaagtaccaggtcaaggagatcctctttggcctgatcaacagtgaggacgatgacataaagttc

catgcaatccaggcactgtatacgtgcatttcttcggagtggagttagtggattgggagggggccaaaggcagcgtctgg

attatcccatggttcaagcaaggagaaccagcaaaggagctgtaggttttaatggctttcttttggagaatgggagcatg

gcatgtgtcacaggacctttccttccgtggcgatctctctctcgtaccagtcaaaggctgcctccgggccgtcgtcctct

atcttatcgtactggatccttcccgagacaaggtctttgattattgtcttcaaacacttgcttgcctcccatcccttgac

ctccgacatccggctcatgtagttggcacccagagagtgcctcgagtcgttgtagaaccctctcac

>Hypothenemus_hampei_contig_18342|c0_g1_i1 similar to An01g09900

gccaggccttgaaccccaatgtcgagcgtggccatggcaaagaatcggtgaagaccaaggccagtgacacgttgatgggc

gccacttctcaggacgttcacgcgggtctgggacagcctgtatctggacagtccagcaccgagatccgtcacgatggcca

gcacggtcgcaagaaccccggcggtggcctagagggcgtcggtgcctctcaggagggcaacttcgagcgcaccatcccta

gccagcgcggaattgaccgggatcaggccaagcctaaccagcgtggcgacaagggtgctctcgctgccgaggatagagag

cctgagagcgcgagcactttggatagag

>Hypothenemus_hampei_contig_18362|c0_g1_i1 hypothetical protein PDE_01114

ctcctccacagccaatcatgagatttgctctcacttcgctggcaatcgctgctgcagtcatcaaccgctcttcctctagc

agcaagggcagcagcaccacttgctccagcaaccaggttgttgtttgcagcggcaacggcaacggcggtctgctctccct

gggcaatattgccccgggccttctcggcgacaactgttctggcggtgatgtctactgctgctcccaggacgatgttgaac

agattggtctggtcaacctgaacgtgaatgctcaatgcagtctcaatcatcttctgtaggaatcatcaagagcctactcc

gagtcttttccaatgcgcttgtatgcaaagcgttttgggcgtcgataataattagttcatatcttttactataataatga

aaacacat

>Hypothenemus_hampei_contig_18390|c0_g1_i1 hypothetical protein CPAR2_700870

ccatctcaagctgctgcttcttccccagccggtgaatctgactcttacgttgatgtcactactaccccagttgaaactgc

ttctaccggtgttgaagctactgaaaccagtcaagacactttatactctactgctccacaaggttctgaatctacttctg

ctggtaactcttcattagctccacaagcttctacttacgaagctgctgctaacggtcaaaacgtcatggttggtttcgct

ggtttagctggtatcgctgccgtcttattgtaatgaaatgatgatccttatatctatttcatttaatgacatcctattta

tgttttgtacagttttattttcttctcatag

>Hypothenemus_hampei_contig_18395|c0_g1_i1 hypothetical protein ECU01_0810

aggttgtcatgtacatttcgcttgtgctggttgtggttgtctcggagagcaggatggtcccgagggcagtctactatctt

gcagggcgctccttccttcgcgtgtcggatgcaaagggcgtgttctgcttcctgttctcgtcgtcgacggtgttcattgg

attcatgctcgaggccatgcgctcggtggcgaagatatgcaagaaggaccggatgaagtacagggtcatgaaggcacagc

gcggattcacatacatgacactgatcttgttcatcgactttctcggagcatacatattcaggtctctgaatgacagaaca

acaagcctgcttgtcctcgggctgtccagccatgtgtgctgctcgctgagcgcgtttttcggaatggacatgctgaacac

ctttttctacatcacctttgcaatgtccaacgtgtgtgtgctgctacttacatacgtgagcgggtttgaccgtgtgtttt

caagcgtaggaggacacctcgagagattcaggctctcctgggcctgaaagcaaagatgcttccgggacgcagcctgttct

gtggctcctcggctgctgccccattggttctcccgggcctggcgattgggcattgggagagggttgtttgagacttc

>Hypothenemus_hampei_contig_18401|c0_g1_i1 RNA polymerase subunit 8

gcaagctgtacacaaacgtctccagggcatacatgagctccgacacagttgcaataaccctcgactacaactccgtcctg

tgcaggctccagcaaaagcacgccgtcgaggtgcggatcttccgcggcgtcaacgaggacatcgagtgccactacctggt

cctgggcagggtctactccgtggagcacagcaacgacagggtccttgtcaaggcgtcctttggaggcctcctgctgctta

tggatgcccccgaggaaagggccaggggaatcggagacaaggacgaggtctcgctggcacttacattcatatagaaccct

caagcc

>Hypothenemus_hampei_contig_18406|c0_g1_i1 superoxide dismutase mitochondrial

ccgcaattgtggaagtgtcggagccgccagtcgtgcgcctgcgcgcaaccaatgagaccgcaacatataaaaagggttaa

tcatctacactgcagcaaataatacatcatgctttcatctaccttgagacacaatgccaagctcgccggccgtagaggtg

tcgctgcctctattggtgctgtcagaaccaaggtttccttgccagatttggactgggactttggtgctttggagccccat

atttccggtcagattaacgaactccactacacaaagcaccaccaaacctatgtcaatggttacaaccaagcagtggagca

attcgccgaggccaaggctaagggtgaggtgaaaaagacaatcgagttgcaaaagaacatcaatttccacggaggtggtt

acaccaaccactgtctcttctggaagaatttggctccagaaaagcaaggaggaggtga

>Hypothenemus_hampei_contig_18434|c0_g1_i1 TOS1 precursor

ggatgtacacaagatggtgggaactactactgttcatcaaccaataaggttacttatcaaaatgttggatattctggtag

ctataaagatgttaccaatatggatgaaagctcaggtaaatgtactcaaaaatcccaatctttctctggtaacttatctc

cattagatgaagaattatctgtccatttcagatgtcctcttaaattattaaaatttggtgtttattatccaagtgatgga

tcaaaccaaaagagagatgaacaagaagattgtaccaccactttacaacatgctcatcataaacataaaagagccaccgt

tgaagttactcaaaccgttcttgttgatggtaatggtaacactgttacttctactgctaccaatgaagcttcatctgctc

cagaagaagaatcatcagaatctccatctaccactagtgctcaaaacaacaatcaagctaaattagctgatagtagagaa

actggtgaacaaaatgtttcttatacttctggtactgaaactgcttctacttcagaatcttcttcctcctcttcttcttc

ttctgatggtggttcagttgcttctggtgattgggaaagagttgcttggtatgaaccaggttctactgataatgttacct

tcatgaattactacggtggttcaggttccggtgtttggtctggaggatttggtaactctattagttatgccaacaaagat

gccagtggtggttcttctaaagctgttgctttggaagaaactactattaaatcaaatgttgaatatatgattttctcttc

ccaatcatgtgatgacggtgatggttgtggttattacagagatggtattccagcttatcatggatttggtggtaatcaaa

aaatctttgtttttgaatttgaaatgccttctgctaatgatgatgctaccatcaatcatgatatgccagctatttggtta

ttaaacgctaaaatcccaagaaccttacaatacggtgatgcttcttgttcttgttggaaatctggttgtggtgaattaga

tgcttgg

>Hypothenemus_hampei_contig_18434|c0_g2_i1 TOS1 precursor

ggatgtacacaagatggtgggaactactactgttcatcaaccaataaggttacttatcaaaatgttggatattctggtag

ctataaagatgttaccaatatggatgaaagctcaggtaaatgtactcaaaaatcccaatctttctctggtaacttatctc

cattagatgaagaattatctgtccatttcagatgtcctcttaaattattaaaatttggtgtttattatccaagtgatgga

tcaaaccaaaagagagatgaacaagaagattgtaccaccactttacaacatgctcatcataaacataaaagagccaccgt

tgaagttactcaaaccgttcttgttgatggtaatggtaacactgttacttctactgctaccaatgaagcttcatctgctc

cagaagaagaatcatcagaatctccatctaccactagtgctcaaaacaacaatcaagctaaattagctgatagtagagaa

actggtgaacaaaatgtttcttatacttctggtactgaaactgcttctacttcagaatcttcttcctcctcttcttcttc

ttctgatggtggttcagttgcttctggtgattgggaaagagttgcttggtatgaaccaggttctactgataatgttacct

tcatgaattactacggtggttcaggttccggtgtttggtctggaggatttggtaactctattagttatgccaacaaagat

gccagtggtggttcttctaaagctgttgctttggaagaaactactattaaatcaaatgttgaatatatgattttctcttc

ccaatcatgtgatgacggtgatggttgtggttattacagagatggtattccagcttatcatggatttggtggtaatcaaa

aaaatctttgtttttgaatttgaaatgccttctgctaatgatgatgctaccatcaatcatgatatgccagctatttggtt

attaaacgctaaaatcccaagaaccttacaatacggtgatgcttcttgttcttgttggaaatctggttgtggtgaattag

atgcttgg

>Hypothenemus_hampei_contig_18437|c0_g1_i1 DEAD-box helicase

atctcggggacgctcctgaagctgatggagcttgggaagctagggcacaagggcatccggatgattgtgcttgacgaggc

ggacgtgctgctggacaaggacatgatggggacgcagatgttccggatcctcaagcttgtgtccgaggcgcagatggtct

tcttttctgcaacgtattccgagcaggtcaagcagacgattgagttctatgcgccggatgcggtgaagatgtacgaggag

cgcaacgggaagccggacgagatcaagctgttctacatcgaggcgtctggggacgcaaagaggaaggcgctgaaggcgct

gtacgagtacctgtctgtcagccagatgatcatatttgtaagtgcaaaggtgacggtcaactacctgcggaagaagctgg

aggaggacctgcactctgtgtcgtgcctgcacggcgacctggagatcgaggagagggagagggcggttgccgagttccgc

agctcgaagaccaagatcctgctgacgacggatgtgttttcgcggggcatggacattccgcaggtgaatctgattgtgaa

ctacgacctgccgatatacaagggggtggcatcgacacagacatacatccaccggatcgggcgttctgggcggtttggcc

gcacggggtttgtggttgactttgtgcgggacgaggagctggaggcgtatctgcggtttcagaacgagctgaagtttgca

tcgaagaagttcacgatcagggctcttgaggaggcggcgacggagcttgggtagcgaataaacgtcttgtgaggacttgg

ggccggcggagcgggtctctgtgcaggcatgtg

>Hypothenemus_hampei_contig_18442|c0_g1_i1 TRANSLATION ELONGATION FACTOR 1-ALPHA

ctgctgaaggccggcagtgtccagaggctgattgtcctggtcaacaagatggacgacccaagcgtcgggtggagcaagga

gaggttcctggaggtccagagcaagatcggtgcgtttgtcaagaagatgttctcggcgcctgtgttcatccccgtcagtg

ggttcaccggcgagtacatcaaggaaaagggcacgtgcccgtggtatgacggggattcgttcctgtgcgagctggacaag

ataacaatccctaggcagccggatgcgccgctggcagtcaccgtgacggagaaggtcaagtcggcaggcacgactgttct

ctatggaaaggtcgagcgcggcagagttgtacccaacatgccaataaaaatcctgccgcagaacacaaggagctttgtct

cggccgtctccgacgaggacgacgtcgagatcgagcagggcgagcccggcgacgtcgtgaagctcaagctgagggaggag

gtcgacgacgtgtctgtcggcagcaagctgcttgcaatcgacagctccgactacaagtccacacaggagttcacctgcgg

gctgaacatcctggacagcgacacggtcatcagctccggatatacctgtgtcctccacatgggcgttgttgcggtcccgt

gcaaggtcaaggagataagagacatc

>Hypothenemus_hampei_contig_18507|c0_g1_i1 V-type ATP synthase

aagacattgaccctgatgatctccaagctgggcccgggcctgatgatctctctctctgcaataggaggcggcctcgggtt

catagcagggtcggaaggaatctgtaaggcggccgaacatgctgttaataccacctactcccttgtcccgatcatcttca

tcactgcgccgacaatgtactccgtgatcctgtacttcatggtctacgacaagcagatcgagtcgctgaaggatgcgctt

ctggtcatgagtgcatgtgtggttaacggggtctcctcgggcgtggctgggtactccataggccactcggcaaaggttgc

atgtgtcacaagagctcagcagaagaagttcaacagcatcttcttcctgatcctgattttcggggaggttgtcggtcttc

ttgggcttgtgtgtgcaatggctatatcatcgagcataggtaacgagtaaccagaaata

>Hypothenemus_hampei_contig_18509|c0_g1_i1 hypothetical protein AC631_03627

atcatctgattctgattctgattcttctgactctgactcttctgacgaagaagaagaaaagaaggaagaaaagaaagaat

catcatctgattccgactcttcatccgattccgactcttcatcagactccgactccgactccgactcttcatcagactct

aaaaaacgtaaagctgattctgaatcctctgaaactgcttctcaatcatctactgaagaaccagttaacaagaaagctaa

aactggtgaaccagctactttattcgttggtagattatcatggaatattgatgatgattggttaaaacgtgaattcgaac

caatc

>Hypothenemus_hampei_contig_1851|c0_g1_i1 Fe(II)-dependent sulfonate alpha-ketoglutarate dioxygenase

caattatctcaattagatagcaagggtaaagatgaattagctctttttgttgctcaaagaggtgttgttgtgttcagaga

gcaagatttcgctgaccaaggcccacaattcgccgtggattacggtagtcactttggtagattacatatccatcctactt

ttggagctccaaagaatcaccctgaattacacattacatacagaagagccgatccaggtgaattcgatagagtgttctct

caaaatacccatgccgtgagttggcacactgacgtctcatacgaattgcaaccacctggtactactttcttcacagtctt

agaaggaccagattca

>Hypothenemus_hampei_contig_18537|c0_g1_i1 gene silencing histone chaperone

gttcatgcaggctgttgaggacggcgcacaggacggaaaggagagagtatttgagtcggagggcaaggacgaggaggacg

gtgaaagggtcgggtccgactcgtactcggagatcaacagggagctcaacaagactgttggggaggcagtatccgagggc

ggagaggacgaggacataatccactatgcaggatacaagatcgacaagaagctgattgagatgaggctggtggacgaccc

catgatcaacacgttcgagatagaatgggaggacgaggcgcctgacgacgaggcgtccagcagcagcgaggagcctgcaa

agaagcagaaggtcgagtaggtctgctttggct

>Hypothenemus_hampei_contig_18545|c0_g1_i1 Mitogen-activated kinase

ggatacttggaagattatgatgtactccgagatccttgacttccacaatattgatcaaggtactgaggctggccaggcgt

tggttactggccctgtgcaaggaaatacttcccaggctttcgcatgaatttcatgaacctatgatccataatttctacgc

gtgtttttctctcggattgtttcctctctccctccacccagggagttgtggtactcaaatcacatatctgttttaaactg

ggaaggtttgcaaagtgggcgtatccgtctcttattctattattatttttctcttttctttggcctattgaaagccaatt

gttcatagctagttcggcgtcgttaagaatcggccggtgaca

>Hypothenemus_hampei_contig_18555|c0_g1_i1 U2 snRNP spliceosome subunit

gggatgcatctcgaagatcgtcgggccgcaggaggtcctgaacatcctgatggacaacctcgagtctgaggacaggaacc

agagggcagggtcctcgctcgggatctctgttataggtgagcacaacgggctgttctctgtcctgccgaccgtgcttgct

gactatgcggttccaagtgcatctgtccagcacggggtgctcaaggcaatgtgccacttcttccagaggacgtttcagga

gtccttgagatatgtgtattcgatgctcccgatgcttgaggatgcaatgatggacgaggacccgtcctatcgatgccttg

ggatggctctggtcaggcacgttgtcctgaaccactcggcagcaactgctgacactcggcttgtcacgcatctgctcaac

ctggtgtgggcaaacgtcctcgacccgacgcct

>Hypothenemus_hampei_contig_18556|c0_g1_i1 cytochrome c oxidase subunit VIa

catgtatccaaattaattaacgaaccagctttccaaccaaataaagaagttggtgatgcttttaaaaaagcttatgaaca

taaaagagatcattcagaaccagttactttattatggaagaaaatcacttactgggtagcattaccagccatcataatca

ctgcaattccagttatcagaactgaaattaaacatgctcatcatagagaacataccaaacatttatctgacgaagaatgg

ccaactcaatatgaatatcaaaatttaagacaaaagaaatttttctggggagatggtgataaaacattattttggaattc

tgatgttaatagacacattgaagcttaaggtatttttacttta

>Hypothenemus_hampei_contig_18566|c0_g1_i1 transketolase

agggcttggagaggacgggccgacccaccagcccatcgaaacgctggcaactctacgggcaacacccaaccttattacga

tgaggccatgcgacggaacagagacaagggcctcgctgcagatcgccctggtaagacaaggcccgaaggctgtcgtcctg

tccaggcaaggcgttcctgagatcccccacacggacatgaagaaggccacgaagggggcgtatttccttgttgaggtgga

ggacccggacgtgacgctgcttgcaacggggtcggaggtccagctgtgctacgaagtggcgaagatactgccgaacgtta

agatgtgcatcgtgtcgtttttctcgtgggagttgttcgaggagcagcccgaggaatacagaagacgcatccttggggat

gcgccgagggtcagtatcgaggccctaggcacgtttggatgggcgaagtattccgatctgcaggttgggatggagacctt

tggggcaagcgggccgtacagagacgtttacaaccattttgggctcaacggtcgggagattgccaagagagtggctagtt

ttataagtaaataag

>Hypothenemus_hampei_contig_18568|c0_g1_i1 multifunctional chaperone

tttgccaggatgtcctgaatgttcttgacgactctttgatccccaaggccgagactggcgagtccaaggtcttctactac

aagatgaagggtgactaccaccgttacctggctgagttcgcctccggcgagaagcgcaaggtcgctgctactgccgccca

cgatgcctacaagaacgctaccgaagttgcccagactgatctcacccccactcaccccatccgcctcggtctcgctctga

acttctccgtattctactatgagatcctcaattcccccgaccgtgcttgccacctcgccaagcaggccttcgacgatgct

attgccgagcttgactctctctccgaggagagctaccgcgacagcaccctgatcatgcaactcctgcgcgacaacctcac

cctctggacctcatccgacagccaggatgctgagggtgccaaggaggagggcgcccccgctgctactgaggaggccgccg

ctcccgccgaggagaagcctactgaggagaccaagcccgctgaggagtcgtaagagggttttggtaggggttgcgacgag

tccgtatgccgatttttttttggtaaagggggcgttcgcatttcttccaggacgtgaattct

>Hypothenemus_hampei_contig_18569|c0_g1_i1 septin

aggtccgagaaaggtcgatagccaagggctttgagctgaacgtgcttgttgtagggagaagagggcttgggtcgacgacc

ctggtcaactcgatatttgccgcgcctctcatcgacaggaagagggaagacacgatcacagcgacgaggaacgagatcgt

cgagaacgacatatctctggaggtgtccgttgtgacctatcacgagtcggacataggccccgtgctggagtacatagatt

ccatgaacagagagtactttgacaacgagcagggcctctacaaggcgttcaaggacaacagggtgcacgtgtgtctgtat

ctcctcccttccgacgtgctgatggagcgcgaggtaagaagcatgtacgagctttcccagaagtgcaaccttgtgccgat

cataccaaaggcagacatgtacacgccggacgagctggcagacgtcaaggagagggtcag

>Hypothenemus_hampei_contig_18588|c0_g1_i1 26S PROTEASOME REGULATORY SUBUNIT 10

gtgattgagctgccgctcaagaacccggacatcttccggcgcatcggggtgaatgcgccgaagggggttctgctgtatgg

gccgcccgggacggggaagacgctgctggcgcggattgttgcggcgacgatggacgtgaacttcctgaaggtcgtgtcgt

ctgcgctgattgagaagtacattggagagtcctctcggatgatcagggagatgtttgcatatgcgcggcggaaggcgcca

tgcatcatcttcatggacgagatcgatgcgattgggggcaagcggtcgcgggagtcgagctcgtccgacagggaggtgca

gaggacgttgatggagcttctgaaccagcttgacgggttcaaggagctggacaacgtgaaggtgatcatggcgacgaaca

ggccggacatcctggaccctgcgctgctgcgtccggggcgactggacaggaagatcga

>Hypothenemus_hampei_contig_18595|c0_g1_i1 PHENYLALANYL tRNA SYNTHETASE BETA SUBUNIT

gaggaagctagcgacccgcaagaagagcgttgtgcttgtggcgaccaaagggtcgatgtttttttgatgctgcatcatac

gtgactgagctgacgtcggaaatgattcttgatgagagctacaagggcctgagcttcaagcactacaacttcgagaccaa

gggaaacatgccccgatgtgggtctgtccatccgcttatgaagatcaaggaggacttcaagaggatattcattgagctgg

gattcagcgagatggcaaccaaccaatatgtggagtcgtccttctggaacttcgatgcgctgttccagcctcaaaaccac

ccttccagagatgcacacgacacattctttctccggaaccccgaggtaagcgaaaggttccccgaggattatctacgtga

ggttggggaaacacatcgaggaggaaagtatggctctctggggcacatgagcagctgggattccaaggaggcacaaagga

acatattgaggacgcacacaacgtctgtcagtgccaggaacctgtacgagcttgggaaaaaggagtttaggcctgtgaag

ctgttctccattgataaggtcttccggaacgaaagcgttgatgcaacacatcttgccgagttccaccaggtcgaggggct

gattgttgataaaggcctgaacataagccatctgatggggattctccaggagtttttcaacaggctggggatgagcgaca

tcaggttcaagccggcattcaatccgtataccgagccttctatggaggtgtttggataccataaggggatggggaggtgg

atggaagttgggaactcggggatattccg

>Hypothenemus_hampei_contig_18616|c0_g1_i1 DNA replication factor C small subunit

aacagtgagggagacaataaaggaatttgcatcgacatgcgctaagacaatgaaactggtcatcctggacgaggctgaca

tgatgagccgagacgcccagaatgcactcaggcggataatggaggacttcagtgcaaatgcaaggttctgcctgatagcc

aaccactcgaggaagataatccctccgattctttccagatgcacgaaattcagatttggccccattgaaaacacagaggg

ccggatagaggagatatgcaagagggaggggatccggtacaccgaggaaggcattaaggccgtgctggagatatcctgtg

gggacatgcgcaaagc

>Hypothenemus_hampei_contig_18627|c0_g1_i1 hypothetical protein EROM_071050

gttcttgtgtcgcatagtaagtatgtgaaccccagcaagtacaagttcatcacgagggcaaggctggtgaagtctggtga

gagacacgtcgtgatctttggagagaatgacatctgcaaggacgggaactctgcggtgaagtgcaaggaggagaggccct

gggacatagacagaaaggagtttggatacaccatctcaaccgacggcatgtgcctcaccaagggccccaacgagtccata

gagatgaagaggtgtgtcaacacggacgaccaagtgtttgggttcaagcttgccgatctcgggggatgcgggagcgtcga

gtctctggtgggaggcagaaagccaaaaggtccaacaaccaacgtgaacatattccagccagacagcgagcacctatcgg

cgattcagataggagaaaagggagacgtgaaggagagcattattgttcggactcccgaggagtctggggagcaagacact

ccccactcctcccatgagggcggcataggtcatcatgcacatgtgctcgagaagctccaccgcccggtctacagggttga

aatgggctctgagaattgcaacacggcttccagggatccagaaagcgtccagagtcctagtgttatccatctgctaggca

atcggcacccgcactttggggccaggccccacagacatctgaggcaccactacagaccccaccaccacagcagtagagac

tccttctaaagccaccgggtccttcatggtcctgcctcga

>Hypothenemus_hampei_contig_1865|c0_g1_i1 hypothetical protein EROM_090950

gacatattagtgcgcttctacagaagatggacaacaacaccttcttcatggccttcctgtccgtcgcacttggactagcg

acgtttgtctttgtggagacaatcatatacgtcttgatggggtactttgcatttcacctatggggaatattggagtcctc

tgcatcggagactatggagggaagcccatattatgtgtaccaagcaattatgctatgcatcggcatgctgacgcttgttc

ttgtcaagcctatcacgtttttgatctatgtccttgtattctcggcctttggttctctcttcatcatagtaggagccaac

ttcggatttgggctgggccttgagctgggagaatgcgtcaacacgctccaagagagtgatttctacttgggcttgatgga

gagtagagcagctataggatatatatctctaatgacaatggggattgtttctcaactccttcttcgaaagaagcgcgtac

ggtaatcaag

>Hypothenemus_hampei_contig_18651|c0_g1_i1 mannose-1-phosphate guanylyltransferase

atacatcctcaactcctccgtgctcgacagggtgcagctgagggagtgctcgatcgagagggaggtctttcccgcaatgg

cccaggagggccagctccgggtgtttgacctcaagggcttctggatggacatcggccagcccgcagactacatccggggc

cagggcatgtacctgaggcaccacaaggacacggccggggcctcttggacgccggatgcagggacgttctcggtcgagga

caacgtcgttgtggggagaaacgtcaagatcgggaagaacgtcgcaatctcaaacagcgccatcttcgacaacgtggaga

taggagacaacgtcacgatccgggacagcatcgtggggtggaacacaaagatcaaggacaacgcaacaataagcatgtgc

tgtgtcctaggatatgcaacgaccgtcgagacgtccgccacactgacctccgtccggacacttccaccagagcccatggt

cctcgaggacgcctaagcaccg

>Hypothenemus_hampei_contig_18653|c0_g1_i1 hypothetical protein ECU09_0790

gcatccaggagagaacaatcgaaagaggatcggagagctttccggcctgtatgggtgcccagaaagaatgtcctttgagc

tgatccacgcaagcatatgtctgagaatggtttccaggcacagggatgagctgttcaaagggctgagcgagcagaagatg

aacgaaaagcaggacctgatcgagaggatgatttttgcaaccgacctgaagctaaacaaggagataatcgacatgtttga

ctccaagtacttcgagaggtcggaggcaaacccacctgggcgccgtgtgaggagcgaggttggtaaagagatatccgaga

tagagctcaaaatgattgtgaagatagccgacctgagttcgtgctacaaagactacggaatcttcaacaggaactcgact

gcattctggtcggagatgtatggcgaagacgattatgatagaacactagaagacatatctggagacatctggcttcttga

gaggatatcgattcccttggcagattcgttttcccgggtgtttgagaacacccggtttctgtatgaccaagcgattgcca

acttggagaagcacaggaaatactacgatgatataaagacccgtgaagaaggtgggtactcttccgacaaggaggtatgc

taggaggatagctaccgctcaataaacttgttctgtcttgcatgtgagctcgcgggagagcctacattaaaacacaaaaa

atttgatccccatggcgctaacgttcaaagagagactggagtttctggag

>Hypothenemus_hampei_contig_18665|c0_g1_i1 ATP-dependent 6-phosphofructokinase

gaggttttcattcccgagcggaagtacctgatcgggcacctgtccgaggctgcacagaggttgcggagccgattccgcga

gaagtccaggatgggaatagtgatattcaggaacgagaagaccttctgctcgatcccaacagagagcttctgcaagatcc

tgaaggcggacagcgagggcctatttgagacagactactcgatcctcggacacatccaacagggcctgaatccgtcaccg

atcgaccggatcaatgcagtggtgctgggaatcgaggccgtcgatctgtgcatggcgggatgcggagtcggggttgttgg

gctgttcaaggacaagacccggttcacaaagatttctgaggcgctggaggactttgacgacgccctcgacagggtgcggg

agccctgctggctcaag

>Hypothenemus_hampei_contig_18697|c0_g1_i1 hypothetical protein ECU06_1600

ggtgagcaaatggctgctttggaacaagctgtcgagatacacgtcgcggattgccaaggcgatcacggtggtgtctgcaa

ttgcatgcacggttgcagcaatgtacggagtgttttcgatgctgaatgtcatggcgaactttgcggttgtggtggtagtg

gagcagccgctgaggccaagtacaagcaatgagatcaagagtgggattcttgattcgggccgtagattgggcaaacgtgc

tttcagcggctttaaggacgcccttgggctttctcccaaaagggtggtggaagggtccataagaaaggcggttttgcgag

ggagctgagcaatggagattctttccgaacgatacacagacactgcaagttatgggataggcggggttttgtctatccag

atagactgaaat

>Hypothenemus_hampei_contig_18706|c0_g1_i1 Lipopolysaccharide ABC ATP-binding

gacgaaccgtttgccggggttgacccgatctcggttatcgacattaaacgcatcattgagcacctgcgcgacagcggcct

gggcgtgctgatcactgaccacaacgtgcgtgaaacactggcggtttgtgaacgcgcttatatcgtcagtcaggggcatt

tgatcgcccacggcacgcctacagaaatcttacaagacgaacacgttaagcgtgtataccttggggaagacttcagactc

tgatagggtagaagtttgcgacgttttagcaggagagtacgattctgaacatgaagcaaggtttgcaactcaggcttagc

caacaactggcgatgacgccacagctccaacaggcaattcgtctgttgcagttgtcgacgctggaacttcagcaggagct

acagcaggcgctggagagtaatccgctgcttgagcaaatcgacactcatgaagaaatcgacacccgcgaaacgcaagaca

gtgaaacgctggacaccgccgacgcgctcgaacaaaaaggtatgccggaagagctgccgctcgat

>Hypothenemus_hampei_contig_1872|c0_g1_i1 seventh step in lysine biosynthesis pathway

tgtcaggattcaagtggttgcaattattcagtgacaagaagatcactccaaagggcaatgccttggatactttatgtgcc

actttagaagaattgatgcaatacgaagaaggtgaaagagattgtgttatcttgcaacacaaattcggtatcgaatggga

aaatggtgaaaccgaaaccagaacttccactttagtcgatgttggtgatccaaagggttactcttccatggctaaattgg

ttggtattccatgtgccgttgctgtccaacaaattttcgatggtactttaactaaggtcggtttggtcgctccattatcc

tctgacgttagtgctccacttt

>Hypothenemus_hampei_contig_18747|c0_g1_i1 hypothetical protein CANTEDRAFT_126793

ctaatacaacgttggtttgggttacaagagtccattatactccattggtttaactgccacctccaacttatctgttttct

ctgctgcttactaccacaagatttctccattagttgaagccggtgctaaggccacttgggattccgtcaaatctaacaac

gttaacgtggaatttgctaccaaataccaattggattcttccagtttcttaaaggccaagatctctgattccggtttgac

tgctttgtcttactctcaaaaattgagacctggtgttactttaggtttaggtgcttctttcgatgctcttaaattatctg

aaccagtccaca

>Hypothenemus_hampei_contig_18761|c0_g1_i1 elongation factor 1 gamma domain-containing

agtacctcttcggtgctcagtccgtcttcggtaccaactacgactgcgccatccgtggtgtcttcttggtccgtggccag

gagtccgcccccgctttcgaggttgcccccgactgggagagctacaacttcgagaagctcgacccctccaaggaggagga

ccgcaagtacgttgaggacatgtgggcctgggacacctccatcaccatcaacgacaaggaatacccctgggttgatggcc

acgtcttcaaatagacggttccttccgaaccccaaggcaattttcttgtcaccctgacctgttttcaagtcgggtgagct

tctagatgaaacccggctccatgaaaaccacgtctccctacctctcccttaaggtgaagagtgctcccggggcagctggg

ttatgaaagcctcgtatcccgcctgtccgctct

>Hypothenemus_hampei_contig_1877|c0_g1_i1 nlpB lipofamily partial

tcgtggtcgttatcaaatctctgttaagccgcagggttatcagcaggcggttacggttaaactgctgaacctggaacagg

cgggcaaaccggttgcagacgcggcttccatgcagcgttacagcacggagatgatgaacgttatttccgccggtctggat

aaatctgccactgacgccgcgaacgctgcgcaaaatcgtgcctccaccactatggacgtacaaagtgcagctgatgacac

cggtttaccaatgctggtcgtacgcgggccgttcaatgtggtttggcaacgtctgccagcggcgctggaaaaagtgggca

tgaaagtgaccgacagcacccgttcgcagggcaacatggccgtaacttataagccgctgtctgacagcgactggcaggaa

ctgggcgcgagcgatccaggcctggcatccggtgactataaactgcaggttggcgatttagataaccgcagcagcctaca

gttcatcgatccgaaaggtcatactctgactcagagtcagaacgacgcgctggtagctgtcttccaggctgcgtttagca

agtaaaaatacag

>Hypothenemus_hampei_contig_18786|c0_g1_i1 GAMMA GLUTAMYL TRANSPEPTIDASE

cataagcgggatagccgagaagcatgcaaacctgccggagggcggcaagaggccgttttcctcgatctcgcccatcctcc

tgctcaaggacggcgaggtcattgccctaggcgcagccggcggcatcaggatcccgacgtcgattgtgtccacactgttc

cacctctctgcaggagacagccttgaagaggcaatcatggagacaaggatccacaaccagctgttcccaaacgtgacgtt

tgtagagtatgggatctcaagggcccttgagaagtaccttctgggcgccgggcatcgaatagagaagtcgttgcagaata

cgatcttcacctctgtgcagggcattctttccaaggaggccaatggaagcagggccatccacgccgtgtcggatccccgc

>Hypothenemus_hampei_contig_18787|c0_g1_i1 hypothetical protein EROM_060500

aacaaggacgagatcgactggctccagatccaggagatctaccatgtcaacgacctgttctactttgacgtcctggttgg

ggagctcaatgggccccatgtggtcaagcggggacagcatctggagataggaaaggagctcgtgctgcttctcaaggact

acgagttcaagctcctgtcgaagctgtccatcctcgaccggctttacaggagtctgtatgcaagcgggatgctgagcgag

gatcttgggcgcaggatcgggcgggtgccgcaacaaggtgtggccagggaggtgtccaagaagacaaaggaggctggagt

cgacagaagcgaggggagcggcaggaagaaaaggcccagcagcatagcgattgtcgtcaagggcagcca

>Hypothenemus_hampei_contig_18806|c0_g1_i1 60S ribosomal L24

ccaatcctccaaatctgcttctttgttccaacaaagaaagaacccaagaaagattgcttggaccgtcttatacagaagac

aacacaagaagggtatttctgaagaaacctccaagagaagaaccagaaagaccgtcaagaaccaaagagctattgttggt

gcttcccttgatttgattaaagaaagaagatcccaaaagccagctgaaagaaaggctgccagagaagacagaattgctaa

ggataaagaagctaagaagaacgctaaggctgctagaaaggctgaaaaggctaagtccgctgctaactctgctcaatcta

gagtttccaaacaacaacaaaagggtgctttccaaaaggttcacgctacttcccgttaagaca

>Hypothenemus_hampei_contig_18808|c0_g1_i1 unnamed protein product

caaccagaaaaggtagccgagctgtcggaaccaacatctccagagttgaaagaaacaattcattctgttgtatacggtct

cttggccaccctctcccctaaaatgcattcgagggcatctaatttcatggaggacacttccattgggaccagaaatactg

cagatggagatgattgcattgaagtggagaacacttcacttcaatttcgaccgcatgtcttgttaactagggattatctg

gctcggcttttgttttggtgcatgctacttgggcactatcttagagggctcgagtatcggttggagctgacggaacttct

ctctttgccgtgtgttgtaaagaatgaaacttggaatgggta

>Hypothenemus_hampei_contig_18817|c0_g1_i1 phosphatidyl synthase

atctggagcgtgtgctctacggtaagcccgagcgggccacctacatctacgccgacgaggtcatgaaggcgtggatggag

cagattcaccagcagagcaagctgcccaagaacatctacatggtcggcgacaaccccgcgtccgacatcatcggtggaaa

cctgtacggctggaacacctgcctggtccgaacgggtgtcttccagggcggcgagaacgatgagcaaaatcctgccaact

tcggtgtcttcccgaacgttatggaggctgttaagtcggctatccgtaaggagctgggcgacgacttcggcttcaagtgg

gagcccaagatcagcaacc

>Hypothenemus_hampei_contig_18819|c0_g1_i1 thymidylate kinase

ggtagttctttgcgagcgctactctatgagcggaatagcatattctgccgccaagggcctggacctggagtggtgtgcca

tgtccgaaagccggcttcctcgccccgaccttaccgtgtttatagacgtccctatggacgaaatttgccggcgaaagggg

ttcggagaggaggtccacgacaacctcttgttcctcaagaaggtacactcctcgtatatgaggcttcttgagaaagagga

aaacgttctagtcgtcgacggtaccctctctgtggacgagattgttgaggtcgtcagccaaagaatacttggatagctat

ttgctttactggcattgacatgtgagccgccgggaaaatggatattacttgactgtcgaacccacttctagaggctcact

cgaccttttccaggtttgaggacacatcgtcgtcgctcccatttggctcctcggtgcggggggcctcgtcgtcctccttt

ccgtcatccttcccaaagatgttctttactccgcgggtctgtgagatgtacttcaggagatagtactgg

>Hypothenemus_hampei_contig_18827|c0_g1_i1 phosphoinositide polyphosphatase

caagagtgcttttggtgacatacacaagaaactcgtcgtcggcccccctcctctcggccctcaggatcccaaggaccgcc

tggaactcggcctgccttaggtccggccttctgcaggctgctgcggcgcaggaaagagtggctacgccggactctgtctc

ccacgagatcctcctggaggatgccgagtacaccagggttgccatccggttgctatggggttggcttccggcctgtttgt

ttgatgccggcagggcgcaggcacagagacggcgtgcggggtggctcttgaggcagaggtgaaaccaagtgtcaagcagg

ccaaata

>Hypothenemus_hampei_contig_18830|c0_g1_i1 alpha-1,2 mannosyl-transferase

aagctggaggcagaggagtgggaggttccgaagtggatagacatggagagggccaaagaggagatgaagaagatggagga

taggaagatcatctacggagggtctttgtcgtacaggaagatgtgccggttcttctctgggttcttctacaagaacaagc

tagtccagaagtacgactattactggagaatcgagccggacgtgaagttcctatgccagatagagtacgacccgttcaca

tatctaagggagaacaacaagcagtatgggtttgtgatatcgctggtcgagtttatggagacgattcccagcctctttgg

agaggttgtgaagtttgtgacgcagaacctgaatctggtcaggaaggcggacgggaacaggttcatactggggaacaaag

ggtcgtacaacggatgccacttctggtcgaatttcgagatagcaagctttgggttcttcaggagcgagacgtaccagaaa

tact

>Hypothenemus_hampei_contig_18837|c0_g1_i1 hypothetical protein EROM_020190

aggcactctgcagcggcagcattctgataagcgaggtcaatagcaagacagacaccttcaagttcaacaacatgatcctg

aacatcgagaagtcgggtgcatttgaaaagagcataggccacaagctcggtagaccgggatacatggtgaggggggaggg

aagtaaattccagatagagttcttcacgagcatggacaacctccgggtaagcaggataagccccaataagataaaggttg

cctacagttccaaggacaagcttgcacagaacacctatctagagatatcgattaagaaccacatgattaagagctttaag

atattcgacttctacatgctgggaaaggcacgcctcagctcgaaactagacataacaacttgagcctcagtaaacctgta

agcaacattttat

>Hypothenemus_hampei_contig_18849|c0_g1_i1 prenylated rab acceptor 1

caacgccatctgggaaaggatctgattggtgccatggagcaagtctcctggacagcgcccgattggtgggcaggctgaga

ctcaccaatcggctttgaaacagcgcggaaaccaaagccatctttggtttaaacaaagaggccccggcaaaacatgcaag

gaggaatccaggaggagaaagctttccagaagaacatcaaggaggtcttctcaaacaacgcaaccacaagggacttcttc

agcatacgcagggtctctgtcccaaaggacttcggcgacgcaaagagaagggtccttctcaacctagatagattcaagtt

ccattatcttgcaatggcaagtgcattcacgctgatctatgtcctgtaccgcctggagctcgtcatactcatcggaatcg

tcgcaactgctgcgtatgtgtacaagacaaagccaaccctgtggaacgtcgagatggagccgaggtctgtgtgcatagcg

ggggccgtcggcgtgcttgtcttcttcatcttcttcaaggaggcaatcgtggggcttcttgcaatatccgccctctgcgg

catgataaccctgacacatgctgcatcgctcgagggcgatcttgagagggacg

>Hypothenemus_hampei_contig_18850|c0_g1_i1 Ca2+-dependent lipid-binding

attaagagcggaagtaacatccagtctgcaacccggaatcccgtcgacagctactgtaaaattttagtcaatgagacgaa

ggtgtactcgaccaagcccgtgcgcaagagtagtgcccccgtcttcaacgagtcgtttatcatggagatagacaagacaa

aggatgtgtttggggtccagatctgcgactacaacgtctcggagagaaacgcgcttctatactacacggagttttccttg

cacaacctgtccgaagggttcagcgagatggagttcgagctctcggatggaaagacctttcagccaacggggtcaacgat

ccggattggattcaacttctgtgccgaccacaagagcctgaggatcaggaagaaagggattctgagcgacttctttgggt

tttaagcctaggctttgaccgg

>Hypothenemus_hampei_contig_18853|c0_g1_i1 40S ribosomal S17

tctctaaaatgggtcgcgttagaactaaaaccgttaaaagagcttcaaaagttttaattgaacgtttctatccaaaatta

accttggatttcgaaaccaacaaaagattaactgatgaaattgctgaaatccaatctaaaagattaagaaacaagattgc

tggttacaccacccacttgatgaaacgtattcaaaaaggtccagtcagaggtatttctttcaaattacaagaagaagaac

gtgaaagaaaagatcaatatgttccagaagtttctgctttggacttatctcacaccaacggtcaattagaagttgattct

gaaactgctgatttagttaaaactttaggtttcaaaattccaattcaaaccgttgccatctcttctcaaa

>Hypothenemus_hampei_contig_1886|c0_g1_i1 Rho-associated coiled-coil domain-containing kinase

caaggagtacgagcaaaacctcaaccaggaggtcctcttccgcaaaagtgttgaggaggagctgaactttctgaagaagg

agaggctgagggtgcacaaggtggctgtaaagcagtctttttcgtgctggctcaccaacggagagtcaacaaccataagg

atggaggaggatcgcctatggattggcgacgagacacaggatatctgcaacacatacatcggcgagctgcggcctaatga

gctccaccatcttccgcagaagaagatatcgctgactcttaggattgttttcatgagcgaggaggtcaagagcgtcagct

cctcgggaagaaga

>Hypothenemus_hampei_contig_18863|c0_g1_i1 diphthine synthase

gccctgataaggatgtccgagtgcgtcgttgcaaagagtggcgcaccaacaacaagcagtgagacgtcctcagcagcagc

ctcgtcgactatcctgtctgtctgctccatcatctctctgtctgcaagacacaccttcttgcccaccagctcctcaaatg

catccaggggctctccatggattgatgtgtagctctcgagatacaccttgctgctgtctctggcagcctcgagcccgcgg

agagtgatgtccttgtaggagtgcagcccgaatccgatgagatagagcatgggagcttttgaggcggggaatttatgacc

catagatggacgggcctgtgtacaaggttcggaaggaggactacatgaggtatgtggatgggacgccctacatgatctac

acgtcgctggcgcttgtgatggtgggcttctctatattctcagcgccgtctctgtttgtgtacagcctcgtgcctctgtt

catggcggcagcaatcgggtccccgaagcaggagcgggtgctggtggcgattctggggtgcatcatggcgtttgtggtgc

cgatgtacttcaagaagtagcgaggtgaagaaataaaag

>Hypothenemus_hampei_contig_19023|c0_g1_i1 hypothetical protein M970_110220

ggattctgtgcgagtggaaggagggaggagaagatgatgtccctgtggatctcctggccttgctggtggatggcaaatcg

actgtcgattacgagtggatggacaagattttgtacatgtacaagaaaacatcgatgaagaaggacgatcggataaaagg

cctcaaggcggctgttgaggcgaggttccgagcggaggcaaacagagggcatggggtctcgggagcctatttgctgaacg

accttgatgctgtttccaagagtgttgctgctcttattcgacggggtcggaagaagctcgagagacacggggggatggag

agaacagacgttggcagggcagtaaaga

>Hypothenemus_hampei_contig_19034|c0_g1_i1 NADH-ubiquinone oxidoreductase subunit GRIM-19

cttccagggtactcgcgagcaacgtgagcttgcccgtgagaagatgtgggcccgtatgtacttgactcctctcctccagg

ccgaggaggaccgtgaccaggtccgtcgtcacttcgccaactctgcccgtgagaaggagctccttggcagcgagcagaag

atctaccactccgaccgcttcgtccctcccactatcacctatactcccccggcgactccctccggaaaggcttggtacca

gttctggtaaactccttcgggaaccgaccttgcatcctcatgattcagcaatcttggtgtctgggctatgctgctgttta

cctgcagtgggctggagttgaacgtggtcgaaggaagtattttggggcagaaacagaagagatcgtgtggtgaaatgtgc

ttgataagaccgagcaaattactgtaga

>Hypothenemus_hampei_contig_19077|c0_g1_i1 nucleotide triphosphosphatase of the ham1 family

ggtggagctggtatcttttgagatggatgagattcaagggacaaaggaggagataatcatggacaagctcaggagggtga

gccatctagcctcggaaggtgtgctggtctttgtggacgacacgagcatccatctggacgggctgggagggttccctggg

cagtatgccaaggactttctggccatggggtttccacggatacttgagattgcctcgaaggttggtgggggatgtctgta

ctccacggcgatcggagttatccatatgcacaaaggggagcttgttgcaaggtccttcacgggggaggtgagagggactg

ttggtaggaagaagggggttgatccgaaggta

>Hypothenemus_hampei_contig_19127|c0_g1_i1 60S ribosomal L3

ggtgcctggcaccctaaccacgtccagtggactgttgctcgtgccggtcagatgggttaccaccaccgtacctcttgcaa

ccacaaggtcttccgtatcggcaagggctccgatgagggcaacgccgccaccgacttcgacatctccaagaagcagatta

ctcctctgggtggcttcgtccactacggtgaggtcaagaacgatttcctcctgctcaagggctccgttcccggtgtcaag

aagcgcgtcatgaccctgcgcaagactctgtacccccagaccaaccgccgcgctaccgagaaggtcgacctcaagtggat

cgatacctcctccaagttcggtcacgg

>Hypothenemus_hampei_contig_19127|c0_g2_i1 60S ribosomal

ggtcaacggtggctccgttgccgacaaggttgatttctcccgcaacctgttcgagaagaccatcgatatcgacagcatct

tcgagaaggacgagatgatcgatgtcatcgccgttaccaagggtcacggtttctccggtgtcaccagccgttggggtacc

accaagctgccccgtaagacccacaagggtctgcgtaaggtcgcctgtattggtgcctggcaccctaaccacgtccagtg

gactgttgcccgtgccggtcaggacggataccaccaccgtacctcctgcaaccacaaggtcttccgcattggtaaggcca

ccgatgagggctccgcctccaccgagttcgacatctccaagaagcagatcactcctatgggtggtttcgtccactacggt

>Hypothenemus_hampei_contig_19160|c0_g1_i1 ATP synthase alpha mitochondrial precursor

gtgtctcgtgtcggttccgccgcccaggtcaaggccatgaagcaggtcgctggttccctcaagctcttcttggcccagta

ccgtgaagtcgctgcctttgcccagttcggttccgatctggatgcctccaccaagcagacccttgcccgtggtgagcgtc

tgaccgagctgctcaagcagaagcagtactcccccatggccgtctctgacatggttcccctgatcttcgctggtgtcaac

ggtttccttgaccagatccccgtcgccaagatcctccagtgggagtctgacatcctcgctcacctgaagagctcccaccc

cgagatccagcagaccatcgagaaggagggccaggtctccaaggagcttgaggctaagctgaaggaggtcatcggtgcct

>Hypothenemus_hampei_contig_19160|c0_g1_i2 ATP synthase alpha mitochondrial precursor

ctggatgcctccaccaagcagactctcagccgtggtgagcgtctcactgagctcctcaagcagaagcagtactcccccat

ggccgtttccgacatggttcccctcatcttcgccggtgtcaacggtttccttgaccagatccctgtcgccaagatccttc

agtgggaggccgacctcctcgctcacctgaagagctctcaccccgagatccagcagaccattgagaaggagggccaggtc

agcaaggagctcgagaaccagctccgtgaggtcatcggtgccttcaacaagtctttcaacgcatagacaagttagcttgt

acaatttgagaccactgttttcttttctttttcatcaga

>Hypothenemus_hampei_contig_19160|c0_g1_i3 ATP synthase alpha mitochondrial precursor

gtgtctcgtgtcggttccgccgcccaggtcaaggccatgaagcaggtcgctggttccctcaagctcttcttggcccagta

ccgtgaagtcgctgcctttgcccagttcggttccgatctggatgcctccaccaagcagacccttgcccgtggtgagcgtc

tgactgagctgcttaagcagaagcagtactcccccatggccgtttccgacatggttcccctcatcttcgccggtgtcaac

ggtttccttgaccagatccctgtcgccaagatccttcagtgggaggccgacctcctcgctcacctgaagagctctcaccc

cgagatccagcagaccattgagaaggagggccaggtcagcaaggagctcgagaaccagctccgtgaggtcatcggtgcct

tcaacaagtctttcaacgcatagacaagttagcttgtacaatttgagaccactgttttctttttcttttcaatcagagct

tcgggttggctgagagccttcgaggaaccatagcgggtgtccttttggttttgcaacctggt

>Hypothenemus_hampei_contig_19160|c0_g1_i4 ATP synthase alpha mitochondrial precursor

gtgtctcgtgtcggttccgccgcccaggtcaaggccatgaagcaggtcgctggttccctcaagctcttcttggcccagta

ccgtgaagtcgctgcctttgcccagttcggttccgatctggatgcctccaccaagcagacccttgcccgtggtgagcgtc

tgactgagctgcttaagcagaagcagtactcccccatggccgtttccgacatggttcccctcatcttcgccggtgtcaac

ggtttccttgaccaggtccccgtcgccaagatcctccagtgggagtccgacatccttgctcacctcaagagcaaccaccc

cgagatccagcagacca

>Hypothenemus_hampei_contig_19207|c0_g1_i1 U6 snRNA-associated ribonucleo

aagggggccgcggcggtccgaatcaaagaaaccgacctgcagcaagatgctgttctacgagttctttaggtccagtgtgg

gaagccgggtttttgtgatgctgaaggcgggtgtgtatgtgtccggggtgcttgagagcatagacccgtatctcaacgtg

aatctgtcggacgcggaggtgctcagcatgcatcctgggctgagcggggtgtctgtgtgctcgatccgggggtcgtcgat

aaagtacatccttgccgagaggaacgacgagttggtgcggggcgtgaatgccgggagcaggctgcggatgatccttgaca

agtgttactaatgctggg

>Hypothenemus_hampei_contig_19223|c0_g1_i1 60S ribosomal L33-A

ccgatctaatgactgaatctcacagattatacgttaagggtagacacttatcttaccaacgttctaagaacgttaacaac

ccaaacgtttctttgattcaaattgaaggtgttaacaatgctcaagatgctaaattctacttaggtaaacgtattgctta

cgtttacagagcttctaaagaaatcagaggttctaaaatcagagttatttggggtaaagttaccagatctcacggttcta

acggtgttgttagagctaacttcagaaaaaacttaccagctaaaactttcggtgcttccgttagaattatgttgtatcca

tctaacatttaaattaatttatcatcttatcaatgattaatttatcttacttacctttcaccttattgtatgttaaataa

tacgaattatatattataataaaaactcttata

>Hypothenemus_hampei_contig_19240|c0_g1_i1 hypothetical protein

ctttcgcaggtaggaggcgatctcggtgtccaggggaggtgcagactgtctgtatattccttggagactctcgaactcct

gggtgtctacgaggcgaatgcacactactcaataagagacgtgctgataacgtctctcgaccaggaggtttctctgtatc

gccgaggggtaaaggtacttgagatctcgatgccgggaagaataagtagactatgcacagacccactgctaacgaacata

tattgcggggcgcatgatggaaagatattctgctgcagcatgaactataaagaccccactacgatggtgtaccacaagag

cagaatagtgggcctcgaggtcagcttttgtggaggatacctttattcagcagatgcagagggtgttgtctgtatatggg

atacaagaaccaatgtcgtggttgggaaagtggacatggagtcggagataaggggaatgcagatggtgtatgtatccgaa

tggcaaggaaacttggatgcagtcctgagcgagcttgtggggccgacgggataagtaaataagg

>Hypothenemus_hampei_contig_19242|c0_g1_i1 hypothetical protein EROM_110580

cagagcctgttctgctacatgacgggcgcaccttcggggtgtggagcaacccagagggcgtctggatctcgctcgagcct

cgtcttgaggtcgaatgcccgacaaatttcctgatccactttgcagactttgccttcgagaggagcgccctccttaggct

tgttgcagccagggatagagagggtgtggaggcaatccttgcgagatacgtgatctttgatgcacccttgcagctcccgg

accccaggttgcttgtgcctgcggctaaatgtggcagagcgcatgccgaggagcagcagcaggacgtctgcttcgagtac

ctcttcagtggagagacatacaaggacacaatggattgcattgagatataggcaaaggctcaagccgagccccttgatga

agcccgggaaggtagccatggagtttctggtagagggcggaagggtgctctgtagggccgtggcaaggacgatctccgag

gtcatggccggaggtaggatgtcgagggcagaggccgagctgattcttgatgtggcgccgaggcagggtggcgacgtggc

cagggactcgttccttaggatgtacagcgcaaacgcaagggagaacggcgggtcgccatacctgcagtcgaagatgcttg

ctgcatacagcgtgctgtctgggtccaagcccgacccaaagtcccttggggacgcttgttggaagcgctctccacagcgg

cag

>Hypothenemus_hampei_contig_19247|c0_g1_i1 unnamed protein product

ttcttgaagtcagtcattcattcgaggttgatcctccattactgtcactttagtatctctgtaaatcagttgactacttt

tgtcttaccactttcactccctcaatttacaatcttccatcatgactttcaacgtcttccttaccggccttctcgctgcc

accgccgctgctctcccgcactccgctcgatccggagaatccggcgccgttgagacccgcgattccagcggcggcagcct

tcagattgtcaacaacttggataccaacgtctacctgtggaccacctcatccgattctgggtccatgaagaccctcagca

ccggaggtgactacagcgaagactggatcaccaactccaacggtggcggtatctccatcaagatgtcgaccagcgagagc

gaggatagcgttcttcagttcgaatacacccaggatggtgacaccctttactgggacatgtcctccatcgatctcgactc

gacctccgagtttgtcaagtcgggcttcaccgttgtccccagtgactcgagctgcaagactgtttcttgtgccgctggcg

attcggactgttcggacgcttatcagctcccagatgacgtgaacacctactcttgctctctggacgctactttcactctg

actctgggatgaattctcgtctttttcttctctcaaccggcgggaccgatggatttttgtttttttttgtttttttcctt

atgatttgcagaatattactgcctccttgggtttgatgaaccaactcttggatatgtatataaacttctgcagaactcga

tgttt

>Hypothenemus_hampei_contig_19278|c0_g1_i1 60S ribosomal L12

atcaaaattcgacccatcagaggtgaagttcatctacttgagagccgtaggaggtgaagtcggtgcttcttctgcacttg

cccctaagatcggacctttgggtttgtctcctaagaaggtcggtgaagatatcgccaaggccaccaaggatttcaagggt

atcaaggttactgtgcaattgagaatccaaaacagacaagctactgcttctgttgtcccatctgcatcttctttggtgat

caccgccttgaaggaaccagtcagagacagaaagaaggagaagaacgtcaagcactctggtaacattccattggaccaaa

tctttgaaatcgccagacaaatgaaggagaagtccttcggtaagaacttggcttctgtcaccaaggaaatcttgggtact

gctcaatctgt

>Hypothenemus_hampei_contig_19314|c0_g1_i1 hypothetical protein ECU09_1100

aacattgcggctcagaagtaccttgcgaaggaaataactgacgaggaggtgaggaataagacgctgaagagaatagacct

tctatgtcttgagcctgaagagttcgaggtgagcgaggtaagaaacagaggaaaggccctaggaattcggacgagcaacg

agtgttccgacagaatggagcgagatattctcgagtactcaaagaggctccatgggaaggttaagaagtttgcagagagt

gtcgatcttgatagcaaggtgctaagagaagttacagacaagatgtcgaaaaatctggcagggacgtcttcggcgctaag

atctcttcggatggacgggcacaatgttcctgtcctacggatcctggtctctgcaatggctatttttgttgtaatgtact

tcattataaggtttttgtagaaggctcgtggccgatgtttaatgaactgtcggcttgcttactaaactttgctcata

>Hypothenemus_hampei_contig_19327|c0_g1_i1 synaptic transmission and general secretion Sec1

ccaggacgagctatggaggaggaacagtaacgagtactttcctgtcgtggtcgagagggttgagaaggagcttcttgagt

acaagaaggagatggcgcttagaagcgtcgacgagaggacagacaagaaggccatccaggaggttcttgaaaaggccccc

gagctggcgaagagaaacgagtctgtgaatgcacatatatcgatatgctccgagatggtcgagatcataaaggaaagagc

tattgacgacttctacaaggtcgagaggggaggccatactagccaggagcttctggaggtgtctgagaagggaagtgacg

aggacattctgaggctggcgatcctactgctcaacaccaaggactatgaccttattgacccgctgctgcagaagagggga

atcaaatcgggggtggtcgagtttttcaggaagcacaagagcatccgaccggagaagacggggactttttactcacaggt

ggtttcaaacctcatgggcaacgttaagaagcttttgcctgtgaaggagcagacgcccgtgtcgtctcttgcagagtctg

tgtatggcgatatcaagtcgcaggcatactcgagcctcaacgtgttcgaccccacaggctcgaggaacatctatgcaaac

gagatctccagggttgttgtcttttcggtcggaggaggaacatacacagagctgaagaccctgaagctcctggaggagag

aatagaagtgcccatcatctacgggtccacggagattgtgaatgcaagggaatttctgcgccagagat

>Hypothenemus_hampei_contig_1935|c0_g1_i1 UTP--glucose-1-phosphate uridylyltransferase

acgtcaacgagttcaagtctatcaagaagttcaagtacttcaacaccaacaacatttggatgaacgtccaggctatcaag

cgtgttgttgaggagaacgagcttgagatggagatcatcgccaacgagaagtccatccctgctgacaagaagggcgaggc

cgaccaggctatctaccagctggagaccgctgtcggtgctgccatccgtcacttcaagaacgcccacggtgtcaacgtgc

cccgtcgtcgtttcctgcctgtcaagacctgctccgacctgatggttgtcaagtctgatctataccgcctggagcacggc

cagctggtcatggaccccaaccgtttcggtggtgtccccgtcatcaagctgggctctgacttcaagaaggtctccgactt

ccagaagcgcatcggcagcattcctcgcatcgtcgagctggaccacctgaccatcaccggt

>Hypothenemus_hampei_contig_19369|c0_g1_i1 40S ribosomal partial

gatctcacatacatccaaaatggctgacgctgctcctcgtggacgtggtggtttcggttcgcgcggcgaccgtggtggtg

accgtcgcggtcgtggccgtggccgtggccgtcgtggtggaaagcaggaggagaaggagtggcagcccgtcactaagctc

ggtcgtctcgtcaaggccggcaagatcaccagcatggagcagatctacctgcactctctgcccatcaaggagtaccagat

tgtcgacttcttcctgcctaagctgaaggatgaggttatgaagatcaatcccgtccagaagcagacccgtgccggtcagc

gtacccgtttcaaggccgtcgttgtcatcggtgactccgagggtcacatcggtctcggtatcaagacctccaaggaagtc

gctaccgccatccgtgccgccatcaccatcgccaagctggctgtcctccccgtccgtcgcggttactggggttccaacct

cggtgagcctcactctctgcccgtcaagcagagcggcaagtgtggttccgtctccgtccgtctcattcccgccccccgtg

gtactggtctcgttgcctcccccgccgtcaagcgtctcctccagcttgctggtgtcgaggatgcctacacctcctcttcc

ggttccaccaagaccctcgagaacaccctcaaggctaccttcgtcgccgtcgtcaacacctacggtttcctcacccctaa

cctctggaaggacaccaagctcatccgttcccctctggaggagttcggtgatgtcctccgccagggcaagaagtactaga

gtgcgatttgcgacgatgcatgatttagttccgagtttctttaccccccttcgagctggatgacgaaaagtggcttgcgg

gttgattccccctcgggctccgggtgtcctttggatgtatttatcgggatggg

>Hypothenemus_hampei_contig_194|c0_g1_i1 60S ribosomal L4-A

cgaaacctgttccgttaagcacttaggtttgttacaattggctccaggtgctcacttgggtagatttgttatctggactc

aaggtgctttcgaatccttagactctatctacggttccgaatctaccaagtctgtcaagtctaactacgctttaccatct

aacatcatcaccaacactgatgtcaccagattgatcaactcttctgaaatccaagctgttgttagaccagctggtcaagc

cactcaaaagagaactcacgtcttgaagaagaacccattgaagaacaagcaagtcttattgagattaaacccttacgcta

agaccttctcttctgaaaaattaggttctgctaaggctgaccaatctaaggct

>Hypothenemus_hampei_contig_19540|c0_g1_i1 40S ribosomal S20

gtagtctttgaacggccatcatcaccttaccgaaccccgaagttcaggtcttgattggcaattagcctcgtttgtgccat

ctcaatcccaccggtctcatccaacctcagtcaagatgtctttccagaagcccgagaaggatttcggcgagggccccaag

gtccacaagatccgtatcaccctcacctcccgcaaggtcgctgcccttgagaaggtgtgctcggagctcatcgaccgtgc

ccgctccaaggctctccaggtcaagggccccgtccgtctgcccaccaagaccctgcacatctccacccgcaagaccccca

acggtgagggttccaagacctgggacaagtacgagatgcgcatccacaagcgtcttatcgacctgctcgcccccaccgag

accgtcaagcagatcatcatcaacatcgaggctggtgttgaggttgaggtcaccattgctgcttaaattaattagctcac

gcaatggaccagggagggtcctgggctggtctatgtaatgccggtcgttatgatatgctggtgttttcaggactggtcgt

ttacttttgagccttattctgggaattttggtagatacccgttcgtacctg

>Hypothenemus_hampei_contig_19540|c0_g1_i2 40S ribosomal S10a

aggtcttgattggcattcgccttaacaccagcaaaaacaaaaactcaagatgtctttccagaagcccgagaaggatttcg

gcgagggccccaaggtccacaagatccgtatcaccctgacttcccgcaaggtcgccgccctcgagaaggtctgccaggag

ctcatcgaccgtgctcgctccaagtctctgcaggtcaagggtcccgtccgtctgcccaccaagaccctgcacatctccac

ccgcaagacccccaacggtgagggttccaagacctgggacaagtacgagatgcgcatccacaagcgtcttatcgacctgc

tcgcccccaccgagaccgtcaagcagatcatcatcaacatcgaggctggtgttgaggttgaggtcaccattgctgcttaa

attaattagctcacgcaatggaccagggagggtcctgggctggtctatgtaatgccggtcgttatgatatgctggtgttt

tcaggactggtcgtttacttttgagccttattctgggaattttggtagatacccgttcgtacctg

>Hypothenemus_hampei_contig_19540|c0_g2_i1 Ribosomal S10

aggtcttgattggcattcgccttaacaccagcaaaaacaaaaactcaagatgtctttccagaagcccgagaaggatttcg

gcgagggccccaaggtccacaagatccgtatcaccctcacctctcgcaaggtcgccgccctcgagaaggtctgccaggag

ctgatcgaccgtgctcgctccaagtccctgcaggtcaagggtcccgtccgtctgcccaccaagactctgcagatctccac

ccgtaagacccccaacggtgagggttccaagacctgggacaagtacgagatgcgcatccacaagcgtctcatcgacctgc

tcgcccctaccgagaccgtcaagcagatcatcatcaacatcgaggctggtgttgaggttgaggttaccattgccgcttaa

atgcgctacccttaggtagttgttacaatggctcagggagggccctgggctagctacttggtgtgaaacgagctttggat

ctcgaggcttgccaatacggaggagttggccgtttgctgtgagcccgaaagggaattgatggcagatgcccgttac

>Hypothenemus_hampei_contig_19563|c0_g1_i1 60S ribosomal L9-B

gtcccagaaggtgttaccgtcgctatcaaagctagaaacatcaccgttgttggtccaagaggtactttaaccaaggactt

gaaacacattgatgttacttttgaaaaaattaacaacaaagctgttaaaatcattgtccacaatggtgacagaaaacatg

ttgctgctttaagaactgttaaatctttgatctctaacttgatcaccggtgttaccaaaggttacaaatataaattaaga

tatgtctatgcgcatttcccaattaacgtcaacattgttgaagaacaaggcgaaagtttcgttgaaatcagaaacttctt

gggtgaaaagagagttagaagagttaaggttcacgaaggtgttaccattgctccatctgctaaccaaaaagatgaattaa

ccgtcaccggtaactctttagaagctgtttctcaaaatgctgctgatatccaacaaatttgtcgtgttagaaacaaggat

atccgtaaattcttggatggtatctatgtttctgaacgtggtactattgaaactgattaaattaattagtttttcataat

ttacaccccttccctctaaaaagtgtttttttgtattaatacaatttctcttttgtctatattttcatttccttttgttt

tttatgagttttcagggttatatagttaattttggttttat

>Hypothenemus_hampei_contig_19572|c0_g1_i1 60S ribosomal L5

tcaagaaggatgaggatgagggctccaagaagaccaaggaggagtggaaggccgagtccaagaagtacaaggtcgagaag

ctctcccacgagcagcgcaaggcccgcgtccaggagaagattcgcgagctcgctgcttaagcgccttgtcggttttattc

ctgatgagtccttcggtccgctgtttaagctcaaaagtttcctacaaaaatttaatgagggaattggctttgccagtcgg

cgtttcttttttctgatgaatgtagattgatggatcatggcacataccacttttgtttacgttaaagggtgttttctggg

gcaactagttcggtgttcacaaggcaattttctttttcacagttctaatcgctcccaatgtccaag

>Hypothenemus_hampei_contig_19572|c0_g2_i1 60S ribosomal L5

tcaagaaggatgaggatgagggctccaagaagaccaaggaggagtggaaggccgagtccaagaagtacaaggtcgagaag

ctctcccacgagcagcgcaaggcccgcgtccaggagaagattcgcgagctcgctgcttaagcgccttgtcggttttattc

ctgatgagtccttcggtccgctgtttaagctcaaaagtttcctacaaaaatttaatgagggaattggctttgccagtcgg

cgtttcttttttctgatgaatgtagattgatggatcatggcacataccacttttgtttacgtttaagggtgttttctggg

gcaactagttcggtgttcacaaggcaattttctttttcacagttctaatcgctcccaatgtccaag

>Hypothenemus_hampei_contig_19573|c0_g1_i1 ribosomal P0

accaggtaagacctctttcttccaagctttaggtgttccaaccaagattgctagaggtaccattgaaattgtttccgatg

ttaaggttgttgaagctaaccaaagagttggtcaatccgaagcttccttgttgaacatgttgaacatttctccattcacc

tacggtatgtctgttatccaagtttacgacaacggtcaagttttcccagcttctatcttggatattaccgatgaagaatt

ggttagccactttgtttctgctatcaacaccattgcttctatctctttggctgttggttacccaaccattccatctgttg

gtcactctgttgttaaccactacaagaacgtcttggctttatctattgctactgactacacttttgaaggttctgaagcc

gttaaggacagattagctaacccagaagcttacgctgctgctgctccagttgctgctgcttccggtgatgctccagctgc

tgaagaagccgctgaagaagaagctgaagaatctgatgacgatatgggattcggtttattcgattagagcctgtagtcg

>Hypothenemus_hampei_contig_19577|c0_g1_i1 60S ribosomal L24

tacccgccgtgccgttaagcaccagcgtcccatcgtcggtgcctccctcgacgtgatcaaggagcgccgcaaccagcgcc

ccgaggcccgtgccgccgctcgccagcaggccatcaaggacgccaaggacaagaaggccgcctccgagagcaagaagaag

gctgagaaggccaagctcgccgccaacaagggtggtgctcagcgcatccagagcaagcagggcgccaagggctctgctcc

caaggtcgccgccaagtcgcgttaagataaacgaaatcgaagcggtttaggcaaattggggctggggaaatttctttaaa

aagaggaaaaacatgttctttttttatttgacgctgggaaacttgggaggttattaaccggagccggaaatgaaattttg

tgtgtggttttcacgttactcactttacacaaaaccttcgaacctt

>Hypothenemus_hampei_contig_19577|c0_g2_i1 60S ribosomal L24

tacccgccgtgccgttaagcaccagcgtcccatcgtcggtgcctccctcgacgtgatcaaggagcgccgcaaccagcgcc

ccgaggcccgtgccgccgctcgccagcaggccatcaaggacgccaaggacaagaaggccgcctccgagagcaagaagaag

gctgagaaggccaagctcgccgccaacaagggtggtgctcagcgcatccagagcaagcagggcgccaagggctctgctcc

caaggtcgccgccaagtcgcgttaagataaacgaaatcgaagcggtttaggcaaattggggctggggaaatttctttaaa

aagaggaaaaacatgttcttttttttatttgacgctgggaaacttgggaggttattaaccggagccggaaatgaaat

>Hypothenemus_hampei_contig_19583|c0_g1_i1 Glucose-6-phosphate 1-dehydrogenase

taaagttgaaataagaattcaatttaaaccagttgctaaaggtatgtttaaagaaattcaaagaaatgaattggttataa

gaatacaacctgatgaagcggtttatatgaagattaattcaaaaatccctggtatatcaactgaaacttctttaactgat

ttagatttaacttactcaaagagatactctgaagatttttggattcctgaagcttatgaagctttaatcagagattgtta

tttgggtaatcattctaatttcgttagagatgatgaattggatgtttcttgggcattgttcactcctcttttgaaatata

tcgaatctgataatttccctaaacctgaaggttatgcttatggttcaaaaggtccaaaaggtttaagacaatttttggaa

aaacacaattatgctttcagtcaagaaggatcttatcaatggcctttaaccaccccaaatatcaagggtaaaatttaatg

cttt

>Hypothenemus_hampei_contig_19593|c0_g1_i1 Glutamate Leucine Phenylalanine Valine dehydrogenase

tcatgttcggccagtacaagaagattcgcaaccagtgggagggtgttctcaccggcaagggtctcagctggggtggttct

ctcatccgtcccgaggccactggttacggtgttgtctactacgtcgaccacatgatcaagcacgcctccggtggtaagga

gtccttcgccggcaagcgcgtcgccatctccggttccggaaacgtggctcagtacgccgctctcaaggtcatcgagctgg

gtggatccgtcgtgtccctatctgacagccagggttcgctggtcgccaaggatggcagcagcttcacccccgaggagatc

gacatcatcgccaagctgaaggtcgaccgcaagcagatcgcctccatctccggcaccgagaccttcgcctccaagttccg

ctacatccccggtgctcgtccctggatccacgtcggcaaggtcgacg

>Hypothenemus_hampei_contig_19593|c0_g2_i1 Glutamate Leucine Phenylalanine Valine dehydrogenase

gttgtctactacgttgatcacatgatcaagcacgcttcgggcggcaaggagtctttcgctggaaagcgcgtcgccatctc

cggttccggaaacgtggctcagtacgccgctctcaaggtcatcgagctgggtggatccgtcgtgtccctatctgacagcc

agggttcgctggtcgccaaggatggcagcagcttcacccccgaggagatcgacatcatcgccaagctgaaggtcgaccgc

aagcagatcgcctccatctccggcaccgagaccttcgcctccaagttccgctacatccccggtgctcgtccctggatcca

cgtcggcaaggtcgacg

>Hypothenemus_hampei_contig_19624|c0_g1_i1 40S ribosomal S2

gtaccggcctggttgcctcccccgccgtcaagcgtctactccagcttgctggtgtccaggacgcctacacctcgtcgtcc

ggttcgaccaagacccttgagaacaccctcaaggccaccttcgttgccgtcgtcaacacctacggcttccttactcccaa

cctgtggaaggagaccaagctcatccgcagccctctggaggagttcggtgacgttctgcgtcagggcaagaagtactaaa

aaaatagtgcagtgatgggaataaatttgcatgattgtttttgaaatcgaaccattttcctacggggttttcttctactt

tttcctcttcttttggctgtaggatcggcaaggctccagcgaactcgacacttaacgcttttac

>Hypothenemus_hampei_contig_19637|c0_g1_i1 ER lumen retaining receptor

aacacgctctgtctcgaacctgctgtacaccatgtccctcttcctggagagcgttgccatccttccgcagcttgtccagc

tgcaggaggccggcgagtcggagacaatgacctccaagtacgtcctgctgctcgggctctacagggccgcctacaccgtg

tacttcgtcgtcaacaggctgtacggcacaaggaggatcgggaacatccccattgcatgcggcgttgtccagaccctgct

gtacctggacttctttgtgatctactacagatacgtcttcaggcggtccggactatccgagcggctcccctcgtcggcca

ttaaagacgagctataa

>Hypothenemus_hampei_contig_19694|c0_g1_i1 GENERAL NEGATIVE REGULATOR OF TRANSCRIPTION

cataagcaaggacaagatgagggaaatcgtgtctatagtcagggaaaagaagtaccaggacccatcgacatactacccgc

cgtccttgctcttgaggatgcttccaaactgctatccaaaggcgccgaacgaggggattttcaaggttaacatagaggac

atgaagatggacaacctgcacgaggagacgctcttctacatattctactcgtttccaaacgacaagctccaggtcaaggc

gtatgataacatcctcaagagaaagtacgtcttctgcaaggtgtacaaatgctttgttgtattcaacttcccactggtgg

cagaccatgtcaagaggccgatcgtgatgttcgacccattcagctggtcaaaggtgtcgacagaagttgtcttcgacgag

aagttcgtgcgatccctggagagataaga

>Hypothenemus_hampei_contig_19697|c0_g1_i1 transcription initiation factor TFIIE subunit beta

gagacacacaaacacatacatccatgccgtaattagcctcctgaagcagcatgataggcccttgtcgttcgaggagatca

gggagaagacgcggatcgatctgcacaacaactacatgctcctgcagtcgatcaagaagaacccgaggattgttgcgaca

cacagcacgctgatgttcaagccgctgtactccataagaagcatcgaggacatgaggagggttgtgcgtggcctcaacgg

ggaggaagggctagagatggacaagctcatggactcgcctgtcgacgtcacgccctttgtcgaggagctccggaggaccg

gggaggtgattgttctgagcgacatggacgggtccgaagttgtgttttggaacgatacggaagagaagcctgttgatccg

cagatacgggacctgtggggccaggtccggatatccacataccacgacctcatccgggagctcaacacggcagggctgaa

gacagaaaaggtcgagaacatcaagaagaagtcgatgatcaagatcaagaagggaaagaaaaacagaaggaagatcagga

taaccaacacgcatgtgaagggcctggacctcagcggcatgcaggacgatggctgagggtctctgggagcgagtccatct

ttcgagtgcggagatcg

>Hypothenemus_hampei_contig_1973|c0_g1_i1 BELONGS TO THE NAP SET FAMILY

gggagatcgacgacgagcgcaggaggcttgcggcgcgggacttcgagctcaagctgtcgcaccacgtggcgatcaagccg

ctgctggccaggagggacgaggttgtgcgcagcggcgcggggtgtgcaggatactgggcgcgcgcgctgggcagctacgc

ggccggggcggcactgcttccgcgcgacgcacacaaccgcgctgcagccgggtggatgaggtctctggaggcagactacc

tgcccgggctggcgtacagggtgcacgtcgagctgaacgagaacgagtttgtgtcgaacaggtggctgacgaagagcatg

cggctgggcgcgcgcgaggcagagaagactgcggttgtgtggagagacaacaggaggtggcctgtgtttgagttcttcga

gaccgatgccgaggacctggacgtcttcgacatcctgtacgagctctacgtcaactcggcgtcgtacttcctgggctgcc

gcgtgccctgactgcccggaaatatacttttggactcaaa

>Hypothenemus_hampei_contig_19764|c0_g1_i1 hypothetical protein

cacagggtcacaagaacgaagaacacgtggaaatgagcaatgttgaatatgatcaggagaaggaaggaaaagaccaggac

agacatgaatatctggtctgtgctgaggatcaccgagtccctcctcttcttgagcgggttgtggctgctgttgttgagca

aatggacggcctcgactagcgagcctgttagagcggagtacatgcgcgagaagacagacaagcacatggtggttgggacc

atgtggagcccgcccacaagcacaactgggcctcccagcctcaggagcatcttctgggcagtgtaaaaaggccgcaagag

ggttcctagtgttgttttgatcgttatggcgtctcctgcctctgccgacgcatctgctatgtgtgctatcacacgccgcg

gggcctcggccaggaagtactcgacggcccctcttgccagccagagggccacggggacgaggaagcagctcaccctcctc

ttcctcaggcgcatcacagccttgtccgtagtggcggccggggccctggtggccagcctcgcaagaagatggctcttcga

caggaagaagtcgtatggccggggattgggagaaagctcgacaaactcaagggtgccgtccatgggatacacggtctcca

aata

>Hypothenemus_hampei_contig_19803|c0_g1_i1 Tat binding 1-interacting

aggagagcgtgttccagatgatgtacagatgcaacaggccgacgtctaagacagaggtggcggcgtacttcaagggggag

atgggagggacggcgatacaggaggctctggaggggcttgaggagagcggaaggctggtgacgaaggtctatgggaagtc

caagatatatcttgtcaaccaggaactgttccaggacgaggaggacgtcgagctggacaaggagatccaggggcatgaga

aggagatcaaagcgctgagggaagaggccgattctgttgatgccgagattaagatgcttgagaagatgctgtctattgag

aagctcgaggagagcattgagtcgctgggcaaggcgatcaaaagtaacagcgagagactcgaggggcttagaagcggagg

ccgcgaggtgacaaagaaggacatgagcattgcaaggaaggggtatgaaaaggcacagtccacgctcaagaagatcagaa

ggaccttcaacgaggttgttgagaggctttccgaaggcatggacatgaagaagtccgacctgtacgaggaaatcgggatt

gaggcgtagcggcgccccagaaaagcattaaatcaatggcatttttgtagaagaactgccagccctgatgagggacaaca

agccaataatcctgataaagaaaaaggttgaaggccaggacag

>Hypothenemus_hampei_contig_19831|c0_g1_i1 probable chaperone HSP31

ctttgcctcggcgggccacggcaccttgtttgactatcccaaggccagtggcttgcaagagcttgcccagaagatatatg

ccaagggaggagtcgttgctgctgtgtgccatggaccagcaatctttgccaacttgatcgacaccacaactggcagacca

attgtcgagggcaagactgctacaggcttcaccgacaagggagaagaacagatgcaagtggacacgatcatgaagagcaa

aaacttggagtcagtggaagcgctctacaagagattgggtgcaaaattctcggcccctgccggaccatgggacgaccatt

ctgtggtagatggcagaatagtcactggtgttaacccacagtcggccacttccactgctaaaaagg

>Hypothenemus_hampei_contig_1988|c0_g1_i1 hypothetical protein ECU08_0790

ggaggtgattgtgcggggaaggttttccgggcatgtggagccatgccttcgagaggagtcgaacgagcccacgtacatcc

acatcgttgcgaagaccaagggtgagataagagaggcaaggcgggcatgccgggaggcgattggaaagtacgcgaagaca

tagggattggtgtctttgcaagggttctgttggtggctctgcatggcgaccatcgattggcaatggtttgatgtcctgga

tcagaattctgaaacagagttgctataaagacagcttgtttattagatgtctaatggatgcaggtgctctggagtctttg

ctagcaagccggctcaagatcgtatttggtggtgcggcatgcccttttccctgcccggtgcttgatgcagagacggttga

ggtgtttacgcttgagagattcaagaagctgatcgtttcagcagcaaaagaagggcgggacttcatcattgcagaggtga

cgacccaggacccgtcgac

>Hypothenemus_hampei_contig_19918|c0_g1_i1 sulfonate biosynthesis enzyme

ttccattggccacgtttctaacatgtccacatatagattcgatgtgatcgaactatcatccggcttcttgtcgttcccgg

aagatgactggcttcgtctggtcgacaaggtccattcttacaagttgaaagcgaaacctgagttgggaattcagttcggc

gctggcggcgacaccccggcctctggacttgaggcaattggcacttccgatccgggaaagctggttaacctgggccagaa

gtttttgaatgccggcgttgagcgcctgatgatcgaatccgagggtatcacagagaacgtggagtcgtggcgcaccgacg

tggtgtctaagattatgaaggagcttccatctgagcgcgtcatgt

>Hypothenemus_hampei_contig_19928|c0_g1_i1 vacuolar ATP synthase subunit c

acgccgtcgagaaggtcagcagcgacgacgagtactgcctgatcaagctctatgtcctccatcacagcgaggaggatgtg

agggccatgatgcatgcagcgggctttgcaatccgggacgtcgacgaggctgggatctcctcggaggagatcactgcaag

gagaaggagggcagaggagaggttctcttctgtggagagggttctgacgacgtttgtgcacgtccatctgacggagattc

tcaaggtgcttgtccatgtcaagctcctgcggctattcgtcgagtctgtctatagatacggccttcccacggagtacgtg

ttctttgtgaccagtggcgaaaagacaaaggtcctgagccagtggacagccatagccaagggatggccttctgatagagt

cgtttacgaggaggacgcagacaaccacgacgaaaacgaggttttctttgcgttttcggagatcggcacatacgacgacg

aggagtagccaaggcatgccgacctcctgcagctggggaa

>Hypothenemus_hampei_contig_20010|c0_g1_i1 tyrosyl-tRNA synthetase

gcttgatcacaaggaacctccaggaggtgcttggactggacgacctaaagaaggtggtgtccgagaaggagttgagtgta

tactggggaacggccataaccggaaggccgcacatagcctatctaattccgctcatgaaggtcaaggactttgtggatgc

cgggtgcaaggtcaagatcctgcttgcagacatccacgggttcctggacaacctcaaggcgccgatcgagaaggtccggg

acagatgtgtgtactacgagaggctgatcaagtctgcactcaggatgctgggcgtggatcttgggagggttgagtttgtc

aagggaagcgactaccagaggtccgaggcatatgcaatggacctgtacaggatcctatccctgacgtccgagcatgatgc

gaaaaaggccggggcacaggttgtcaagcaggtggagaacccgatggtgagctcgctggtgtatccggcaatgcaggctc

tcgacgaggagcacctgtgcgtggatgcgcagttcgggggagttgaccagaggaagatattcacgtatgcacggaagtac

ctcccggagctcgggtacaggaagaggatccacctgatgagcccgatgcttcctgggctgaactcggacaagatgagcag

ctcggacgagctgtcgaagatcgacctgatggactcgaaagaggcgattgggcggaagatcaagaagtgcttctgcagcg

agggcgacaaggacaatgggctgatgacgattttctcgcacgtgatcttccctgtggtgcagggaatgggggagcgggtc

gggattgtggatagagacgggaaggagacggtgttttccgagtatctggagcttgagagggagtttgttaggaaggagat

ccacccgggagacctcaagagcaatgcggcacggctgatcgagcgcattgtggggccgatacgggaggagatggagaagg

acatggaggttg

>Hypothenemus_hampei_contig_20021|c0_g1_i1 MBF1_DEBHA ame: Full=Multi -bridging factor 1

gtaaagtgatttctcaagccagacaagagaagaaattgacccaaaaggatttagccaccaaaattaatgaaaaacctcaa

gttatcaatgattatgaagctggtagagccgttccaaaccaacaattattggctaaaatggaaagagctttgggtgttaa

attaagaggtaaacaaattggtgaacctttattcaagaagaaagcttaggttttggatttgtagatgatatgacgaatac

acgttaaaccaaaaccatgatttacccgtgatagattaaggtataagctcagctagaggattagtagtgtctactgttac

gccaccactataacagttgcagctccgactgaaggcagcaggataaaaactttactgccatccaggtgggggtgcttata

aactctctgatcaaccgattgcaaaccatcagtttgttgaggagg

>Hypothenemus_hampei_contig_20022|c0_g1_i1 NOG1 nucleolar GTP-binding

gttgcaaagctgtgcgatgttgaggacaaggagatgattatgagctttctggcggggaagaagtacatggagatgagctc

ggcaaaggaggagaacgtggatgcagtcaaggcaatggcatgcgacctcctgcttgacgagaggtttgagaagaagatca

acagcgagaagctttctgagtacattaaccggattgcggtcattcgacccaaggaagtgcgagagaaggcggagtcgttt

atctgctccagagagataacggagattgagaatgagcaggaaaggtactttgttgctccggagtacaagtacgacattgt

gcccgagattgtcgacgggaagaacgtggctgatttcttcgacccggacatcgagaagaagctcggggagattgaggagg

aggaggagaagctgcttccgctctactgcaagacgtacgacgtgctgtcacccgaggagaggatggccaaggacgaggtt

actgttgggatcgagaggaggaggatcatcaacaggctcaaggagaagaagaggcttccggactcgtggaagcagcggtc

caggaacagcgggggagatgtggctcctgtccgaagggcagaaggccggacacaggtgatgcagcctccaagggccccga

gcaagaagaaggccaagttcgacgacaagcagtactacgacaagaagccgaagcacctctac

>Hypothenemus_hampei_contig_20022|c0_g2_i1 NOG1 nucleolar GTP-binding

gttgcaaagctgtgcgatgttgaggacaaggagatgattatgagctttctggcggggaagaagtacatggagatgagctc

ggcaaaggaggagaacgtggatgcagtcaaggcaatggcatgcgacctcctgcttgacgagaggtttgagaagaagatca

acagcgagaagctttctgagtacattaaccggattgcggtcattcgacccaaggaagtgcgagagaaggcggagtcgttt

atctgctccagagagataacggagattgagaatgagcaggaaaggtactttgttgctccggagtacaagtacattgtgcc

cgagattgtcgacgggaagaacgtggctgatttcttcgacccggacatcgagaagaagctcggggagattgaggaggagg

aggagaagctgcttccgctctactgcaagacgtacgacgtgctgtcacccgaggagaggatggccaaggacgaggttact

gttgggatcgagaggaggaggatcatcaacaggctcaaggagaagaagaggcttccggactcgtggaagcagcggtccag

gaacagcgggggagatgtggctcctgtccgaagggcagaaggccggacacaggtgatgcagcctccaagggccccgagca

agaagaaggccaagttcgacgacaagcagtactacgacaagaagccgaagcacctctac

>Hypothenemus_hampei_contig_20025|c0_g1_i1 unnamed protein product

cgagtcccttatcattgttcagtacatagatgaagtgtggcatgacaagaatccgttgctgccatctgatccttaccaga

gagctcaagctaggttctgggctgacttcgttgacaaaaaggtttatgattgtggaaggaggatctgggccacaaaggga

gaagaaaaggaggcagccaagaaggaattcatagggattatgaaaacattggaaggagaacttggaaataagccttactt

tggaggggaagactttggatatgtggatgtggctttgattcctttctactgttggttccatgcctatgagaactttggga

atttcaagacagaaacggagtgtccaaagctggtggaatgggccaaaagatgcatgcagagggagagcgtttccaagtcc

cttgctgatcctcacaaggtttacgaatttgttgtgtccttgaagaagaagcttgggatcgaataaagtctctttcaact

ttttggctaaatgg

>Hypothenemus_hampei_contig_20072|c0_g1_i1 1,3-beta-glucanosyltransferase Bgt1

ccaatattgaaacgttgaactaattccggccgacggcacctacgatgatatcatctccagccccaagatcaactacttct

tggccaatggcttcgcctactggcagggtcaggaaatcggcaacgccaccaacacctatttcgacgacatgtcccaggcc

aagaagcgtgtcgagagccgtgctgctggcagaaaaatccgtttcggcaacggcgaaaccggctggcctaccgatggcgg

aactgactacgaggccgccattgccaagaccgagtatgccgcaaagtactggaaggatgccgtctgcgggatcctcacct

ggggcgtcgacgtcttctactttgaggcgttcgacgagccctggaagccggtcagcaagggtgacaacgacgaggagaag

gatgagactcactggggtctgttcacctcggaccgcaagcccaagttcgattattcgtgccctcaataagggcgagactt

cttccgtgtgaatatttctataccttgaaaggtggagatcctggagggatctgttacgatggttaaattcgtttgtatca

tatcatgctcacggagtaataaatataaccgatgaaaaggggaattgtacgaagcatatgttgttcac

>Hypothenemus_hampei_contig_20072|c0_g1_i2 1,3-beta-glucanosyltransferase Bgt1

ccaatattgaaacgttgaactaattccggccgacggcacctacgatgatatcatctccagccccaagatcaactacttct

tggccaatggcttcgcctactggcagggtcaggaaatcggcaacgccaccaacacctatttcgacgacatgtcccaggcc

aagaagcgtgtcgagagccgtgctgctggcagaaaaatccgtttcggcaacggcgaaaccggctggcctaccgatggcgg

aactgactacgaggccgccattgccaagaccgagtatgccgcaaagtactggaaggatgccgtctgcgggatcctcacct

ggggcgtcgacgtcttctactttgaggcgttcgacgagccctggaagccggtcagcaagggtgacaacgacgaggagaag

gatgagactcactggggtctgttcacctcggaccgcaagcccaagttcgattattcgtgccctcaataagggcgagactt

cttccgtgtgaatatttctataccttgaaaggtggagatcctggagggatctgttacgatggttaaattcgtttgtatca

tatcatgctcacggagtaataaatataaccgatgaaaatgtaaaatttataataaattacaca

>Hypothenemus_hampei_contig_20072|c0_g1_i3 1,3-beta-glucanosyltransferase Bgt1

caggaactcttttgccctcccctttctttaccgatcttattttgattcggtccctctttctcttccttctctctaccttc

cgtactgaaaggactcgaggcggtttccttcgttccctcttgtcttgaattaactgcccgtccagctgattacctgtatc

ttctttcatttgactcttttaattgaagagtttcacaatgcgtatcgctgccttgctccccaccctactggcggccatcc

ccgccgttgccgccgaccgaggcaaactgggactcgccttgggcaacaagaacgtcaatggccaatgcaaacagaccagc

gactacgaggccgactttgacgccctcaagaaggtgaccacgttggtccgcacctactccgccagcgattgcgacaccgc

gaagaatatcattcctgccgccaagaacaagaaattccaggtggtgttgggcgtgtggcctgactatgacgagtccttca

acaaggatttcaacatcctgaagcagtacgtccacggcaacgaggacgtcgtccagggtatcaccgtcggttccgaggtc

ctgtaccgcggagacctccccgccgccaagctcaaggaaagaattatccaggtggtggatgaattccccaaggtgactgt

cggtacggtggacagctggaacaagttcgccgacggctcctacgacggcatcatcgccgaccccaagatcaactacttct

tggccaatggcttcgcctactggcagggccaggagatcggcaatgccaccaacacctacttcgacgacatgtcgcaggcc

aagaagcgggtcgagagcgttgccggcagaaaggtccgttttggcaacggcgaaaccggctggcctaccgatggcggaac

tgactacgaggccgccattgccaagaccgagtatgccgcaaagtactggaaggatgccgtctgcgggatcctcacctggg

gcgtcgacgtcttctactttgaggcgttcgacgagccctggaagccggtcagcaagggtgacaacgacgaggagaaggat

gagactcactggggtctgttcacctcggaccgcaagcccaagttcgattattcgtgccctcaataagggcgagacttctt

ccgtgtgaatatttctataccttgaaaggtggagatcctggagggatctgttacgatggttaaattcgtttgtatcatat

catgctcacggagtaataaatataaccgatgaaaaggggaattgtacgaagcatatgttgttcac

>Hypothenemus_hampei_contig_20133|c0_g1_i1 mitochondrial import Mas5

ctcactgctcttggcggcggtaccctcaacattgagcacctggatgaccggtggttgaccgtcaacatcgctcctggcga

agtcatcacgcctggtgccatcaaggttatcaagggccagggtatgccttcgtaccgtcaccacgacttcggcaacctct

acatccagttcgacgtcaagttccccgagaaggatcagctgcagaacctcaaccttctggaacaggtcctgcctccccgc

ctggagcagaccaagccgcccaccgacgctatggtggaggactttgagctggaggagattgacggtagcgagtactcgca

agcgcgcgcccacggtgctgccggcggtatggacgaggatgatgacgacgttcc

>Hypothenemus_hampei_contig_20145|c0_g1_i1 UTP-glucose-1-phosphate uridylyltransferase

caacttgaaggcggtgaaaaagttggtggagtctaatagcatccaggccgaaatcatccccaaccaaaaatccatttcca

agggcaattccgagttcaatgtgttgcaattggagaccgctgttggtgctgccatcagacactttgaaggtgcacacgga

gtggtggttcctcgcgccagattcttgccggtgaaaacctgttctgatttgttacttgtcaagtcagatttgttccactt

ggagcatggtgctttgaaattggacccagcaagatatggttttgccaacccacttatcaagttgggttctcactttaaaa

aggtttccggcttcct

>Hypothenemus_hampei_contig_20154|c0_g1_i1 short-chain dehydrogenase reductase family

acctactttcaacatgaactcccagaaccttttcgatgtcaagggcaaagtggtgctggtcaccggcggagccaaaggca

ttggtcgaatgatttccgaaggatttgtcgcgaacggcgctacggtctatatctcgtcgcgggatgccaaggcctgtgaa

gccgcggtaaaagagctcaacgctcttggaaagggcaaggcccattccattcccgccaacttctacaaagaggaagatgt

caagaaactggccgaggagcttggaaagcgtgaaagcaaactccatattctggtcaacaattccggatcgaactggggtg

ctccttatgatgaatacccctcgtc

>Hypothenemus_hampei_contig_20157|c0_g1_i1 TPA: Nuclear pore complex An-Nic96 (Eurofung)

aggaggcggccggtgatatttttggcctggaggatatccagaccgacattaccgaaatcggccaaaaagtattcaccaag

ggccaagatggccccggtggttacggaacgttcttcctcctgcaaattctcggaggcatgttcgagcaggctgtggctta

tctgggaacctataactctgttgtggctgtccattttgctatcgcattggcatactacggtctgctgcgcgtgtccgact

tctatgtttctggagatgaaattctttcattcacagttaaacagttcccgcaaataaacttcggatacctcatcacgcaa

tata

>Hypothenemus_hampei_contig_20166|c0_g1_i1 ribosomal S8e

cgctgccaaagatccacccgctgccagagaaggaagtgtttggggttgtcacaagtgggaaaaacaagtccaggcactgg

aagaggatggtcaccaagccttgctttgtcggaagcgacttcacaaggaagccgccgaagtacgagaggttcataaggcc

catggcaatgaggttcaagactgcgcacgtgagccatccggagctcagctcgaccttcaacctcaagatcatcggcctga

agcagaacccgcactccgaggtttacacggggcttgggatcttgaccaagggaaccatcatagaggtcgatgtcagcccc

ctcgggcttgtcaacggctccgggcacattgtgtggggcaagtatgcccagataacaaacaaccccgagaacacagggtg

tgtcaatgcagtcctgctcgtgtgacggagccctgcgtctccatgctcgatggtagggtgcctatgcccaggtagcgttc

ccgggcggtgcgccactcgactaaattaaacctagctgcttt

>Hypothenemus_hampei_contig_20168|c0_g1_i1 dihydroxyacetone kinase isoenzyme I

tttcatgaaatcttttataaataaattacaaatattaatcgctgatgaaaattcaaaatcagaaaatgttttcggtatcg

ctttacttcatggtataactaccttgtgtgagtttaccaaagccagaatcggtcatagaacagtgatggatgtcttgata

ccattctgtgaatcttttgctaaatcaaatgatatcaataccgcagtggaagttgcaagaaaagccgctgaaggtactag

aaaattgaaaccaaagttgggtcgtgctacttacgttggtggtattgataaccaaactgatttccctcctgatcctggtg

cttatggggtatacgaaatcattgatgctttaaaattgcttgctta

>Hypothenemus_hampei_contig_20173|c0_g1_i1 nucleotide-sugar transporter

acctgtccttcatcctcatggccgtcagctcctacgtgggcgacgcatctgacgttgttcctgctgcgggatatgcctgg

atatccgtcaacgtgttgtcgacaactgcatatgtgctgtcgctgaaagttgttgtcgatgcagacagtaaggccaaggt

cgagtccgtgtactactcgaacctcattgccatgccgatcctcggcgccctggcggccgtgttcgacgaacgagacgtcg

gaggcgccggcgtcaaggtgtctgtgtggatccttgtatcgtctgtctgtgcgttcttcacgtcgttcagcacggcatgg

acactgaac

>Hypothenemus_hampei_contig_20174|c0_g1_i1 ECU08_1425

gtcgagggcaaggccgtctactcgttcggcaaggtgcaaggaaaggcgggctgcggcaagtacgaggtggcgttccgcgg

gagaagcattgtgattgcaacagagaagcacctggaggcaggcgtgtggatccgcatgtacggcacgttccggtccggcg

tgctgaggacagtctttgtcggccggctggacggcgtcgacatcaaccttcttgaaaaggccgtcaggcacgtggcagag

catctgtagcaggcggtttaatgtcggtttgcatggcccctgcagtgcaagcgccgctgttaataagatccggccgcgtc

tggagaaccatcctctctcttctctagtaaaacgttg

>Hypothenemus_hampei_contig_2026|c0_g1_i1 -like periplasmic divalent cation tolerance

tttggtgtccacagtggcatatgcatttgtgctccacacgctagccataagaccattattctacagcccatcgaccaccg

aggacgtcttaatcatcctctcttcacttgtgttattcgtcttcttcctggtccatggcgccctaggcattgaaggaggg

aaaagaaagcccatgtaggcacaccagccaggtactggctcgaatccccttcgaacccggatactatggctggcctgctt

caagcgtggatctcatccactccaagtacggcttggatacgaagtggacctccacgctaattatctgggggatgtcgtaa

ggatgacccaaggctatatagtcctcgagcttgggcaataaagaagcgaatgtctttatggccagcttgtactcggtctc

ctgtgccacctttccttcccaaggatagatggatgttatttcagatatctgggagcatacggc

>Hypothenemus_hampei_contig_2037|c0_g1_i1 hypothetical protein ECU04_1540

gagcagcccaggctagcgtcgcggttctacacgtttctcttcccatcatttgttgttagagacggggggcgcagctacca

tctgcctgtggacacgtttgaaaggcttgaggagattataaagaagagggagtggggaatgttcaaacccagaaggccat

ggatggatgtggactcaaggctcatgggggtgtactcgatcgcaaacgacgcctttttccgagtcatgcaaaggtcgtat

gtggtttttgatgcggttcctggctgggcgatcaaccttgggttcagtgcgatcattgtctatgtggtgtactctatatg

cactatatttacagt

>Hypothenemus_hampei_contig_20388|c0_g1_i1 alanyl-tRNA synthethase

gaagggccgaggagcttgatgcagaggcagaggagtatctgacgcggctcaggggcggggaggtgatcaacgtggacaag

acgattcctctgttctacagacagaggattgaggcgctgaaccaggagaacaagaagaagggggagaggctcagcaaggc

tcagtacgacacgaacaggagagaggtggtcgagatgatatcgagtgctggtgcaggcgccgggaaggatgtggtagtct

acttctaccaggcgacagaggcatgtggcaagagggagctggtcaagtacatagggctgctttgcaacgaggcaatgaag

agggacgtgcaatgcttcatccacgcgacagccaatggagagcacctggtggcggccagggcatccgacaacgaggagct

gatggggcggcttgcctcgaagtatgcggacacgcatctgaggatcagcaaggggatggtgcagggaagcatgcgctcta

tgcctcggcaagaagac

>Hypothenemus_hampei_contig_2039|c0_g1_i1 BINDS MICROTUBULES

atctctgggatagaggaccttgggaggggggttgctatctgccggctgctgtccatcattcacgaggactttccctcgaa

ctttgttcgcaaccccagtggcgagaccgactatctgaggaacatgaagatatgccaggggttctttatgtcgagaaaca

tcaagctttacttccccgtcgacaaacttgtcaagtgtaagatgcaggacaacctggaggttgcacagtggctggcaagc

tattatgctaaaaagatgggggcccggaggcccggagatgcttctgaaggaaggaaagaggccttgctcccggggcctgt

ggactcctctaggaagaagacacagggaagcagctccaaagccgtggaggaagaggatactgttgctgcatgctgcctgg

gatgcaaaaatgctgaggaggtcataagcgcaaaggacaatagaatcagggacctggaagacaaaattgcccagataaga

aatcaggcacttcaggtggaagtcgaggaccacggggccaaggaagagacgacggagcccaagagtagtccaggaggatt

tgatcacgaggaggttcagtccctactcatgacgtttgaaagggaaagggatttctacttcaagaagttattcacaatcg

agagatactttcttgacaacaaggacctcgacgagg

>Hypothenemus_hampei_contig_20390|c0_g1_i1 hypothetical protein

agccatgtcctggagacagccacgtccctaggattcgacatggttgtattccaggcaagtgcagaggccgagtccgtgct

tgctcttgggtcgatctgccgtggagccggggccgtagagtctgtcgagcaccatgtctcaagcacgggcaggtgcaagg

ccaagaacgcgctcggaagggtcagggcaaaggagatagggtactactgctacctcaacagagaggcaattcctcggggc

accccctccaggatgtccggcatggacctggtcctctcgaggtttgtcaggcggatggacagcaagaacagccttgcagt

gttcaacatcctgaacactgttaagaaggttgtgtcagacagggatgggattgatgcctcagactgaggaggtgaatcag

gtgtgagagagatggcaaaacagag

>Hypothenemus_hampei_contig_20426|c0_g1_i1 heat shock Hsp30-

ggtcgggtcgaacggcagtacaatgacacagcatccgacgccaacgaaggcgtcgatgagcccgccgatgacacctcctc

caacaagtcccttcaacccacggttgaagacgaagacgaattgaactcctcggtgactcctgaatccacggcatccactt

ctcatccggcagctgcaccaagcaagtccggctccccctgcaaatactgggcgttggagcgctccattggccaatttcaa

cgaacgttctcattcccaaccagagtggatcaagaagcagtcagggccagcttgagaaacggaattctctcggtcatagt

acctaaagaggccgctcctaaacttaagaagatccgagttgaataggtctctccggtctttgactcctcaatctacaact

gttttgcatgacgtttctggtggatatgaatagctcgaattggttgggaagcttctcttttctgtcttctgatttctctt

gttgtcttacaatcagcatgtacgaacagttacgccataaaagatc

>Hypothenemus_hampei_contig_20426|c0_g2_i1 heat shock Hsp30-

ggtcgggtcgaacggcagtacaatgacacagcatccgacgccaacgaaggcgtcgatgagcccgccgatgacacctcctc

caacaagtcccttcaacccacggttgaagacgaagacgaattgaactcctcggtgactcctgaatccacggcatccactt

ctcatccggcagctgcaccaagcaagtccggctccccctgcaaatactgggcgttggagcgctccattggccaatttcaa

cgaacgttctcattcccaaccagagtggatcaagaagcagtcagggccagcttgagaaacggaattctctcggtcatagt

acctaaagaggccgctcctaagcttctcttttctgtcttctgatttctcttgttgtcttacaatcagcatgtacgaacag

ttacgccataaaagatc

>Hypothenemus_hampei_contig_2043|c0_g1_i1 hypothetical protein

gatctcggcgcgtccgggtacacggcagggtacatcatagaggcgttcaagagcgagaacgtccggcttgcgctggcagc

aaggaacatagggaggatcaaggacaaggcgtttcctgtgtacgagtatgggatcgacaagatcgacgaggttgcgtcga

tgacgcgtgtcctgatcaactgcgtggggccgtactgccaccatggcgaggagatagtcaagtcgtgcatccggaacggg

acgcactacatggacatctctggcgaggtgtacttcttcgagctgatcatcaacaagtaccatgacgaggcgacgcgcaa

gggcgtgtacattgtcaactgctgtgggtttgactcgctccccgtcgacgtgggcgt

>Hypothenemus_hampei_contig_20440|c0_g1_i1 hypothetical protein ECU11_1960

tcatggaactagatagagtgaggcagcttcaaagagaaagaaaatggatgcagttctgcagccttataatcgattgcatg

gggtcctcgcccatggacaaactcctggacatctttctggaaagcgcggggcaggcccatcccaggtcttcgtgcgaagt

gtttctggcctttgcagagaggctctcccccgaggagggaataaaggtcctagcgattggggagaagataataagagagg

ctgtcctgtactccgagtccttcgaggtcgagaagatgctgctggacatgaggaagtgccttctgctggtcgagagaggt

gatctgaaggggatcgagaagaagctttttgaatggaagggcgtcaagatggccaggaatgtggacatgatgtacaatct

gcttgggtttaagctttacgagaaaatccagaacatcgaggttgcgttcccgtacctcctgagatacgtgcgggcgtcgg

agtccgaggaccttgtagacaccctggttgagtacgcaatgctaagcaaggagtttttcaacttcacgctgataacatct

ctgcccagcttcgagaagatgaagagcagcagcctgagagagatattcgtcatgttcagagagggtgacataagggggct

gaagaatagacacgataagtttactgagatattccgggacaggtccgagatagtcgaggaaaagatatatatgatagccc

taataaacatctgcttctcggagacagagaagaggatctccatcaagaaggtcgaggagttgctggagatttctaacaag

accgcggtttacatcatcctgaagtcctttgggctggggttgattgaggggtggatggatggcgagaaaggaattctcta

ctttgactgccttgtcccgagggcgctgcaaacgggagaaataaccaagttgaaggagaagttcaacagctgggagcaga

gggtgcacgaggtcatttcgatgctaaagtaactaaatatcctgt

>Hypothenemus_hampei_contig_20463|c0_g1_i1 translation elongation factor 2

aggtgcacgatctgatgacgaaccagaagaacatcaggaacatctctgtgatagcacatgtggaccatgggaagagcaca

ctgacggactgccttgtgattaaggcgaagattgtgtcgaaggactctggcggaggaaggtacatggactcgcgggagga

cgagcagcagagggggatcacgatcaagagctctgcgatttcgctgcacttccaggtgcagaaggacgttctggaggcgt

acacgaaggcggaggacacgaacgggacggagttcctgatcaacctgatcgacagccctgggcacgtcgacttcagtagc

gaggtgactgcggcgctacgggtgaccgacggggcgcttgttgttgtggactgtgtggacgggatctgtgtgcagacgga

gactgtg

>Hypothenemus_hampei_contig_20477|c0_g1_i1 karyopherin beta

ctcggaggccacgcacagagacctcaagcccgtgatcctgtctgtgtttggggacattgcactggcgctggagaggaact

tcgagatgtatctggacatggttgttatgctgctgcagcagatttcggagctcaacaggcggcccgacgaggagtatgtc

gacgagcttcgcaagaacgccctccagctcatcaactgctcgctggtggccattggcgacagctccagggtcagggccag

cttgccgcgcatcatctccatagcgcacaagatcggtgccgaggacacggaccgcaaggtgcacgaggatcttcttgggc

ttgtcgacgaccttgtcgggatgtatggaaagaacttcgggctggacgagctgtggattaaggacttcctgtacggcatg

atgaagttcagcaacgacaagaatagaaagagggccagccatacgctggagatgctaagatga

>Hypothenemus_hampei_contig_20488|c0_g1_i1 DEHA2G04488p

ctattaaaaccaaacaagagttcactgatgctttagcacacgacggtttagtcgttgttgattttttcgctacttggtgt

ggtccatgtaaaatgattgctccattattggacaaatttgccaaagaatatgccactgctaaattcataaaagttgatgt

tgatgaatttggtgaaattgcccaagaatacgaaatttcttctatgccaactgttttattcctcaaaggtggtaaagttg

ttaccaaagttattggtgccaatcctccagctttgaaacaagctttagctgctaatgcttagaattgatatcgaatcaat

ctattacttttatttctaacttaattgtgtacctttttataaaattgaataaaaattc

>Hypothenemus_hampei_contig_20496|c0_g1_i1 hypothetical protein ECU09_1580

tccgttcaagatcaaaggagtgcgtccgagaatcagaatggatgcccgggtgtctcggaaggaagaccttgtctcgttta

gtatcgagtacgacatggatcttgccatagacaatgccgagctcaagacaaaggaggacgccgggaaggagttcttatat

gtatacaagtttgcagatctggatggcgctgtggagttcatggagagtggacgcgtaagagctcttcagggaggaaagcc

gctggatgttgaaaaggtggaaagagagatggacgtattcatggagaagtatgaggctggggagagggagctcaagaaga

agaaaaagacgcttgttgttggggaggatgggttcatgaaatacgtgtgagccgcaatggcacttgggaaggaaacagtc

tttattg

>Hypothenemus_hampei_contig_20509|c0_g1_i1 60S ribosomal L5

aggccgttgagtccgatgacggcagcagccgccgccccttcaaggccttccttgacgttggtcttgcccgtacctccacc

ggtgcccgtgtcttcgctgccatgaagggtgcctccgacggtggtatctacgtcccccactccgagaaccgcttccccgg

cttcgacatcgagtccgaggagctcgacgccgagactctccgcaactacatcttcggtggccacatcgccgagtacatgg

agggtcttgccgacgacgatgaggagcgtttccgtggtcagttccacaagtacgttgacgctggcgtcgaggccggtgac

attgaggagctctacaccgaggcccacaaggccatccgtgaggaccccttcaagaaggatgaggaggccgagcccaagaa

gaccaaggagcagtggaaggccgagagcaagaagtaccagcagaagaagctttcc

>Hypothenemus_hampei_contig_20523|c0_g1_i1 mannose-6-phosphate class I

gtgggcaaagcgaccttggagaagagcggccctgagatccctttcttgcctaagatcctctccttcgccaaatccctccc

tctccaaatccaccccgacaaatccctcgccgagaagctccacgccaaagaccctcataaattcggcgacagcaaccaca

aacccgagatcgccatcgccctgtcccacttcgagctcttcgccggcttcaagcccttcgacgagatcgccaccatcatg

cacctggccccattaacccggtacctgccctcccaatccaacccatcatccggcggagaaacctcacccgaaatcaacgc

ctccaccctccgcacaatcagcaaaaccctcctctccctgcaacccaacatcgtc

>Hypothenemus_hampei_contig_20527|c0_g1_i1 similarity to HYPOTHETICAL PROTEIN YQBP_BACSU

cagcgtgctaccagatataccagggtcctgctcagcagccgctttgcgcaacattctcccagccggtggtgacgacaggc

gtcgtgccgcagaggcccgggatacagtcgaggctcagccccgaccttgtgaggctgatccagtcgatccccggtgcgtc

cgagaagctctcgagcttcctggagtacaagggcatcacgcagccatggccagagatattcgacctgagctcggacaggc

actacgggctccctgccccgcagccgatgttcgccaagatccaatcgctgatgaacgtcctcgaggacatcaaggagctg

ctgtccaacgagctgttcaaggtcgagacatacataagtagagggaaggcacatctgtgcgagagcatcaagaggctcag

aagggacgacgggatcctcaagaagtatctgaagaagatatcctctgcaagaacagccaagtccaagcacaactacgcca

ccaagtacacggagttcagcgagaaggcccatgaggaagccgagtcgttctacgagacaaagaaatggctgcagaccata

gtggcactgttcaggtcgctgatctagtgcacgtcctggacatgtactcatggattcccaaaggcagagcacagcccccg

ctttccccgaagg

>Hypothenemus_hampei_contig_20537|c0_g1_i1 60S ribosomal L5

tggtgctaaaatctttggtgtcttaaaaggtgcttctgatggtggtttatacgttcctcactctccaaacagattcccag

gttgggatatggaatccgaagaattggatgctgaattattaagaaaatacatcttcggtggtcacattgctgaatacatg

gaagaattaatggatgatgatgaagaaaaatacagacaagtcttcaaaaactacatcgatgaagaaattgaagttgaaga

tattgaagaactttacaccaacgctcacgaagctatcagagctgacccagctttcaaaccagctgaaaagaaattcacca

aagaacaatacaaagctcacggtaagaaattcagacaattcaaactctctaaagctgaaagagatgctagagttgctgaa

aaaatcgctgctttcaaagctgaaaagaaattcaccaaag

>Hypothenemus_hampei_contig_20541|c0_g1_i1 hypothetical protein Eint_111960

gcgacggaagcgttcgagttgttcgtggttggtccgaggtcgaggtatctttacagttcaaggagacgacagagacccta

attgctcttggtataacccagagaggcgatgattcgggcgacgaatccccaatagatgccagcaaagttgaaattcccgt

agccatcaagtccatgattctggggttcctagccccttacagactagggataagaatagatctcaacgtggtaagcaacg

atggaaacgtgtatgacctcttctttgccggtctcaacgccctgctcggggatcttgaaatacccgttgtgaacgacctt

gggaggaccgagaggagagggctggacctcccgctttcaagaacagccgctctatttgacaatgggggctttgtggttga

tccgacaaggatcgaggaaaaggcatcatgcggactgatgcatgtatttgttgacactaaggggatgctaatgggatgcc

ttccagagggccaatggaacacaaccgaggagacgctcgccgcaactatacaggagatgatttc

>Hypothenemus_hampei_contig_20553|c0_g1_i1 GPI anchored cell wall

cactattggcttcggccaccgctgtcaatgcccttgttagccgcggcggcagttgctgctttcaccttgatgcctctggt

ggagcttctggctctgttggtcaattgagtgatggtcagaaccgaattggggatgatagtctccagcctgcgcagttctg

catcgattcaaacggtgccatcactgatggaaatggccgtggatgtatcctgactcccccgaccactcaattccaatgtg

atgaaggtgccactcccactccgggattctccgttggatctgatggtcaactggagttccacggcagccctgattttgtc

gcctgcgagaccggccaaaatggcgggctgaacatctacaccactgagagcaccgatctcagcagatgtaagaagat

>Hypothenemus_hampei_contig_20564|c0_g1_i1 similarity with ornithine decarboxylase DCOR_YEAST

gtggacagcaactatgaggtggtggaaatgattgaagggaagttcatggacgttgctgtgtctccggagacagtttatgc

catgggcttcgggggcgacatagttgtgctgacgcgcgacctgcaggttctgaggaaggcgagcttcgaagacaagtttg

acttccggtccatatactttgccggagacagggtgtttgttgggacgggaatggggatgaaggtgtttgacaaggacatg

aatcctgtcaaggagcttatgaatcttaaggacgagcccacggggtgcatagagcacggggagtatgttgtatatgggtc

gaagtatgacaactctctgaagataattcttcctggcctgcggtgcttcaa

>Hypothenemus_hampei_contig_20571|c0_g1_i1 CDP-diacylglycerol insitol-3-phosphatidyltransferase

aacatcccaaacaccgtcggatacttcagaatcgctctcctggtcctgtcgacattcacatctggccttgggtttattgt

gttgtatggtctatcctccgccctcgacttcttcgatgggcatcttgcaaggatgttcaaccagtgctcacagctaggat

cgtgcctagacatgataaccgacagggtttccacagtggtgatatccttgcggatcatccatgcaaagccggagtactcg

cagttcctaacgctttacatgatattcgacctggtctcgcacttcatatacttccacatgtctgcgctgtccggaaggca

tcacaagagaacaagcaacagggtactgaggatttactacgacaagcgggtccttgggccgatctgcctgctgagcgagg

tgtttttcatgtatgtatactacatcaaccacagagggatacttttgtatgtccttacagggattgcaactgccaaggcc

gtgttccacttggcccaactctcagaagctatttgtggcatgagcgacacccatagcgatgggaaatgacggcattaaac

ca

>Hypothenemus_hampei_contig_20579|c0_g1_i1 40S ribosomal S4

acttgaagcggacgacggcgccgaagtcgtggatgctggacaagctgggcgggacgtttgcactgatgcctgccagcggg

ccgcacaggaagctggagtgcattccgctggggtatttgatctcgcggttcctgaggtatgcaagcaatgcaaaggagct

tgtgatcatcctgcgcgggaagaacatcaaggtgaacgggaaggtgaggactgactcgcacttccctgtggggctgtttg

acgttgtgtcgattgagaagactggggagcacttccgggtgctgtacaacgtggcgaagaagttccatctgcacaagatc

agctcttccgaggcggggtaccggcttgcaaaggccacgaagaagtacgaggaccaggggatcccgtatgttgtgtcgag

ctgcgggctgaatatccgattctgcgacccatctattgtgcttgggagcacgatcaagatctcgaacgagacgggtaaga

ttctggagcacaccgtgcctggggccgacaaggttgtgtttgtgtcgaagggcaactcgcgtgggcgtgtcggggtgatc

aacacgatcagcacgcagggcaaggagacgatctacgggatgacggatcttgcgggaaatgcgttttcctgtacgagcag

gaactgcattgtgattggcgagtccgagcaggacatctggatcacgcttccgaaggagaggggaatcaaggtctctgagt

ttgagaaggtcaactccaggctgggtgagatggttgacacagaggttgaggctagcgaataaaac

>Hypothenemus_hampei_contig_20582|c0_g1_i1 glutamine--fructose-6-phosphate transaminase (isomerizing) partial

cgataaaggcatttaccgtcactacatgcagaaagagatctacgaacagccgaacgcgatcaaaaacacccttaccggac

gcatcagccacggtcaggttgatttaagcgagctgggaccgaacgccgacgaactgctgtcgaaggttgagcatattcag

atcctcgcctgtggtacttcttataactccggtatggtttcccgctactggtttgaatcgctagcaggtattccgtgcga

cgtcgaaatcgcctctgaattccgctatcgcaaatctgccgtgcgtcgtaacagcctgatgatcaccttgtcacagtctg

gcgaaaccgcggataccctggctggcctgcgtctgtcgaaagagctgggttaccttggttcactggcaatctgtaacgtt

ccgggttcttctctggtgcgcgaatccgatctggcgctaatgaccaacgcg

>Hypothenemus_hampei_contig_20591|c0_g1_i1 proteasome subunit beta type-1

aggtctagtagcagatcgagcctgggggtgcttcagaactatgatctgtttgaggacgtcactgatagcatggagccatg

gcatgtgttcgggccggctgctaggaggcagaaagatggttccaaggcagggactgtgagggattttgcagaaggtttgt

ttacgggcaagagagccgaaggcacgagggtggaggacggggagagattcaatccgtacgaggataactcgggaaccacg

atctgcctgaagcaggacggcttcattgttgttgcaggggacacaagacactcttcggacatggtcatcaactccaggga

gatgtcgaagatattccgaatcggggactttcttcttaacgggacgggattctatgcagatagtcatgaggtctatgtga

agatggtgtatgagatgcggcagtacgagatcgaagggccgatgagtatccatagtgcagccaatcttctttcgaagacc

ttgtactccagaaggttcttcccgctgtactcgtactgtgctctgagcgggtttgatggaggagagccctatatctatgc

atacgatcccttggggtccttcaagctcgtgacatgcatatgtaatgggagtgggagggcaatgatccagccgctgctcg

actcgttcattgacaagaagaactggaataatgcagagagggcgccgataagccaggaggactgcatacggctcgttgcc

aaggcgttcggggcggccgccgaaagagatgtgaagacaaaggacaacctcgaggtctatgtgatgagcgagaacgacct

ggtgcacagggttattccgctgaggagagattaaagcttcttg

>Hypothenemus_hampei_contig_20593|c0_g1_i1 protease regulator

agaggcgcgtgcgtacaaggcccagaccatcctggaagctcagggtgaagtggcgcgctttgctaaacttctgccggaat

ataaagccgcgccggaaattactcgcgagcgtctgtatatcgagacgatggaaaaagtgttgggtaacacccgcaaagtg

ctggttaacgataaaggtggcaacctgatggttctgccgttagaccagatgctgaaaggtggtaacgcccctgcggcgaa

gagcgataacggtgccagcaatctgctgcgtctgccgccagcctcttcctccacaaccagtggagcaagcaacacgtcgt

ccaccagtcagggcgatattatggaccaacgccgcgccaacgcgcagcgtaacga

>Hypothenemus_hampei_contig_20595|c0_g1_i1 hypothetical protein ECU09_0490

cggatgttcaagaactaccagaacccgaaggggctttttcttgcatatgtgatgctgaaggactacgacggggcaatgag

ccttatgttcaagggcgacttcccgatgattccgtcgtacttcataatgaaggccctgtgccctaggtgcacgagcaaaa

aggtatttgtggactttgtgttcctggcggccacgaagttcacgtcgacgcagaggaaggtcgagctgctaagttctctg

aagcacaccgtcgacgggtactacgagatagttcccgaaatgataatcaagatgggcctgtacgacgtgcttgggatcga

ggacgggacctgcttgtacctggacaagaagattaaccagagggtgatcgagattctgaaagggaagaacgagaagaaga

agcttatcaagctgtactacctgattgatgacgaggggctggttgt

>Hypothenemus_hampei_contig_20610|c0_g1_i1 hypothetical protein ECU08_0420

tacaggaggagtgcatgcgatacaaaggggatgtggagagaatgagcgtggagatggctgaactacgggcaaggaatgcg

gagagtgctagagcgattgaagagatggacaggagcaaggccgagcttgaggaggagatgaaggggattgtgaaggagaa

caaccggctggcgaaggagaatgtttgcctgggtgagctcagaaggtcgcttggggagaaggatgagatcatatctgcgc

taaaggagaacatgaggcagaagtctgagatgattgagatgcagaagaggctggctgagaatgcttctcggaaggccaag

gttgtgatgagcgaggatgtgtgcaatgtgtttgatgacgagatgttgaaggcggaggacagcgggaatgggctagatct

ggacgagtacggcggcgcccggaccgaggagcgtctctgggacgacattcccatcaagccacgctctgtaggccggaaga

ctacaggcactgctggggccaggagaaagacaggagtggataaaggagagaaggtgcccaagagagggccaaagacagtg

gctgcaggaacaaatagaaacaaagaaaacataggcaatgaaattggggggaaatcatcagcagccagtgcgacctctcg

agtcgccgaacctcccaggatatttgctccaggaggcctcctcaagccggagaactcctcgtactttgccgacttgacat

tcaacaactcaagccctgtgatcaagaaggaccggcttgacttgcccaaaaagaagtagacagactaatttgaagtaggc

tagtgtgtttgattaaaccgacgtgtgcccatg

>Hypothenemus_hampei_contig_20617|c0_g1_i1 60S ribosomal L44

gatttgatgccctgtgctgtatggcggcgtgtctgcggacccatgtgccgcctggcgagacgggcatggccagcaaaaaa

ttgctttgtcggggcccttggaaagatggtgaacattccaaaggcaaggaacacgtactgcaagaagtgcagcgggtact

ctgcacacaaggtctcgcagtcgaagaagtccaaggacaacccgagggcacagggaaacaggcggtatgcagcgaagcag

agagggtacggtgggcagacaaagcccattctcaggaggaaggcaaaggtcaccaagaagcttgtcctgaagctggagtg

cacgaagtgcaagtttgcccaccagaagcccctgaagagagccaagcatgtgatatttggcggggagaagaaggtgaagg

gagaggcgctggtttattaaagatctgcttctatcgtgcacggcatttattggcccggagaccccc

>Hypothenemus_hampei_contig_20623|c0_g1_i1 NADH-ubiquinone oxidoreductase subunit GRIM-

gtctactacctcctcggaatgcacatgttcatggcctacggcatgtacaagctcttctacggtatccgcgaacaaaacga

actcgcccgtgagaagatctggggccgtctccacctggtccctctccttcaggccgaagaagaccgtgaccaggtccgtc

gctactatgccgacaaggcccgtgagaaggagcttctcggatccgagaccaaggtctacaactctgaccgcttcgtccgc

cctacttacgcctacatgccgtccaaggtcactcaataagcgactggaattgcaatgggcttgggctcgtttacgtttat

tctcgcttctttcattgagtgtgcgatgcgtgaaaccctgatcttatacggatcttgggtgtcaaaaaagaaggtcgaat

gctccgacgcggatttcttcgaaacaggatagaagagaggtaccaggaaagtggaaagcagagttgtatgatgtagatat

accgtttgaaagaaattaactagtcccgcaatctatgctgttttgttgttttct

>Hypothenemus_hampei_contig_20636|c0_g1_i1 thioredoxin reductase

gactgccgtggacaactacccggggtttcctgacgggacccagggccctgtgctcaccgggctgatgaaggagcaggctg

ttgggcgtgggctgcgggtggttagggagacggtgtctgcggtccgcagggaggaggcgagctttgtggtttgtagcgag

agtggggagcatcggacaagggttgtgattgttgcgacgggggcttctgcaaggcggctctttgtgcctgggaccggaga

cgacgagttctggcaaaggggggtgagctcgtgtgctgtgtgcgacgggtttgcttatgtcaagaagatcacctgtgtga

ttggcggtggggatgcagcgatggaggaggcactgtatctgtcgaacattgccaagaaggtttatattatccaccggaga

aatgaattccgcgcaaggagcgacatggtcgagaaggcaagggccgcggagaacatcgagataatgacgccctatgtgct

cgaggaggctgttggggggcgcagggtggaggagatcaaggtcaggaacgtgtcgacggacgagattcgcacggtgccga

tgaacggggtgttttttgggatagggcacgatccgaacacgtgcttgctgaaggacacggaggttgagctggacgccaac

ggatatgttgttgtacgggatgaggtgtgcacaagcgtgccggggctgtttgcagccggggacgtgtgcgacaagaggta

tcggcaggccgtgacggcagcggcgtctggggcgattgcgggtttgctttaaaaatcatctttgaatggg

>Hypothenemus_hampei_contig_20642|c0_g1_i1 UBIQUITIN CARBOXYL TERMINAL HYDROLASE

ggcatagaccgctgctacgaggacgtgtgcctggacggcaaggacgcatggagatgtgacaggtgcggcgagaagagggg

gtcgcgcaagaagacagaggtgcttgtgcacccagacgtcctgatcgtccacatctcgaggttccatccacagggatgga

agaacacggcaagcgtcgaggtcaacgacacgctgtgcttcaacaatagaaaatacagcctcttcggggtgatctgccag

aacgggaccctgagcggaggacattactttgcagaggccaagaggtccgggacatggaatctatacaacgacgagagcgt

gacaaaggacttccacagctacaacgggtcgcacccgtacattgtgttctacactctttgacgccggattaaatccttga

cggactcctgggtgccgggccatctgatttgtttttgggtcttcaagggctctcccggatttgccagcaggcacgctcgc

t

>Hypothenemus_hampei_contig_20647|c0_g1_i1 hypothetical protein EROM_010060

ctgtcgatgacggacttcgaccggctggaggcgttcattgcaggcacggtcgactgcctgaggaacacggatgtcaacta

cgtcgaggtgcggtccgaaatcgacttcttcgttgtaaagaaaagcaatgccatccatctgtatgtcagggacctgatcc

acaggagggagaggaagttcatggagaggtttaatcggatgtttgagaagcttggggcacaggcatgactggatagagag

agagcagatatataataaacttaacccgcgtcctcggctgtcgaacatgttcttgggggctggctggcctgcacggtcct

gatagatcg

>Hypothenemus_hampei_contig_20671|c0_g1_i1 TATA binding associated factor 4

gtggatgagtgctgaggatgtccgtgtgaacgaggagagcacggcattcaactcgatctactcgccgtttgacgagaagg

ccttggaaaggaaggtgctgaacaggacaataacgatgaaggattttctgtatgtgcttgagagggaccggaggtataac

aagtcgatattcaccatccagcactacttcaagtagggtttctgagtggatgagccccctccatgtgtatttgctgcggc

tataaatggaactagggtgcgagggagcgaaaaacaacgcggagaatggacgacgagagtttgccgaagttcagggcgca

gacagagcttctgtcgagcaaggagattgagagggctgtgaagaagctaaacaaggaggaggcagaacggtttatagaga

ccattgagcagtacaatgacgagctgaggagggagcttgagcgtgcaaggagtgatcttgaaagaagagtagaaaaccac

aaggtgcttcttgaggatgtgcgggaggcca

>Hypothenemus_hampei_contig_20675|c0_g1_i1 TPA: heat shock 70 kDa (Eurofung)

atcacaatggccgactctgaggtctacgacggggcgatcggtatcgatcttggtaccacctactcttgtgttgccaacta

tgagggcaccaacgttgagatcatcgccaacgaccagggtagctacactaccccctccttcgtctctttcaccgacaagg

agcgtctgatcggtgaggccgctaagaaccaggctgctatgaaccctaagaataccgtcttcgacatcaagcgtctgatt

ggtcgccgtttcgatgaccccattgtcaagaaggatgttgagtcctggcccttcaaggtcgttgaccagggtacctcccc

tgccgtcgaggtcgagtacctcggtgagaacaagaccttcactccccaggagatctcctccatggtcctcatgaagatga

aggaggttgctgagaccaagctcggcaagaaggttgagaaggccgtcatcactgtccccgcctactt

>Hypothenemus_hampei_contig_20685|c0_g1_i1 E3 ubiquitin ligase subunit cullin

agctgtacatgatcggggacgttggaaaggccgagaaggacatccgggcgttctactgctgcgaaagcgtctcgatggct

cacgaggtcgacagaaatgtatattaccagtcggtggtgtcgggcatactgaagaggcgcaagaagatgtgcatggagga

aatctcgaaggaggtcctttcagcccacactgagcagtttgagtacgatgaggacgtgctgcaggagggcatgagggctc

tctgcgacaagggaatcatcgaggatgtggagaatgtctatgtgtatctcccctagggcaggatgtatatgttgcgaggc

agaccccgggatggaggcgcagggacagaagacaaaggaggagtatttgaaggagaag

>Hypothenemus_hampei_contig_20689|c0_g1_i1 SNF2 DNA RNA helicase

tgctgaagcacaaggacgacgtcgaggagtaccacaaggtgttctgggagagggttggggagctgagagatgcagagaag

atcctggggtccatcgagaggagccagcggcggatgaagaagaagtccacgatcagagagatagcagaaggagatgtgga

cgagattgtcgagaggctagggaagtttcgggccgggatcggggactacaacaagctcctgttgaccctgtacagaaaac

atttggatcgtcccgactgggcgctgcatgtgcgcagagacatcctggagtctgccgaccacaggttcgattactacctg

ctgtccaggaccggggcggacatccagaagcacattgggcaccttgttgctgtgctggccaaagatcg

>Hypothenemus_hampei_contig_20707|c0_g1_i1 hypothetical protein CLUG_00565

ggtaccgacgaacaaccttctccagtcatgtattggttgatgaatcaaataaattaaccattgctccaaccccagtcgaa

ggactgactgggtttggtatcgaggattctttattaacttaccaaaatgtatctacttttgttgcttgtcctgatgagaa

cttcagaggcgaatattctatttattggaatactgattgtcctaatcaagctcaaggttattccattgagttgttagtcc

aatccgatgcaactgttaattataaccctgatacaaatcagtaatttctcaatttactaattctctattcactcattacg

attaactcattacgattaactcattacgattaactcattacgattaactcattacgattaactcattac

>Hypothenemus_hampei_contig_20743|c0_g1_i1 inverse autotransporter beta-barrel domain-containing

gaacactatggagtgaatggggttatatgaagttttttggttggaacattgatgacatgtactggacaaatgatactgat

tcagagggttatcattatttaaccctcttggaatatggtagtcttcatagtggtacttatgcgcaagacgtgggaactag

atacgttatgtgtagcaatgaaattccatcagatgtaataactgttactgcgcatggtcatattttttatgttgagagtg

gttttccaacaacaggattcaaaggagcaaattttaaaataaacatacataaagacaaaaactttactttttcatctaat

caaaattggttgggggttaatagtgatggtatcgttactattactgcacaacctaacgccggaaatactgatgcgttaat

aacaattacgtctgaatcagatgatgaagtgttatcttattcatttaaccttgactactggtttgttaatgcagggaaac

agcgatattcgatagcagaagctgaaaagtattgtgccgatatcggactctccttgcccccctataagatagttaccaat

gctgataatgtgaaacaaacaggttacagagatctcggaacactatggagtgaatggggtaaccttgattttttcgggtg

gggtaattatgtaagctactggacaaatgatactggtccagagggctatcattatatagtcgacgttgttagtggttatc

tttttaatgaggctatttctggatttgactccaatagtttatttattacatgtagcaataaattgcaacctgcctcagat

gtaattaatgttattgcgaattaagtatcgcaaaaaatcaagtaatgaaatcgatctcgagaagtacacctggtcagaaa

acaaagttttagcaagtaggttaaactaattattgttatgataagcatctattgtaaaagattaccattttagttgcagt

aattaagggggtcactctaaagattttctttgtattgttctctaattctccatttcttgtaaaaatttcagaaaaggatt

ccgcctgtgtgaaatttgttcatttctattcactttataaatagacatatcggtacctcagagcaaatgggtaggtacta

acttgtaagtccaaaatttgaattttccgttttttttattctgatttctttatattacgtgtatgaggtccaatatggta

catacatcatgaggggcaaaactatattacatccaaaaaacgtattaagcccacatactgcaaattaaataattttaatg

ttctctctcttataaaatttacacacttttgctctgatttgctctggatttaatacacttttgctctgaggtatcgatat

gcatattaatagggactattttctagtattcatttattttatttggcgaaccacttattcaaactctagacctactcaaa

caggctaatttggattggaacccattttcaagaagagtcatcaaattacgtggctagctttatatcatagttgaaagatc

tctctatacggtttctcaaggctgtggtaatttttcagattttgaagttttagttgactttgaaacaagcccatttgttt

attaaataaatataacttttactgtaaaaaaattcgaaaggggctttaatttgggctgcgatacgttctttgaaactatc

aataaagggaaattat

>Hypothenemus_hampei_contig_2075|c0_g1_i1 alkyl hydroperoxide reductase 1

caaccgataaaaaattagcatacgttccaattaccgctggtaatcattctcttacttcttgtagtggtccaaccgaattg

gacttggctaaagagttagctggtaaaaaagtggttattactggggctccaggggctttcactccggcctgtaccgaaca

acacattcctgattacttgaaacatattaaggattttaaggccaaaggtgttgataaggtaattgtcatcactgccaatg

acccatttgtcaatgctgcttggggtaaggccttaggatacgttgacgatgagaattactttattttcgcagccgatgtc

aatgctgaatttgccaaggctttaggtggtgatgcctatgctgtggacttatccaaggctggcttgggtaccagagttgc

tagattcacatcttttgctgaagatggtaagttgaagtatttggaatccgaagatactttggactacacccagatttcta

gtgccaacactattcttgaaaagttgtagttgtgtatatttacaatataaatg

>Hypothenemus_hampei_contig_20827|c0_g1_i1 26S proteasome regulatory subunit 8

aggtgctgcgtgtgctggagcagaagagggcggagctcaacagaagggttcggctgctccgggaggagatctcgattgtg

caggagcccagctcgaatgtcggggtggttgtcgagaagatggggaagatgcaggtgctggtgaagacgaacccggacgg

gaagtaccttgtgaaggtggaggcgggaataaactacgatgacctcaagcccggggtgcgtgttgccctgcgcagcgact

cgtacgacgtccaccggatcctgccgacaaaggtggatccgcttgtgtctctgatgatggttgagaaggtgcccgactcg

acgtaccagatgatcgggggtcttgacgagcagatcaaggagatcagagaagtgattgagcttccgatcaagcacccaga

gctgtttgagaaccttgggattgcacagccgaagggcgtgctgctgtacgggccgcccgggacgggcaagacgatgcttg

tcaaggcggttgcaaaccacacgaaggcgacgttcatccgggtcaacgggtctgagtttgtgcagaagtaccttggcgag

ggcccgaggatggtgcgggacgtgttccggcttgcgcgggagaaggcgccgtcgattgtgtttatcgacgaggtggactc

gattgcaacgaagcggttcgacgcgtcgacgtctgcagacagggaggtgcagcgcgtgctgatcgagctgctgaaccaga

tggacgggtttgaccaggcggcaaacgtcaaggtgatcatg

>Hypothenemus_hampei_contig_20942|c0_g1_i1 2 -hydroxyisoflavone reductase

agtccaaaaaaatattcctgacttccttagtgttaaaagtgatcattctaccaaaatcagagaattcggggttaaagttg

ttgatatttacacttcattatttgctattccagaatctgcattatacgaagtggttggtcaagttggtattaataaagaa

gaaaatactgctactctatatggaccacctcagaccaaagtagcaatttcatttctaccagatattggtaatgccgtcgc

ctcgcttgcttcaattgaaccactgaaattgcccaattccatccgtatccaatcggatgaagtttcttatgcagatatca

ttaacagatatgaagttgatcataatatcaaattgaaagtaaaccaaatcgatggccagcaaaccttgaaatcggcccag

gaaaaatatagtaaactgaagacttttggtcctggtgactttttcttttacttaacccttttcggtggattgggcactga

caatggattaagctactcttccaacgatgatgaattggttaatccaaatggccaattatggagctggtccaagttcaata

actaaaaacaccatccataagtatgcatttgccta

>Hypothenemus_hampei_contig_20953|c0_g1_i1 ubiquitin- ligase

cgatctccgacttctacaggtcgctcgtgtggatccgggacaacccggtcgacgagtctctggggctgacgttttccttc

gacgacgtggcgtttggggtccacaagacagtcgaccttgttaaaggaggctcgaccgtctttgtctccgactcgaacaa

ggcagagtacataaggcttgccacacagcacaggctcttcaacgggatcgagctccagctatctgcactgaagtcggggc

tatttgagatcctggggcccaaggcactggagatgttcgacgagaacgagcttgagctgcttatctgcggcatcccggac

atcgacgtcgacgactggaaaagcaacaccctctactacggatacacggaaagctcgaagacagtcatctggttctggaa

ggccgtcaagagcctggactccgtgggcagggcaaagctgctacagtttgtgaccgggacgtccacgctgccgttcgagg

gcttttcgcatctgcagggaaacagcgaggtgcagaagttctcgatccacaaggtctccgacagagtggacagcctgccc

acggcacacacatgcttcaaccagcttgtgttgcccgagtactcctcatacgagagcctcctgaggcgcctggtgttggc

aatcaacgagtgctccacggggtttgggttcatctgaataaatgttcaatgccgagatc

>Hypothenemus_hampei_contig_20957|c0_g1_i1 ETHYLENE INSENSITIVE 3-like

aatatctttgaagatactaatgtatccagcaaccgttctatttttcagcagggagatcgttttgaagatgctaacattcc

agctagtcgtcccatgtttcagcatggtgaccggtttgaccagtgcaagataacaagttctccattcaacaactccaatg

agaatttccagttcatgttcgggtcaccattcaatctaccttctgttgactttactgaaggcttgcctgggatttcgagg

gataatgcatcaaagcaggacctcccaatatggtaccattaagaaaagattagctgaaatgagtatcaagtagtcagtct

tggcaatcaatcaaagcaaagctcgttccggtgatcggagggttcatcagataataggtctatctgctctataaggtgga

tttttaatcgccagaatattatcttagtttgttgttttccttacattgtagttatagaa

>Hypothenemus_hampei_contig_21006|c0_g1_i1 Ribosomal S3Ae

aacgtcaccgtgaagactactgatgactacctcattcgcctgttcgccattgccttcaccaagagacgccccaaccagat

caagaagaccacctacgcccgctcgtctcaaatccgtgctatccgcaagaagatgaccgagattatccagcgcgaggcca

ccagctgcactctgtcccggctcacccacaagctcattcccgaggtcattggccgcgagattgagaagtccacccagggc

atctaccccctccagaatgtccacattcgcaaggtcaagctgctcaagcagcccaagttcgaccttggtgctctcctctc

ccttcacggtgagtcagccaccgatgacaagggccagaaggttgagcgggagttcaaggagcaggttctggagaacgttt

aaattgcgagacacgattagggaggtgtttcttttacgtgatgtcaattactggtcaatcatgatggcatttgttgtctt

ctgatcatagg

>Hypothenemus_hampei_contig_21006|c0_g2_i1 40S ribosomal S1

ttcgaccttccagaccagggatgttggaaaaactcttgtgaaccgcaccagtggtctcaagaatgcgaacgactctctga

agggacggattttcgaggtctctctcgctgacctgcaaaacgatgaagaccatgctttccgcaaggtcaagcttcgtgtc

gacgagatccaaggaaagaactgcttgaccaacttccacggactggactttacgaccgacaagctgcgatccctcgttcg

aaagtggcagtcgcttatcgaggccaacgtgacggtgaagacgactgacgactaccttcttcgtctgtttgcgatcgctt

tcaccaagagacgccccaaccagatcaagaagaccacatatgctcgctcgtctcagattcgcgcaatccggaagaagatg
[truncated: 1,426,540 more chars]
